# Identification and characterization of the gene expression profiles for protein coding and non-coding RNAs of pancreatic ductal adenocarcinomas

**DOI:** 10.18632/oncotarget.4233

**Published:** 2015-05-22

**Authors:** María Laura Gutiérrez, Luis Corchete, Cristina Teodosio, María Eugenia Sarasquete, María del Mar Abad, Manuel Iglesias, Carmen Esteban, José María Sayagues, Alberto Orfao, Luis Muñoz-Bellvis

**Affiliations:** ^1^ Cytometry Service-NUCLEUS, Department of Medicine, Cancer Research Center (IBMCC-CSIC/USAL) and IBSAL (University of Salamanca), Salamanca, Spain; ^2^ Cancer Research Center and Service of Hematology (University Hospital of Salamanca), Salamanca, Spain; ^3^ Department of Pathology (University Hospital of Salamanca), Salamanca, Spain; ^4^ Service of General and Gastrointestinal Surgery and IBSAL (University Hospital of Salamanca), Salamanca, Spain

**Keywords:** pancreatic ductal adenocarcinoma, gene expression profile, mRNA, non-coding RNA, EMT-like tumors

## Abstract

Significant advances have been achieved in recent years in the identification of the genetic and the molecular alterations of pancreatic ductal adenocarcinoma (PDAC). Despite this, at present the understanding of the precise mechanisms involved in the development and malignant transformation of PDAC remain relatively limited. Here, we evaluated for the first time, the molecular heterogeneity of PDAC tumors, through simultaneous assessment of the gene expression profile (GEP) for both coding and non-coding genes of tumor samples from 27 consecutive PDAC patients. Overall, we identified a common GEP for all PDAC tumors, characterized by an increased expression of genes involved in PDAC cell proliferation, local invasion and metastatic capacity, together with a significant alteration of the early steps of the cellular immune response. At the same time, we confirm and extend on previous observations about the genetic complexity of PDAC tumors as revealed by the demonstration of two clearly distinct and unique GEPs (e.g. epithelial-like *vs*. mesenchymal-like) reflecting the alteration of different signaling pathways involved in the oncogenesis and progression of these tumors. Our results also highlight the potential role of the immune system microenvironment in these tumors, with potential diagnostic and therapeutic implications.

## INTRODUCTION

In recent years, important advances have been achieved in the identification of the genetic and molecular alterations of pancreatic ductal adenocarcinoma (PDAC). Such studies have also shown that PDAC is a genetically highly-heterogeneous and complex group of tumors [1-6]. However, the knowledge about the precise mechanisms underlying the development and malignant transformation of PDAC, still remain largely unknown. In this regard, global gene expression profiling (GEP) at both the mRNA and the protein levels has proven to allow identification of distinct molecular tumor subtypes in many different human cancer types [7-9]. In recent years, many GEP studies have been also reported in PDAC [4, 10-24]; such studies, have mainly focused on the definition of molecular signatures associated with disease progression; but, to the best of our knowledge, only Collisson *et al*. [25] described (three) PDAC subtypes based on microarray analysis of GEP which were associated with different clinical outcomes and therapeutic responses: the classical, quasi-mesenchymal and exocrine-like subtypes of PDAC tumors. Currently, it is well-established that the cellular mechanisms involved in tumor genesis and progression depend, not only on the protein-coding GEP, but also on the expression profile of post-transcriptional regulators such as the miRNAs. Thus, simultaneous assessment of the mRNA and the non-coding RNA gene expression profiles may contribute to a better understanding of the molecular pathways of PDAC and a more accurate definition of the distinct molecular subtypes of these tumors. To date, only a few studies by Donahue *et al.* [26] and Frampton *et al*. [27] have combined global mRNA and miRNA expression analysis of PDAC tumors. In the former study, combined GEP data and DNA copy number alterations were investigated in a cohort of 25 primary PDAC tumors in an attempt to identify tumoral molecular profiles associated with a distinct patient survival. By contrast, Frampton *et al.* [27] analysed the impact of miRNA expression on the whole mRNA GEP in a small cohort of PDAC tumors (*n* = 9) and cell lines (*n* = 2) aiming at the identification of functional miRNA-mRNA interactions that could contribute to PDAC growth.

Here we evaluate the molecular heterogeneity of PDAC tumors based on simultaneous assessment of the overall GEP of both coding mRNA and non-coding RNA genes -including miRNA, small nucleolar and large intergenic RNAs- in primary tumor samples from 27 consecutive PDAC patients vs. non-tumoral pancreatic tissue. Overall, our results define a common GEP for all PDAC tumors, at the same time they confirm and extend on previous observations about the existence of two clearly distinct molecular subtypes of PDAC.

## RESULTS

### The global transcription profile of PDAC tumors

Supervised analysis of the PDAC GEP showed a total of 1,428 mRNA and 171 small RNA deregulated genes, with an average expression level ≥ 2-fold difference in PDAC tumors (*n* = 27) vs. non-PDAC pancreatic tissues (*n* = 5) (FDR < .0001; Supplementary Tables 2 and 3). More than half of these mRNA transcripts were up-regulated in PDAC samples (923/1428; 64%) while most small RNA transcripts (135/171; 78%) were down-regulated in PDAC samples. Among other genes, POSTN, SULF1, GREM1 and DKK1 mRNAs and the miR-203, miR-708, miR-31 and miR-4298 miRNA transcripts were those found to be overexpressed at the greatest levels, while the ALB, PDIA2, SYNCN, RBPJL mRNAs and the miR-216-a and miR-216-b, miR-217, miR-148a and miR-4286 miRNAs were those showing the most pronounced down-regulation across all PDAC samples analyzed (Table 1). ROC curve analysis based on those mRNA and miRNA transcripts differentially expressed in PDAC vs. non-tumoral pancreatic tissues, revealed a combination of just 5 genes (S100A11, GPR137B, SULF1, POSTN and miR-155) that allowed accurate classification (32/32 samples correctly classified) of PDAC tumor vs. non-tumoral pancreatic tissues (Table 2).

**Table 1:** Top 20 up- and down-regulated mRNA and miRNA and other small non-coding RNA transcripts in PDAC (n=27) vs non-tumoral pancreatic tissues (n=5)

Gene Name	Gene ID	Fold Change T vs Non-T	Fold Change GEP-A vs Non-T	Fold Change GEP-B vs Non-T	Chromosomal localization				Transcript description
					Chr. band	Start (bp)	Stop (bp)	Strand	
***Up-regulated mRNA transcripts***									
POSTN	ENSG00000133110	46.8	48.7	31.6	13q13	38136720	38172981	−	protein-coding
SULF1	ENSG00000137573	20.5	20.9	17.3	8q13	70378859	70573150	+	protein-coding
GREM1	ENSG00000166923	17.5	13.3	50.9	15q13	33010175	33026870	+	protein-coding
DKK1	ENSG00000107984	16.7	11.9	55.5	10q21	54074056	54077802	+	protein-coding
MMP11	ENSG00000099953	14.7	15.7	6.2	22q11	24110413	24126503	+	protein-coding
INHBA	ENSG00000122641	14.5	14.1	17.2	7p14	41724712	41742706	−	protein-coding
FN1	ENSG00000115414	12.9	12.5	16.8	2q35	216225163	216300895	−	protein-coding
THBS2	ENSG00000186340	12.3	12.1	13.6	6q27	169615875	169654139	−	protein-coding
SEMA3C	ENSG00000075223	12.2	11.1	21.4	7q21	80371854	80551675	−	protein-coding
GALNT5	ENSG00000136542	11.7	11.5	13.7	2q24	158114110	158170723	+	protein-coding
***Up-regulated small RNA transcripts***									
hsa-mir-203	MI0000283	43	39	75	14q32	104583742	104583851	+	hsa-miR
hsa-miR-708	MI0005543	25	26.7	NS	11q14	79113066	79113153	−	hsa-miR
hsa-miR-31	MI0000089	23.4	25.9	NS	9p21	21512114	21512184	−	hsa-miR
hsa-miR-4298	MI0015830	21.4	14.1	80.3	11p15	1880694	1880766	−	hsa-miR
hsa-miR-155	MI0000681	21.2	21.3	20.5	21q21	26946292	26946356	+	hsa-miR
hsa-miR-21	MI0000077	13.1	12.9	NS	17q23	57918627	57918698	+	hsa-miR
hsa-miR-503	MI0003188	11.6	12.4	NS	Xq26	133680358	133680428	−	hsa-miR
hsa-miR-10a	MI0000266	10.8	10.5	12.7	17q21	46657200	46657309	−	hsa-miR
hsa-miR-199a-3p	*	4.1	4.2	3.5	-	-	-		hsa-miR
hsa-miR-199b-3p	MI0000282	3.9	4	3.2	9q34	131007000	131007109	−	hsa-miR
***Down-regulated mRNA transcripts***									
ALB	ENSG00000163631	−28.9	−26.6	−90.9	4q13	74262831	74287129	+	protein-coding
PDIA2	ENSG00000185615	−23.4	−22.1	−43.3	16p13	333152	337215	+	protein-coding
SYCN	ENSG00000179751	−23	−21.1	−84.6	19q13	39693471	39694906	−	protein-coding
RBPJL	ENSG00000124232	−21	−20.2	−31	20q13	43935491	43945803	+	protein-coding
GNMT	ENSG00000124713	−19.5	−19.7	−17.6	6p21	42928496	42931618	+	protein-coding
PNLIPRP1	ENSG00000187021	−16.8	−15.1	−236.4	10q25	118349897	118368687	+	protein-coding
TRHDE	ENSG00000072657	−16.7	−16.5	−18.1	12q21	72481046	73059422	+	protein-coding
EGF	ENSG00000138798	−16.3	−15.6	−25.6	4q25	110834040	110933422	+	protein-coding
SERPINI2	ENSG00000114204	−16.2	−14.8	−72	3q26	167159577	167196792	−	protein-coding
ERP27	ENSG00000139055	−15	−13.5	−166.5	12p12	15066969	15092016	−	protein-coding
***Down-regulated small RNA transcripts***									
hsa-miR-216a	MI0000292	−68.1	61	−1250.2	2p16	56216085	56216194	−	hsa-miR
hsa-miR-217	MI0000293	−31.1	−27.7	−1834.4	2p16	56210102	56210211	−	hsa-miR
hsa-miR-216b	MI0005569	−26	−23.2	−664.4	2p16	56227849	56227930	−	hsa-miR
hsa-miR-148a	MI0000253	−15.3	−13.7	−189.3	7p15	25989539	25989606	−	hsa-miR
hsa-miR-4286	MI0015894	−13.7	−12.7	−38.52	8p23	10524488	10524580	+	hsa-miR
SNORA24	ENSG00000207130	−13.5	−12.5	−32.6	3q21	128433414	128433548	−	snoRNA
ACA24	ENSG00000269893	−13.1	−11.9	−58.1	4q26	119200345	119200475	+	HAcaBox
hsa-miR-130b	MI0000748	−13	−12.2	−31.1	22q11	22007593	22007674	+	hsa-miR
SNORA24	ENSG00000206903	−11.7	−10.7	−48.2	15q22	65577799	65577929	−	snoRNA
hsa-miR-148a-star	MI0000253	−10	−10	−9.9	7p15	25989539	25989606	−	hsa-miR

*q-values* < .0001; T: tumoral samples; Non-T: non-tumoral samples; GEP-A/B: gene expression profile subgroups of PDAC tumors as assessed by principal component (PCA) and unsupervised hierarchical clustering (HCA) analyses; hsa-miR: human micro-RNA; snoRNA: small nucleolar RNA; HAcaBox: H/ACA box small nucleolar RNA; NS: statistically not-significant; * miRNA transcripts with various annotated stem-loop sequences

**Table 2:** Receiver operating characteristic (ROC) curve analysis for genes previously selected by the prediction algorithms which better contributed to the discrimination between tumoral and non-tumoral pancreatic tissues (n=27 vs. n=5, respectively)

Gene name	Gene ID	Fold Change T vs Non-T	q-value(%)	AUC	SE	p-value	CI (95%)
*mRNA transcripts*							
GNMT	ENSG00000124713	−19.5	0	1.00	0.00	<0.001	1 - 1
GPT2	ENSG00000166123	−10.6	0	1.00	0.00	<0.001	1 - 1
KLF15	ENSG00000163884	−6.4	0	1.00	0.00	<0.001	1 - 1
CTTNBP2NL	ENSG00000143079	2.8	0	1.00	0.00	<0.001	1 - 1
MSN	ENSG00000147065	3.4	0	1.00	0.00	<0.001	1 - 1
**S100A11**	**ENSG00000163191**	**4.3**	**0**	**1.00**	**0.00**	**<0.001**	**1 - 1**
**GPR137B**	**ENSG00000077585**	**4.5**	**0**	**1.00**	**0.00**	**<0.001**	**1 - 1**
**SULF1**	**ENSG00000137573**	**20.5**	**0**	**0.99**	**0.02**	**0.001**	**0.950 - 1.020**
**POSTN**	**ENSG00000133110**	**46.8**	**0**	**0.97**	**0.03**	**0.001**	**0.910 - 1.020**
*Small RNA transcripts*									
**hsa-miR-155**	**MI0000681**	**21.5**	**0**	**1.00**	**0.00**	**<0.001**	**1 - 1**

AUC: area under the curve; SE: standard error; CI: confidence interval; genes showing an AUC >0.96 and fold change >4 that discriminate PDAC tumors vs. pancreatic non-tumoral tissues are displayed in bold.

### The gene expression profiling of PDAC *vs*. non-tumoral pancreatic tissues defines two molecular subgroups of PDAC tumors

**Figure 1:**
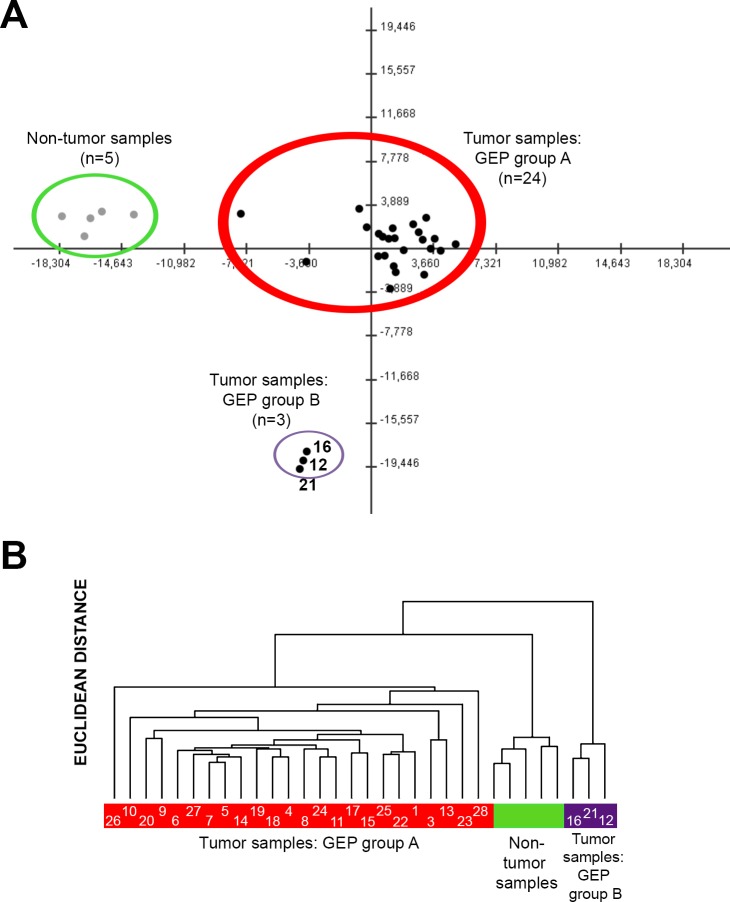
Classification of PDAC tumors and non-tumoral pancreatic tissues based on coding (mRNA) and non-coding (small nuclear and microRNA) gene expression profiles (GEP) Both principal component (Panel A) and unsupervised hierarchical clustering (Panel B) analyses differentiated tumoral vs. non-tumoral tissues (*n* = 5; color coded in green), at the same time they showed the existence of two major subgroups of PDAC tumors: GEP group A (*n* = 24; color coded in red) and GEP group B (*n* = 3; color coded in purple). Case ID of tumors are shown inside the colored bars.

Despite there were global differences in the GEP of PDAC vs. non-tumoral pancreatic tissues (Table 1; Supplementary Tables 2 and 3), both unsupervised PCA (Figure 1A) and HCA (Figure 1B), showed two well-defined subgroups of PDAC tumor samples with distinct GEP: 1) a major group consisting of 24/27 PDAC samples (GEP-A subgroup of tumors) and 2) a minor subgroup of three PDAC tumors which clustered together, clearly apart from the GEP-A PDAC tumors (GEP-B subgroup of PDAC). Of note both the GEP-A and GEP-B subgroups of PDAC tumors also clustered separately from the non-tumoral pancreatic tissue samples (*n* = 5; Figure 1).

Taking in account these GEP-based subgroups of PDAC tumors, supervised analysis showed a total of 2,594 mRNA and 214 small RNA altered genes among GEP-A and GEP-B tumors vs. non-tumoral pancreatic tissue samples (Table 1; Supplementary Tables 2 and 3). Upon comparing the GEP of the GEP-A and GEP-B subgroups of PDAC tumors: 1,605/2,594 (62%) and 181/214 (85%) differentially expressed mRNA and small RNA genes were associated with the GEP-A cluster, respectively, while 1,522/2,594 (59%) and 103/214 (48%) mRNA and small RNA genes were associated with the GEP-B cluster, respectively; a total of 533 (21%) mRNA and 70 (33%) small RNA transcripts were simultaneously altered in the two subgroups of PDAC tumors (Supplementary Tables 2 and 3). The altered gene profile common to the GEP-A and GEP-B tumors included increased expression of mRNA coding for the RAC1 and RHOC GTP-binding proteins, the insulin-like growth factor binding protein 3 (IGFBP3), several members of the S100A and the MMP gene families (e.g.: S100A6, 11 and 16, and MMP2, 11 and 14), as well as the PDAC-associated miRNAs miR-155 and miR-203, which are known to be typically altered in PDAC; in addition, both subgroups of PDAC tumors also showed loss of expression of normal pancreatic genes such as the CELA2A (pancreatic elastase), the CEL, PNLIP, PNLIPRP1 and PNLIPRP2 genes (pancreatic lipases and related proteins), the SERPINI2 serin peptidase inhibitor gene and the miR-216, miR-217 and miR-148 miRNAs. In turn, those genes which were found to be differentially altered in the GEP-A and GEP-B tumor subgroups, included, among other, the KRAS oncogene, the CEACAM1 and CEACAM5 epithelial marker carcinoembrionary antigens, the SERPINB5 gene, as well as the miR-21, miR-221 and miR-222 miRNAs which were all overexpressed in GEP-A vs. GEP-B tumors (Supplementary Tables 2 and 3).

Supervised analysis further showed differential expression for another 20 genes in GEP-A vs. GEP-B PDAC tumors (Supplementary Table 2). Among other altered genes, these included greater expression in GEP-A (vs. GEP-B) PDAC of the CEACAM6 gene, as well as of genes associated with the inflammatory response and chronic pancreatic diseases such as the integrin β4 and β6 genes (ITGB4 and ITGB6), the cytochrome b-245 beta polypeptide (CYBB), lysozyme (LZY), the SERPINA1 antiproteinase and the antitrypsin serpin peptidase inhibitor genes, together with genes involved in tumor metastasis and invasion –e.g. the MMP7 matrix metalloproteinase and the tetraspanin 8 (TSPAN8) genes- (Supplementary Table 2).

### Functional characterization of deregulated GEP in PDAC tumors

**Figure 2:**
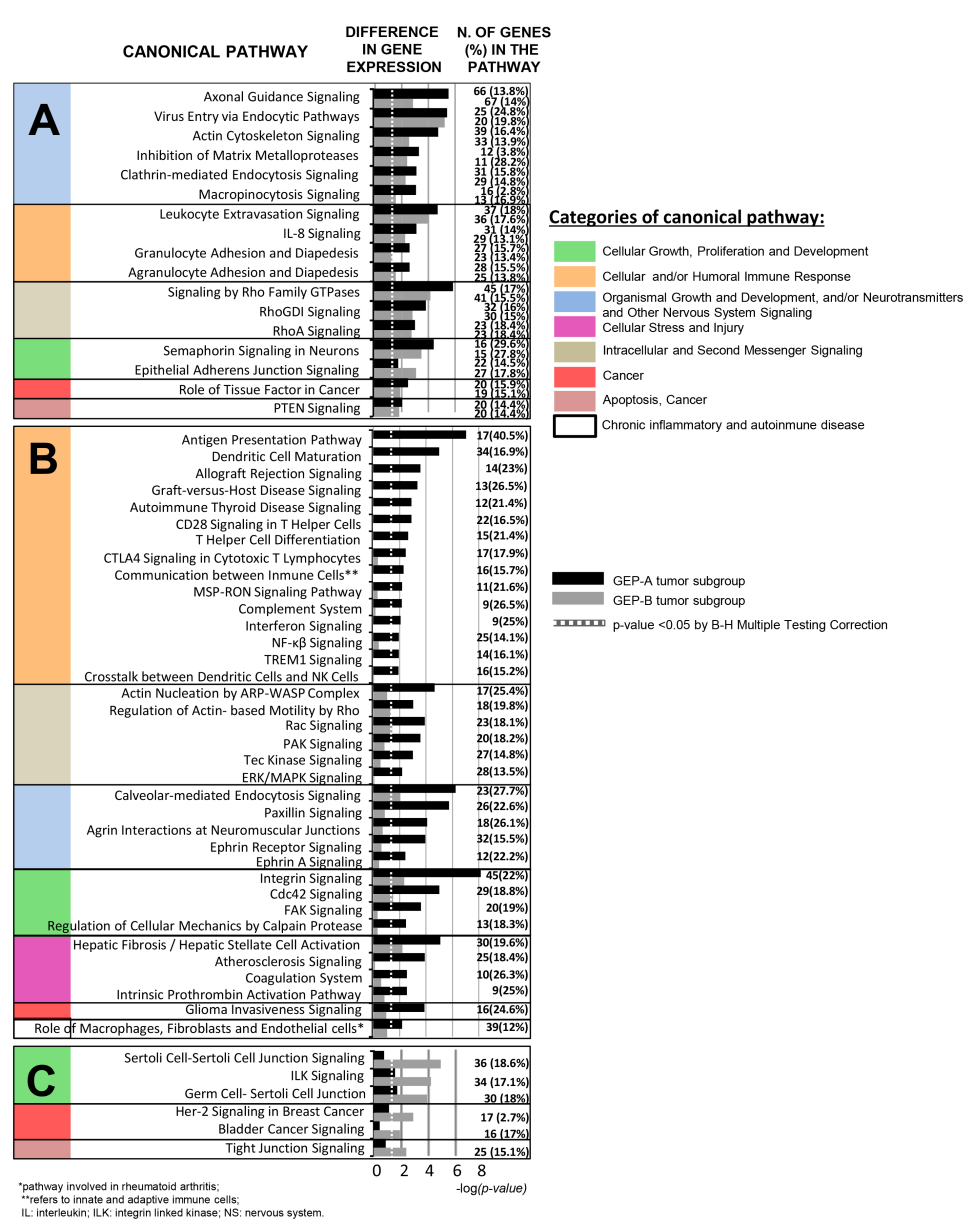
Most representative canonical pathways involved in PDAC tumors as defined by their GEP for both coding and non-coding RNAs (*n* = 27; *p* < .05) Shared canonical pathways by the two GEP-A and GEP-B subgroups of PDAC tumors are shown in panel A, while those pathways specific for the GEP-A and GEP-B subgroups of PDAC tumors are displayed in panels B and C, respectively.

Analysis of the biological and functional significance of the deregulated GEPs observed in our PDAC tumors, revealed > 55 significantly altered canonical pathways vs. non-tumoral pancreatic tissues. Among those pathways more commonly altered in PDAC tumors we observed increased expression of genes involved in axonal guidance, the actin cytoskeleton and/or endocytosis processes such as integrins (TGA5, ITGB1), GTP-ases (RRAS and RAC1) and actin-related proteins (ACTR3); in addition, genes that participate in the early steps of inflammatory cell responses, such as genes associated with leukocyte extravasation, cell adhesion and diapedesis, and with IL-8 signaling, together with genes involved in cell motility, were altered in tumoral tissues from both groups of PDAC, as reflected by an increased expression of the MMP2, MMP11 and MMP14 matrix metalloproteinases and the MSN (moesin), CDH11 (cadherin 11), RHOC (Ras homolog C) and CFL1 (cofilin 1) genes, in parallel to a decreased expression of the CLDN3 (claudin 3) gene (Figure 2A; Supplementary Table 4). Both subgroups of tumors also displayed increased expression of genes involved in bladder cancer signaling pathways and glioma invasiveness (Figure 2).

### Functional characterization of GEP differentially altered in GEP-A and GEP-B PDAC

Canonical pathways found to be deregulated in GEP-A vs. GEP-B PDAC (Figure 2B and 2C) included multiple genes involved in innate and adaptive cellular and humoral immune responses. Among others, these included interleukin 18 (IL18), several IL receptors (IL2RA, IL2RG, IL10RA) and the IL1RN IL-1 antagonist, the CD80 receptor gene, major histocompatibility complex class I (HLA-A, HLA-B, HLA-E and HLA-F) and class II (HLA-DRA, HLA-DMA, HLA-DMB, HLA-DPA1 and -DQB1) molecules, toll-like receptors 4 and 6 (TLR-4 and TLR-6) and both the janus kinase family members 1 and 2 genes (JAK1 and JAK2) and their signal transducer and activator of transcription 2 gene (STAT2). In contrast to GEP-B cases, GEP-A tumors also displayed an altered expression of genes involved in cell stress, injury responses and chronic inflammatory disease pathways; this included overexpression of the COL3A1 and COL10A1 collagen genes, the PLA2G7, 10 and 16 phospholipases, the APOL1 and APOC1 apolipoproteins and the PLAT and PLAU plasminogen activator-associated kinase genes (Figure 2B and Supplementary Table 5). Conversely, GEP-B tumors displayed a less altered GEP, which consisted of decreased expression of genes related with cell junction and intercellular adhesion -e.g. the E-cadherin (CDH1), OCLN (occludin) and CGN (cingulin) genes, and several members of the claudin gene family (CLDN1, CLDN4, CLDN7 and CLDN10)- together with increased expression of the ILK signaling pathway, due to overexpression of the ILK gene and of other genes involved in the ephitelial-to-mesenchymal transition (EMT) such as SNAI1, SNAI2 and vimentin (VIM) (Figure 2C and Supplementary Table 6). Of note, GEP-B tumors also showed a GEP which was associated with other key elements of the GEP signature of EMT; thus, they showed overexpression of the N-cadherin (CDH2), TWIST1 and S100A4 mesenchymal phenotype-associated markers, together with decreased expression of epithelial phenotype markers such as the CDH1, cytokeratins (KRT8 and KRT18), desmoplakin (DSP), the chymotrypsinogen B1 (CTRB1), insulin (INS) and GCG genes.

From all differentially expressed RNA transcripts, a combination of 63 mRNA genes overexpressed in GEP-A and 97 mRNA genes overexpressed in GEP-B tumors (vs. non-tumoral pancreatic tissues) allowed for a clear cut discrimination of these two subgroups of PDAC tumors (Supplementary Table 7). A list of those highly-discriminant genes which were found to be most differentially expressed (≥10 fold difference) in GEP-A and GEP-B tumors, with a power to classify them with a 100% accuracy, are shown in Table 3. These genes included PDAC epithelial markers (e.g., CEACAM5 and SERPINB5) for the definition of GEP-A tumors and the SNAI2 mesenchymal marker for GEP-B tumors.

**Table 3:** Receiver operating characteristic (ROC) curve analysis for the most overexpressed (≥10 fold greater expression) genes which contribute most to the discrimination between PDAC tumor GEP group A (24 group A tumors vs 3 group B tumors plus 5 non-tumoral tissues) and group B (3 group B tumors vs 24 group A tumors plus 5 non-tumoral tissues)

Gene name	Gene ID	Fold Change GEP vs Non-T	AUC	p-value	CI (95%)
***Selected markers for GEP group A of PDAC***					
CEACAM5	ENSG00000105388	58.1	0.96	>0.001	0.882 - 1.028
SLC6A14	ENSG00000087916	34.6	0.99	>0.001	0.951 - 1.019
CST1	ENSG00000170373	23.3	1.00	>0.001	0.999 - 1.000
TSPAN1	ENSG00000117472	22.5	0.97	>0.001	0.897 - 1.033
LAMC2	ENSG00000058085	21.0	1.00	>0.001	0.979 - 1.011
TMPRSS4	ENSG00000137648	20.9	1.00	>0.001	0.979 - 1.011
PLAC8	ENSG00000145287	17.6	0.97	>0.001	0.897 - 1.033
LCN2	ENSG00000148346	14.3	0.97	>0.001	0.916 - 1.024
ITGA2	ENSG00000164171	13.9	0.98	>0.001	0.937 - 1.023
GPX2	ENSG00000176153	13.8	0.98	>0.001	0.923 - 1.027
MUC13	ENSG00000173702	13.4	0.96	>0.001	0.883 - 1.037
CTSE	ENSG00000196188	13.0	0.97	>0.001	0.897 - 1.033
TMC5	ENSG00000103534	10.5	0.98	>0.001	0.923 - 1.027
SLPI	ENSG00000124107	10.3	0.97	>0.001	0.910 - 1.030
SERPINB5	ENSG00000206075	10.2	1.00	>0.001	0.999 - 1.000
APOL1	ENSG00000100342	10.1	0.96	>0.001	0.883 - 1.037
LAMB3	ENSG00000196878	10.1	0.99	>0.001	0.951 - 1.019
***Selected markers for GEP group B of PDAC***							
MME	ENSG00000196549	65.9	1.00	0.005	1.000 - 1.000
PSG5	ENSG00000204941	62.6	1.00	0.005	1.000 - 1.000
AK5	ENSG00000154027	48.4	1.00	0.005	1.000 - 1.000
SERPINE2	ENSG00000135919	39.1	1.00	0.005	1.000 - 1.000
KCNK2	ENSG00000082482	33.4	1.00	0.005	1.000 - 1.000
MFAP5	ENSG00000197614	33.0	1.00	0.005	1.000 - 1.000
TNFRSF11B	ENSG00000164761	31.8	1.00	0.005	1.000 - 1.000
PSG3	ENSG00000221826	28.5	1.00	0.005	1.000 - 1.000
IL13RA2	ENSG00000123496	21.9	1.00	0.005	1.000 - 1.000
CLDN11	ENSG00000013297	21.0	1.00	0.005	1.000 - 1.000
PAPPA	ENSG00000182752	18.1	1.00	0.005	1.000 - 1.000
FST	ENSG00000134363	15.5	1.00	0.005	1.000 - 1.000
POPDC3	ENSG00000132429	14.8	1.00	0.005	1.000 - 1.000
CDH13	ENSG00000140945	14.1	1.00	0.005	1.000 - 1.000
FGF5	ENSG00000138675	13.9	1.00	0.005	1.000 - 1.000
CCBE1	ENSG00000183287	13.6	1.00	0.005	1.000 - 1.000
XG	ENSG00000124343	13.4	1.00	0.005	1.000 - 1.000
FAM180A	ENSG00000189320	13.2	1.00	0.005	1.000 - 1.000
NRN1	ENSG00000124785	13.2	1.00	0.005	1.000 - 1.000
RXFP1	ENSG00000171509	13.2	1.00	0.005	1.000 - 1.000
ACTC1	ENSG00000159251	12.5	0.97	0.009	0.902 - 1.031
ALPK2	ENSG00000198796	12.0	1.00	0.005	1.000 - 1.000
RECK	ENSG00000122707	11.9	1.00	0.005	1.000 - 1.000
TBX15	ENSG00000092607	11.8	1.00	0.005	1.000 - 1.000
ECM1	ENSG00000143369	11.6	0.97	0.009	0.902 - 1.031
SNAI2	ENSG00000019549	11.2	1.00	0.005	1.000 - 1.000
SEMA3D	ENSG00000153993	11.2	1.00	0.005	1.000 - 1.000
AC0997591	ENSG00000105889	11.0	1.00	0.005	1.000 - 1.000
TBX18	ENSG00000112837	11.0	1.00	0.005	1.000 - 1.000
HMOX1	ENSG00000100292	11.0	1.00	0.005	1.000 - 1.000
VGLL3	ENSG00000206538	10.8	1.00	0.005	1.000 - 1.000
FADS1	ENSG00000149485	10.4	1.00	0.005	1.000 - 1.000
BNC1	ENSG00000169594	10.3	1.00	0.005	1.000 - 1.000

### Validation of tumor-associated markers with high discriminating power between the GEP-A *vs*. GEP-B subgroups of PDAC

**Figure 3:**
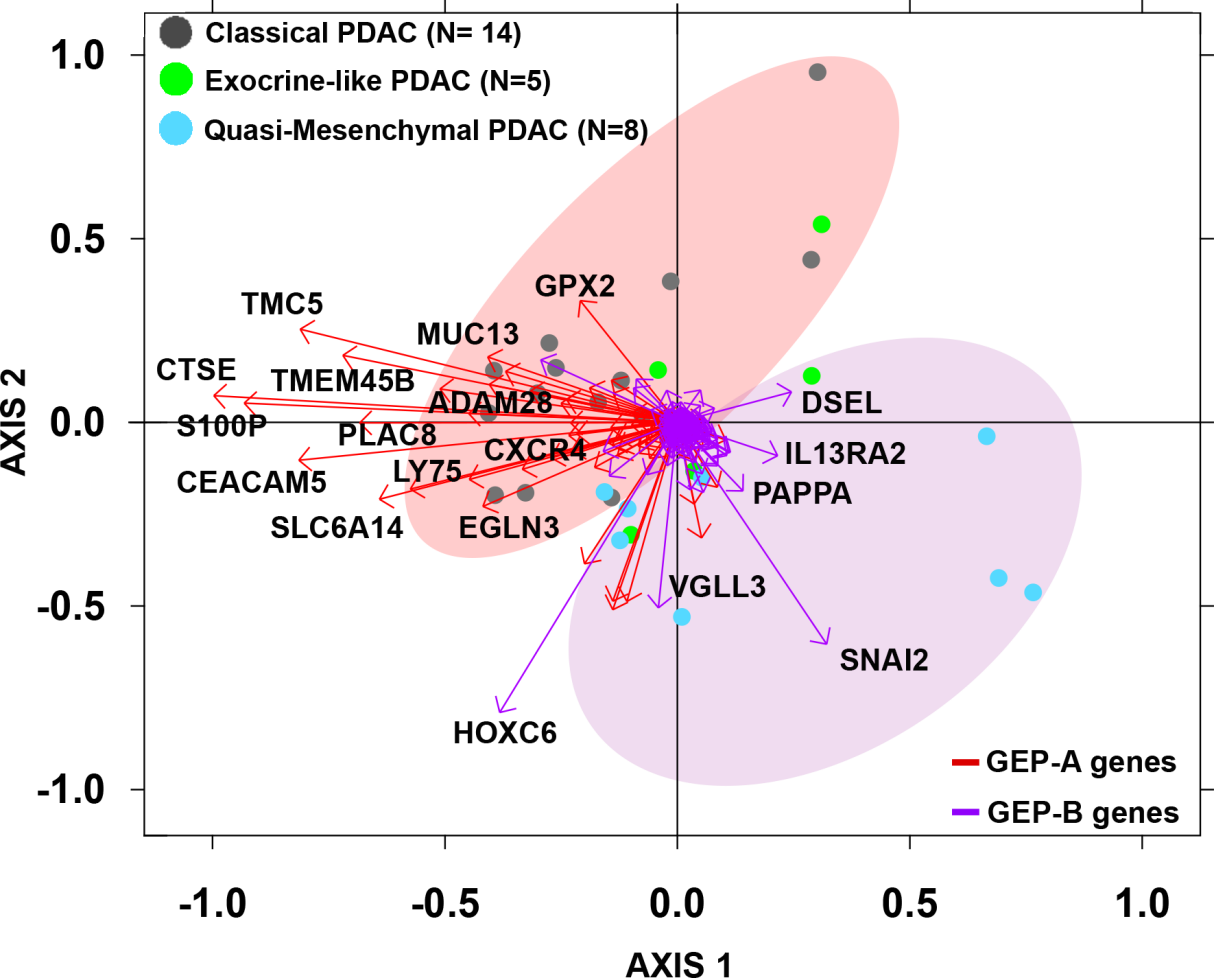
Biplot analysis of 27 PDAC tumors from an independent external validation dataset [25] evaluated for the expression of GEP-A and GEP-B overexpressed tumor markers identified in our series PDAC samples previously classified by Collisson *et al.* as “classical PDAC” tumors (grey dots) were mostly represented by the expression of GEP-A associated genes (red vectors) while “quasi-mesenchymal PDAC” tumors (light blue dots) were grouped by the expression of GEP-B associated markers (purple vectors).

The discriminating value of those genes differentially expressed (overexpressed) in GEP-A vs. GEP-B PDAC tumors (Supplementary Table 7) was further validated using GEP data from an independent series of PDAC available at the public GEO database (*n* = 27; Figure 3). In line with the findings described above for our cases, 14/14 (100%) PDAC samples previously classified by Collisson *et al.* [25] as showing a “classical PDAC” GEP were shown to have GEP-A-associated markers; in contrast, 7/8 (89%) “quasi-mesenchymal PDAC” tumors as defined by Collisson *et al.* [25] had a typical GEP-B phenotype. In this series, the most discriminating GEP-A and GEP-B genes (higher variation between samples with an SD = 1) were: 1) ADAM28, CEACAM5, CTSE, CXCR4, EGLN3, LY75, PLAC8, SLC6A14, S100P, TMC5 and TMEM45B, and 2) HOXC6, PAPPA, SNAI2 and VGLL3, respectively (Figure 3).

### miRNAs genes which may inhibit gene expression in PDAC

In order to determine the impact of the miRNAs signature on the GEP of PDAC tumors, both the miRNA and mRNA gene expression data sets were combined to investigate potential correlations between miRNAs and mRNA genes which are altered in PDAC. Evaluation of each pair of potential miRNA-mRNA interacting genes identified potential interactions for 51 inversely correlated and 139 positively correlated (absolute value of R ≥0.7; *p* < .0001) pairs of miRNA-mRNA genes. Based on currently available miRNA target prediction and database tools, such interactions corresponded to 27 predictable and 1 experimentally validated (miR-30a-star/SLC7A6) interactions for the negatively correlated miRNA-mRNA pairs (Table 4). Of note, both the experimentally validated pair of mRNA/miRNA genes and other 4 predicted miRNA-mRNA interactions (miR-130b-star/TSHZ3, miR-148a/BBS7, miR-148a/LIMA1 and miR-30a/PLAUR) were systematically altered in the 27 PDAC samples analyzed; in turn, another 10 predicted miRNA-mRNA pairs were specifically altered in GEP-A cases (miR-148a stem loop transcript/ACSL5, CTSE, SLC44A4, TNFRSF21 or TSPAN15, and miR-23a/COQ10A) or in GEP-B tumors (miR-1180/BMPER, miR-1244/C10orf118, miR-362-5p/FGD1 and the miR-423 stem loop transcript/RSPO3).

**Table 4:** miRNA-mRNA interactions in PDAC samples (n=27) identified by Spearman correlation analysis of the expression signal identified for those transcripts differentially expressed in pancreatic tumoral vs non-tumoral tissues as detected by the Affymetrix HuGene 1.0 ST and microRNA 2.0 expression arrays

miRNA	Gene Name	Gene ID	ρ	Classification of Interaction	Source of validation/prediction
hsa-miR-30a-star	SLC7A6	ENSG00000103064	−0.74	Validated	IPA; miRSystem; Tarbase5
hsa-miR-1180	BMPER	ENSG00000164619	−0.73	Predicted	PITA, RNAhybrid
hsa-miR-1180	RSPO3	ENSG00000146374	−0.72	Predicted	PITA, RNAhybrid
hsa-miR-1244	C10orf118	ENSG00000165813	−0.71	Predicted	Mirwalk; miRanda; PICTAR5
hsa-miR-130b	TSHZ3	ENSG00000121297	−0.72	Predicted	miRanda; PICTAR5
hsa-miR-130b	CSPG4	ENSG00000173546	−0.75	Predicted	PITA, RNAhybrid
hsa-miR-145	JMJD5	ENSG00000155666	−0.76	Predicted	DIANAmT; miRSystem; Mirwalk; PICTAR5; PITA; Targetscan
hsa-miR-145	KIT	ENSG00000157404	−0.71	Predicted	miRanda; PICTAR5; Targetscan
hp_hsa-miR-148a	TSPAN15	ENSG00000099282	−0.76	Predicted	PITA, RNAhybrid
hp_hsa-miR-148a	ACSL5	ENSG00000197142	−0.79	Predicted	IPA: Moderately
hp_hsa-miR-148a	CTSE	ENSG00000196188	−0.72	Predicted	MiRanda; RNAhybrid
hp_hsa-miR-148a	SLC44A4	ENSG00000204385	−0.79	Predicted	Mirwalk
hp_hsa-miR-148a	TNFRSF21	ENSG00000146072	−0.71	Predicted	Mirwalk
hsa-miR-148a	BBS7	ENSG00000138686	−0.73	Predicted	IPA: Moderately; DIANAmT; miRanda; miRDB; Mirwalk; PICTAR5; Targetscan
hsa-miR-148a	LIMA1	ENSG00000050405	−0.73	Predicted	MiRanda; mirwalk; PITA; RNAhybrid
hsa-miR-148a	SNX24	ENSG00000064652	−0.71	Predicted	Mirwalk; miRanda; PICTAR5
hsa-miR-181c	OSR2	ENSG00000164920	−0.73	Predicted	PITA, RNAhybrid
hsa-miR-193b	SLC25A45	ENSG00000162241	−0.73	Predicted	IPA: Moderately; DIANAmT; Mirwalk; miRanda; PICTAR5; Targetscan
hsa-miR-216a	PPP1R15A	ENSG00000087074	−0.70	Predicted	Mirwalk
hsa-miR-23a	COQ10A	ENSG00000135469	−0.76	Predicted	PITA, RNAhybrid
hsa-miR-23a	KIT	ENSG00000157404	−0.81	Predicted	Mirwalk; PICTAR5
hsa-miR-23a	ZNF828	ENSG00000198824	−0.73	Predicted	Mirwalk; PICTAR5
hsa-miR-29c	PIM1	ENSG00000137193	−0.70	Predicted	Mirwalk
hsa-miR-29c	FST	ENSG00000134363	−0.72	Predicted	RNAhybrid
hsa-miR-30a	PLAUR	ENSG00000011422	−0.70	Predicted	RNAhybrid
hsa-miR-362-5p	FGD1	ENSG00000102302	−0.81	Predicted	Mirwalk
hp_hsa-miR-423	RSPO3	ENSG00000146374	−0.76	Predicted	PITA, RNAhybrid
hsa-miR-939	WDR63	ENSG00000162643	−0.73	Predicted	Mirwalk; DIANAmT; miRanda; PICTAR5; Targetscan

*p-values*<0.0001; IPA: Ingenuity Pathways Software; Moderately: moderately significant level of prediction by IPA software database.

## DISCUSSION

PDAC is currently recognized as a genetically heterogeneous group of tumors, but limited information exists about the biological significance of such variability. In order to gain insight into the genetic heterogeneity of PDAC, here we analyzed for the first time, the global coding and non-coding GEP of a relatively large cohort of PDAC tumors vs. non-tumoral pancreatic tissues. Overall, our results showed two clearly defined subtypes of PDAC which shared a GEP clearly distinct from that of non-tumoral pancreatic tissues. Globally, this included increased expression of genes linked to PDAC cell proliferation, local invasion and metastatic capacity. Thus, the most top-ranked altered networks (e.g.: axonal guidance, inhibition of matrix metalloproteinases, semaphorin, epithelial adherent junction and Rho family of GTPases signaling pathways) are directly involved in cell-cell and cell-matrix adhesion, extracellular matrix degradation and tissue remodeling, angiogenesis, tumor cell migration and invasiveness [28-31]. In addition, cytoskeleton remodeling which is essential for cell movement and growth, is also altered in PDAC tumor cells as reflected by the alteration of axonal guidance, actin cytoskeleton, virus-entry via endocytosis, clathrin-mediated endocytosis, macropinocytosis signaling, as well as signaling pathways activated by the Rho family of GTPases [28-30, 32]; of note, many of such processes had been previously described to be altered in PDAC [27, 29, 30, 33]. PDAC tumors also showed a significant alteration of the early steps of cellular immune responses; this is possibly due to a host response against the tumor [34], as reflected by the alteration of cell adhesion, diapedesis and extravasation, IL8 signaling and antigen presentation via macropinocytosis signaling [35]. However, since the tumors here analyzed represented relatively advanced stages of the disease, alteration of such pathways could also be due to inflammation-mediated cell migration mechanisms [36]. Altogether, these processes found to be altered in PDAC encompass a pro-tumoral scenario; in such scenario PDAC tumor cells secrete factors that actively enhance recruitment of immune cells, while activated immune cells, produce cytokines and growth factors that may exert a direct effect on the tumor cells and the stroma [37]. This hypothesis was fully supported by the observation of areas containing significant leucocyte infiltrates in the tumoral vs. non-tumoral pancreatic tissues, through immunostainings for CD45 and CD15 of formalin-fixed, paraffin-embedded tissues from the same cases (data not shown).

Interestingly, in addition to the common GEP, the two subgroups of PDAC here identified also showed clearly different GEPs. Thus, enrichment in genes involved in the innate and adaptative immune response was predominantly detected in GEP-A vs. GEP-B cases, even when both subgroups of tumors presented similar levels of infiltration by inflammatory cells (data not shown). These findings, together with the increased expression of genes correlated to immune and chronic pancreatic diseases, cellular stress and injury conditions, among GEP-A vs. GEP-B cases, point out the potential involvement of immune selection mechanisms (e.g.: selection of non-immunogenic tumor-cell variants) in the former subgroup of PDAC [30]. Additionally, GEP-A tumors also showed an altered expression of genes involved in cell proliferation, angiogenesis, cell motility, invasion and tumor progression (e.g. genes involved in the MSP-RON, actin nucleation by the AR-WASP complex and by the Rho, Rac, PAK, Cdc42, integrin, ERK/MAPK, Paxilin, FAK, NF-KB, calpain protease and glioma tumor invasiveness pathways [28, 38-48], among other genes [31, 49, 50]), would confer a highly-aggressive phenotype to GEP-A tumor cells. Of note, GEP-A tumors retained an epithelial GEP phenotype which includes an increased expression of epithelial markers, carcinoembrionary antigens (CEACAM1, CEACAM6 and CEACAM 5) and cytokeratins (KRT7 and KRT19).

In contrast to GEP-A tumors, GEP-B PDAC cases showed fewer specifically altered canonical pathways, despite an overall similar number of altered genes was found in both subgroups of tumors (1,183 vs. 1,012 altered genes in GEP-A vs. GEP-B cases, respectively). Of note, GEP-B cases showed no specific GEPs associated to tumor cell proliferation; moreover, they had decreased expression of genes linked to canonical pathways associated with immune responses. Thus, GEP-B tumors had: i) enhanced self-defense mechanisms against complement-dependent cytotoxicity, as reflected by overexpression of the KIT mast cell-associated molecule [51]; ii) defective expression of major histocompatibility complex (MHC) molecules which are that frequently involved in tumor immune escape [52], and/or; iii) greater cancer-driven immunosuppression as a consequence of increased expression of the programmed cell death 1 ligand 2 (PDCD1LG2) [53] and the VGFC [51] genes. Most interestingly, our results indicate activation of epithelial-mesenchymal transition (EMT) genes in GEP-B tumors as depicted by their higher expression of mesenchymal signature genes (e.g: CDH2, SNAI1, SNAI2 and VIM) and other EMT-related genes (e.g. S100A4), together with decreased expression of epithelial markers (e.g: CEACAM6, EPCAM, CDH1, KRT8 and KRT18) [54-56], which activate the integrin linked kinase (ILK) signaling pathway [57], inhibit genes involved in cell-cell junction signaling pathways and expression of adhesion molecules (e.g: DSG2, DSC2 and PKP2 genes) [58]. Altogether, these results suggest that in GEP-B tumors, immunosuppression linked to an EMT phenotype could be involved in the pathogenesis of PDAC. Whether immunosuppression precedes or develops after acquisition of an EMT phenotype, remains to be determined.

Overall, the above results confirm and extend on previous observations about the existence of distinct molecular subgroups of PDAC tumors as identified by GEP, including a “classical epithelial” and a “quiasi-mesenchymal” subtype of PDAC [25]. However, despite this, we failed to detect a third subtype of PDAC tumors with an exocrine-like phenotype, as previously described by Collisson *et al.* in a larger patient cohort [25]. Such apparently discrepant results could potentially be due to differences in the size of the cohort analyzed (27 tumoral samples in our study vs. 63 PDAC samples in the series of Collisson *et al.*), the methodology used (e.g. macrodissected freshly-frozen PDAC tissues vs. a mixture of formalin-fixed paraffin-embedded and freshly-frozen PDAC tissues, with or without microdissection), and/or the comparison against non-pancreatic reference tissues done in our series, but not in the study by Collisson *et al.* [25]. Of note, we also failed to confirm the previously reported association between specific GEP and the clinical and histopathological features of the disease (e.g.: the association between a mesenchymal phenotypes and both adverse tumor features and a poorer prognosis) [25, 56, 59]. Independently of the pathogenic significance of the distinct GEP and tumor phenotypes here described, the understanding of such biological pathways may contribute to better identify more efficient treatment strategies and to e.g. avoid standard PDAC therapy with gemcitabine and 5-fluorouracil in patients with GEP-B, due to the high chemoresistance of PDAC cells with an EMT phenotype to these treatments [56, 60].

Despite all the above, a major concern remains regarding the functional effect of microRNA expression levels on the mRNA transcript expression. Here we identified several miRNAs to be significantly correlated with expression of specific genes at the mRNA level. Among other miRNA-mRNA pairs, the miR-30a-star emerged in our series, as significantly correlated with an increased expression of the SLC7A6 gene transcript. The SLC7A6 (solute carrier 7 member of this family of genes) has known functions in the transport of leucin, being involved in promoting cell growth in many cancers [61, 62] and podocyte development [63]. Furthermore, expression of the miR-30 family of miRNAs is a key element during embryonic pancreatic development to maintain the epithelial phenotype of pancreatic tissues [64], their inhibition mediating an EMT phenotype in several types of cancer [65, 66]. Although, we were not able to detect any other (validated) inverse correlation for other miR-30 elements-genes, decreased expression of miR-30a, miR-30c and miR-30d was found in both GEP-A and GEP-B tumors with an epithelial vs. EMT phenotype, respectively; these results suggest that the EMT phenotype is potentially promoted in all PDAC tumors, but only those tumors carrying additional molecular/genomic alterations associated with immunosuppression and/or activation of ILK signaling could more clearly acquire a mesenchymal phenotype. Other miRNAs found to be altered in PDAC were exclusively deregulated among GEP-A or GEP-B tumors. Interestingly deregulated miRNA genes in GEP-A tumors included the stem loop transcript of miR-148a. The miR-148a miRNA possibly mediates overexpression of genes involved in tumor cell growth (e.g. acetyl-CoA sintetase, ACSL5), migration (e.g. the TSPAN15 tetraspanin) with an effect also on both apoptosis and immune responses (e.g. the TNFRSF21 tumor necrosis factor receptor); in turn miR-23a inhibits the antioxidative effect of the coenzyme Q10 homologe A (COQ10A) gene. In contrast, those miRNA genes which were overexpressed in GEP-B tumors included the miR-1180, miR-362-5p and the miR-423, all of which promote tumor cell proliferation and invasion through e.g. the BMP binding endothelial regulator (BMPER), the FYVE Rho GEF and PH domain containing 1 (FGD1) and the R-spondin 3 (RSPO3) genes.

Interestingly, clear cut discrimination between GEP-A and GEP-B tumors carrying an epithelial vs. mesenchymal-like molecular profile could be obtained via a set of 63 and 97 mRNA genes overexpressed in GEP-A and GEP-B tumors, respectively, as also confirmed in an external series of 27 PDAC patients [25]. These results indicate that these gene signatures could potentially serve in the future as prior knowledge for the discovery of biomarker candidates (i.e: CEACAM5, GPX2, MUC13, S100P and TMEM45B for GEP-A cases, and PAPPA and VGLL3 for GEP-B tumors) that may contribute to more efficient treatment and/or monitoring of both subtypes of PDAC tumors. In addition, in our series a small panel of 5 overexpressed PDAC markers (S100A11, GPR137B, SULF1, POSTN and miR-155) would allow precise distinction between PDAC and non-tumoral pancreatic tissues. In line with this hypothesis, strong expression of the S100A11 and GPR137B genes has been reported at the protein level in PDAC tissues, while SULF1 and POSTN are expressed at more variable patterns [67-69]; of note all four proteins have been also found to be secreted and present in both tumor tissues and the plasma [67, 68, 70] from PDAC patients. Altogether, secretion of these proteins outside the tumor cell, supports the potential utility of these genes as candidate markers for the diagnosis and monitoring of PDAC patients.

In summary, the present study provides evidence for a common GEP of tumor cells in PDAC, at the same time it confirms the genetic complexity and heterogeneity of these tumors with at least two clearly distinct and unique GEPs (e.g. epithelial-like vs. mesenchymal-like genomic profiles), potentially reflecting different pathways involved in the oncogenesis and progression of PDAC. In addition, our results also highlight the potential role of the tumor microenvironment, particularly of the immune system, in PDAC, with potential diagnostic and therapeutic implications.

## MATERIALS AND METHODS

### Patients and samples

Tumor tissue specimens were obtained at diagnostic surgery from 27 consecutive sporadic PDAC patients (18 males and 9 females; mean age of 67 years, ranging from 41 to 79 years); in addition, non-tumoral pancreatic tissue specimens were also collected from another 5 patients each having a different pancreatic disease (pancreatic fibrosis with inflammation, chronic pancreatitis, an ampullary tumor, a neuroendocrine tumor and a PDAC, respectively). All PDAC patients underwent surgical tumor resection at the Division of Hepatobiliary and Pancreatic Surgery of the University Hospital of Salamanca (Salamanca, Spain). PDAC tumors were diagnosed and classified according to Adsay *et al*. [71] with the following distribution: 8 cases corresponded to well-differentiated/grade I tumors; 11 to moderately-differentiated/grade II, and; 8 to poorly-differentiated/grade III PDAC. Histopathological grade was confirmed in all cases in a second independent evaluation by an experienced pathologist. Most tumors (21/27, 78%) were localized in the head of the pancreas, while the remaining six cases were localized in the pancreatic body (1/27, 4%), the tail (3/27, 11%) and the pancreatic body/tail (2/27, 7%). Mean tumor size at diagnostic surgery was of 3.0±0.82 cm, 6 cases corresponding to TNM stage IIA tumors and 21 to TNM stage IIB. The most relevant clinical and laboratory patient characteristics are summarized in Supplementary Table 1.

Pancreatic tissue samples were collected immediately after surgical resection, snap frozen and stored in OCT at −80°C (Tumor Biobank of the University Hospital of Salamanca, Red de Bancos de Tumores de Castilla y León, Salamanca, Spain). The study was approved by the local ethics committee of the University Hospital of Salamanca (Salamanca, Spain) and informed consent was given by each individual prior to entering the study, according to the Declaration of Helsinki. Once the histopathological diagnosis had been established, sections from the paraffin-embedded tissue samples were cut from three different areas representative of the tumoral tissue with > 70% tumor cell infiltration by hematoxylin-eosin staining, excluding stroma-enriched tumor areas. Selection of the neighbour areas of the tumor containing ≥70% tumor cells was performed on dissected samples stored in OCT.

### RNA extraction and gene expression profiling (GEP) microarray studies

For GEP, sample preparation was performed as described in the Affymetrix GeneChip Expression Analysis Manual (Santa Clara, CA, USA). Briefly, each frozen tissue (≥0.3 g) was crushed to powder at cryogenic temperatures and homogeneized in Trizol (Life Technologies, Inc., Rockville, MD). Total RNA was then extracted using the miRNeasy mini kit according to the manufacturer's protocol (Qiagen, Valencia, CA); subsequently, the quality and integrity of the RNA was evaluated in an Agilent 2100 Bioanalyzer (Agilent Technologies Inc., Santa Clara, CA, USA). Total RNA (100-1,000ng) from both tumoral and non-tumoral pancreatic tissues was hybridized to both the Affymetrix Human Gene ST 1.0 Expression and the microRNA 2.0 Expression arrays, according to the instructions of the manufacturer. Fluorescence signals were detected using the GeneChip Scanner 3000 7G (Affymetrix) and data stored as .CEL files.

For data analysis, GEP raw data derived from the Affymetrix Human Gene Expression ST 1.0 microarray and the microRNA 2.0 microarray, was normalized with the Robust Multi-array Average (RMA) algorithm; this included sequentially background correction, intra- and inter-microarray well normalization, probe set summarization and calculation of expression signals, respectively [72]. Unsupervised classification of samples and genes −28,869 mRNA and 4,544 human small non-coding RNA transcripts- was performed by principal component (PCA) and hierarchical clustering analyses (HCA) using the expression signal detected for each gene for each probe set, and the MultiExperiment Viewer (MeV, version 4.8.1) [73] and Cluster 3.0 software programs (PAM software; http://www-stat.stanford.edu/~tibs/PAM). Clustering was run using an Euclidean correlation metric and the average linkage method. For visualization of dendograms, the TreeView software (version 1.0.4) [74] was used. Differentially expressed genes between all tumor samples or GEP-defined subgroups of PDAC samples vs. non-tumoral samples were identified by supervised two-class unpaired Significance Analysis of Microarray (SAM; MeV software) [75] based on a false discovery rate (FDR) cut off of < .0001 and an absolute fold change cutoff of ≥2.0.

In order to identify the best combination of genes for the discrimination between the GEP of PDAC tumors and non-neoplastic pancreatic tissues, a two-step strategy was used. In the first step, five prediction algorithms were used: 1) PAM (PAM software v 2.1; University of Stanford, CA) [76], 2) Partial Least Squares algorithms (PLS; SIMFIT software v.6.9.9; www.simfit.org.uk), 3) Support Vector Machines (SVM), 4) K-Nearest Neigbour (KNN) and, 5) Random Forest algorithms; the latter three algorithms are implemented in the Babelomics suite (http://babelomics.bioinfo.cipf.es/) [77]. For this purpose, GEP data from two-thirds of the tumoral samples was randomly selected as a training dataset, while the remaining were used to build the validation dataset. In this first step, informative genes were defined as those represented in ≥ 4/5 analyses. In the second step, the discriminative power of each informative gene was assessed by receiver operating curve (ROC) analysis (SPSS 15.0 Inc, Chicago, IL, USA). Finally, those genes which depicted a high predictive power-area under the curve (AUC) ≥0.96- together with an expression fold change (vs. non-tumoral tissues) > 4, were selected. Validation of genes was performed in the same pancreatic sample series (27 tumoral plus 5 pancreatic non-tumoral samples) applying the PAM and SVM models, using a 10-fold and a leave-one-out-cross validation method, respectively.

For the identification of miRNA candidates acting as gene-regulators in PDAC samples, Spearman correlation analyses were performed to identify significant correlations between individual miRNA and mRNA gene transcripts across tumoral (*n* = 27) and non-tumoral (*n* = 5) samples. Each miRNA-mRNA interaction identified was subsequently evaluated with the Ingenuity Pathway Analysis software (IPA, Ingenuity Systems, www.ingenuity.com), as well as with available databases of experimentally validated miRNA interactions (TarBase 6.0 and miRWalk-database) and miRNA target prediction tools (DIANA-microT-CDS v5.0, miRWalk-database and miRecords) [78, 79]. Functional enrichment analysis of deregulated genes, analysis of canonical pathways, correlation networks, as well as gene-gene and gene-miRNA interactions were defined using the IPA software.

### Validation of gene expression profiles by quantitative real-time PCR assays

TaqMan Gene Expression Assays were used to validate GEP in the same samples used for microarray studies via the Step One Plus Real-Time PCR System-Applied Biosystems (ABI; Foster City, CA, USA) according to the manufacturer's instructions. The assays ID for the genes studied were as follows: Hs_00429010_m1 (*PDIA2*), Hs_00170815_m1 (*POSTN*), Hs_00418420_m1 (*SCYN*), 002220 (hsa-miR-216a), 002337 (hsa-miR-217), 002623 (hsa-miR-155) and 000507 (hsa-miR-203). Each PCR was carried out in duplicate in a final volume of 10 uL using the TaqMan Fast Universal Mastermix (ABI) and the following cycling parameters: incubation at 95ºC (20 s), followed by 50 cycles at 95ºC (1s) and an incubation at 60ºC (20s). GEP and miRNA expression data was normalized against the GAPDH internal housekeeping gene and the RNU43 internal control, and it was further analyzed using the StepOne software (v2.0; ABI). The relative amounts of the quantified genes were calculated using the following equation: 2^−ΔCT^ (ΔC_T_ = C_T_GENE-C_T_GAPDH or RNU43) expressed as arbitrary units (AU); results showed a high degree of correlation between data from both microarrays and RQ-PCR methods, for all genes evaluated (r^2^≥ 0.66, *p* < .0001; Supplementary Figure 1).

### External validation series of PDAC tumors

External validation of the predictive value of the differentially expressed genes that discriminated between the distinct GEP-defined subgroups of PDAC tumors found in our series, was performed in a group of previously reported PDAC patients (*n* = 27). GEP array data files (Affymetrix Human Genome U133 Plus 2.0 Array) are publicly available at the GEO database (accession number GSE17891) [25]. Downloaded data CEL files were normalized using the RMA algorithm and overlapping probe sets were defined on the basis of probe specificity, using the GATExplorer server [80]. Probe sets with the best specificity to the interrogated genes (see Supplementary Table 7) were selected, and the expression signals detected for each gene for each probe set were further analyzed using the column metric preserving biplot assay [81] implemented in the SIMFIT statistical software (http://www.simfit.org.uk/).

### Other statistical methods

The Mann-Whitney U test and a linear regression model were used to evaluate the statistical significance of differences observed between groups and to explore the degree of correlation between different variables, respectively (SPSS 15.0 Inc.). P-values ≤.05 were considered to be associated with statistical significance.

## SUPPLEMENTARY FIGURE AND TABLES
















