# Supplementary material for: Identification and characterization of the gene expression profiles for protein coding and non-coding RNAs of pancreatic ductal adenocarcinomas
Source: Oncotarget. 2015 May 22;6(22):19070–86. doi: 10.18632/oncotarget.4233 (PMC4662476; doi:10.18632/oncotarget.4233)
Supplement: Supplementary file 1 [file oncotarget-06-19070-s001.pdf]

Identification and characterization of the gene expression profiles for protein coding and non-coding RNAs of pancreatic ductal adenocarcinomas

Supplementary Material

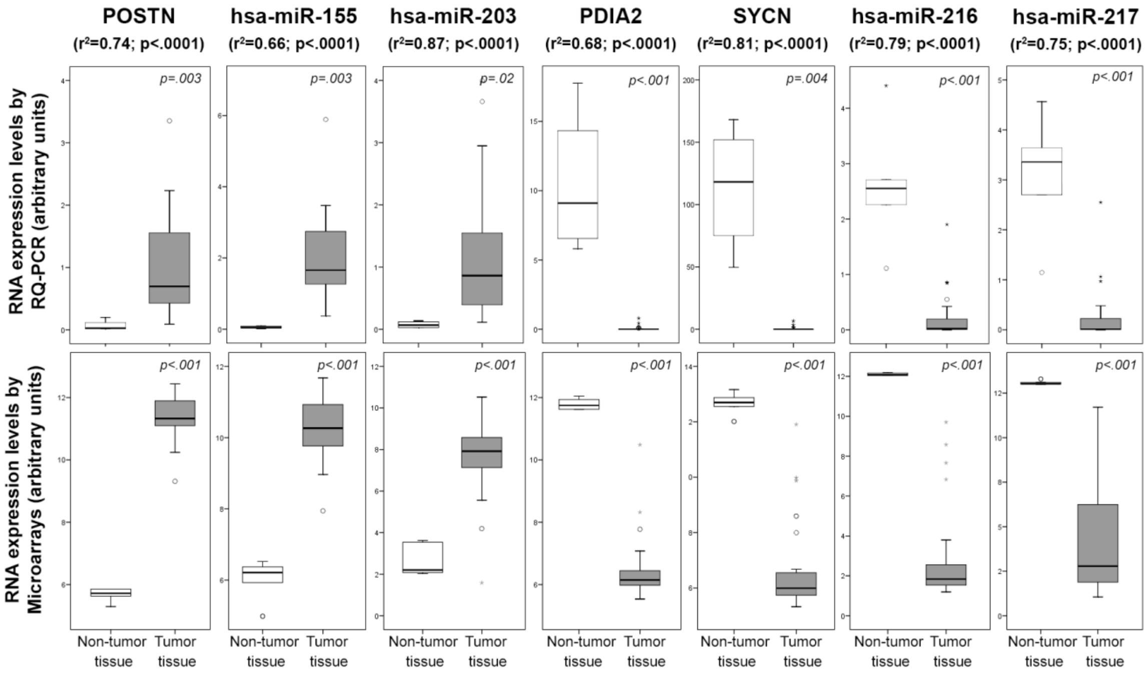

Supplementary Figure 1: Expression levels of the PDIA2, SYCN and POSTN mRNA coding genes and the hsa-mir-217, hsa-mir-216, hsa-mir-155 and hsa-mir-203 miRNA transcripts in PDAC tumor tissues (n=27) vs. non-tumoral pancreatic tissues (n=5) as assessed by the RQ-PCR method used to validate GEP microarray data. Notched boxes represent 25th and 75th percentile values. The line in the middle and vertical lines correspond to the median values and 95% confidence intervals, respectively. Outliers (values that are between 1.5 and 3 times the interquartile range) are marked with a circle and extreme cases (values that are more than three times the interquartile range) with an asterisk. Values of the correlation analysis performed between the RQ-PCR and the microarrays expression levels of the PDAC-deregulated are showed in parenthesis.

Supplementary Table 1: Clinical and biological characteristics of those PDAC patients included in this study (n= 27).

Supplementary Table 2: Gene transcripts differentially expressed in PDAC tumor tissues (n=27), and both the GEP-A (n=24) and GEP-B (n=3) subgroups of PDACs vs. non- tumoral pancreatic tissues (n=5) analyzed with the Affymetrix Human Gene 1.0 ST Expression array.

Supplementary Table 3: Gene transcripts differentially expressed in PDAC tumor tissues (n=27), and both the GEP-A (n=24) and GEP-B (n=3) subgroups of PDACs vs. non- tumoral pancreatic tissues (n=5) analyzed with the Affymetrix miRNA 2.0 Expression array.

Supplementary Table 4: Most representative canonical pathways involved in PDAC tumors as identified through analysis of the GEP of coding and non-coding RNAs (n=27) which were shared by the GEP-A and GEP-B subgroups of PDAC tumors.

Supplementary Table 5: Most representative canonical pathways involved in the GEP-A subgroup of PDAC tumors (n=24) as identified through analysis of the GEP of coding and non-coding RNAs.

Supplementary Table 6: Most representative canonical pathways involved in the GEP-B subgroup of PDAC tumors (n=3) as identified through analysis of the GEP of coding and non-coding RNAs.

Supplementary Table 7: Receiver operating characteristic (ROC) curve analysis specifically performed for genes previously selected based on the predictive algorithms which contributed most to the discrimination of the GEP subgroups A (24 tumors vs. 3 tumors and 5 non-tumoral tissues) and B (3 tumors vs. 24 tumors and 5 non-tumoral tissues) of PDAC tumor tissues.
