# Supplementary material for: Identification and characterization of the gene expression profiles for protein coding and non-coding RNAs of pancreatic ductal adenocarcinomas
Source: Oncotarget. 2015 May 22;6(22):19070–86. doi: 10.18632/oncotarget.4233 (PMC4662476; doi:10.18632/oncotarget.4233)
Supplement: Supplementary file 2 [file oncotarget-06-19070-s002.pdf]

SUPPLEMENTARY TABLE 1. Clinical and biological characteristics of those PDAC patients included in this study (n= 27).

Patient ID	Gender	Age (years)	CA19.9 serum levels (U/ml)	Site of primary tumor	Tumor size (cm)	Histological grade		TNM stage		Type of surgical resection	% of cytogenetcally altered cells by iFISH in the tissue analyzed	Patient outcome status	OS (months)	GEP group *
1	M	74	144	Head	2.5	Well	I	T2N1M0	IIB	R1	85	Dead	93	GEP-A
2	M	67	<2.5	Head	2	Well	I	T3N1M0	IIB	R1	64	Dead	11	GEP-A
3	M	59	319	Head	2	Well	I	T2N1M0	IIB	R0	70	Dead	5	GEP-A
4	M	62	177	Head	3.5	Well	I	T3N1M0	IIB	R1	90	Dead	22	GEP-A
5	M	79	232	Head	4	Well	I	T3N1M0	IIB	R0	69	Dead	23	GEP-A
6	F	55	4.32	Tail	3	Well	I	T3N1M0	IIB	R0	90	Alive	11	GEP-A
7	M	78	150	Head	2.5	Well	I	T3N1M0	IIB	R0	62	Alive	71	GEP-A
8	F	66	290	Head	3	Well	I	T3N1M0	IIB	R0	77	Alive	48	GEP-A
9	F	77	192	Head	3	Moderately	II	T3N1M0	IIB	R0	65	Alive	15	GEP-A
10	M	76	>500	Head	3.5	Moderately	II	T3N1M0	IIB	R0	75	Dead	7	GEP-A
11	M	61	3	Head	3	Moderately	II	TxN0M0	IB/IIA	R1	66	Dead	16	GEP-B
12	M	52	>500	Head	2.5	Moderately	II	T3N1M0	IIB	R1	37	Dead	0	GEP-A
13	M	72	19	Head	2.2	Moderately	II	T3N0M0	IIA	R0	96	Dead	5	GEP-A
14	M	74	45.3	Head	3.5	Moderately	II	T2N1M0	IIB	R1	70	Dead	15	GEP-A
15	M	65	<2.5	Head	3	Moderately	II	T3N1M0	IIB	R0	60	Dead	12	GEP-B
16	M	77	NA	Tail	4.5	Moderately	II	T3N0M0	IIA	R0	60	Dead	18	GEP-A
17	F	79	59	Head	3	Moderately	II	T3N1M0	IIB	R1	85	Dead	14	GEP-A
18	F	60	22.20	Body/tail	4	Moderately	II	T3N0M0	IIA	R0	85	Dead	21	GEP-A
19	M	71	52	Head	4	Moderately	II	T3N1M0	IIB	R1	65	Dead	8	GEP-A
20	F	45	>500	Head	3.5	Poor	III	T2N1M0	IIB	R0	56	Dead	19	GEP-B
21	M	78	176	Head	3.5	Poor	III	T2N1M0	IIB	R0	63	Dead	12	GEP-A
22	F	75	124	Body/tail	5.8	Poor	III	T2N1M0	IIB	R0	55	Dead	50	GEP-A
23	F	41	150	Head	2.7	Poor	III	T3N1M0	IIB	R1	63	Dead	19	GEP-A
24	F	57	>500	Body/tail	5	Poor	III	T3N0M0	IIA	R1	70	Dead	18	GEP-A
25	F	69	30.40	Head	3	Poor	III	T3N1M0	IIB	R0	70	Dead	17	GEP-A
26	M	54	NA	Head	3.5	Poor	III	T3N1M0	IIB	R1	60	Alive	22	GEP-A
27	M	74	305	Head	3	Poor	III	T2N0M0	IB	R0	65	Alive	6	GEP-A

M: male; F: female; CA19.9: carbohydrate associated antigen 19.9; R0: microscopically negative resection margins; R1: microscopically positive resection margins; OS: overall survival; *defined by principal component and hierarchical clustering analyses of GEP obtained by both mRNA and non-coding RNA microarray studies. NA: data not available.
