# Supplementary material for: Identification and characterization of the gene expression profiles for protein coding and non-coding RNAs of pancreatic ductal adenocarcinomas
Source: Oncotarget. 2015 May 22;6(22):19070–86. doi: 10.18632/oncotarget.4233 (PMC4662476; doi:10.18632/oncotarget.4233)
Supplement: Supplementary file 3 [file oncotarget-06-19070-s003.pdf]

SUPPLEMENTARY TABLE 2. Gene transcripts differentially expressed in PDAC tumor tissues (n=27), and both the GEP-A (n=24) and GEP-B (n=3) subgroups of PDACs vs non- tumoral pancreatic tissues (n=5) analyzed with the Affymetrix Human Gene 1.0 ST Expression array.

Gene ID	Gene name (by Affymetrix array)	Transcript description	Ratio T vs Non-T	Fold Change T vs Non-T	q-value (%)	Ratio T GEP-A vs Non-T	Fold Change GEP-A vs Non-T	q-value (%)	Ratio T GEP-B vs Non-T	Fold Change GEP-B vs Non-T	q-value (%)
ENSG00000148584	A1CF	protein_coding	0.26	-3.9	0	0.27	-3.6	0	0.11	-8.8	0
ENSG00000144959	AADACL1	protein_coding	2.09	2.1	0	2.08	2.1	0	NS	NS	-
ENSG00000008311	AASS	protein_coding	0.24	-4.1	0	0.25	-3.9	0	NS	NS	-
ENSG00000183044	ABAT	protein_coding	0.22	-4.7	0	0.21	-4.7	0	NS	NS	-
ENSG00000165029	ABCA1	protein_coding	2.51	2.5	0	2.6	2.6	0	NS	NS	-
ENSG00000154265	ABCA5	protein_coding	0.34	-2.9	0	0.36	-2.8	0	NS	NS	-
ENSG00000114770	ABCC5	protein_coding	2.16	2.2	0	2.23	2.2	0	NS	NS	-
ENSG00000033050	ABCF2	protein_coding	NS	NS	-	NS	NS	-	2.79	2.8	0
ENSG00000099204	ABLIM1	protein_coding	NS	NS	-	NS	NS	-	0.21	-4.7	0
ENSG00000173210	ABLIM3	protein_coding	2.38	2.4	0	2.33	2.3	0	2.77	2.8	0
ENSG00000002726	ABP1	protein_coding	2.57	2.6	0	2.73	2.7	0	NS	NS	-
ENSG00000159842	ABR	protein_coding	2.23	2.2	0	2.12	2.1	0	NS	NS	-
ENSG00000240347	AC004862.1	misc_RNA	0.44	-2.3	0	0.45	-2.2	0	NS	NS	-
ENSG00000183444	AC004967.6	pseudogene	0.47	-2.1	0	0.45	-2.2	0	NS	NS	-
ENSG00000233404	AC005035.1	protein_coding	2.12	2.1	0	2.06	2.1	0	NS	NS	-
ENSG00000224287	AC005538.3	pseudogene	3.2	3.2	0	3.09	3.1	0	NS	NS	-
ENSG00000119686	AC007182.1	protein_coding	2.39	2.4	0	2.56	2.6	0	NS	NS	-
ENSG00000180672	AC007362.2	pseudogene	8.88	8.9	0	9.28	9.3	0	NS	NS	-
ENSG00000187534	AC007842.1	pseudogene	2.74	2.7	0	2.66	2.7	0	NS	NS	-
ENSG00000244080	AC008481.1	misc_RNA	0.49	-2	0	0.5	-2	0	NS	NS	-
ENSG00000230259	AC008738.1	pseudogene	0.51	-2	0	0.51	-2	0	0.5	-2	0
ENSG00000242113	AC009311.1	misc_RNA	NS	NS	-	NS	NS	-	5.77	5.8	0
ENSG00000176824	AC009951.1	pseudogene	2.8	2.8	0	3	3	0	NS	NS	-
ENSG00000197813	AC011450.2	lincRNA	0.43	-2.3	0	0.43	-2.3	0	0.37	-2.7	0
ENSG00000167642	AC011479.2	protein_coding	NS	NS	-	NS	NS	-	0.16	-6.3	0
ENSG00000213406	AC013477.3	pseudogene	2.01	2	0	NS	NS	-	3	3	0
ENSG00000159289	AC013486.2	protein_coding	0.25	-3.9	0	0.25	-4	0	NS	NS	-
ENSG00000163364	AC017048.1	lincRNA	NS	NS	-	NS	NS	-	2.93	2.9	0
ENSG00000187812	AC019294.2	pseudogene	0.3	-3.3	0	0.31	-3.2	0	NS	NS	-
ENSG00000242863	AC020704.1	misc_RNA	0.49	-2.1	0	0.49	-2	0	NS	NS	-
ENSG00000230551	AC021078.4	processed_transcript	2.78	2.8	0	2.67	2.7	0	NS	NS	-
ENSG00000206567	AC022007.1	lincRNA	0.48	-2.1	0	0.47	-2.1	0	NS	NS	-
ENSG00000105520	AC024575.2	protein_coding	NS	NS	-	NS	NS	-	3.34	3.3	0
ENSG00000143429	AC027612.7	pseudogene	NS	NS	-	NS	NS	-	8.85	8.8	0
ENSG00000091592	AC055839.1	protein_coding	2.06	2.1	0	2.1	2.1	0	NS	NS	-

ENSG00000244242	AC068580.3	protein_coding	2.35	2.3	0	2.24	2.2	0	3.16	3.2	0
ENSG00000232742	AC073869.12	pseudogene	NS	NS	-	NS	NS	-	4.54	4.5	0
ENSG00000225422	AC078889.3	pseudogene	4.1	4.1	0	3.71	3.7	0	7.19	7.2	0
ENSG00000147642	AC079061.1	protein_coding	0.15	-6.8	0	0.15	-6.7	0	NS	NS	-
ENSG00000235734	AC079337.1	pseudogene	NS	NS	-	0.48	-2.1	0	NS	NS	-
ENSG00000236654	AC079780.1	pseudogene	4.63	4.6	0	3.99	4	0	9.78	9.8	0
ENSG00000239776	AC079949.1	novel miRNA	NS	NS	-	NS	NS	-	0.44	-2.3	0
ENSG00000241588	AC090825.4	misc_RNA	0.48	-2.1	0	0.47	-2.1	0	NS	NS	-
ENSG00000236062	AC091492.2	pseudogene	0.48	-2.1	0	0.47	-2.1	0	NS	NS	-
ENSG00000187185	AC092118.1	protein_coding	0.49	-2	0	0.49	-2.1	0	NS	NS	-
ENSG00000168367	AC092327.1	lincRNA	0.51	-2	0	0.5	-2	0	NS	NS	-
ENSG00000214944	AC093283.3	protein_coding	NS	NS	-	NS	NS	-	0.4	-2.5	0
ENSG00000152117	AC093838.4	pseudogene	1.96	2	0	2	2	0	NS	NS	-
ENSG00000183900	AC099522.1	pseudogene	NS	NS	-	NS	NS	-	3.88	3.9	0
ENSG00000153815	AC099524.2	protein_coding	NS	NS	-	1.99	2	0	NS	NS	-
ENSG00000105889	AC099759.1	protein_coding	NS	NS	-	NS	NS	-	10.96	11	0
ENSG00000213373	AC100793.3	lincRNA	0.21	-4.7	0	0.21	-4.8	0	0.25	-4	0
ENSG00000173517	AC107883.1	protein_coding	2.22	2.2	0	1.95	2	0	4.32	4.3	0
ENSG00000138640	AC108065.1	protein_coding	0.43	-2.3	0	0.45	-2.2	0	0.26	-3.8	0
ENSG00000240545	AC112518.2	misc_RNA	NS	NS	-	NS	NS	-	0.44	-2.3	0
ENSG00000123384	AC137834.1	protein_coding	3.55	3.6	0	3.32	3.3	0	5.38	5.4	0
ENSG00000076555	ACACB	protein_coding	0.41	-2.4	0	0.43	-2.3	0	0.21	-4.7	0
ENSG00000115361	ACADL	protein_coding	0.12	-8.3	0	0.13	-8	0	0.09	-11.7	0
ENSG00000075239	ACAT1	protein_coding	NS	NS	-	0.35	-2.9	0	NS	NS	-
ENSG00000120437	ACAT2	protein_coding	2.48	2.5	0	2.16	2.2	0	5.01	5	0
ENSG00000107897	ACBD5	protein_coding	NS	NS	-	1.96	2	0	NS	NS	-
ENSG00000130234	ACE2	protein_coding	NS	NS	-	NS	NS	-	0.25	-4.1	0
ENSG00000102575	ACP5	protein_coding	2.61	2.6	0	2.8	2.8	0	NS	NS	-
ENSG00000123983	ACSL3	protein_coding	2.21	2.2	0	2.22	2.2	0	NS	NS	-
ENSG00000068366	ACSL4	protein_coding	2.97	3	0	2.94	2.9	0	NS	NS	-
ENSG00000197142	ACSL5	protein_coding	NS	NS	-	6.96	7	0	NS	NS	-
ENSG00000005187	ACSM3	protein_coding	0.34	-3	0	NS	NS	-	0.07	-13.7	0
ENSG00000154930	ACSS1	protein_coding	0.45	-2.2	0	0.46	-2.2	0	0.3	-3.3	0
ENSG00000107796	ACTA2	protein_coding	5.98	6	0	5.89	5.9	0	NS	NS	-
ENSG00000159251	ACTC1	protein_coding	NS	NS	-	NS	NS	-	12.48	12.5	0
ENSG00000072110	ACTN1	protein_coding	3.1	3.1	0	2.93	2.9	0	4.46	4.5	0
ENSG00000138107	ACTR1A	protein_coding	NS	NS	-	NS	NS	-	2.86	2.9	0
ENSG00000138071	ACTR2	protein_coding	2.23	2.2	0	2.28	2.3	0	NS	NS	-
ENSG00000115091	ACTR3	protein_coding	2.2	2.2	0	2.21	2.2	0	2.17	2.2	0
ENSG00000106526	ACTR3C	protein_coding	NS	NS	-	NS	NS	-	0.17	-5.8	0
ENSG00000115170	ACVR1	protein_coding	2.62	2.6	0	2.52	2.5	0	3.46	3.5	0
ENSG00000196839	ADA	protein_coding	NS	NS	-	NS	NS	-	4.44	4.4	0

ENSG00000137845	ADAM10	protein_coding	2.17	2.2	0	2.16	2.2	0	NS	NS	-
ENSG00000148848	ADAM12	protein_coding	8.44	8.4	0	5.91	5.9	0	28.65	28.6	0
ENSG00000151694	ADAM17	protein_coding	2.19	2.2	0	2.16	2.2	0	NS	NS	-
ENSG00000135074	ADAM19	protein_coding	4.21	4.2	0	4.43	4.4	0	NS	NS	-
ENSG00000042980	ADAM28	protein_coding	NS	NS	-	7.23	7.2	0	NS	NS	-
ENSG00000149451	ADAM33	protein_coding	NS	NS	-	NS	NS	-	1.99	2	0
ENSG00000168615	ADAM9	protein_coding	3.62	3.6	0	3.64	3.6	0	3.46	3.5	0
ENSG00000151388	ADAMTS12	protein_coding	7.97	8	0	7.84	7.8	0	8.94	8.9	0
ENSG00000140470	ADAMTS17	protein_coding	0.44	-2.3	0	0.45	-2.2	0	0.42	-2.4	0
ENSG00000087116	ADAMTS2	protein_coding	3.38	3.4	0	3.2	3.2	0	4.82	4.8	0
ENSG00000154736	ADAMTS5	protein_coding	NS	NS	-	NS	NS	-	9.52	9.5	0
ENSG00000049192	ADAMTS6	protein_coding	2.54	2.5	0	NS	NS	-	2.73	2.7	0
ENSG00000156218	ADAMTSL3	protein_coding	NS	NS	-	NS	NS	-	0.13	-7.4	0
ENSG00000143382	ADAMTSL4	protein_coding	NS	NS	-	NS	NS	-	3.81	3.8	0
ENSG00000164742	ADCY1	protein_coding	NS	NS	-	0.51	-2	0	NS	NS	-
ENSG00000141433	ADCYAP1	protein_coding	NS	NS	-	NS	NS	-	0.41	-2.4	0
ENSG00000147872	ADFP	protein_coding	NS	NS	-	NS	NS	-	4.37	4.4	0
ENSG00000147576	ADHFE1	protein_coding	0.25	-4.1	0	0.25	-4	0	0.19	-5.1	0
ENSG00000148926	ADM	protein_coding	NS	NS	-	NS	NS	-	10.93	10.9	0
ENSG00000173020	ADRBK1	protein_coding	2.21	2.2	0	2.35	2.3	0	NS	NS	-
ENSG00000211848	AE000662.33	TR_J_gene	3.1	3.1	0	3.32	3.3	0	NS	NS	-
ENSG00000106624	AEBP1	protein_coding	4.45	4.5	0	4.36	4.4	0	NS	NS	-
ENSG00000181026	AEN	protein_coding	NS	NS	-	NS	NS	-	2.28	2.3	0
ENSG00000188512	AF279873.1	pseudogene	1.96	2	0	1.97	2	0	NS	NS	-
ENSG00000196526	AFAP1	protein_coding	2.93	2.9	0	2.79	2.8	0	4.04	4	0
ENSG00000119844	AFTPH	protein_coding	NS	NS	-	NS	NS	-	0.47	-2.1	0
ENSG00000204310	AGPAT1	protein_coding	NS	NS	-	NS	NS	-	3.2	3.2	0
ENSG00000106541	AGR2	protein_coding	NS	NS	-	5.26	5.3	0	NS	NS	-
ENSG00000135744	AGT	protein_coding	4.08	4.1	0	4.28	4.3	0	NS	NS	-
ENSG00000144891	AGTR1	protein_coding	NS	NS	-	NS	NS	-	2.91	2.9	0
ENSG00000185567	AHNAK2	protein_coding	12.53	12.5	0	10.77	10.8	0	26.6	26.6	0
ENSG00000106546	AHR	protein_coding	3.16	3.2	0	3.37	3.4	0	NS	NS	-
ENSG00000106305	AIMP2	protein_coding	NS	NS	-	NS	NS	-	2.46	2.5	0
ENSG00000106992	AK1	protein_coding	NS	NS	-	NS	NS	-	3.11	3.1	0
ENSG00000154027	AK5	protein_coding	NS	NS	-	NS	NS	-	48.44	48.4	0
ENSG00000131016	AKAP12	protein_coding	1.98	2	0	1.97	2	0	NS	NS	-
ENSG00000179841	AKAP5	protein_coding	NS	NS	-	2.02	2	0	NS	NS	-
ENSG00000127914	AKAP9	protein_coding	NS	NS	-	NS	NS	-	0.39	-2.6	0
ENSG00000135334	AKIRIN2	protein_coding	NS	NS	-	NS	NS	-	2.39	2.4	0
ENSG00000187134	AKR1C1	protein_coding	NS	NS	-	NS	NS	-	7.67	7.7	0
ENSG00000151632	AKR1C2	protein_coding	NS	NS	-	NS	NS	-	5.4	5.4	0
ENSG00000162482	AKR7A3	protein_coding	0.48	-2.1	0	0.51	-2	0	NS	NS	-

ENSG00000204673	AKT1S1	protein_coding	NS	NS	-	NS	NS	-	1.96	2	0
ENSG00000240625	AL136304.2	misc_RNA	0.49	-2	0	0.5	-2	0	NS	NS	-
ENSG00000197358	AL139023.1	pseudogene	0.19	-5.2	0	0.16	-6.2	0	NS	NS	-
ENSG00000241174	AL591215.1	misc_RNA	0.49	-2	0	0.49	-2	0	NS	NS	-
ENSG00000023330	ALAS1	protein_coding	NS	NS	-	NS	NS	-	2.18	2.2	0
ENSG00000163631	ALB	protein_coding	0.03	-28.9	0	0.04	-26.6	0	0.01	-90.9	0
ENSG00000165092	ALDH1A1	protein_coding	NS	NS	-	NS	NS	-	0.04	-22.5	0
ENSG00000144908	ALDH1L1	protein_coding	0.47	-2.1	0	0.47	-2.1	0	NS	NS	-
ENSG00000136010	ALDH1L2	protein_coding	0.35	-2.9	0	0.3	-3.4	0	NS	NS	-
ENSG00000116127	ALMS1	protein_coding	NS	NS	-	NS	NS	-	0.45	-2.2	0
ENSG00000012779	ALOX5	protein_coding	NS	NS	-	2.49	2.5	0	0.16	-6.2	0
ENSG00000132965	ALOX5AP	protein_coding	NS	NS	-	3.95	4	0	NS	NS	-
ENSG00000198796	ALPK2	protein_coding	NS	NS	-	NS	NS	-	11.98	12	0
ENSG00000163286	ALPPL2	protein_coding	0.49	-2	0	0.48	-2.1	0	NS	NS	-
ENSG00000155755	ALS2CR4	protein_coding	NS	NS	-	NS	NS	-	2.75	2.8	0
ENSG00000106927	AMBP	protein_coding	0.31	-3.2	0	NS	NS	-	0.12	-8.5	0
ENSG00000135409	AMHR2	protein_coding	0.39	-2.6	0	0.38	-2.6	0	NS	NS	-
ENSG00000139211	AMIGO2	protein_coding	3.7	3.7	0	3.88	3.9	0	NS	NS	-
ENSG00000114019	AMOTL2	protein_coding	NS	NS	-	NS	NS	-	3.86	3.9	0
ENSG00000145020	AMT	protein_coding	NS	NS	-	NS	NS	-	0.34	-3	0
ENSG00000240038	AMY2B	protein_coding	0.34	-3	0	0.36	-2.8	0	NS	NS	-
ENSG00000141552	ANAPC11	protein_coding	NS	NS	-	NS	NS	-	2.45	2.4	0
ENSG00000013523	ANGEL1	protein_coding	0.43	-2.3	0	0.44	-2.3	0	0.37	-2.7	0
ENSG00000136859	ANGPTL2	protein_coding	4.3	4.3	0	3.25	3.3	0	12.7	12.7	0
ENSG00000145362	ANK2	protein_coding	NS	NS	-	NS	NS	-	4.24	4.2	0
ENSG00000151150	ANK3	protein_coding	NS	NS	-	NS	NS	-	0.13	-7.8	0
ENSG00000154065	ANKRD29	protein_coding	NS	NS	-	NS	NS	-	3.56	3.6	0
ENSG00000132623	ANKRD5	protein_coding	0.26	-3.9	0	0.27	-3.7	0	NS	NS	-
ENSG00000151458	ANKRD50	protein_coding	3.42	3.4	0	3.51	3.5	0	2.73	2.7	0
ENSG00000181626	ANKRD62	protein_coding	0.44	-2.3	0	0.44	-2.3	0	NS	NS	-
ENSG00000011426	ANLN	protein_coding	8.99	9	0	9.28	9.3	0	6.67	6.7	0
ENSG00000131620	ANO1	protein_coding	NS	NS	-	6.81	6.8	0	NS	NS	-
ENSG00000171714	ANO5	protein_coding	0.44	-2.3	0	0.47	-2.1	0	0.18	-5.5	0
ENSG00000166825	ANPEP	protein_coding	0.17	-5.7	0	0.12	-8.3	0	NS	NS	-
ENSG00000169604	ANTXR1	protein_coding	6.48	6.5	0	6.42	6.4	0	6.99	7	0
ENSG00000163297	ANTXR2	protein_coding	3.49	3.5	0	3.42	3.4	0	NS	NS	-
ENSG00000182718	ANXA2	protein_coding	2.45	2.5	0	2.38	2.4	0	NS	NS	-
ENSG00000231991	ANXA2P2	pseudogene	2.18	2.2	0	2.16	2.2	0	NS	NS	-
ENSG00000196975	ANXA4	protein_coding	NS	NS	-	NS	NS	-	0.31	-3.2	0
ENSG00000164111	ANXA5	protein_coding	2.04	2	0	NS	NS	-	3.21	3.2	0
ENSG00000197043	ANXA6	protein_coding	NS	NS	-	NS	NS	-	3.41	3.4	0
ENSG00000143412	ANXA9	protein_coding	0.38	-2.6	0	NS	NS	-	0.16	-6.4	0

ENSG00000138356	AOX1	protein_coding	0.17	-5.9	0	0.11	-9.2	0	NS	NS	-
ENSG00000150687	AP000654.2	protein_coding	3.1	3.1	0	2.38	2.4	0	8.82	8.8	0
ENSG00000165895	AP000872.1	protein_coding	2.8	2.8	0	2.93	2.9	0	NS	NS	-
ENSG00000166250	AP000926.2	protein_coding	NS	NS	-	NS	NS	-	9.32	9.3	0
ENSG00000179240	AP002360.3	protein_coding	0.47	-2.1	0	0.49	-2.1	0	NS	NS	-
ENSG00000072958	AP1M1	protein_coding	NS	NS	-	NS	NS	-	3.14	3.1	0
ENSG00000129354	AP1M2	protein_coding	NS	NS	-	NS	NS	-	0.23	-4.4	0
ENSG00000106367	AP1S1	protein_coding	NS	NS	-	NS	NS	-	2.61	2.6	0
ENSG00000152056	AP1S3	protein_coding	NS	NS	-	3.38	3.4	0	NS	NS	-
ENSG00000196961	AP2A1	protein_coding	2.06	2.1	0	NS	NS	-	3.99	4	0
ENSG00000006125	AP2B1	protein_coding	2.16	2.2	0	2.1	2.1	0	2.68	2.7	0
ENSG00000161203	AP2M1	protein_coding	2.49	2.5	0	2.21	2.2	0	4.72	4.7	0
ENSG00000042753	AP2S1	protein_coding	NS	NS	-	NS	NS	-	2.73	2.7	0
ENSG00000070718	AP3M2	protein_coding	2.3	2.3	0	2.43	2.4	0	NS	NS	-
ENSG00000081014	AP4E1	protein_coding	NS	NS	-	NS	NS	-	2.16	2.2	0
ENSG00000105290	APLP1	protein_coding	0.27	-3.7	0	0.27	-3.7	0	NS	NS	-
ENSG00000124701	APOBEC2	protein_coding	0.39	-2.6	0	NS	NS	-	0.19	-5.1	0
ENSG00000244509	APOBEC3C	protein_coding	2.6	2.6	0	2.25	2.2	0	5.43	5.4	0
ENSG00000243811	APOBEC3D	protein_coding	2.13	2.1	0	2.21	2.2	0	NS	NS	-
ENSG00000239713	APOBEC3G	protein_coding	3	3	0	3.2	3.2	0	NS	NS	-
ENSG00000130208	APOC1	protein_coding	NS	NS	-	7.13	7.1	0	NS	NS	-
ENSG00000091583	APOH	protein_coding	0.28	-3.6	0	0.29	-3.4	0	NS	NS	-
ENSG00000100342	APOL1	protein_coding	9.09	9.1	0	10.07	10.1	0	NS	NS	-
ENSG00000221963	APOL6	protein_coding	2.39	2.4	0	2.43	2.4	0	NS	NS	-
ENSG00000184945	AQP12A	protein_coding	0.31	-3.2	0	0.31	-3.2	0	0.32	-3.1	0
ENSG00000185176	AQP12B	protein_coding	0.22	-4.5	0	0.22	-4.5	0	0.22	-4.6	0
ENSG00000103375	AQP8	protein_coding	0.05	-19.1	0	0.06	-17.9	0	NS	NS	-
ENSG00000047365	ARAP2	protein_coding	NS	NS	-	NS	NS	-	0.14	-7.3	0
ENSG00000164144	ARFIP1	protein_coding	2	2	0	2.03	2	0	NS	NS	-
ENSG00000175220	ARHGAP1	protein_coding	2.3	2.3	0	2.15	2.1	0	3.54	3.5	0
ENSG00000198826	ARHGAP11A	protein_coding	2.71	2.7	0	2.76	2.8	0	NS	NS	-
ENSG00000187951	ARHGAP11B	protein_coding	2.27	2.3	0	2.28	2.3	0	NS	NS	-
ENSG00000075884	ARHGAP15	protein_coding	NS	NS	-	NS	NS	-	0.33	-3	0
ENSG00000225485	ARHGAP23	protein_coding	NS	NS	-	NS	NS	-	2.22	2.2	0
ENSG00000145819	ARHGAP26	protein_coding	2.53	2.5	0	2.72	2.7	0	NS	NS	-
ENSG00000111348	ARHGDIB	protein_coding	NS	NS	-	4.25	4.3	0	NS	NS	-
ENSG00000242173	ARHGDIG	protein_coding	0.2	-5	0	0.2	-5	0	0.19	-5.1	0
ENSG00000104728	ARHGEF10	protein_coding	NS	NS	-	NS	NS	-	2.95	2.9	0
ENSG00000116584	ARHGEF2	protein_coding	2.61	2.6	0	2.52	2.5	0	NS	NS	-
ENSG00000150347	ARID5B	protein_coding	NS	NS	-	NS	NS	-	3.69	3.7	0
ENSG00000213465	ARL2	protein_coding	NS	NS	-	NS	NS	-	4.21	4.2	0
ENSG00000188042	ARL4C	protein_coding	3.55	3.6	0	3.36	3.4	0	5.08	5.1	0

ENSG00000175906	ARL4D	protein_coding	NS	NS	-	NS	NS	-	4.42	4.4	0
ENSG00000135931	ARMC9	protein_coding	NS	NS	-	NS	NS	-	3.74	3.7	0
ENSG00000184867	ARMCX2	protein_coding	NS	NS	-	NS	NS	-	2.28	2.3	0
ENSG00000133794	ARNTL	protein_coding	2.54	2.5	0	2.58	2.6	0	NS	NS	-
ENSG00000029153	ARNTL2	protein_coding	5.87	5.9	0	6.37	6.4	0	NS	NS	-
ENSG00000241685	ARPC1A	protein_coding	2.23	2.2	0	2.24	2.2	0	NS	NS	-
ENSG00000130429	ARPC1B	protein_coding	2.48	2.5	0	2.55	2.5	0	NS	NS	-
ENSG00000111229	ARPC3	protein_coding	1.96	2	0	1.96	2	0	NS	NS	-
ENSG00000157399	ARSE	protein_coding	0.22	-4.6	0	0.24	-4.2	0	0.08	-13.2	0
ENSG00000180801	ARSJ	protein_coding	NS	NS	-	NS	NS	-	3.63	3.6	0
ENSG00000156219	ART3	protein_coding	0.37	-2.7	0	0.37	-2.7	0	NS	NS	-
ENSG00000214435	AS3MT	protein_coding	0.36	-2.8	0	0.33	-3	0	NS	NS	-
ENSG00000153317	ASAP1	protein_coding	2.25	2.3	0	2.12	2.1	0	NS	NS	-
ENSG00000151693	ASAP2	protein_coding	2.77	2.8	0	2.87	2.9	0	NS	NS	-
ENSG00000112249	ASCC3	protein_coding	NS	NS	-	NS	NS	-	2.17	2.2	0
ENSG00000128203	ASPHD2	protein_coding	2.76	2.8	0	2.94	2.9	0	NS	NS	-
ENSG00000066279	ASPM	protein_coding	3.95	4	0	4.13	4.1	0	NS	NS	-
ENSG00000106819	ASPN	protein_coding	5.57	5.6	0	6.05	6	0	NS	NS	-
ENSG00000166669	ATF7IP2	protein_coding	NS	NS	-	NS	NS	-	0.17	-6	0
ENSG00000145782	ATG12	protein_coding	NS	NS	-	NS	NS	-	2.14	2.1	0
ENSG00000101974	ATP11C	protein_coding	NS	NS	-	1.97	2	0	NS	NS	-
ENSG00000127249	ATP13A4	protein_coding	NS	NS	-	NS	NS	-	0.29	-3.4	0
ENSG00000143153	ATP1B1	protein_coding	NS	NS	-	NS	NS	-	0.14	-7	0
ENSG00000129244	ATP1B2	protein_coding	NS	NS	-	NS	NS	-	0.51	-2	0
ENSG00000174437	ATP2A2	protein_coding	2.07	2.1	0	1.97	2	0	2.86	2.9	0
ENSG00000074370	ATP2A3	protein_coding	NS	NS	-	NS	NS	-	0.42	-2.4	0
ENSG00000058668	ATP2B4	protein_coding	2.43	2.4	0	2.28	2.3	0	NS	NS	-
ENSG00000017260	ATP2C1	protein_coding	2.07	2.1	0	2.11	2.1	0	NS	NS	-
ENSG00000105675	ATP4A	protein_coding	0.29	-3.5	0	0.28	-3.6	0	NS	NS	-
ENSG00000241468	ATP5J2	protein_coding	2.13	2.1	0	1.98	2	0	NS	NS	-
ENSG00000159720	ATP6V0D1	protein_coding	NS	NS	-	NS	NS	-	3.69	3.7	0
ENSG00000131100	ATP6V1E1	protein_coding	NS	NS	-	NS	NS	-	2.33	2.3	0
ENSG00000128524	ATP6V1F	protein_coding	NS	NS	-	NS	NS	-	3.68	3.7	0
ENSG00000124406	ATP8A1	protein_coding	NS	NS	-	NS	NS	-	0.09	-11.5	0
ENSG00000143515	ATP8B2	protein_coding	2.41	2.4	0	2.04	2	0	5.42	5.4	0
ENSG00000104043	ATP8B4	protein_coding	2.51	2.5	0	2.62	2.6	0	NS	NS	-
ENSG00000134146	ATPBD4	protein_coding	NS	NS	-	NS	NS	-	0.36	-2.8	0
ENSG00000167601	AXL	protein_coding	2.6	2.6	0	2.29	2.3	0	5.03	5	0
ENSG00000160862	AZGP1	protein_coding	0.12	-8.4	0	0.13	-7.6	0	0.02	-49.1	0
ENSG00000166710	B2M	protein_coding	3.25	3.3	0	3.39	3.4	0	NS	NS	-
ENSG00000162885	B3GALNT2	protein_coding	0.39	-2.6	0	0.39	-2.6	0	NS	NS	-
ENSG00000162630	B3GALT2	protein_coding	NS	NS	-	NS	NS	-	5.5	5.5	0

ENSG00000187676	B3GALTL	protein_coding	NS	NS	-	NS	NS	-	2.19	2.2	0
ENSG00000179913	B3GNT3	protein_coding	NS	NS	-	3.49	3.5	0	NS	NS	-
ENSG00000086062	B4GALT1	protein_coding	2.07	2.1	0	1.98	2	0	2.81	2.8	0
ENSG00000121578	B4GALT4	protein_coding	NS	NS	-	2.19	2.2	0	NS	NS	-
ENSG00000186318	BACE1	protein_coding	0.2	-4.9	0	0.18	-5.5	0	NS	NS	-
ENSG00000112208	BAG2	protein_coding	NS	NS	-	NS	NS	-	6.09	6.1	0
ENSG00000156735	BAG4	protein_coding	NS	NS	-	NS	NS	-	2.44	2.4	0
ENSG00000030110	BAK1	protein_coding	1.99	2	0	2.01	2	0	NS	NS	-
ENSG00000125888	BANF2	protein_coding	0.15	-6.7	0	0.15	-6.7	0	NS	NS	-
ENSG00000176788	BASP1	protein_coding	NS	NS	-	NS	NS	-	4.82	4.8	0
ENSG00000204469	BAT2	protein_coding	NS	NS	-	NS	NS	-	2.05	2	0
ENSG00000087088	BAX	protein_coding	NS	NS	-	NS	NS	-	2.61	2.6	0
ENSG00000140463	BBS4	protein_coding	NS	NS	-	NS	NS	-	2.17	2.2	0
ENSG00000138686	BBS7	protein_coding	NS	NS	-	NS	NS	-	2.73	2.7	0
ENSG00000187244	BCAM	protein_coding	NS	NS	-	NS	NS	-	0.41	-2.4	0
ENSG00000137936	BCAR3	protein_coding	NS	NS	-	NS	NS	-	3.08	3.1	0
ENSG00000064787	BCAS1	protein_coding	7.55	7.5	0	8.29	8.3	0	NS	NS	-
ENSG00000105552	BCAT2	protein_coding	0.47	-2.1	0	0.46	-2.2	0	NS	NS	-
ENSG00000142867	BCL10	protein_coding	2.18	2.2	0	2.19	2.2	0	2.14	2.1	0
ENSG00000175730	BCL2L7P1	pseudogene	1.95	2	0	1.96	2	0	NS	NS	-
ENSG00000186174	BCL9L	protein_coding	NS	NS	-	NS	NS	-	2.64	2.6	0
ENSG00000151917	BEND6	protein_coding	NS	NS	-	NS	NS	-	5.96	6	0
ENSG00000133134	BEX2	protein_coding	0.38	-2.6	0	0.38	-2.6	0	0.34	-3	0
ENSG00000182492	BGN	protein_coding	3.93	3.9	0	4.15	4.2	0	2.19	2.2	0
ENSG00000180535	BHLHA15	protein_coding	0.1	-10.5	0	0.1	-10.2	0	0.07	-13.9	0
ENSG00000151746	BICD1	protein_coding	2.57	2.6	0	2.67	2.7	0	NS	NS	-
ENSG00000023445	BIRC3	protein_coding	NS	NS	-	3.97	4	0	NS	NS	-
ENSG00000197299	BLM	protein_coding	2.05	2	0	2.13	2.1	0	NS	NS	-
ENSG00000095585	BLNK	protein_coding	NS	NS	-	NS	NS	-	0.23	-4.4	0
ENSG00000168487	BMP1	protein_coding	NS	NS	-	NS	NS	-	2.45	2.5	0
ENSG00000138756	BMP2K	protein_coding	2.1	2.1	0	2	2	0	NS	NS	-
ENSG00000164619	BMPER	protein_coding	NS	NS	-	NS	NS	-	6.83	6.8	0
ENSG00000204217	BMPR2	protein_coding	2.02	2	0	2.07	2.1	0	NS	NS	-
ENSG00000169594	BNC1	protein_coding	NS	NS	-	NS	NS	-	10.32	10.3	0
ENSG00000173068	BNC2	protein_coding	3.35	3.3	0	3.15	3.1	0	NS	NS	-
ENSG00000176171	BNIP3	protein_coding	0.18	-5.7	0	0.15	-6.5	0	NS	NS	-
ENSG00000162813	BPNT1	protein_coding	2.3	2.3	0	2.35	2.3	0	NS	NS	-
ENSG00000139618	BRCA2	protein_coding	2.1	2.1	0	2.16	2.2	0	NS	NS	-
ENSG00000164713	BRI3	protein_coding	NS	NS	-	NS	NS	-	2.63	2.6	0
ENSG00000174672	BRSK2	protein_coding	0.28	-3.5	0	0.28	-3.6	0	0.33	-3.1	0
ENSG00000119411	BSPRY	protein_coding	0.32	-3.1	0	0.34	-2.9	0	0.17	-5.8	0
ENSG00000109743	BST1	protein_coding	2.49	2.5	0	2.3	2.3	0	4.06	4.1	0

ENSG00000130303	BST2	protein_coding	NS	NS	-	2.51	2.5	0	NS	NS	-
ENSG00000169814	BTD	protein_coding	NS	NS	-	0.5	-2	0	NS	NS	-
ENSG00000159388	BTG2	protein_coding	0.24	-4.1	0	0.25	-4.1	0	0.2	-5	0
ENSG00000010671	BTK	protein_coding	NS	NS	-	2.42	2.4	0	NS	NS	-
ENSG00000026950	BTN3A1	protein_coding	2.02	2	0	2.14	2.1	0	NS	NS	-
ENSG00000186470	BTN3A2	protein_coding	3.27	3.3	0	3.5	3.5	0	NS	NS	-
ENSG00000111801	BTN3A3	protein_coding	2.72	2.7	0	2.85	2.9	0	NS	NS	-
ENSG00000169679	BUB1	protein_coding	3.1	3.1	0	3.25	3.2	0	NS	NS	-
ENSG00000156970	BUB1B	protein_coding	2.35	2.4	0	2.43	2.4	0	NS	NS	-
ENSG00000154473	BUB3	protein_coding	2.24	2.2	0	2.28	2.3	0	NS	NS	-
ENSG00000106245	BUD31	protein_coding	NS	NS	-	NS	NS	-	2.12	2.1	0
ENSG00000112276	BVES	protein_coding	NS	NS	-	NS	NS	-	8.05	8	0
ENSG00000180525	C10orf108	protein_coding	0.43	-2.3	0	0.44	-2.3	0	NS	NS	-
ENSG00000165813	C10orf118	protein_coding	NS	NS	-	NS	NS	-	0.29	-3.5	0
ENSG00000173124	C10orf129	protein_coding	0.36	-2.8	0	0.35	-2.8	0	NS	NS	-
ENSG00000166272	C10orf26	protein_coding	2.33	2.3	0	2.33	2.3	0	NS	NS	-
ENSG00000165633	C10orf72	protein_coding	NS	NS	-	NS	NS	-	5.07	5.1	0
ENSG00000148735	C10orf81	protein_coding	NS	NS	-	NS	NS	-	0.1	-10.4	0
ENSG00000171067	C11orf24	protein_coding	2.05	2.1	0	NS	NS	-	3.56	3.6	0
ENSG00000110427	C11orf41	protein_coding	NS	NS	-	NS	NS	-	3.82	3.8	0
ENSG00000182919	C11orf54	protein_coding	0.5	-2	0	NS	NS	-	0.28	-3.6	0
ENSG00000149357	C11orf59	protein_coding	2.07	2.1	0	NS	NS	-	4.56	4.6	0
ENSG00000175573	C11orf68	protein_coding	NS	NS	-	NS	NS	-	2.18	2.2	0
ENSG00000204856	C12orf24	protein_coding	NS	NS	-	NS	NS	-	3.14	3.1	0
ENSG00000134548	C12orf39	protein_coding	0.04	-23	0	0.04	-23.2	0	NS	NS	-
ENSG00000047621	C12orf4	protein_coding	2.06	2.1	0	NS	NS	-	2.22	2.2	0
ENSG00000151287	C13orf27	protein_coding	NS	NS	-	NS	NS	-	2.76	2.8	0
ENSG00000165480	C13orf3	protein_coding	3.02	3	0	3.05	3.1	0	NS	NS	-
ENSG00000100557	C14orf105	protein_coding	NS	NS	-	NS	NS	-	0.06	-17.9	0
ENSG00000179933	C14orf119	protein_coding	NS	NS	-	NS	NS	-	2.87	2.9	0
ENSG00000227051	C14orf132	protein_coding	NS	NS	-	NS	NS	-	3.53	3.5	0
ENSG00000139971	C14orf37	protein_coding	2.08	2.1	0	NS	NS	-	3.46	3.5	0
ENSG00000178761	C15orf17	protein_coding	NS	NS	-	NS	NS	-	1.96	2	0
ENSG00000166920	C15orf48	protein_coding	6.13	6.1	0	6.62	6.6	0	NS	NS	-
ENSG00000103148	C16orf35	protein_coding	NS	NS	-	NS	NS	-	2.15	2.2	0
ENSG00000124074	C16orf48	protein_coding	0.48	-2.1	0	0.48	-2.1	0	NS	NS	-
ENSG00000103544	C16orf62	protein_coding	NS	NS	-	NS	NS	-	2.14	2.1	0
ENSG00000070761	C16orf80	protein_coding	NS	NS	-	NS	NS	-	2.78	2.8	0
ENSG00000167861	C17orf28	protein_coding	0.44	-2.3	0	0.47	-2.1	0	0.13	-7.5	0
ENSG00000125319	C17orf53	protein_coding	NS	NS	-	NS	NS	-	2.25	2.3	0
ENSG00000166845	C18orf54	protein_coding	NS	NS	-	NS	NS	-	2.56	2.6	0
ENSG00000167644	C19orf33	protein_coding	NS	NS	-	3.17	3.2	0	NS	NS	-

ENSG00000131944	C19orf40	protein_coding	NS	NS	-	NS	NS	-	2.02	2	0
ENSG00000105771	C19orf61	protein_coding	2.12	2.1	0	2.07	2.1	0	2.53	2.5	0
ENSG00000106392	C1GALT1	protein_coding	2.69	2.7	0	2.76	2.8	0	NS	NS	-
ENSG00000117597	C1orf107	protein_coding	NS	NS	-	NS	NS	-	2.54	2.5	0
ENSG00000175147	C1orf126	processed_transcript	0.35	-2.9	0	0.38	-2.7	0	0.12	-8.3	0
ENSG00000160767	C1orf2	protein_coding	NS	NS	-	NS	NS	-	2.07	2.1	0
ENSG00000118292	C1orf54	protein_coding	NS	NS	-	2	2	0	NS	NS	-
ENSG00000162819	C1orf58	protein_coding	NS	NS	-	1.97	2	0	NS	NS	-
ENSG00000198715	C1orf85	protein_coding	NS	NS	-	NS	NS	-	7.87	7.9	0
ENSG00000173947	C1orf88	protein_coding	0.44	-2.3	0	0.42	-2.4	0	NS	NS	-
ENSG00000173372	C1QA	protein_coding	NS	NS	-	3.35	3.4	0	NS	NS	-
ENSG00000173369	C1QB	protein_coding	NS	NS	-	3.97	4	0	NS	NS	-
ENSG00000159189	C1QC	protein_coding	NS	NS	-	3.69	3.7	0	NS	NS	-
ENSG00000159403	C1R	protein_coding	3.35	3.3	0	3.25	3.2	0	NS	NS	-
ENSG00000182326	C1S	protein_coding	3.66	3.7	0	3.68	3.7	0	NS	NS	-
ENSG00000125869	C20orf103	protein_coding	7.1	7.1	0	7.89	7.9	0	NS	NS	-
ENSG00000229230	C20orf127	pseudogene	0.42	-2.4	0	0.42	-2.4	0	NS	NS	-
ENSG00000188559	C20orf74	protein_coding	NS	NS	-	NS	NS	-	0.22	-4.6	0
ENSG00000149346	C20orf94	protein_coding	NS	NS	-	NS	NS	-	0.45	-2.2	0
ENSG00000235374	C21orf122	pseudogene	0.44	-2.3	0	0.43	-2.3	0	NS	NS	-
ENSG00000215386	C21orf34	lincRNA	3.23	3.2	0	3.4	3.4	0	NS	NS	-
ENSG00000205929	C21orf62	protein_coding	0.19	-5.3	0	0.2	-5	0	NS	NS	-
ENSG00000100364	C22orf9	protein_coding	2.16	2.2	0	2.02	2	0	3.28	3.3	0
ENSG00000205502	C2CD4B	protein_coding	0.23	-4.4	0	0.23	-4.4	0	0.24	-4.2	0
ENSG00000213699	C2orf18	protein_coding	NS	NS	-	NS	NS	-	2.46	2.5	0
ENSG00000183833	C3orf15	protein_coding	0.26	-3.8	0	0.28	-3.6	0	0.14	-7.1	0
ENSG00000114529	C3orf52	protein_coding	0.25	-4	0	0.27	-3.7	0	0.08	-12.5	0
ENSG00000174899	C3orf55	protein_coding	NS	NS	-	NS	NS	-	5.58	5.6	0
ENSG00000180611	C3orf59	protein_coding	2.01	2	0	2.06	2.1	0	NS	NS	-
ENSG00000163378	C3orf64	protein_coding	NS	NS	-	NS	NS	-	2.96	3	0
ENSG00000164074	C4orf29	protein_coding	0.42	-2.4	0	0.43	-2.3	0	NS	NS	-
ENSG00000173376	C4orf31	protein_coding	4.09	4.1	0	3.92	3.9	0	NS	NS	-
ENSG00000106804	C5	protein_coding	0.16	-6.3	0	0.17	-6	0	NS	NS	-
ENSG00000134986	C5orf13	protein_coding	4.8	4.8	0	4.63	4.6	0	6.16	6.2	0
ENSG00000113583	C5orf15	protein_coding	2.03	2	0	1.99	2	0	2.38	2.4	0
ENSG00000152620	C5orf33	protein_coding	0.43	-2.3	0	0.44	-2.3	0	0.34	-2.9	0
ENSG00000039537	C6	protein_coding	0.27	-3.7	0	NS	NS	-	0.04	-23.6	0
ENSG00000146386	C6orf115	protein_coding	3.53	3.5	0	3.38	3.4	0	NS	NS	-
ENSG00000196748	C6orf126	protein_coding	0.51	-2	0	0.5	-2	0	NS	NS	-
ENSG00000198937	C6orf129	protein_coding	NS	NS	-	NS	NS	-	3.42	3.4	0
ENSG00000124596	C6orf130	protein_coding	NS	NS	-	NS	NS	-	0.42	-2.4	0
ENSG00000164430	C6orf150	protein_coding	2.3	2.3	0	2.41	2.4	0	NS	NS	-

ENSG00000233237	C6orf155	lincRNA	0.49	-2	0	0.49	-2	0	NS	NS	-
ENSG00000203760	C6orf173	protein_coding	NS	NS	-	NS	NS	-	2.33	2.3	0
ENSG00000203778	C6orf225	protein_coding	NS	NS	-	NS	NS	-	4.05	4	0
ENSG00000112936	C7	protein_coding	NS	NS	-	NS	NS	-	0.01	-165.9	0
ENSG00000188732	C7orf46	protein_coding	NS	NS	-	NS	NS	-	0.15	-6.5	0
ENSG00000175854	C9orf119	protein_coding	NS	NS	-	NS	NS	-	2.26	2.3	0
ENSG00000066697	C9orf30	protein_coding	NS	NS	-	NS	NS	-	4.01	4	0
ENSG00000147894	C9orf72	protein_coding	NS	NS	-	NS	NS	-	0.28	-3.6	0
ENSG00000176058	C9orf75	protein_coding	0.48	-2.1	0	0.48	-2.1	0	NS	NS	-
ENSG00000185015	CA13	protein_coding	2.93	2.9	0	3.08	3.1	0	NS	NS	-
ENSG00000104267	CA2	protein_coding	NS	NS	-	NS	NS	-	0.08	-12.4	0
ENSG00000167434	CA4	protein_coding	0.43	-2.3	0	0.42	-2.4	0	0.44	-2.3	0
ENSG00000163050	CABC1	protein_coding	0.36	-2.8	0	0.37	-2.7	0	0.25	-4	0
ENSG00000134508	CABLES1	protein_coding	NS	NS	-	NS	NS	-	2.98	3	0
ENSG00000153956	CACNA2D1	protein_coding	2.46	2.5	0	2.32	2.3	0	NS	NS	-
ENSG00000167535	CACNB3	protein_coding	2.45	2.4	0	2.31	2.3	0	3.53	3.5	0
ENSG00000084774	CAD	protein_coding	NS	NS	-	NS	NS	-	1.96	2	0
ENSG00000105767	CADM4	protein_coding	0.51	-2	0	0.51	-2	0	0.47	-2.1	0
ENSG00000081803	CADPS2	protein_coding	NS	NS	-	NS	NS	-	0.16	-6.3	0
ENSG00000122786	CALD1	protein_coding	3.69	3.7	0	3.54	3.5	0	NS	NS	-
ENSG00000138172	CALHM2	protein_coding	NS	NS	-	NS	NS	-	3.39	3.4	0
ENSG00000143933	CALM2	protein_coding	NS	NS	-	NS	NS	-	1.97	2	0
ENSG00000128595	CALU	protein_coding	3.09	3.1	0	2.67	2.7	0	6.42	6.4	0
ENSG00000183049	CAMK1D	protein_coding	NS	NS	-	NS	NS	-	0.36	-2.8	0
ENSG00000145349	CAMK2D	protein_coding	2.56	2.6	0	2.42	2.4	0	3.67	3.7	0
ENSG00000148660	CAMK2G	protein_coding	NS	NS	-	NS	NS	-	2.38	2.4	0
ENSG00000118200	CAMSAP1L1	protein_coding	2.46	2.5	0	2.4	2.4	0	NS	NS	-
ENSG00000131236	CAP1	protein_coding	2.88	2.9	0	2.83	2.8	0	3.26	3.3	0
ENSG00000042493	CAPG	protein_coding	4.94	4.9	0	5	5	0	4.41	4.4	0
ENSG00000162909	CAPN2	protein_coding	2.15	2.1	0	2.09	2.1	0	2.64	2.6	0
ENSG00000077549	CAPZB	protein_coding	2.35	2.3	0	2.25	2.3	0	3.1	3.1	0
ENSG00000198286	CARD11	protein_coding	3.18	3.2	0	3.47	3.5	0	NS	NS	-
ENSG00000132357	CARD6	protein_coding	2.33	2.3	0	2.32	2.3	0	NS	NS	-
ENSG00000142453	CARM1	protein_coding	NS	NS	-	NS	NS	-	2.57	2.6	0
ENSG00000137812	CASC5	protein_coding	4.9	4.9	0	5.13	5.1	0	NS	NS	-
ENSG00000147044	CASK	protein_coding	2.11	2.1	0	2.2	2.2	0	NS	NS	-
ENSG00000164305	CASP3	protein_coding	2.21	2.2	0	2.17	2.2	0	2.54	2.5	0
ENSG00000064012	CASP8	protein_coding	NS	NS	-	2.07	2.1	0	NS	NS	-
ENSG00000133962	CATSPERB	protein_coding	NS	NS	-	NS	NS	-	0.05	-18.5	0
ENSG00000107745	CBARA1	protein_coding	2.11	2.1	0	2	2	0	3.04	3	0
ENSG00000067955	CBFB	protein_coding	2.04	2	0	2.14	2.1	0	NS	NS	-
ENSG00000110395	CBL	protein_coding	2.46	2.5	0	2.47	2.5	0	NS	NS	-

ENSG00000114423	CBLB	protein_coding	1.97	2	0	1.98	2	0	NS	NS	-
ENSG00000160200	CBS	protein_coding	0.16	-6.2	0	0.15	-6.9	0	NS	NS	-
ENSG00000172785	CBWD1	protein_coding	0.36	-2.8	0	0.39	-2.6	0	0.12	-8.2	0
ENSG00000183741	CBX6	protein_coding	NS	NS	-	NS	NS	-	2.12	2.1	0
ENSG00000183287	CCBE1	protein_coding	NS	NS	-	NS	NS	-	13.56	13.6	0
ENSG00000156026	CCDC109A	protein_coding	2.95	3	0	3.02	3	0	NS	NS	-
ENSG00000005059	CCDC109B	protein_coding	3.17	3.2	0	3.15	3.2	0	NS	NS	-
ENSG00000168491	CCDC110	protein_coding	0.19	-5.4	0	0.19	-5.2	0	NS	NS	-
ENSG00000164366	CCDC127	protein_coding	NS	NS	-	NS	NS	-	2.2	2.2	0
ENSG00000163492	CCDC141	protein_coding	0.33	-3.1	0	0.34	-2.9	0	0.23	-4.4	0
ENSG00000183978	CCDC56	protein_coding	0.51	-2	0	0.49	-2	0	NS	NS	-
ENSG00000162069	CCDC64B	protein_coding	0.4	-2.5	0	0.4	-2.5	0	NS	NS	-
ENSG00000216937	CCDC7	protein_coding	NS	NS	-	NS	NS	-	0.48	-2.1	0
ENSG00000091986	CCDC80	protein_coding	NS	NS	-	NS	NS	-	4.59	4.6	0
ENSG00000015133	CCDC88C	protein_coding	NS	NS	-	NS	NS	-	0.42	-2.4	0
ENSG00000119242	CCDC92	protein_coding	NS	NS	-	NS	NS	-	2.56	2.6	0
ENSG00000040275	CCDC99	protein_coding	2.74	2.7	0	2.48	2.5	0	NS	NS	-
ENSG00000185972	CCIN	protein_coding	NS	NS	-	NS	NS	-	1.96	2	0
ENSG00000110148	CCKBR	protein_coding	0.13	-8	0	0.12	-8.3	0	NS	NS	-
ENSG00000137077	CCL21	protein_coding	NS	NS	-	NS	NS	-	0.2	-4.9	0
ENSG00000145386	CCNA2	protein_coding	2.63	2.6	0	2.64	2.6	0	NS	NS	-
ENSG00000157456	CCNB2	protein_coding	4.96	5	0	5.08	5.1	0	NS	NS	-
ENSG00000156535	CD109	protein_coding	9.16	9.2	0	7.37	7.4	0	23.4	23.4	0
ENSG00000174807	CD248	protein_coding	NS	NS	-	2.62	2.6	0	18.32	18.3	0
ENSG00000185275	CD24L4	pseudogene	NS	NS	-	NS	NS	-	0.01	-136.1	0
ENSG00000103855	CD276	protein_coding	2.47	2.5	0	2.06	2.1	0	5.8	5.8	0
ENSG00000174059	CD34	protein_coding	NS	NS	-	NS	NS	-	0.22	-4.6	0
ENSG00000117091	CD48	protein_coding	NS	NS	-	NS	NS	-	0.46	-2.2	0
ENSG00000169442	CD52	protein_coding	NS	NS	-	4.21	4.2	0	NS	NS	-
ENSG00000143119	CD53	protein_coding	NS	NS	-	3.44	3.4	0	0.11	-9.3	0
ENSG00000196352	CD55	protein_coding	2.9	2.9	0	3.04	3	0	NS	NS	-
ENSG00000116815	CD58	protein_coding	2.51	2.5	0	2.65	2.7	0	NS	NS	-
ENSG00000135404	CD63	protein_coding	NS	NS	-	NS	NS	-	2.33	2.3	0
ENSG00000129226	CD68	protein_coding	3.64	3.6	0	3.56	3.6	0	4.25	4.3	0
ENSG00000019582	CD74	protein_coding	NS	NS	-	3.29	3.3	0	NS	NS	-
ENSG00000121594	CD80	protein_coding	NS	NS	-	2.52	2.5	0	NS	NS	-
ENSG00000066294	CD84	protein_coding	NS	NS	-	3.02	3	0	NS	NS	-
ENSG00000010278	CD9	protein_coding	2.61	2.6	0	2.73	2.7	0	NS	NS	-
ENSG00000125810	CD93	protein_coding	NS	NS	-	NS	NS	-	0.19	-5.4	0
ENSG00000123146	CD97	protein_coding	NS	NS	-	1.98	2	0	NS	NS	-
ENSG00000101224	CDC25B	protein_coding	2.89	2.9	0	2.47	2.5	0	6.25	6.3	0
ENSG00000149798	CDC42EP2	protein_coding	NS	NS	-	NS	NS	-	3.51	3.5	0

ENSG00000163171	CDC42EP3	protein_coding	NS	NS	-	NS	NS	-	2.63	2.6	0
ENSG00000158985	CDC42SE2	protein_coding	NS	NS	-	NS	NS	-	0.3	-3.4	0
ENSG00000031081	CDGAP	protein_coding	2.01	2	0	1.95	2	0	NS	NS	-
ENSG00000039068	CDH1	protein_coding	NS	NS	-	NS	NS	-	0.03	-32.3	0
ENSG00000140937	CDH11	protein_coding	10.49	10.5	0	9.99	10	0	14.48	14.5	0
ENSG00000140945	CDH13	protein_coding	NS	NS	-	NS	NS	-	14.09	14.1	0
ENSG00000170558	CDH2	protein_coding	NS	NS	-	NS	NS	-	5.6	5.6	0
ENSG00000107736	CDH23	protein_coding	0.31	-3.2	0	0.32	-3.1	0	NS	NS	-
ENSG00000062038	CDH3	protein_coding	7.36	7.4	0	8.19	8.2	0	NS	NS	-
ENSG00000179776	CDH5	protein_coding	NS	NS	-	NS	NS	-	0.29	-3.5	0
ENSG00000113361	CDH6	protein_coding	4.93	4.9	0	5.35	5.3	0	NS	NS	-
ENSG00000108465	CDK5RAP3	protein_coding	NS	NS	-	NS	NS	-	0.34	-2.9	0
ENSG00000124762	CDKN1A	protein_coding	NS	NS	-	NS	NS	-	6.25	6.2	0
ENSG00000100526	CDKN3	protein_coding	3.92	3.9	0	3.86	3.9	0	NS	NS	-
ENSG00000185267	CDNF	protein_coding	0.33	-3	0	0.33	-3	0	NS	NS	-
ENSG00000064309	CDON	protein_coding	NS	NS	-	NS	NS	-	4.8	4.8	0
ENSG00000163624	CDS1	protein_coding	NS	NS	-	NS	NS	-	0.33	-3	0
ENSG00000079385	CEACAM1	protein_coding	6.29	6.3	0	6.96	7	0	NS	NS	-
ENSG00000105388	CEACAM5	protein_coding	51.72	51.7	0	58.08	58.1	0	NS	NS	-
ENSG00000086548	CEACAM6	protein_coding	NS	NS	-	23.23	23.2	0	0.2	-4.9	0
ENSG00000172216	CEBPB	protein_coding	NS	NS	-	NS	NS	-	2.38	2.4	0
ENSG00000170835	CEL	protein_coding	0.1	-9.9	0	0.11	-8.8	0	0	-239.6	0
ENSG00000142615	CELA2A	protein_coding	0.09	-11.6	0	0.1	-10.4	0	0	-256.5	0
ENSG00000215704	CELA2B	protein_coding	0.1	-9.6	0	0.12	-8.6	0	0.01	-128.2	0
ENSG00000142789	CELA3A	protein_coding	0.15	-6.5	0	NS	NS	-	0.01	-161.7	0
ENSG00000219073	CELA3B	protein_coding	0.14	-7.2	0	0.15	-6.5	0	0.01	-99.6	0
ENSG00000170827	CELP	pseudogene	0.07	-14.4	0	0.08	-13.2	0	0.02	-65.9	0
ENSG00000075275	CELSR1	protein_coding	NS	NS	-	2.38	2.4	0	NS	NS	-
ENSG00000138778	CENPE	protein_coding	2.68	2.7	0	2.74	2.7	0	2.2	2.2	0
ENSG00000117724	CENPF	protein_coding	5.86	5.9	0	6.01	6	0	NS	NS	-
ENSG00000102384	CENPI	protein_coding	2.83	2.8	0	2.85	2.8	0	2.66	2.7	0
ENSG00000123219	CENPK	protein_coding	3.46	3.5	0	3.6	3.6	0	NS	NS	-
ENSG00000166451	CENPN	protein_coding	NS	NS	-	NS	NS	-	3.41	3.4	0
ENSG00000138092	CENPO	protein_coding	NS	NS	-	NS	NS	-	2.49	2.5	0
ENSG00000174799	CEP135	protein_coding	1.98	2	0	2.03	2	0	NS	NS	-
ENSG00000143702	CEP170	protein_coding	3.32	3.3	0	3.29	3.3	0	3.51	3.5	0
ENSG00000138180	CEP55	protein_coding	3.49	3.5	0	3.6	3.6	0	NS	NS	-
ENSG00000114107	CEP70	protein_coding	0.48	-2.1	0	NS	NS	-	0.2	-5.1	0
ENSG00000167123	CERCAM	protein_coding	NS	NS	-	NS	NS	-	4.56	4.6	0
ENSG00000243649	CFB	protein_coding	3.51	3.5	0	3.78	3.8	0	NS	NS	-
ENSG00000000971	CFH	protein_coding	3.22	3.2	0	3.49	3.5	0	NS	NS	-
ENSG00000244414	CFHR1	protein_coding	NS	NS	-	2.03	2	0	NS	NS	-

ENSG00000205403	CFI	protein_coding	NS	NS	-	NS	NS	-	0.17	-5.9	0
ENSG00000172757	CFL1	protein_coding	2.82	2.8	0	2.69	2.7	0	3.8	3.8	0
ENSG00000165410	CFL2	protein_coding	NS	NS	-	NS	NS	-	4.76	4.8	0
ENSG00000001626	CFTR	protein_coding	NS	NS	-	NS	NS	-	0.01	-131	0
ENSG00000104818	CGB2	protein_coding	0.42	-2.4	0	0.43	-2.3	0	NS	NS	-
ENSG00000143375	CGN	protein_coding	NS	NS	-	NS	NS	-	0.38	-2.6	0
ENSG00000128849	CGNL1	protein_coding	NS	NS	-	NS	NS	-	0.25	-4	0
ENSG00000138135	CH25H	protein_coding	NS	NS	-	NS	NS	-	9.67	9.7	0
ENSG00000128965	CHAC1	protein_coding	0.2	-5.1	0	0.17	-6	0	NS	NS	-
ENSG00000136457	CHAD	protein_coding	0.19	-5.2	0	0.19	-5.3	0	0.21	-4.8	0
ENSG00000171316	CHD7	protein_coding	NS	NS	-	NS	NS	-	0.22	-4.6	0
ENSG00000016391	CHDH	protein_coding	0.5	-2	0	NS	NS	-	0.32	-3.2	0
ENSG00000149554	CHEK1	protein_coding	2.56	2.6	0	2.35	2.4	0	4.22	4.2	0
ENSG00000110721	CHKA	protein_coding	NS	NS	-	NS	NS	-	0.31	-3.2	0
ENSG00000203668	CHML	protein_coding	2.53	2.5	0	2.63	2.6	0	NS	NS	-
ENSG00000164695	CHMP4C	protein_coding	NS	NS	-	NS	NS	-	0.16	-6.4	0
ENSG00000086065	CHMP5	protein_coding	2.68	2.7	0	2.71	2.7	0	2.47	2.5	0
ENSG00000106069	CHN2	protein_coding	0.38	-2.6	0	0.42	-2.4	0	0.09	-10.7	0
ENSG00000133019	CHRM3	protein_coding	0.23	-4.3	0	0.25	-4	0	0.12	-8.2	0
ENSG00000170175	CHRNB1	protein_coding	0.45	-2.2	0	0.45	-2.2	0	NS	NS	-
ENSG00000171310	CHST11	protein_coding	4.02	4	0	4.28	4.3	0	NS	NS	-
ENSG00000122863	CHST3	protein_coding	NS	NS	-	NS	NS	-	2.18	2.2	0
ENSG00000154080	CHST9	protein_coding	0.28	-3.6	0	0.3	-3.3	0	0.08	-11.9	0
ENSG00000179583	CIITA	protein_coding	NS	NS	-	2.98	3	0	NS	NS	-
ENSG00000099622	CIRBP	protein_coding	0.46	-2.2	0	0.47	-2.1	0	0.31	-3.2	0
ENSG00000179862	CITED4	protein_coding	0.49	-2	0	0.49	-2	0	NS	NS	-
ENSG00000136108	CKAP2	protein_coding	4.39	4.4	0	4.19	4.2	0	5.96	6	0
ENSG00000175216	CKAP5	protein_coding	1.99	2	0	NS	NS	-	2.32	2.3	0
ENSG00000217555	CKLF	protein_coding	3.71	3.7	0	3.96	4	0	NS	NS	-
ENSG00000123975	CKS2	protein_coding	9.5	9.5	0	8.37	8.4	0	18.59	18.6	0
ENSG00000186510	CLCNKA	protein_coding	0.47	-2.1	0	0.47	-2.1	0	NS	NS	-
ENSG00000163347	CLDN1	protein_coding	NS	NS	-	NS	NS	-	0.09	-10.8	0
ENSG00000134873	CLDN10	protein_coding	NS	NS	-	NS	NS	-	0.05	-20.6	0
ENSG00000013297	CLDN11	protein_coding	NS	NS	-	NS	NS	-	21.05	21	0
ENSG00000066405	CLDN18	protein_coding	NS	NS	-	14.28	14.3	0	NS	NS	-
ENSG00000165215	CLDN3	protein_coding	0.46	-2.2	0	0.48	-2.1	0	0.29	-3.4	0
ENSG00000189143	CLDN4	protein_coding	NS	NS	-	NS	NS	-	0.25	-4	0
ENSG00000181885	CLDN7	protein_coding	NS	NS	-	NS	NS	-	0.07	-14.2	0
ENSG00000110852	CLEC2B	protein_coding	2.65	2.7	0	2.52	2.5	0	NS	NS	-
ENSG00000069493	CLEC2D	protein_coding	2.99	3	0	3.26	3.3	0	NS	NS	-
ENSG00000213719	CLIC1	protein_coding	2.49	2.5	0	2.49	2.5	0	2.48	2.5	0
ENSG00000169504	CLIC4	protein_coding	2.02	2	0	NS	NS	-	3.08	3.1	0

ENSG00000105270	CLIP3	protein_coding	NS	NS	-	NS	NS	-	4.27	4.3	0
ENSG00000115295	CLIP4	protein_coding	2.08	2.1	0	2.03	2	0	NS	NS	-
ENSG00000113240	CLK4	protein_coding	NS	NS	-	NS	NS	-	0.29	-3.4	0
ENSG00000165959	CLMN	protein_coding	NS	NS	-	NS	NS	-	0.05	-19.3	0
ENSG00000137392	CLPS	protein_coding	0.11	-9.1	0	0.12	-8.1	0	0	-253.6	0
ENSG00000139182	CLSTN3	protein_coding	NS	NS	-	1.97	2	0	NS	NS	-
ENSG00000122705	CLTA	protein_coding	NS	NS	-	NS	NS	-	2.02	2	0
ENSG00000175416	CLTB	protein_coding	2.17	2.2	0	2.21	2.2	0	NS	NS	-
ENSG00000141367	CLTC	protein_coding	NS	NS	-	NS	NS	-	2.02	2	0
ENSG00000140931	CMTM3	protein_coding	3.23	3.2	0	3.2	3.2	0	3.48	3.5	0
ENSG00000091317	CMTM6	protein_coding	2.15	2.2	0	2.24	2.2	0	NS	NS	-
ENSG00000170293	CMTM8	protein_coding	0.45	-2.2	0	0.47	-2.1	0	0.23	-4.3	0
ENSG00000100528	CNIH	protein_coding	2.02	2	0	1.95	2	0	NS	NS	-
ENSG00000143771	CNIH4	protein_coding	NS	NS	-	NS	NS	-	3.47	3.5	0
ENSG00000142675	CNKSR1	protein_coding	NS	NS	-	NS	NS	-	0.27	-3.7	0
ENSG00000153721	CNKSR3	protein_coding	0.33	-3	0	0.33	-3	0	0.3	-3.3	0
ENSG00000064666	CNN2	protein_coding	3.25	3.3	0	2.91	2.9	0	6.03	6	0
ENSG00000166997	CNPY4	protein_coding	NS	NS	-	NS	NS	-	3.25	3.2	0
ENSG00000119865	CNRIP1	protein_coding	NS	NS	-	NS	NS	-	2.84	2.8	0
ENSG00000176563	CNTD1	protein_coding	0.36	-2.8	0	0.36	-2.8	0	NS	NS	-
ENSG00000108797	CNTNAP1	protein_coding	NS	NS	-	NS	NS	-	2.72	2.7	0
ENSG00000082438	COBLL1	protein_coding	0.45	-2.2	0	0.46	-2.2	0	NS	NS	-
ENSG00000100473	COCH	protein_coding	0.3	-3.4	0	0.3	-3.3	0	NS	NS	-
ENSG00000123500	COL10A1	protein_coding	6.67	6.7	0	7.28	7.3	0	NS	NS	-
ENSG00000111799	COL12A1	protein_coding	10.6	10.6	0	9.69	9.7	0	17.94	17.9	0
ENSG00000108821	COL1A1	protein_coding	9.29	9.3	0	9.37	9.4	0	8.6	8.6	0
ENSG00000164692	COL1A2	protein_coding	9.19	9.2	0	9.26	9.3	0	8.62	8.6	0
ENSG00000168542	COL3A1	protein_coding	8.28	8.3	0	8.87	8.9	0	NS	NS	-
ENSG00000187498	COL4A1	protein_coding	3.26	3.3	0	3.47	3.5	0	NS	NS	-
ENSG00000134871	COL4A2	protein_coding	2.81	2.8	0	2.97	3	0	NS	NS	-
ENSG00000130635	COL5A1	protein_coding	3.07	3.1	0	3.18	3.2	0	2.18	2.2	0
ENSG00000204262	COL5A2	protein_coding	7.15	7.2	0	7.37	7.4	0	5.4	5.4	0
ENSG00000142156	COL6A1	protein_coding	NS	NS	-	NS	NS	-	5.63	5.6	0
ENSG00000142173	COL6A2	protein_coding	NS	NS	-	NS	NS	-	3.33	3.3	0
ENSG00000163359	COL6A3	protein_coding	6.3	6.3	0	6.15	6.1	0	NS	NS	-
ENSG00000144810	COL8A1	protein_coding	6.62	6.6	0	6.89	6.9	0	4.47	4.5	0
ENSG00000158270	COLEC12	protein_coding	NS	NS	-	NS	NS	-	8.4	8.4	0
ENSG00000105664	COMP	protein_coding	6.23	6.2	0	6.77	6.8	0	NS	NS	-
ENSG00000198612	COPS8	protein_coding	NS	NS	-	NS	NS	-	2.66	2.7	0
ENSG00000005243	COPZ2	protein_coding	NS	NS	-	NS	NS	-	5.44	5.4	0
ENSG00000135469	COQ10A	protein_coding	0.5	-2	0	0.49	-2.1	0	NS	NS	-
ENSG00000145244	CORIN	protein_coding	3.6	3.6	0	3.45	3.4	0	NS	NS	-

ENSG00000110880	CORO1C	protein_coding	2.62	2.6	0	2.4	2.4	0	4.37	4.4	0
ENSG00000106789	CORO2A	protein_coding	4.03	4	0	4.42	4.4	0	NS	NS	-
ENSG00000103187	COTL1	protein_coding	2.15	2.1	0	2.15	2.1	0	NS	NS	-
ENSG00000176340	COX8A	protein_coding	NS	NS	-	NS	NS	-	3.2	3.2	0
ENSG00000047457	CP	protein_coding	NS	NS	-	18.86	18.9	0	NS	NS	-
ENSG00000091704	CPA1	protein_coding	0.15	-6.7	0	NS	NS	-	0	-253.9	0
ENSG00000158516	CPA2	protein_coding	0.13	-7.7	0	NS	NS	-	0	-534.4	0
ENSG00000128510	CPA4	protein_coding	NS	NS	-	0.48	-2.1	0	NS	NS	-
ENSG00000153002	CPB1	protein_coding	0.17	-6	0	NS	NS	-	0	-333.1	0
ENSG00000107864	CPEB3	protein_coding	NS	NS	-	NS	NS	-	0.39	-2.5	0
ENSG00000103381	CPPED1	protein_coding	2.55	2.5	0	2.29	2.3	0	NS	NS	-
ENSG00000021826	CPS1	protein_coding	NS	NS	-	NS	NS	-	2.58	2.6	0
ENSG00000121898	CPXM2	protein_coding	2.85	2.9	0	2.58	2.6	0	5.08	5.1	0
ENSG00000143320	CRABP2	protein_coding	NS	NS	-	NS	NS	-	13.46	13.5	0
ENSG00000095321	CRAT	protein_coding	NS	NS	-	0.47	-2.1	0	NS	NS	-
ENSG00000213676	CREBL1	protein_coding	NS	NS	-	NS	NS	-	2.18	2.2	0
ENSG00000106113	CRHR2	protein_coding	0.42	-2.4	0	0.41	-2.4	0	NS	NS	-
ENSG00000103196	CRISPLD2	protein_coding	2.18	2.2	0	2.2	2.2	0	NS	NS	-
ENSG00000176390	CRLF3	protein_coding	2.06	2.1	0	2.11	2.1	0	NS	NS	-
ENSG00000109846	CRYAB	protein_coding	NS	NS	-	NS	NS	-	8.07	8.1	0
ENSG00000172346	CSDC2	protein_coding	0.5	-2	0	0.48	-2.1	0	NS	NS	-
ENSG00000198223	CSF2RA	protein_coding	NS	NS	-	2.47	2.5	0	NS	NS	-
ENSG00000169826	CSGALNACT2	protein_coding	2.66	2.7	0	2.71	2.7	0	NS	NS	-
ENSG00000033100	CSGLCA-T	protein_coding	NS	NS	-	NS	NS	-	2.36	2.4	0
ENSG00000213218	CSH2	protein_coding	0.49	-2	0	0.49	-2	0	NS	NS	-
ENSG00000164796	CSMD3	protein_coding	0.23	-4.4	0	0.22	-4.4	0	NS	NS	-
ENSG00000173546	CSPG4	protein_coding	NS	NS	-	NS	NS	-	4.73	4.7	0
ENSG00000170373	CST1	protein_coding	20.79	20.8	0	23.29	23.3	0	NS	NS	-
ENSG00000170369	CST2	protein_coding	4.35	4.3	0	4.73	4.7	0	NS	NS	-
ENSG00000160213	CSTB	protein_coding	2.39	2.4	0	2.29	2.3	0	3.19	3.2	0
ENSG00000205644	CTD-2375G15.1	pseudogene	0.5	-2	0	0.5	-2	0	NS	NS	-
ENSG00000116761	CTH	protein_coding	0.19	-5.1	0	0.18	-5.5	0	NS	NS	-
ENSG00000164932	CTHRC1	protein_coding	2.29	2.3	0	2.33	2.3	0	NS	NS	-
ENSG00000169862	CTNND2	protein_coding	0.21	-4.8	0	0.22	-4.6	0	0.12	-8.2	0
ENSG00000168925	CTRB1	protein_coding	0.15	-6.6	0	0.17	-5.9	0	0.02	-62.4	0
ENSG00000168928	CTRB2	protein_coding	0.12	-8.1	0	0.14	-7.3	0	0.02	-61	0
ENSG00000162438	CTRC	protein_coding	0.08	-12.6	0	0.09	-11.4	0	0.01	-121.4	0
ENSG00000141086	CTRL	protein_coding	0.08	-12.6	0	0.09	-11.4	0	NS	NS	-
ENSG00000064601	CTSA	protein_coding	2.27	2.3	0	2.06	2.1	0	3.98	4	0
ENSG00000164733	CTSB	protein_coding	3.38	3.4	0	3.18	3.2	0	4.98	5	0
ENSG00000109861	CTSC	protein_coding	2.31	2.3	0	2.48	2.5	0	NS	NS	-
ENSG00000196188	CTSE	protein_coding	NS	NS	-	13.01	13	0	NS	NS	-

ENSG00000143387	CTSK	protein_coding	8.93	8.9	0	6.35	6.4	0	29.52	29.5	0
ENSG00000135047	CTSL1	protein_coding	NS	NS	-	NS	NS	-	6.15	6.2	0
ENSG00000163131	CTSS	protein_coding	NS	NS	-	3.8	3.8	0	NS	NS	-
ENSG00000085733	CTTN	protein_coding	2.51	2.5	0	2.47	2.5	0	2.87	2.9	0
ENSG00000077063	CTTNBP2	protein_coding	0.4	-2.5	0	0.43	-2.3	0	0.2	-4.9	0
ENSG00000143079	CTTNBP2NL	protein_coding	2.76	2.8	0	2.72	2.7	0	3.1	3.1	0
ENSG00000142544	CTU1	protein_coding	0.49	-2	0	0.48	-2.1	0	NS	NS	-
ENSG00000138161	CUZD1	protein_coding	0.1	-10.4	0	0.11	-9.3	0	0	-238.1	0
ENSG00000154639	CXADR	protein_coding	NS	NS	-	NS	NS	-	0.06	-16.8	0
ENSG00000169245	CXCL10	protein_coding	NS	NS	-	9.74	9.7	0	NS	NS	-
ENSG00000161921	CXCL16	protein_coding	NS	NS	-	3.28	3.3	0	NS	NS	-
ENSG00000189377	CXCL17	protein_coding	NS	NS	-	NS	NS	-	0.14	-7	0
ENSG00000121966	CXCR4	protein_coding	NS	NS	-	4.47	4.5	0	NS	NS	-
ENSG00000185753	CXorf38	protein_coding	1.96	2	0	2.06	2.1	0	NS	NS	-
ENSG00000166347	CYB5A	protein_coding	0.41	-2.4	0	0.39	-2.6	0	NS	NS	-
ENSG00000166394	CYB5R2	protein_coding	2.16	2.2	0	2.01	2	0	NS	NS	-
ENSG00000100243	CYB5R3	protein_coding	NS	NS	-	NS	NS	-	4.47	4.5	0
ENSG00000165168	CYBB	protein_coding	NS	NS	-	4.46	4.5	0	0.13	-7.6	0
ENSG00000138061	CYP1B1	protein_coding	6.38	6.4	0	5.93	5.9	0	9.98	10	0
ENSG00000135929	CYP27A1	protein_coding	2.47	2.5	0	NS	NS	-	2.85	2.8	0
ENSG00000213908	CYP2A7P1	pseudogene	NS	NS	-	0.51	-2	0	NS	NS	-
ENSG00000108242	CYP2C18	protein_coding	NS	NS	-	5.84	5.8	0	NS	NS	-
ENSG00000138115	CYP2C8	protein_coding	NS	NS	-	NS	NS	-	0.42	-2.4	0
ENSG00000130649	CYP2E1	protein_coding	0.46	-2.2	0	0.45	-2.2	0	NS	NS	-
ENSG00000146233	CYP39A1	protein_coding	0.39	-2.5	0	0.4	-2.5	0	NS	NS	-
ENSG00000021461	CYP3A43	protein_coding	0.45	-2.2	0	0.44	-2.3	0	NS	NS	-
ENSG00000106258	CYP3A5	protein_coding	NS	NS	-	NS	NS	-	0.1	-10.1	0
ENSG00000145476	CYP4V2	protein_coding	0.5	-2	0	0.51	-2	0	NS	NS	-
ENSG00000205795	CYS1	protein_coding	0.5	-2	0	0.5	-2	0	0.47	-2.1	0
ENSG00000008256	CYTH3	protein_coding	2.61	2.6	0	2.38	2.4	0	4.44	4.4	0
ENSG00000100055	CYTH4	protein_coding	NS	NS	-	2.16	2.2	0	NS	NS	-
ENSG00000128487	CYTSB	protein_coding	NS	NS	-	1.97	2	0	NS	NS	-
ENSG00000165617	DACT1	protein_coding	2.67	2.7	0	2.79	2.8	0	NS	NS	-
ENSG00000164535	DAGLB	protein_coding	NS	NS	-	NS	NS	-	2.28	2.3	0
ENSG00000070190	DAPP1	protein_coding	5.23	5.2	0	5.78	5.8	0	NS	NS	-
ENSG00000078725	DBC1	protein_coding	NS	NS	-	NS	NS	-	8.77	8.8	0
ENSG00000113758	DBN1	protein_coding	NS	NS	-	NS	NS	-	2.62	2.6	0
ENSG00000164465	DCBLD1	protein_coding	2.54	2.5	0	2.63	2.6	0	NS	NS	-
ENSG00000057019	DCBLD2	protein_coding	3.67	3.7	0	NS	NS	-	4.7	4.7	0
ENSG00000146038	DCDC2	protein_coding	0.2	-5.1	0	0.21	-4.7	0	0.07	-15.4	0
ENSG00000133083	DCLK1	protein_coding	NS	NS	-	NS	NS	-	5.01	5	0
ENSG00000118655	DCLRE1B	protein_coding	NS	NS	-	NS	NS	-	1.99	2	0

ENSG00000172795	DCP2	protein_coding	1.96	2	0	2.08	2.1	0	NS	NS	-
ENSG00000204843	DCTN1	protein_coding	NS	NS	-	NS	NS	-	2.3	2.3	0
ENSG00000213722	DDAH2	protein_coding	1.99	2	0	NS	NS	-	2.52	2.5	0
ENSG00000134574	DDB2	protein_coding	NS	NS	-	NS	NS	-	2.55	2.6	0
ENSG00000132437	DDC	protein_coding	0.4	-2.5	0	0.42	-2.4	0	NS	NS	-
ENSG00000162733	DDR2	protein_coding	2.7	2.7	0	2.2	2.2	0	NS	NS	-
ENSG00000137628	DDX60	protein_coding	NS	NS	-	4.2	4.2	0	NS	NS	-
ENSG00000181381	DDX60L	protein_coding	2.9	2.9	0	3.12	3.1	0	NS	NS	-
ENSG00000164825	DEFB1	protein_coding	NS	NS	-	NS	NS	-	0.06	-17.8	0
ENSG00000105339	DENND3	protein_coding	1.98	2	0	2.09	2.1	0	NS	NS	-
ENSG00000105928	DFNA5	protein_coding	2.91	2.9	0	NS	NS	-	5.82	5.8	0
ENSG00000237517	DGCR5	antisense	0.46	-2.2	0	0.46	-2.2	0	NS	NS	-
ENSG00000102780	DGKH	protein_coding	4	4	0	4.32	4.3	0	NS	NS	-
ENSG00000114956	DGUOK	protein_coding	NS	NS	-	NS	NS	-	2.44	2.4	0
ENSG00000116133	DHCR24	protein_coding	3.24	3.2	0	2.95	3	0	5.56	5.6	0
ENSG00000172893	DHCR7	protein_coding	NS	NS	-	NS	NS	-	5.51	5.5	0
ENSG00000228716	DHFR	protein_coding	2.85	2.9	0	2.91	2.9	0	NS	NS	-
ENSG00000147202	DIAPH2	protein_coding	1.95	2	0	2.03	2	0	NS	NS	-
ENSG00000139734	DIAPH3	protein_coding	NS	NS	-	NS	NS	-	2.64	2.6	0
ENSG00000162595	DIRAS3	protein_coding	0.47	-2.1	0	0.45	-2.2	0	NS	NS	-
ENSG00000107984	DKK1	protein_coding	16.71	16.7	0	11.87	11.9	0	55.49	55.5	0
ENSG00000155011	DKK2	protein_coding	NS	NS	-	3.1	3.1	0	24.23	24.2	0
ENSG00000050165	DKK3	protein_coding	3.43	3.4	0	3.1	3.1	0	6.06	6.1	0
ENSG00000126787	DLGAP5	protein_coding	3.75	3.7	0	3.97	4	0	NS	NS	-
ENSG00000185559	DLK1	protein_coding	0.29	-3.4	0	0.3	-3.4	0	NS	NS	-
ENSG00000137094	DNAJB5	protein_coding	NS	NS	-	NS	NS	-	2.74	2.7	0
ENSG00000108176	DNAJC12	protein_coding	0.51	-2	0	NS	NS	-	0.26	-3.8	0
ENSG00000138246	DNAJC13	protein_coding	2.07	2.1	0	1.98	2	0	2.75	2.7	0
ENSG00000213918	DNASE1	protein_coding	0.14	-7	0	0.15	-6.8	0	0.11	-9.4	0
ENSG00000130816	DNMT1	protein_coding	1.99	2	0	1.97	2	0	2.09	2.1	0
ENSG00000135905	DOCK10	protein_coding	3.48	3.5	0	3.54	3.5	0	NS	NS	-
ENSG00000147251	DOCK11	protein_coding	4.39	4.4	0	4.45	4.4	0	NS	NS	-
ENSG00000134516	DOCK2	protein_coding	NS	NS	-	4.12	4.1	0	NS	NS	-
ENSG00000147459	DOCK5	protein_coding	2.46	2.5	0	2.49	2.5	0	NS	NS	-
ENSG00000116641	DOCK7	protein_coding	NS	NS	-	NS	NS	-	2.26	2.3	0
ENSG00000107099	DOCK8	protein_coding	NS	NS	-	NS	NS	-	0.19	-5.4	0
ENSG00000101134	DOK5	protein_coding	3.7	3.7	0	3.33	3.3	0	6.61	6.6	0
ENSG00000166171	DPCD	protein_coding	NS	NS	-	NS	NS	-	2.19	2.2	0
ENSG00000015413	DPEP1	protein_coding	0.15	-6.6	0	0.17	-6	0	0.03	-29.4	0
ENSG00000175497	DPP10	protein_coding	0.13	-8	0	0.13	-7.9	0	NS	NS	-
ENSG00000197635	DPP4	protein_coding	NS	NS	-	NS	NS	-	15.49	15.5	0
ENSG00000173852	DPY19L1	protein_coding	2.51	2.5	0	2.57	2.6	0	NS	NS	-

ENSG00000113657	DPYSL3	protein_coding	5.2	5.2	0	5.33	5.3	0	4.13	4.1	0
ENSG00000136048	DRAM1	protein_coding	NS	NS	-	2.03	2	0	12.4	12.4	0
ENSG00000175550	DRAP1	protein_coding	NS	NS	-	NS	NS	-	2.29	2.3	0
ENSG00000134755	DSC2	protein_coding	NS	NS	-	NS	NS	-	0.1	-10.2	0
ENSG00000111817	DSE	protein_coding	4.21	4.2	0	3.82	3.8	0	7.27	7.3	0
ENSG00000171451	DSEL	protein_coding	NS	NS	-	NS	NS	-	9.45	9.4	0
ENSG00000046604	DSG2	protein_coding	NS	NS	-	NS	NS	-	0.07	-15	0
ENSG00000096696	DSP	protein_coding	NS	NS	-	NS	NS	-	0.15	-6.6	0
ENSG00000151914	DST	protein_coding	2.25	2.3	0	2.22	2.2	0	NS	NS	-
ENSG00000143476	DTL	protein_coding	3.44	3.4	0	3.39	3.4	0	NS	NS	-
ENSG00000134769	DTNA	protein_coding	0.23	-4.3	0	0.23	-4.3	0	0.24	-4.1	0
ENSG00000168393	DTYMK	protein_coding	NS	NS	-	NS	NS	-	2.55	2.6	0
ENSG00000175826	DULLARD	protein_coding	NS	NS	-	NS	NS	-	2.12	2.1	0
ENSG00000140279	DUOX2	protein_coding	NS	NS	-	13.27	13.3	0	NS	NS	-
ENSG00000161326	DUSP14	protein_coding	NS	NS	-	NS	NS	-	8.99	9	0
ENSG00000111266	DUSP16	protein_coding	NS	NS	-	NS	NS	-	0.33	-3.1	0
ENSG00000167065	DUSP18	protein_coding	2.53	2.5	0	2.52	2.5	0	2.67	2.7	0
ENSG00000141627	DYM	protein_coding	NS	NS	-	0.5	-2	0	NS	NS	-
ENSG00000088986	DYNLL1	protein_coding	NS	NS	-	NS	NS	-	2.6	2.6	0
ENSG00000125971	DYNLRB1	protein_coding	NS	NS	-	NS	NS	-	2.77	2.8	0
ENSG00000146425	DYNLT1	protein_coding	2.87	2.9	0	2.8	2.8	0	3.39	3.4	0
ENSG00000165169	DYNLT3	protein_coding	2.22	2.2	0	2.16	2.2	0	NS	NS	-
ENSG00000143479	DYRK3	protein_coding	NS	NS	-	NS	NS	-	4.19	4.2	0
ENSG00000165891	E2F7	protein_coding	NS	NS	-	NS	NS	-	2.38	2.4	0
ENSG00000147155	EBP	protein_coding	NS	NS	-	NS	NS	-	2.3	2.3	0
ENSG00000145194	ECE2	protein_coding	0.5	-2	0	0.48	-2.1	0	NS	NS	-
ENSG00000134463	ECHDC3	protein_coding	0.39	-2.6	0	0.38	-2.6	0	NS	NS	-
ENSG00000143369	ECM1	protein_coding	NS	NS	-	NS	NS	-	11.62	11.6	0
ENSG00000114346	ECT2	protein_coding	5.53	5.5	0	5.7	5.7	0	4.12	4.1	0
ENSG00000164176	EDIL3	protein_coding	6.13	6.1	0	6.06	6.1	0	NS	NS	-
ENSG00000151617	EDNRA	protein_coding	7.5	7.5	0	8.16	8.2	0	NS	NS	-
ENSG00000102189	EEA1	protein_coding	NS	NS	-	NS	NS	-	2.95	3	0
ENSG00000177685	EFCAB4A	protein_coding	0.45	-2.2	0	0.46	-2.2	0	NS	NS	-
ENSG00000172638	EFEMP2	protein_coding	NS	NS	-	2.03	2	0	6.68	6.7	0
ENSG00000184349	EFNA5	protein_coding	5.9	5.9	0	6	6	0	5.09	5.1	0
ENSG00000125266	EFNB2	protein_coding	3.9	3.9	0	4.15	4.2	0	NS	NS	-
ENSG00000100842	EFS	protein_coding	NS	NS	-	NS	NS	-	2.12	2.1	0
ENSG00000138798	EGF	protein_coding	0.06	-16.3	0	0.06	-15.6	0	0.04	-25.6	0
ENSG00000129521	EGLN3	protein_coding	NS	NS	-	5.37	5.4	0	NS	NS	-
ENSG00000110047	EHD1	protein_coding	NS	NS	-	NS	NS	-	2.78	2.8	0
ENSG00000024422	EHD2	protein_coding	NS	NS	-	NS	NS	-	5.85	5.8	0
ENSG00000013016	EHD3	protein_coding	NS	NS	-	NS	NS	-	3.67	3.7	0

ENSG00000135373	EHF	protein_coding	NS	NS	-	NS	NS	-	0.03	-32.9	0
ENSG00000114784	EIF1B	protein_coding	NS	NS	-	NS	NS	-	2.15	2.1	0
ENSG00000172071	EIF2AK3	protein_coding	0.38	-2.6	0	0.39	-2.6	0	NS	NS	-
ENSG00000128829	EIF2AK4	protein_coding	NS	NS	-	NS	NS	-	2.6	2.6	0
ENSG00000156976	EIF4A2	protein_coding	NS	NS	-	NS	NS	-	0.35	-2.9	0
ENSG00000141543	EIF4A3	protein_coding	NS	NS	-	NS	NS	-	2.46	2.5	0
ENSG00000187840	EIF4EBP1	protein_coding	0.48	-2.1	0	0.46	-2.2	0	NS	NS	-
ENSG00000075151	EIF4G3	protein_coding	1.98	2	0	NS	NS	-	2.5	2.5	0
ENSG00000163435	ELF3	protein_coding	NS	NS	-	NS	NS	-	0.1	-9.6	0
ENSG00000102034	ELF4	protein_coding	3.41	3.4	0	3.34	3.3	0	3.93	3.9	0
ENSG00000111145	ELK3	protein_coding	2.76	2.8	0	2.59	2.6	0	4.14	4.1	0
ENSG00000066322	ELOVL1	protein_coding	2.17	2.2	0	2	2	0	NS	NS	-
ENSG00000197977	ELOVL2	protein_coding	0.36	-2.8	0	0.37	-2.7	0	0.31	-3.2	0
ENSG00000012660	ELOVL5	protein_coding	2.45	2.5	0	2.35	2.3	0	NS	NS	-
ENSG00000170522	ELOVL6	protein_coding	NS	NS	-	NS	NS	-	3.14	3.1	0
ENSG00000164181	ELOVL7	protein_coding	NS	NS	-	NS	NS	-	0.12	-8.1	0
ENSG00000170571	EMB	protein_coding	3.22	3.2	0	3.47	3.5	0	NS	NS	-
ENSG00000132205	EMILIN2	protein_coding	NS	NS	-	NS	NS	-	3.7	3.7	0
ENSG00000165521	EML5	protein_coding	NS	NS	-	NS	NS	-	0.47	-2.1	0
ENSG00000213853	EMP2	protein_coding	2.38	2.4	0	2.41	2.4	0	NS	NS	-
ENSG00000142227	EMP3	protein_coding	3.41	3.4	0	2.54	2.5	0	10.36	10.4	0
ENSG00000170370	EMX2	protein_coding	NS	NS	-	NS	NS	-	4.76	4.8	0
ENSG00000132464	ENAM	protein_coding	0.46	-2.2	0	0.48	-2.1	0	0.3	-3.4	0
ENSG00000171617	ENC1	protein_coding	NS	NS	-	2.04	2	0	NS	NS	-
ENSG00000149218	ENDOD1	protein_coding	2.59	2.6	0	2.66	2.7	0	NS	NS	-
ENSG00000106991	ENG	protein_coding	2.15	2.2	0	NS	NS	-	3.02	3	0
ENSG00000074800	ENO1	protein_coding	2.77	2.8	0	2.74	2.7	0	NS	NS	-
ENSG00000136960	ENPP2	protein_coding	NS	NS	-	NS	NS	-	11.01	11	0
ENSG00000138185	ENTPD1	protein_coding	NS	NS	-	3.86	3.9	0	NS	NS	-
ENSG00000168032	ENTPD3	protein_coding	0.29	-3.4	0	0.31	-3.2	0	NS	NS	-
ENSG00000095203	EPB41L4B	protein_coding	0.22	-4.5	0	0.24	-4.3	0	0.13	-7.8	0
ENSG00000115109	EPB41L5	protein_coding	0.47	-2.1	0	0.5	-2	0	0.28	-3.6	0
ENSG00000158856	EPB49	protein_coding	NS	NS	-	NS	NS	-	0.43	-2.3	0
ENSG00000119888	EPCAM	protein_coding	NS	NS	-	NS	NS	-	0.02	-60.8	0
ENSG00000044524	EPHA3	protein_coding	NS	NS	-	3.38	3.4	0	NS	NS	-
ENSG00000116106	EPHA4	protein_coding	3.41	3.4	0	3.6	3.6	0	1.96	2	0
ENSG00000143819	EPHX1	protein_coding	0.32	-3.1	0	0.29	-3.4	0	NS	NS	-
ENSG00000120915	EPHX2	protein_coding	0.24	-4.2	0	0.25	-4	0	0.15	-6.9	0
ENSG00000151491	EPS8	protein_coding	2.36	2.4	0	2.4	2.4	0	NS	NS	-
ENSG00000133106	EPSTI1	protein_coding	3.95	3.9	0	4.31	4.3	0	NS	NS	-
ENSG00000164307	ERAP1	protein_coding	2.12	2.1	0	2.25	2.2	0	NS	NS	-
ENSG00000065361	ERBB3	protein_coding	NS	NS	-	NS	NS	-	0.2	-5	0

ENSG00000178568	ERBB4	protein_coding	0.38	-2.7	0	0.38	-2.6	0	0.33	-3	0
ENSG00000178607	ERN1	protein_coding	0.3	-3.4	0	0.3	-3.3	0	NS	NS	-
ENSG00000197930	ERO1L	protein_coding	2.66	2.7	0	2.82	2.8	0	NS	NS	-
ENSG00000086619	ERO1LB	protein_coding	0.11	-8.9	0	0.12	-8.1	0	0.03	-31.9	0
ENSG00000139055	ERP27	protein_coding	0.07	-15	0	0.07	-13.5	0	0.01	-166.5	0
ENSG00000116285	ERRFI1	protein_coding	0.34	-2.9	0	0.37	-2.7	0	NS	NS	-
ENSG00000104413	ESRP1	protein_coding	NS	NS	-	NS	NS	-	0.04	-22.8	0
ENSG00000103067	ESRP2	protein_coding	NS	NS	-	NS	NS	-	0.19	-5.2	0
ENSG00000196482	ESRRG	protein_coding	0.23	-4.3	0	0.24	-4.1	0	0.16	-6.1	0
ENSG00000120705	ETF1	protein_coding	NS	NS	-	NS	NS	-	2.09	2.1	0
ENSG00000105755	ETHE1	protein_coding	NS	NS	-	NS	NS	-	2.91	2.9	0
ENSG00000139163	ETNK1	protein_coding	NS	NS	-	2.14	2.1	0	NS	NS	-
ENSG00000134954	ETS1	protein_coding	2.17	2.2	0	2.18	2.2	0	NS	NS	-
ENSG00000157557	ETS2	protein_coding	NS	NS	-	2.02	2	0	NS	NS	-
ENSG00000006468	ETV1	protein_coding	3.4	3.4	0	3.52	3.5	0	NS	NS	-
ENSG00000126860	EVI2A	protein_coding	2.54	2.5	0	2.54	2.5	0	NS	NS	-
ENSG00000185862	EVI2B	protein_coding	2.57	2.6	0	2.7	2.7	0	NS	NS	-
ENSG00000070367	EXOC5	protein_coding	NS	NS	-	NS	NS	-	2	2	0
ENSG00000138190	EXOC6	protein_coding	NS	NS	-	NS	NS	-	0.19	-5.2	0
ENSG00000182197	EXT1	protein_coding	NS	NS	-	NS	NS	-	4.08	4.1	0
ENSG00000012232	EXTL3	protein_coding	NS	NS	-	NS	NS	-	2.46	2.5	0
ENSG00000104313	EYA1	protein_coding	NS	NS	-	NS	NS	-	4.37	4.4	0
ENSG00000088926	F11	protein_coding	0.09	-11.3	0	0.1	-10.5	0	0.03	-29.2	0
ENSG00000158769	F11R	protein_coding	NS	NS	-	NS	NS	-	0.09	-11.3	0
ENSG00000124491	F13A1	protein_coding	NS	NS	-	NS	NS	-	0.1	-10.4	0
ENSG00000181104	F2R	protein_coding	6.13	6.1	0	6.57	6.6	0	NS	NS	-
ENSG00000164220	F2RL2	protein_coding	8.39	8.4	0	8.88	8.9	0	4.46	4.5	0
ENSG00000185010	F8	protein_coding	0.11	-8.7	0	0.11	-8.8	0	NS	NS	-
ENSG00000117480	FAAH	protein_coding	NS	NS	-	NS	NS	-	0.46	-2.2	0
ENSG00000121769	FABP3	protein_coding	NS	NS	-	4.42	4.4	0	46.36	46.4	0
ENSG00000149485	FADS1	protein_coding	NS	NS	-	NS	NS	-	10.43	10.4	0
ENSG00000134824	FADS2	protein_coding	4.52	4.5	0	3.1	3.1	0	15.87	15.9	0
ENSG00000221968	FADS3	protein_coding	NS	NS	-	NS	NS	-	3.49	3.5	0
ENSG00000162636	FAM102B	protein_coding	2.72	2.7	0	2.88	2.9	0	NS	NS	-
ENSG00000145569	FAM105A	protein_coding	NS	NS	-	NS	NS	-	0.08	-12	0
ENSG00000168309	FAM107A	protein_coding	0.44	-2.3	0	0.44	-2.3	0	NS	NS	-
ENSG00000129968	FAM108A1	protein_coding	0.5	-2	0	0.5	-2	0	NS	NS	-
ENSG00000169122	FAM110B	protein_coding	NS	NS	-	NS	NS	-	2.87	2.9	0
ENSG00000184731	FAM110C	protein_coding	0.42	-2.4	0	0.43	-2.3	0	0.35	-2.9	0
ENSG00000189057	FAM111B	protein_coding	3.33	3.3	0	3.48	3.5	0	NS	NS	-
ENSG00000197712	FAM114A1	protein_coding	2.15	2.1	0	2.03	2	0	3.09	3.1	0
ENSG00000197798	FAM118B	protein_coding	2.08	2.1	0	1.98	2	0	NS	NS	-

ENSG00000134590	FAM127A	protein_coding	2.16	2.2	0	NS	NS	-	4.83	4.8	0
ENSG00000203950	FAM127B	protein_coding	NS	NS	-	NS	NS	-	3.14	3.1	0
ENSG00000135842	FAM129A	protein_coding	0.28	-3.6	0	0.27	-3.7	0	NS	NS	-
ENSG00000109794	FAM149A	protein_coding	0.49	-2	0	0.51	-2	0	0.4	-2.5	0
ENSG00000189292	FAM150B	protein_coding	0.31	-3.3	0	0.32	-3.1	0	0.22	-4.5	0
ENSG00000164142	FAM160A1	protein_coding	0.3	-3.3	0	0.33	-3	0	0.06	-17	0
ENSG00000104427	FAM164A	protein_coding	2.06	2.1	0	2.03	2	0	2.32	2.3	0
ENSG00000144369	FAM171B	protein_coding	NS	NS	-	NS	NS	-	3.88	3.9	0
ENSG00000185442	FAM174B	protein_coding	0.35	-2.8	0	0.37	-2.7	0	0.25	-4	0
ENSG00000189320	FAM180A	protein_coding	NS	NS	-	NS	NS	-	13.21	13.2	0
ENSG00000222011	FAM185A	protein_coding	NS	NS	-	NS	NS	-	0.48	-2.1	0
ENSG00000184305	FAM190A	protein_coding	NS	NS	-	NS	NS	-	0.26	-3.9	0
ENSG00000164125	FAM198B	protein_coding	3.41	3.4	0	3.43	3.4	0	NS	NS	-
ENSG00000108950	FAM20A	protein_coding	2.92	2.9	0	2.51	2.5	0	NS	NS	-
ENSG00000178033	FAM26E	protein_coding	3.78	3.8	0	3.36	3.4	0	7.14	7.1	0
ENSG00000154864	FAM38B	protein_coding	3.28	3.3	0	3.3	3.3	0	NS	NS	-
ENSG00000183844	FAM3B	protein_coding	NS	NS	-	NS	NS	-	0.02	-47.8	0
ENSG00000119979	FAM45A	protein_coding	NS	NS	-	NS	NS	-	5.17	5.2	0
ENSG00000183508	FAM46C	protein_coding	0.34	-3	0	0.37	-2.7	0	0.08	-13	0
ENSG00000189157	FAM47E	protein_coding	0.39	-2.6	0	0.41	-2.5	0	0.26	-3.9	0
ENSG00000153310	FAM49B	protein_coding	2.39	2.4	0	2.57	2.6	0	NS	NS	-
ENSG00000071859	FAM50A	protein_coding	NS	NS	-	NS	NS	-	2.17	2.2	0
ENSG00000144815	FAM55C	protein_coding	NS	NS	-	NS	NS	-	3.36	3.4	0
ENSG00000167695	FAM57A	protein_coding	NS	NS	-	NS	NS	-	3.42	3.4	0
ENSG00000128923	FAM63B	protein_coding	2.35	2.3	0	2.4	2.4	0	NS	NS	-
ENSG00000039523	FAM65A	protein_coding	NS	NS	-	NS	NS	-	2.83	2.8	0
ENSG00000154511	FAM69A	protein_coding	4.03	4	0	3.19	3.2	0	10.7	10.7	0
ENSG00000133477	FAM83F	protein_coding	NS	NS	-	NS	NS	-	0.5	-2	0
ENSG00000166595	FAM96B	protein_coding	NS	NS	-	NS	NS	-	2.3	2.3	0
ENSG00000144554	FANCD2	protein_coding	2.24	2.2	0	2.38	2.4	0	NS	NS	-
ENSG00000140525	FANCI	protein_coding	2.71	2.7	0	2.8	2.8	0	NS	NS	-
ENSG00000115392	FANCL	protein_coding	NS	NS	-	NS	NS	-	0.31	-3.2	0
ENSG00000078098	FAP	protein_coding	8.98	9	0	7.65	7.6	0	NS	NS	-
ENSG00000064763	FAR2	protein_coding	4.8	4.8	0	4.94	4.9	0	3.71	3.7	0
ENSG00000026103	FAS	protein_coding	3.15	3.1	0	2.91	2.9	0	5.08	5.1	0
ENSG00000169710	FASN	protein_coding	NS	NS	-	NS	NS	-	3.72	3.7	0
ENSG00000083857	FAT1	protein_coding	2.68	2.7	0	2.64	2.6	0	3.06	3.1	0
ENSG00000196159	FAT4	protein_coding	2.65	2.6	0	2.25	2.3	0	5.81	5.8	0
ENSG00000077942	FBLN1	protein_coding	NS	NS	-	NS	NS	-	5.87	5.9	0
ENSG00000163520	FBLN2	protein_coding	NS	NS	-	NS	NS	-	8.22	8.2	0
ENSG00000140092	FBLN5	protein_coding	NS	NS	-	NS	NS	-	3.54	3.5	0
ENSG00000166147	FBN1	protein_coding	9.15	9.2	0	8.57	8.6	0	13.75	13.8	0

ENSG00000138829	FBN2	protein_coding	NS	NS	-	NS	NS	-	15.97	16	0
ENSG00000165140	FBP1	protein_coding	NS	NS	-	NS	NS	-	0.16	-6.3	0
ENSG00000183580	FBXL7	protein_coding	NS	NS	-	NS	NS	-	5.39	5.4	0
ENSG00000214050	FBXO16	protein_coding	0.44	-2.3	0	0.46	-2.2	0	NS	NS	-
ENSG00000116661	FBXO2	protein_coding	0.48	-2.1	0	0.49	-2	0	NS	NS	-
ENSG00000156804	FBXO32	protein_coding	6.41	6.4	0	5.96	6	0	10.04	10	0
ENSG00000178974	FBXO34	protein_coding	1.98	2	0	2.11	2.1	0	NS	NS	-
ENSG00000164049	FBXW12	protein_coding	0.27	-3.8	0	0.27	-3.8	0	NS	NS	-
ENSG00000158869	FCER1G	protein_coding	NS	NS	-	4.03	4	0	NS	NS	-
ENSG00000119616	FCF1	protein_coding	2.07	2.1	0	2.02	2	0	NS	NS	-
ENSG00000143226	FCGR2A	protein_coding	NS	NS	-	3.15	3.1	0	NS	NS	-
ENSG00000072694	FCGR2B	protein_coding	NS	NS	-	3.11	3.1	0	NS	NS	-
ENSG00000066926	FECH	protein_coding	NS	NS	-	NS	NS	-	2.25	2.2	0
ENSG00000101311	FERMT1	protein_coding	NS	NS	-	6.73	6.7	0	NS	NS	-
ENSG00000149781	FERMT3	protein_coding	NS	NS	-	2.17	2.2	0	NS	NS	-
ENSG00000149557	FEZ1	protein_coding	NS	NS	-	NS	NS	-	6.15	6.2	0
ENSG00000102302	FGD1	protein_coding	NS	NS	-	NS	NS	-	2.37	2.4	0
ENSG00000139132	FGD4	protein_coding	NS	NS	-	NS	NS	-	0.29	-3.4	0
ENSG00000154783	FGD5	protein_coding	NS	NS	-	2.26	2.3	0	NS	NS	-
ENSG00000180263	FGD6	protein_coding	NS	NS	-	2.83	2.8	0	NS	NS	-
ENSG00000113578	FGF1	protein_coding	NS	NS	-	NS	NS	-	5.2	5.2	0
ENSG00000138675	FGF5	protein_coding	NS	NS	-	NS	NS	-	13.9	13.9	0
ENSG00000140285	FGF7	protein_coding	NS	NS	-	NS	NS	-	13.53	13.5	0
ENSG00000185390	FGF7P2	pseudogene	NS	NS	-	NS	NS	-	4.41	4.4	0
ENSG00000077782	FGFR1	protein_coding	NS	NS	-	0.46	-2.2	0	NS	NS	-
ENSG00000066468	FGFR2	protein_coding	NS	NS	-	NS	NS	-	0.15	-6.6	0
ENSG00000068078	FGFR3	protein_coding	0.31	-3.2	0	0.32	-3.1	0	NS	NS	-
ENSG00000127418	FGFRL1	protein_coding	NS	NS	-	0.5	-2	0	NS	NS	-
ENSG00000104760	FGL1	protein_coding	0.07	-14.2	0	0.08	-13.1	0	NS	NS	-
ENSG00000115641	FHL2	protein_coding	3.5	3.5	0	3.46	3.5	0	3.83	3.8	0
ENSG00000183386	FHL3	protein_coding	NS	NS	-	NS	NS	-	2.06	2.1	0
ENSG00000172500	FIBP	protein_coding	NS	NS	-	NS	NS	-	2.56	2.6	0
ENSG00000198855	FICD	protein_coding	0.43	-2.3	0	0.4	-2.5	0	NS	NS	-
ENSG00000141756	FKBP10	protein_coding	3.01	3	0	2.7	2.7	0	5.5	5.5	0
ENSG00000134285	FKBP11	protein_coding	0.32	-3.1	0	0.32	-3.2	0	NS	NS	-
ENSG00000106080	FKBP14	protein_coding	NS	NS	-	NS	NS	-	3.46	3.5	0
ENSG00000088832	FKBP1A	protein_coding	NS	NS	-	NS	NS	-	2.15	2.2	0
ENSG00000096060	FKBP5	protein_coding	NS	NS	-	NS	NS	-	0.07	-14.4	0
ENSG00000196924	FLNA	protein_coding	3.67	3.7	0	3.41	3.4	0	NS	NS	-
ENSG00000128591	FLNC	protein_coding	NS	NS	-	NS	NS	-	6.7	6.7	0
ENSG00000161791	FMNL3	protein_coding	3.29	3.3	0	3.51	3.5	0	NS	NS	-
ENSG00000122176	FMOD	protein_coding	2.17	2.2	0	2.06	2.1	0	NS	NS	-

ENSG00000115414	FN1	protein_coding	12.95	12.9	0	12.46	12.5	0	16.83	16.8	0
ENSG00000167363	FN3K	protein_coding	NS	NS	-	0.51	-2	0	NS	NS	-
ENSG00000187239	FNBP1	protein_coding	2.23	2.2	0	2.21	2.2	0	NS	NS	-
ENSG00000164694	FNDC1	protein_coding	6.08	6.1	0	6.4	6.4	0	NS	NS	-
ENSG00000175592	FOSL1	protein_coding	NS	NS	-	NS	NS	-	4.07	4.1	0
ENSG00000125798	FOXA2	protein_coding	0.46	-2.2	0	0.48	-2.1	0	0.37	-2.7	0
ENSG00000111206	FOXM1	protein_coding	NS	NS	-	NS	NS	-	3.41	3.4	0
ENSG00000128573	FOXP2	protein_coding	NS	NS	-	0.45	-2.2	0	NS	NS	-
ENSG00000164946	FREM1	protein_coding	NS	NS	-	NS	NS	-	0.2	-5	0
ENSG00000107130	FREQ	protein_coding	NS	NS	-	NS	NS	-	5.03	5	0
ENSG00000225899	FRG2B	protein_coding	0.43	-2.3	0	0.45	-2.2	0	NS	NS	-
ENSG00000111816	FRK	protein_coding	NS	NS	-	NS	NS	-	0.3	-3.3	0
ENSG00000139926	FRMD6	protein_coding	4.35	4.4	0	3.26	3.3	0	13.07	13.1	0
ENSG00000156869	FRRS1	protein_coding	3.34	3.3	0	3.37	3.4	0	NS	NS	-
ENSG00000075618	FSCN1	protein_coding	2.64	2.6	0	2.25	2.3	0	5.75	5.7	0
ENSG00000134363	FST	protein_coding	NS	NS	-	NS	NS	-	15.46	15.5	0
ENSG00000163430	FSTL1	protein_coding	4.29	4.3	0	4.02	4	0	6.46	6.5	0
ENSG00000165775	FUNDC2	protein_coding	NS	NS	-	0.49	-2	0	NS	NS	-
ENSG00000174951	FUT1	protein_coding	0.44	-2.3	0	0.44	-2.3	0	NS	NS	-
ENSG00000196968	FUT11	protein_coding	2.34	2.3	0	2.27	2.3	0	2.93	2.9	0
ENSG00000176920	FUT2	protein_coding	4.18	4.2	0	4.55	4.6	0	NS	NS	-
ENSG00000137731	FXYD2	protein_coding	0.33	-3	0	NS	NS	-	0.13	-7.7	0
ENSG00000089356	FXYD3	protein_coding	NS	NS	-	4.57	4.6	0	NS	NS	-
ENSG00000089327	FXYD5	protein_coding	3.06	3.1	0	2.93	2.9	0	NS	NS	-
ENSG00000137726	FXYD6	protein_coding	NS	NS	-	NS	NS	-	0.27	-3.7	0
ENSG00000082074	FYB	protein_coding	NS	NS	-	4.95	4.9	0	NS	NS	-
ENSG00000010810	FYN	protein_coding	2.08	2.1	0	2.05	2	0	NS	NS	-
ENSG00000163251	FZD5	protein_coding	NS	NS	-	NS	NS	-	0.27	-3.7	0
ENSG00000160211	G6PD	protein_coding	NS	NS	-	NS	NS	-	5.11	5.1	0
ENSG00000033327	GAB2	protein_coding	NS	NS	-	NS	NS	-	2.01	2	0
ENSG00000136928	GABBR2	protein_coding	NS	NS	-	NS	NS	-	2.38	2.4	0
ENSG00000116717	GADD45A	protein_coding	2.29	2.3	0	2.13	2.1	0	3.52	3.5	0
ENSG00000197093	GAL3ST4	protein_coding	NS	NS	-	NS	NS	-	2.35	2.3	0
ENSG00000054983	GALC	protein_coding	2.05	2	0	2.18	2.2	0	NS	NS	-
ENSG00000141012	GALNS	protein_coding	NS	NS	-	NS	NS	-	2.14	2.1	0
ENSG00000164574	GALNT10	protein_coding	2.46	2.5	0	2.46	2.5	0	NS	NS	-
ENSG00000115339	GALNT3	protein_coding	NS	NS	-	NS	NS	-	0.12	-8.6	0
ENSG00000136542	GALNT5	protein_coding	11.72	11.7	0	11.47	11.5	0	13.7	13.7	0
ENSG00000131386	GALNTL2	protein_coding	NS	NS	-	NS	NS	-	4.26	4.3	0
ENSG00000130005	GAMT	protein_coding	0.3	-3.3	0	0.28	-3.5	0	NS	NS	-
ENSG00000127688	GAN	protein_coding	NS	NS	-	1.96	2	0	NS	NS	-
ENSG00000111640	GAPDH	protein_coding	2.29	2.3	0	2.22	2.2	0	2.84	2.8	0

ENSG00000148935	GAS2	protein_coding	0.2	-5.1	0	0.21	-4.8	0	NS	NS	-
ENSG00000139354	GAS2L3	protein_coding	1.96	2	0	1.96	2	0	NS	NS	-
ENSG00000136574	GATA4	protein_coding	0.36	-2.8	0	0.36	-2.7	0	NS	NS	-
ENSG00000141448	GATA6	protein_coding	NS	NS	-	NS	NS	-	0.46	-2.2	0
ENSG00000171766	GATM	protein_coding	0.18	-5.6	0	0.2	-5	0	0.01	-181.4	0
ENSG00000160844	GATS	protein_coding	0.44	-2.3	0	0.46	-2.2	0	NS	NS	-
ENSG00000177628	GBA	protein_coding	NS	NS	-	NS	NS	-	5.91	5.9	0
ENSG00000160766	GBAP	pseudogene	NS	NS	-	NS	NS	-	4.04	4	0
ENSG00000114480	GBE1	protein_coding	NS	NS	-	NS	NS	-	5.54	5.5	0
ENSG00000117228	GBP1	protein_coding	3.79	3.8	0	3.83	3.8	0	NS	NS	-
ENSG00000162645	GBP2	protein_coding	5.53	5.5	0	5.95	6	0	NS	NS	-
ENSG00000117226	GBP3	protein_coding	3.8	3.8	0	4	4	0	NS	NS	-
ENSG00000162654	GBP4	protein_coding	NS	NS	-	4.46	4.5	0	NS	NS	-
ENSG00000145321	GC	protein_coding	NS	NS	-	NS	NS	-	0.05	-20.1	0
ENSG00000115271	GCA	protein_coding	NS	NS	-	NS	NS	-	0.15	-6.7	0
ENSG00000100116	GCAT	protein_coding	0.36	-2.8	0	0.35	-2.9	0	NS	NS	-
ENSG00000115263	GCG	protein_coding	NS	NS	-	NS	NS	-	0.01	-146.6	0
ENSG00000187210	GCNT1	protein_coding	2.84	2.8	0	2.91	2.9	0	NS	NS	-
ENSG00000176928	GCNT4	protein_coding	0.41	-2.4	0	0.37	-2.7	0	NS	NS	-
ENSG00000104381	GDAP1	protein_coding	0.45	-2.2	0	0.47	-2.2	0	0.29	-3.4	0
ENSG00000107623	GDF10	protein_coding	0.5	-2	0	0.48	-2.1	0	NS	NS	-
ENSG00000125965	GDF5	protein_coding	NS	NS	-	NS	NS	-	2.72	2.7	0
ENSG00000102886	GDPD3	protein_coding	2.74	2.7	0	2.93	2.9	0	NS	NS	-
ENSG00000164949	GEM	protein_coding	3.82	3.8	0	3.9	3.9	0	NS	NS	-
ENSG00000152147	GEMIN6	protein_coding	NS	NS	-	NS	NS	-	2.49	2.5	0
ENSG00000142252	GEMIN7	protein_coding	NS	NS	-	NS	NS	-	1.99	2	0
ENSG00000103365	GGA2	protein_coding	NS	NS	-	NS	NS	-	0.47	-2.1	0
ENSG00000099998	GGT5	protein_coding	NS	NS	-	2.14	2.1	0	NS	NS	-
ENSG00000167741	GGT6	protein_coding	0.43	-2.3	0	0.44	-2.3	0	NS	NS	-
ENSG00000106560	GIMAP2	protein_coding	NS	NS	-	2.7	2.7	0	NS	NS	-
ENSG00000101003	GINS1	protein_coding	NS	NS	-	NS	NS	-	2.1	2.1	0
ENSG00000137960	GIPC2	protein_coding	NS	NS	-	NS	NS	-	0.14	-7.2	0
ENSG00000152661	GJA1	protein_coding	NS	NS	-	NS	NS	-	8.39	8.4	0
ENSG00000165474	GJB2	protein_coding	3.86	3.9	0	4.19	4.2	0	NS	NS	-
ENSG00000182963	GJC1	protein_coding	2.56	2.6	0	2.28	2.3	0	4.85	4.9	0
ENSG00000176402	GJC3	protein_coding	0.43	-2.3	0	0.43	-2.3	0	NS	NS	-
ENSG00000165113	GKAP1	protein_coding	0.5	-2	0	0.51	-2	0	NS	NS	-
ENSG00000149328	GLB1L2	protein_coding	NS	NS	-	NS	NS	-	0.32	-3.1	0
ENSG00000138604	GLCE	protein_coding	1.97	2	0	2.02	2	0	NS	NS	-
ENSG00000074047	GLI2	protein_coding	2.46	2.5	0	2.45	2.5	0	NS	NS	-
ENSG00000106571	GLI3	protein_coding	3.85	3.8	0	2.95	3	0	11.01	11	0
ENSG00000139278	GLIPR1	protein_coding	3.31	3.3	0	2.97	3	0	NS	NS	-

ENSG00000122694	GLIPR2	protein_coding	2.68	2.7	0	NS	NS	-	5.04	5	0
ENSG00000112164	GLP1R	protein_coding	0.39	-2.6	0	0.39	-2.6	0	0.35	-2.8	0
ENSG00000173221	GLRX	protein_coding	3.94	3.9	0	3.79	3.8	0	NS	NS	-
ENSG00000118990	GLRXP3	pseudogene	NS	NS	-	NS	NS	-	1.99	2	0
ENSG00000135423	GLS2	protein_coding	0.26	-3.9	0	0.26	-3.9	0	0.25	-4	0
ENSG00000130309	GLT25D1	protein_coding	2.6	2.6	0	2.44	2.4	0	3.85	3.8	0
ENSG00000120820	GLT8D2	protein_coding	3.12	3.1	0	NS	NS	-	6.09	6.1	0
ENSG00000112312	GMNN	protein_coding	0.21	-4.7	0	0.21	-4.7	0	NS	NS	-
ENSG00000137198	GMPR	protein_coding	0.38	-2.7	0	0.33	-3	0	NS	NS	-
ENSG00000120063	GNA13	protein_coding	1.99	2	0	2.04	2	0	NS	NS	-
ENSG00000060558	GNA15	protein_coding	NS	NS	-	2.78	2.8	0	NS	NS	-
ENSG00000114353	GNAI2	protein_coding	NS	NS	-	NS	NS	-	1.98	2	0
ENSG00000114450	GNB4	protein_coding	5.01	5	0	4.82	4.8	0	6.5	6.5	0
ENSG00000127920	GNG11	protein_coding	NS	NS	-	NS	NS	-	2.29	2.3	0
ENSG00000172380	GNG12	protein_coding	NS	NS	-	NS	NS	-	2.2	2.2	0
ENSG00000133136	GNG5P2	protein_coding	2.21	2.2	0	2.06	2.1	0	NS	NS	-
ENSG00000124713	GNMT	protein_coding	0.05	-19.5	0	0.05	-19.7	0	0.06	-17.6	0
ENSG00000113552	GNPDA1	protein_coding	2.02	2	0	NS	NS	-	4.51	4.5	0
ENSG00000111670	GNPTAB	protein_coding	2.11	2.1	0	NS	NS	-	3.82	3.8	0
ENSG00000135677	GNS	protein_coding	2.32	2.3	0	2.2	2.2	0	3.33	3.3	0
ENSG00000215252	GOLGA8B	protein_coding	0.19	-5.1	0	0.21	-4.7	0	0.04	-23.9	0
ENSG00000173905	GOLIM4	protein_coding	2.44	2.4	0	2.47	2.5	0	NS	NS	-
ENSG00000135052	GOLM1	protein_coding	NS	NS	-	2.62	2.6	0	NS	NS	-
ENSG00000174567	GOLT1A	protein_coding	NS	NS	-	NS	NS	-	0.45	-2.2	0
ENSG00000047932	GOPC	protein_coding	NS	NS	-	NS	NS	-	2.1	2.1	0
ENSG00000169347	GP2	protein_coding	0.1	-10	0	0.11	-8.9	0	0	-232.7	0
ENSG00000183098	GPC6	protein_coding	3.25	3.3	0	3.2	3.2	0	NS	NS	-
ENSG00000115159	GPD2	protein_coding	2.31	2.3	0	2.44	2.4	0	NS	NS	-
ENSG00000164850	GPER	protein_coding	NS	NS	-	NS	NS	-	2.46	2.5	0
ENSG00000149735	GPHA2	protein_coding	0.11	-9.1	0	0.11	-8.7	0	NS	NS	-
ENSG00000105220	GPI	protein_coding	2.1	2.1	0	2.1	2.1	0	NS	NS	-
ENSG00000136235	GPNMB	protein_coding	4.9	4.9	0	4.09	4.1	0	11.46	11.5	0
ENSG00000183671	GPR1	protein_coding	NS	NS	-	NS	NS	-	5.09	5.1	0
ENSG00000153292	GPR110	protein_coding	NS	NS	-	7.42	7.4	0	NS	NS	-
ENSG00000069122	GPR116	protein_coding	NS	NS	-	NS	NS	-	0.19	-5.1	0
ENSG00000020181	GPR124	protein_coding	2.39	2.4	0	2.11	2.1	0	4.66	4.7	0
ENSG00000077585	GPR137B	protein_coding	4.52	4.5	0	4.48	4.5	0	4.78	4.8	0
ENSG00000166073	GPR176	protein_coding	NS	NS	-	NS	NS	-	8.29	8.3	0
ENSG00000169508	GPR183	protein_coding	NS	NS	-	3.46	3.5	0	NS	NS	-
ENSG00000013588	GPRC5A	protein_coding	12.57	12.6	0	13.87	13.9	0	NS	NS	-
ENSG00000170412	GPRC5C	protein_coding	0.47	-2.1	0	0.49	-2	0	NS	NS	-
ENSG00000185477	GPRIN3	protein_coding	NS	NS	-	NS	NS	-	0.15	-6.7	0

ENSG00000121957	GPSM2	protein_coding	4.35	4.4	0	3.83	3.8	0	8.51	8.5	0
ENSG00000166123	GPT2	protein_coding	0.09	-10.6	0	0.09	-10.5	0	0.09	-11.4	0
ENSG00000176153	GPX2	protein_coding	NS	NS	-	13.82	13.8	0	NS	NS	-
ENSG00000164294	GPX8	protein_coding	5.65	5.7	0	4.89	4.9	0	11.7	11.7	0
ENSG00000023171	GRAMD1B	protein_coding	3.49	3.5	0	3.59	3.6	0	2.7	2.7	0
ENSG00000178075	GRAMD1C	protein_coding	0.38	-2.7	0	0.41	-2.5	0	0.14	-7.1	0
ENSG00000155324	GRAMD3	protein_coding	2.31	2.3	0	1.95	2	0	5.15	5.1	0
ENSG00000106070	GRB10	protein_coding	0.38	-2.6	0	0.38	-2.6	0	NS	NS	-
ENSG00000115290	GRB14	protein_coding	0.22	-4.5	0	0.24	-4.2	0	0.11	-8.7	0
ENSG00000166923	GREM1	protein_coding	17.52	17.5	0	13.35	13.3	0	50.87	50.9	0
ENSG00000180875	GREM2	protein_coding	NS	NS	-	NS	NS	-	5.19	5.2	0
ENSG00000083307	GRHL2	protein_coding	NS	NS	-	NS	NS	-	0.14	-7.1	0
ENSG00000178719	GRINA	protein_coding	NS	NS	-	NS	NS	-	3.24	3.2	0
ENSG00000137878	GRINL1A	protein_coding	NS	NS	-	NS	NS	-	0.26	-3.9	0
ENSG00000160007	GRLF1	protein_coding	NS	NS	-	NS	NS	-	2.1	2.1	0
ENSG00000030582	GRN	protein_coding	2.3	2.3	0	2.11	2.1	0	3.88	3.9	0
ENSG00000126010	GRPR	protein_coding	0.08	-11.8	0	0.08	-12.3	0	NS	NS	-
ENSG00000082701	GSK3B	protein_coding	NS	NS	-	NS	NS	-	2.88	2.9	0
ENSG00000100983	GSS	protein_coding	2.09	2.1	0	2.02	2	0	NS	NS	-
ENSG00000244067	GSTA2	protein_coding	0.05	-19.3	0	0.05	-18.7	0	NS	NS	-
ENSG00000174156	GSTA3	protein_coding	0.39	-2.5	0	0.39	-2.6	0	NS	NS	-
ENSG00000084207	GSTP1	protein_coding	2.02	2	0	2.01	2	0	2.04	2	0
ENSG00000185068	GTF2H5	protein_coding	NS	NS	-	NS	NS	-	3.59	3.6	0
ENSG00000138472	GUCA1C	protein_coding	0.06	-16.6	0	0.06	-16.3	0	NS	NS	-
ENSG00000164116	GUCY1A3	protein_coding	NS	NS	-	NS	NS	-	0.12	-8.1	0
ENSG00000165905	GYLTL1B	protein_coding	NS	NS	-	NS	NS	-	0.45	-2.2	0
ENSG00000136732	GYPC	protein_coding	NS	NS	-	NS	NS	-	2.68	2.7	0
ENSG00000162882	HAAO	protein_coding	NS	NS	-	NS	NS	-	0.49	-2.1	0
ENSG00000130956	HABP4	protein_coding	NS	NS	-	NS	NS	-	2.68	2.7	0
ENSG00000140511	HAPLN3	protein_coding	2.06	2.1	0	1.97	2	0	NS	NS	-
ENSG00000135077	HAVCR2	protein_coding	NS	NS	-	2.88	2.9	0	NS	NS	-
ENSG00000172534	HCFC1	protein_coding	NS	NS	-	NS	NS	-	2.4	2.4	0
ENSG00000180353	HCLS1	protein_coding	NS	NS	-	2.47	2.5	0	NS	NS	-
ENSG00000164588	HCN1	protein_coding	0.43	-2.3	0	0.41	-2.4	0	NS	NS	-
ENSG00000206337	HCP5	processed_transcript	NS	NS	-	3.25	3.2	0	NS	NS	-
ENSG00000116478	HDAC1	protein_coding	NS	NS	-	2	2	0	NS	NS	-
ENSG00000129493	HEATR5A	protein_coding	NS	NS	-	NS	NS	-	2.41	2.4	0
ENSG00000089472	HEPH	protein_coding	4.45	4.5	0	4.41	4.4	0	4.81	4.8	0
ENSG00000138646	HERC5	protein_coding	NS	NS	-	NS	NS	-	0.4	-2.5	0
ENSG00000213614	HEXA	protein_coding	2.18	2.2	0	NS	NS	-	4.05	4	0
ENSG00000113924	HGD	protein_coding	NS	NS	-	NS	NS	-	0.05	-18.8	0
ENSG00000152804	HHEX	protein_coding	NS	NS	-	NS	NS	-	0.33	-3	0

ENSG00000148110	HIATL1	protein_coding	NS	NS	-	NS	NS	-	2.36	2.4	0
ENSG00000100644	HIF1A	protein_coding	2.5	2.5	0	2.35	2.4	0	3.62	3.6	0
ENSG00000127946	HIP1	protein_coding	2.05	2	0	2.08	2.1	0	NS	NS	-
ENSG00000184357	HIST1H1B	protein_coding	3.52	3.5	0	3.62	3.6	0	NS	NS	-
ENSG00000180573	HIST1H2AC	protein_coding	2.11	2.1	0	2.15	2.1	0	NS	NS	-
ENSG00000168274	HIST1H2AE	protein_coding	3.3	3.3	0	3.37	3.4	0	2.78	2.8	0
ENSG00000196747	HIST1H2AI	protein_coding	2.06	2.1	0	2.11	2.1	0	NS	NS	-
ENSG00000158373	HIST1H2BD	protein_coding	2.44	2.4	0	2.53	2.5	0	NS	NS	-
ENSG00000197846	HIST1H2BF	protein_coding	4.32	4.3	0	4.54	4.5	0	NS	NS	-
ENSG00000187990	HIST1H2BG	protein_coding	5.74	5.7	0	6.1	6.1	0	NS	NS	-
ENSG00000197459	HIST1H2BH	protein_coding	3.02	3	0	3.1	3.1	0	NS	NS	-
ENSG00000196374	HIST1H2BM	protein_coding	7.91	7.9	0	8.32	8.3	0	NS	NS	-
ENSG00000124693	HIST1H3B	protein_coding	10.71	10.7	0	11.35	11.4	0	NS	NS	-
ENSG00000197409	HIST1H3D	protein_coding	7.89	7.9	0	8.14	8.1	0	NS	NS	-
ENSG00000196966	HIST1H3E	protein_coding	NS	NS	-	NS	NS	-	2.72	2.7	0
ENSG00000178458	HIST1H3G	pseudogene	6.94	6.9	0	7.13	7.1	0	NS	NS	-
ENSG00000203813	HIST1H3H	protein_coding	8.48	8.5	0	8.92	8.9	0	NS	NS	-
ENSG00000182572	HIST1H3I	protein_coding	6.35	6.3	0	6.44	6.4	0	NS	NS	-
ENSG00000197153	HIST1H3J	protein_coding	2.87	2.9	0	2.96	3	0	NS	NS	-
ENSG00000124529	HIST1H4B	protein_coding	0.45	-2.2	0	0.46	-2.2	0	NS	NS	-
ENSG00000158406	HIST1H4H	protein_coding	3.23	3.2	0	2.99	3	0	NS	NS	-
ENSG00000198558	HIST1H4L	protein_coding	2.18	2.2	0	2.21	2.2	0	NS	NS	-
ENSG00000203812	HIST2H2AA4	protein_coding	4.67	4.7	0	4.63	4.6	0	NS	NS	-
ENSG00000184270	HIST2H2AB	protein_coding	2.98	3	0	3.11	3.1	0	NS	NS	-
ENSG00000184678	HIST2H2BE	protein_coding	2.77	2.8	0	2.82	2.8	0	NS	NS	-
ENSG00000156515	HK1	protein_coding	2.45	2.5	0	2.34	2.3	0	3.37	3.4	0
ENSG00000206503	HLA-A	protein_coding	3.12	3.1	0	3.25	3.2	0	NS	NS	-
ENSG00000234745	HLA-B	protein_coding	2.51	2.5	0	2.66	2.7	0	NS	NS	-
ENSG00000204525	HLA-C	protein_coding	NS	NS	-	1.96	2	0	NS	NS	-
ENSG00000204257	HLA-DMA	protein_coding	NS	NS	-	3.01	3	0	NS	NS	-
ENSG00000242574	HLA-DMB	protein_coding	NS	NS	-	3.84	3.8	0	NS	NS	-
ENSG00000231389	HLA-DPA1	protein_coding	NS	NS	-	3.12	3.1	0	NS	NS	-
ENSG00000179344	HLA-DQB1	protein_coding	NS	NS	-	3.79	3.8	0	NS	NS	-
ENSG00000204287	HLA-DRA	protein_coding	NS	NS	-	3.5	3.5	0	NS	NS	-
ENSG00000204592	HLA-E	protein_coding	1.97	2	0	2.05	2.1	0	NS	NS	-
ENSG00000204642	HLA-F	protein_coding	2.64	2.6	0	2.85	2.9	0	NS	NS	-
ENSG00000206341	HLA-H	pseudogene	NS	NS	-	2.02	2	0	NS	NS	-
ENSG00000108924	HLF	protein_coding	NS	NS	-	NS	NS	-	0.22	-4.6	0
ENSG00000143341	HMCN1	protein_coding	5.7	5.7	0	5.42	5.4	0	7.95	7.9	0
ENSG00000164104	HMGB2	protein_coding	2.46	2.5	0	2.55	2.6	0	NS	NS	-
ENSG00000112972	HMGCS1	protein_coding	2.41	2.4	0	2.32	2.3	0	NS	NS	-
ENSG00000100292	HMOX1	protein_coding	NS	NS	-	NS	NS	-	10.97	11	0

ENSG00000189159	HN1	protein_coding	4.87	4.9	0	4.99	5	0	3.88	3.9	0
ENSG00000108753	HNF1B	protein_coding	NS	NS	-	NS	NS	-	0.11	-8.7	0
ENSG00000101076	HNF4A	protein_coding	NS	NS	-	NS	NS	-	0.35	-2.8	0
ENSG00000164749	HNF4G	protein_coding	NS	NS	-	NS	NS	-	0.28	-3.6	0
ENSG00000103942	HOMER2	protein_coding	0.17	-5.9	0	0.18	-5.5	0	0.07	-14	0
ENSG00000134709	HOOK1	protein_coding	0.39	-2.5	0	0.43	-2.3	0	0.08	-12.4	0
ENSG00000095066	HOOK2	protein_coding	NS	NS	-	NS	NS	-	0.37	-2.7	0
ENSG00000105996	HOXA2	protein_coding	NS	NS	-	2.46	2.5	0	NS	NS	-
ENSG00000122592	HOXA7	protein_coding	NS	NS	-	NS	NS	-	4.08	4.1	0
ENSG00000078399	HOXA9	protein_coding	NS	NS	-	NS	NS	-	3.46	3.5	0
ENSG00000120093	HOXB3	protein_coding	2.53	2.5	0	2.66	2.7	0	NS	NS	-
ENSG00000198353	HOXC4	protein_coding	NS	NS	-	NS	NS	-	2.26	2.3	0
ENSG00000172789	HOXC5	protein_coding	NS	NS	-	NS	NS	-	2.46	2.5	0
ENSG00000197757	HOXC6	protein_coding	NS	NS	-	NS	NS	-	8.64	8.6	0
ENSG00000037965	HOXC8	protein_coding	NS	NS	-	NS	NS	-	5.38	5.4	0
ENSG00000164120	HPGD	protein_coding	NS	NS	-	7.11	7.1	0	NS	NS	-
ENSG00000105707	HPN	protein_coding	0.24	-4.2	0	0.24	-4.1	0	0.22	-4.6	0
ENSG00000168004	HRASLS5	protein_coding	0.27	-3.6	0	0.27	-3.6	0	NS	NS	-
ENSG00000125430	HS3ST3B1	protein_coding	NS	NS	-	NS	NS	-	4.32	4.3	0
ENSG00000171004	HS6ST2	protein_coding	0.51	-2	0	0.51	-2	0	NS	NS	-
ENSG00000087076	HSD17B14	protein_coding	NS	NS	-	NS	NS	-	3.84	3.8	0
ENSG00000204228	HSD17B8	protein_coding	0.48	-2.1	0	0.5	-2	0	NS	NS	-
ENSG00000164070	HSPA4L	protein_coding	0.47	-2.1	0	0.43	-2.3	0	NS	NS	-
ENSG00000169271	HSPB3	protein_coding	NS	NS	-	NS	NS	-	2.61	2.6	0
ENSG00000142798	HSPG2	protein_coding	2.7	2.7	0	2.76	2.8	0	NS	NS	-
ENSG00000120694	HSPH1	protein_coding	2.05	2.1	0	2.11	2.1	0	NS	NS	-
ENSG00000109854	HTATIP2	protein_coding	2.56	2.6	0	2.62	2.6	0	NS	NS	-
ENSG00000102468	HTR2A	protein_coding	NS	NS	-	NS	NS	-	4.4	4.4	0
ENSG00000166033	HTRA1	protein_coding	2.88	2.9	0	2.48	2.5	0	6.07	6.1	0
ENSG00000170801	HTRA3	protein_coding	NS	NS	-	2.24	2.2	0	NS	NS	-
ENSG00000142149	HUNK	protein_coding	0.48	-2.1	0	0.45	-2.2	0	NS	NS	-
ENSG00000122986	HVCN1	protein_coding	NS	NS	-	2.42	2.4	0	NS	NS	-
ENSG00000121351	IAPP	protein_coding	0.11	-9.4	0	0.12	-8.5	0	NS	NS	-
ENSG00000003147	ICA1	protein_coding	NS	NS	-	NS	NS	-	0.11	-8.7	0
ENSG00000090339	ICAM1	protein_coding	2.99	3	0	2.82	2.8	0	NS	NS	-
ENSG00000108622	ICAM2	protein_coding	NS	NS	-	NS	NS	-	0.4	-2.5	0
ENSG00000117318	ID3	protein_coding	NS	NS	-	NS	NS	-	12.55	12.5	0
ENSG00000172201	ID4	protein_coding	NS	NS	-	0.49	-2	0	NS	NS	-
ENSG00000138413	IDH1	protein_coding	NS	NS	-	1.99	2	0	NS	NS	-
ENSG00000163565	IFI16	protein_coding	2.96	3	0	3	3	0	NS	NS	-
ENSG00000165949	IFI27	protein_coding	NS	NS	-	7.3	7.3	0	NS	NS	-
ENSG00000216490	IFI30	protein_coding	NS	NS	-	3.56	3.6	0	NS	NS	-

ENSG00000185201	IFITM2	protein_coding	2.45	2.5	0	2.21	2.2	0	NS	NS	-
ENSG00000142089	IFITM3	protein_coding	2.51	2.5	0	2.27	2.3	0	4.38	4.4	0
ENSG00000159110	IFNAR2	protein_coding	2.33	2.3	0	2.49	2.5	0	NS	NS	-
ENSG00000159128	IFNGR2	protein_coding	2.06	2.1	0	2.1	2.1	0	NS	NS	-
ENSG00000006652	IFRD1	protein_coding	0.31	-3.2	0	0.31	-3.2	0	NS	NS	-
ENSG00000109083	IFT20	protein_coding	0.43	-2.3	0	0.43	-2.3	0	NS	NS	-
ENSG00000136231	IGF2BP3	protein_coding	NS	NS	-	NS	NS	-	3.26	3.3	0
ENSG00000197081	IGF2R	protein_coding	NS	NS	-	NS	NS	-	2.3	2.3	0
ENSG00000146674	IGFBP3	protein_coding	4.68	4.7	0	4.23	4.2	0	8.33	8.3	0
ENSG00000115461	IGFBP5	protein_coding	6.39	6.4	0	6.66	6.7	0	NS	NS	-
ENSG00000167779	IGFBP6	protein_coding	NS	NS	-	NS	NS	-	2.89	2.9	0
ENSG00000163395	IGFN1	protein_coding	0.43	-2.3	0	0.43	-2.3	0	NS	NS	-
ENSG00000211895	IGHA1	IG_C_gene	NS	NS	-	NS	NS	-	0.07	-15.1	0
ENSG00000211892	IGHG4	IG_C_gene	NS	NS	-	7.65	7.7	0	NS	NS	-
ENSG00000152580	IGSF10	protein_coding	NS	NS	-	0.45	-2.2	0	NS	NS	-
ENSG00000144847	IGSF11	protein_coding	NS	NS	-	NS	NS	-	0.48	-2.1	0
ENSG00000183067	IGSF5	protein_coding	0.45	-2.2	0	0.45	-2.2	0	NS	NS	-
ENSG00000166130	IKBIP	protein_coding	2.38	2.4	0	NS	NS	-	5.93	5.9	0
ENSG00000030419	IKZF2	protein_coding	2.09	2.1	0	2.2	2.2	0	NS	NS	-
ENSG00000110324	IL10RA	protein_coding	NS	NS	-	2.29	2.3	0	NS	NS	-
ENSG00000131724	IL13RA1	protein_coding	2.1	2.1	0	2.17	2.2	0	NS	NS	-
ENSG00000123496	IL13RA2	protein_coding	NS	NS	-	NS	NS	-	21.87	21.9	0
ENSG00000172349	IL16	protein_coding	NS	NS	-	2.09	2.1	0	NS	NS	-
ENSG00000056736	IL17RB	protein_coding	NS	NS	-	NS	NS	-	0.36	-2.8	0
ENSG00000150782	IL18	protein_coding	NS	NS	-	3.19	3.2	0	NS	NS	-
ENSG00000196083	IL1RAP	protein_coding	3.94	3.9	0	4.2	4.2	0	NS	NS	-
ENSG00000136689	IL1RN	protein_coding	NS	NS	-	4.73	4.7	0	NS	NS	-
ENSG00000142677	IL22RA1	protein_coding	0.23	-4.4	0	0.24	-4.1	0	0.13	-7.9	0
ENSG00000134460	IL2RA	protein_coding	NS	NS	-	2.62	2.6	0	NS	NS	-
ENSG00000147168	IL2RG	protein_coding	NS	NS	-	8.19	8.2	0	NS	NS	-
ENSG00000008517	IL32	protein_coding	NS	NS	-	NS	NS	-	0.21	-4.9	0
ENSG00000104432	IL7	protein_coding	2.32	2.3	0	2.35	2.4	0	NS	NS	-
ENSG00000166333	ILK	protein_coding	NS	NS	-	NS	NS	-	2.02	2	0
ENSG00000141401	IMPA2	protein_coding	0.25	-4	0	0.26	-3.8	0	NS	NS	-
ENSG00000132849	INADL	protein_coding	NS	NS	-	NS	NS	-	0.19	-5.4	0
ENSG00000203485	INF2	protein_coding	NS	NS	-	NS	NS	-	2.42	2.4	0
ENSG00000122641	INHBA	protein_coding	14.46	14.5	0	14.11	14.1	0	17.25	17.2	0
ENSG00000109452	INPP4B	protein_coding	3.74	3.7	0	3.95	4	0	NS	NS	-
ENSG00000168918	INPP5D	protein_coding	NS	NS	-	3.31	3.3	0	NS	NS	-
ENSG00000198825	INPP5F	protein_coding	2.01	2	0	2.09	2.1	0	NS	NS	-
ENSG00000129965	INS	protein_coding	NS	NS	-	NS	NS	-	0.08	-13	0
ENSG00000171105	INSR	protein_coding	NS	NS	-	NS	NS	-	0.17	-5.9	0

ENSG00000108506	INTS2	protein_coding	NS	NS	-	1.98	2	0	NS	NS	-
ENSG00000102786	INTS6	protein_coding	NS	NS	-	NS	NS	-	0.48	-2.1	0
ENSG00000117408	IPO13	protein_coding	NS	NS	-	NS	NS	-	3.54	3.5	0
ENSG00000127080	IPPK	protein_coding	NS	NS	-	NS	NS	-	2.04	2	0
ENSG00000140575	IQGAP1	protein_coding	1.97	2	0	1.99	2	0	NS	NS	-
ENSG00000145703	IQGAP2	protein_coding	NS	NS	-	NS	NS	-	0.02	-40.4	0
ENSG00000146243	IRAK1BP1	protein_coding	0.49	-2	0	0.49	-2.1	0	NS	NS	-
ENSG00000134070	IRAK2	protein_coding	2.08	2.1	0	2.17	2.2	0	NS	NS	-
ENSG00000090376	IRAK3	protein_coding	2.97	3	0	3.19	3.2	0	NS	NS	-
ENSG00000125347	IRF1	protein_coding	2.36	2.4	0	2.28	2.3	0	NS	NS	-
ENSG00000117595	IRF6	protein_coding	NS	NS	-	NS	NS	-	0.15	-6.6	0
ENSG00000140968	IRF8	protein_coding	NS	NS	-	3.23	3.2	0	NS	NS	-
ENSG00000169047	IRS1	protein_coding	2.02	2	0	NS	NS	-	2.87	2.9	0
ENSG00000129009	ISLR	protein_coding	4.71	4.7	0	4.47	4.5	0	NS	NS	-
ENSG00000105655	ISYNA1	protein_coding	0.48	-2.1	0	0.5	-2	0	0.33	-3.1	0
ENSG00000213949	ITGA1	protein_coding	4.06	4.1	0	4.38	4.4	0	NS	NS	-
ENSG00000137809	ITGA11	protein_coding	7.56	7.6	0	5.76	5.8	0	21.93	21.9	0
ENSG00000164171	ITGA2	protein_coding	12.53	12.5	0	13.94	13.9	0	NS	NS	-
ENSG00000005884	ITGA3	protein_coding	5.38	5.4	0	5.55	5.5	0	NS	NS	-
ENSG00000115232	ITGA4	protein_coding	2.88	2.9	0	2.86	2.9	0	NS	NS	-
ENSG00000161638	ITGA5	protein_coding	2.88	2.9	0	2.44	2.4	0	6.41	6.4	0
ENSG00000169896	ITGAM	protein_coding	NS	NS	-	2.28	2.3	0	NS	NS	-
ENSG00000138448	ITGAV	protein_coding	2.38	2.4	0	2.41	2.4	0	NS	NS	-
ENSG00000140678	ITGAX	protein_coding	NS	NS	-	2.65	2.6	0	NS	NS	-
ENSG00000150093	ITGB1	protein_coding	2.36	2.4	0	2.33	2.3	0	2.65	2.6	0
ENSG00000160255	ITGB2	protein_coding	NS	NS	-	2.93	2.9	0	NS	NS	-
ENSG00000132470	ITGB4	protein_coding	NS	NS	-	4.82	4.8	0	0.33	-3	0
ENSG00000082781	ITGB5	protein_coding	3.94	3.9	0	3.61	3.6	0	6.58	6.6	0
ENSG00000115221	ITGB6	protein_coding	NS	NS	-	9.66	9.7	0	0.15	-6.6	0
ENSG00000198542	ITGBL1	protein_coding	4.62	4.6	0	NS	NS	-	6.95	7	0
ENSG00000123104	ITPR2	protein_coding	NS	NS	-	NS	NS	-	0.27	-3.7	0
ENSG00000096433	ITPR3	protein_coding	3.2	3.2	0	3.23	3.2	0	NS	NS	-
ENSG00000148841	ITPRIP	protein_coding	NS	NS	-	NS	NS	-	3.69	3.7	0
ENSG00000205730	ITPRIPL2	protein_coding	NS	NS	-	NS	NS	-	2.02	2	0
ENSG00000198399	ITSN2	protein_coding	NS	NS	-	NS	NS	-	0.29	-3.5	0
ENSG00000182264	IZUMO1	protein_coding	0.45	-2.2	0	0.45	-2.2	0	NS	NS	-
ENSG00000210144	J01415.10	Mt_tRNA	0.31	-3.3	0	0.32	-3.1	0	0.21	-4.7	0
ENSG00000210176	J01415.15	Mt_tRNA	0.46	-2.2	0	0.42	-2.4	0	NS	NS	-
ENSG00000210184	J01415.19	Mt_tRNA	0.43	-2.3	0	0.41	-2.4	0	NS	NS	-
ENSG00000210191	J01415.23	Mt_tRNA	NS	NS	-	0.25	-4	0	NS	NS	-
ENSG00000210140	J01415.28	Mt_tRNA	0.39	-2.6	0	0.39	-2.6	0	NS	NS	-
ENSG00000210112	J01415.29	Mt_tRNA	NS	NS	-	NS	NS	-	0.4	-2.5	0

ENSG00000209082	J01415.7	Mt_tRNA	0.47	-2.1	0	0.45	-2.2	0	NS	NS	-
ENSG00000096968	JAK2	protein_coding	NS	NS	-	2.06	2.1	0	NS	NS	-
ENSG00000105639	JAK3	protein_coding	NS	NS	-	1.99	2	0	NS	NS	-
ENSG00000166086	JAM3	protein_coding	2.51	2.5	0	2.29	2.3	0	4.33	4.3	0
ENSG00000155666	JMJD5	protein_coding	0.49	-2.1	0	0.48	-2.1	0	0.5	-2	0
ENSG00000070495	JMJD6	protein_coding	NS	NS	-	NS	NS	-	2.21	2.2	0
ENSG00000104369	JPH1	protein_coding	NS	NS	-	NS	NS	-	0.32	-3.1	0
ENSG00000011201	KAL1	protein_coding	NS	NS	-	4	4	0	NS	NS	-
ENSG00000197256	KANK2	protein_coding	NS	NS	-	NS	NS	-	2.54	2.5	0
ENSG00000069424	KCNAB2	protein_coding	2.01	2	0	2.08	2.1	0	NS	NS	-
ENSG00000184408	KCND2	protein_coding	2.66	2.7	0	2.73	2.7	0	NS	NS	-
ENSG00000171126	KCNG3	protein_coding	0.47	-2.1	0	0.47	-2.1	0	NS	NS	-
ENSG00000153822	KCNJ16	protein_coding	0.15	-6.6	0	0.17	-6	0	0.03	-31.1	0
ENSG00000123700	KCNJ2	protein_coding	NS	NS	-	NS	NS	-	2.44	2.4	0
ENSG00000120457	KCNJ5	protein_coding	0.23	-4.3	0	0.24	-4.1	0	NS	NS	-
ENSG00000121361	KCNJ8	protein_coding	0.32	-3.1	0	0.33	-3	0	0.26	-3.9	0
ENSG00000082482	KCNK2	protein_coding	NS	NS	-	NS	NS	-	33.42	33.4	0
ENSG00000171303	KCNK3	protein_coding	0.28	-3.6	0	0.28	-3.5	0	NS	NS	-
ENSG00000053918	KCNQ1	protein_coding	NS	NS	-	NS	NS	-	0.36	-2.8	0
ENSG00000162687	KCNT2	protein_coding	NS	NS	-	2.03	2	0	NS	NS	-
ENSG00000110906	KCTD10	protein_coding	2.13	2.1	0	NS	NS	-	3.73	3.7	0
ENSG00000183775	KCTD16	protein_coding	0.46	-2.2	0	0.45	-2.2	0	NS	NS	-
ENSG00000112078	KCTD20	protein_coding	2.15	2.1	0	2.05	2.1	0	NS	NS	-
ENSG00000134901	KDELC1	protein_coding	3.65	3.7	0	3.4	3.4	0	5.72	5.7	0
ENSG00000178202	KDELC2	protein_coding	2.38	2.4	0	2.3	2.3	0	NS	NS	-
ENSG00000105438	KDELR1	protein_coding	NS	NS	-	NS	NS	-	2.66	2.7	0
ENSG00000100196	KDELR3	protein_coding	2.38	2.4	0	2.22	2.2	0	NS	NS	-
ENSG00000128052	KDR	protein_coding	NS	NS	-	NS	NS	-	0.12	-8.6	0
ENSG00000119537	KDSR	protein_coding	NS	NS	-	NS	NS	-	3.1	3.1	0
ENSG00000127463	KIAA0090	protein_coding	NS	NS	-	NS	NS	-	2.42	2.4	0
ENSG00000166803	KIAA0101	protein_coding	2.91	2.9	0	2.91	2.9	0	NS	NS	-
ENSG00000164542	KIAA0895	protein_coding	NS	NS	-	NS	NS	-	0.38	-2.7	0
ENSG00000121210	KIAA0922	protein_coding	0.37	-2.7	0	0.38	-2.6	0	NS	NS	-
ENSG00000103888	KIAA1199	protein_coding	12.52	12.5	0	6.05	6.1	0	64.27	64.3	0
ENSG00000112379	KIAA1244	protein_coding	NS	NS	-	NS	NS	-	0.16	-6.2	0
ENSG00000116299	KIAA1324	protein_coding	0.21	-4.7	0	0.24	-4.2	0	0.02	-45.5	0
ENSG00000133958	KIAA1409	protein_coding	0.32	-3.1	0	0.33	-3	0	0.22	-4.6	0
ENSG00000163507	KIAA1524	protein_coding	2.85	2.9	0	2.93	2.9	0	NS	NS	-
ENSG00000005238	KIAA1539	protein_coding	NS	NS	-	NS	NS	-	2.85	2.9	0
ENSG00000144320	KIAA1715	protein_coding	2.69	2.7	0	2.57	2.6	0	NS	NS	-
ENSG00000146112	KIAA1949	protein_coding	2.35	2.4	0	2.13	2.1	0	4.14	4.1	0
ENSG00000138160	KIF11	protein_coding	2.91	2.9	0	3.03	3	0	NS	NS	-

ENSG00000136883	KIF12	protein_coding	0.45	-2.2	0	0.47	-2.1	0	0.28	-3.6	0
ENSG00000121621	KIF18A	protein_coding	2.84	2.8	0	NS	NS	-	4.21	4.2	0
ENSG00000130294	KIF1A	protein_coding	0.22	-4.5	0	0.23	-4.4	0	0.17	-6	0
ENSG00000112984	KIF20A	protein_coding	2.94	2.9	0	2.89	2.9	0	NS	NS	-
ENSG00000138182	KIF20B	protein_coding	2.05	2.1	0	2.11	2.1	0	NS	NS	-
ENSG00000139116	KIF21A	protein_coding	NS	NS	-	NS	NS	-	0.31	-3.3	0
ENSG00000162849	KIF26B	protein_coding	2.2	2.2	0	2.25	2.3	0	NS	NS	-
ENSG00000084731	KIF3C	protein_coding	NS	NS	-	NS	NS	-	2.51	2.5	0
ENSG00000183853	KIRREL	protein_coding	2.95	3	0	2.61	2.6	0	5.73	5.7	0
ENSG00000126259	KIRREL2	protein_coding	0.32	-3.2	0	0.31	-3.2	0	0.36	-2.8	0
ENSG00000157404	KIT	protein_coding	NS	NS	-	NS	NS	-	3.23	3.2	0
ENSG00000049130	KITLG	protein_coding	3.26	3.3	0	3.21	3.2	0	3.71	3.7	0
ENSG00000134962	KLB	protein_coding	0.28	-3.6	0	0.27	-3.7	0	NS	NS	-
ENSG00000118922	KLF12	protein_coding	2.46	2.5	0	2.45	2.5	0	NS	NS	-
ENSG00000163884	KLF15	protein_coding	0.16	-6.4	0	0.16	-6.4	0	0.16	-6.2	0
ENSG00000172578	KLHL6	protein_coding	NS	NS	-	2.95	3	0	NS	NS	-
ENSG00000167748	KLK1	protein_coding	0.09	-10.8	0	0.1	-9.8	0	0.02	-54.1	0
ENSG00000167759	KLK13	protein_coding	0.43	-2.3	0	0.43	-2.3	0	NS	NS	-
ENSG00000164344	KLKB1	protein_coding	0.32	-3.1	0	0.33	-3	0	0.22	-4.6	0
ENSG00000182481	KPNA2	protein_coding	4.12	4.1	0	3.57	3.6	0	8.49	8.5	0
ENSG00000133703	KRAS	protein_coding	2.59	2.6	0	2.75	2.8	0	NS	NS	-
ENSG00000183762	KREMEN1	protein_coding	NS	NS	-	NS	NS	-	2.33	2.3	0
ENSG00000111057	KRT18	protein_coding	NS	NS	-	NS	NS	-	0.06	-16.2	0
ENSG00000171345	KRT19	protein_coding	8.45	8.4	0	9.44	9.4	0	NS	NS	-
ENSG00000135480	KRT7	protein_coding	4.71	4.7	0	5.09	5.1	0	NS	NS	-
ENSG00000170421	KRT8	protein_coding	NS	NS	-	NS	NS	-	0.04	-25.9	0
ENSG00000189169	KRTAP10-12	protein_coding	0.5	-2	0	0.49	-2	0	NS	NS	-
ENSG00000205445	KRTAP10-2	protein_coding	0.5	-2	0	0.5	-2	0	NS	NS	-
ENSG00000157992	KRTCAP3	protein_coding	NS	NS	-	NS	NS	-	0.41	-2.4	0
ENSG00000141068	KSR1	protein_coding	0.31	-3.2	0	0.32	-3.1	0	NS	NS	-
ENSG00000115919	KYNU	protein_coding	NS	NS	-	6.13	6.1	0	NS	NS	-
ENSG00000198945	L3MBTL3	protein_coding	2.32	2.3	0	2.37	2.4	0	NS	NS	-
ENSG00000154655	L3MBTL4	protein_coding	NS	NS	-	NS	NS	-	0.3	-3.4	0
ENSG00000159166	LAD1	protein_coding	NS	NS	-	NS	NS	-	0.31	-3.3	0
ENSG00000167613	LAIR1	protein_coding	NS	NS	-	2.35	2.3	0	NS	NS	-
ENSG00000101680	LAMA1	protein_coding	NS	NS	-	NS	NS	-	2.75	2.8	0
ENSG00000053747	LAMA3	protein_coding	3.26	3.3	0	3.5	3.5	0	NS	NS	-
ENSG00000112769	LAMA4	protein_coding	4.45	4.5	0	3.93	3.9	0	8.66	8.7	0
ENSG00000091136	LAMB1	protein_coding	2	2	0	2.05	2.1	0	NS	NS	-
ENSG00000196878	LAMB3	protein_coding	9.08	9.1	0	10.09	10.1	0	NS	NS	-
ENSG00000135862	LAMC1	protein_coding	2.41	2.4	0	2.08	2.1	0	5.06	5.1	0
ENSG00000058085	LAMC2	protein_coding	18.84	18.8	0	21.01	21	0	NS	NS	-

ENSG00000185896	LAMP1	protein_coding	NS	NS	-	NS	NS	-	2.39	2.4	0
ENSG00000162511	LAPTM5	protein_coding	NS	NS	-	3.28	3.3	0	0.12	-8.5	0
ENSG00000138709	LARP1B	protein_coding	0.36	-2.8	0	0.38	-2.6	0	0.22	-4.6	0
ENSG00000166173	LARP6	protein_coding	NS	NS	-	NS	NS	-	3.07	3.1	0
ENSG00000002834	LASP1	protein_coding	2.11	2.1	0	NS	NS	-	3.45	3.5	0
ENSG00000090661	LASS4	protein_coding	0.34	-3	0	0.34	-2.9	0	0.27	-3.7	0
ENSG00000172292	LASS6	protein_coding	NS	NS	-	2.09	2.1	0	NS	NS	-
ENSG00000204381	LAYN	protein_coding	5.42	5.4	0	3.76	3.8	0	18.73	18.7	0
ENSG00000168016	LBA1	protein_coding	3.2	3.2	0	3.31	3.3	0	NS	NS	-
ENSG00000213626	LBH	protein_coding	3.17	3.2	0	3.18	3.2	0	NS	NS	-
ENSG00000148346	LCN2	protein_coding	NS	NS	-	14.25	14.3	0	NS	NS	-
ENSG00000136167	LCP1	protein_coding	NS	NS	-	3.49	3.5	0	0.12	-8.5	0
ENSG00000043462	LCP2	protein_coding	NS	NS	-	3.09	3.1	0	NS	NS	-
ENSG00000182195	LDOC1	protein_coding	NS	NS	-	NS	NS	-	4.08	4.1	0
ENSG00000138795	LEF1	protein_coding	10.12	10.1	0	10.91	10.9	0	NS	NS	-
ENSG00000125505	LENG4	protein_coding	NS	NS	-	NS	NS	-	2.48	2.5	0
ENSG00000117385	LEPRE1	protein_coding	NS	NS	-	NS	NS	-	2.19	2.2	0
ENSG00000100097	LGALS1	protein_coding	3.97	4	0	3.61	3.6	0	6.86	6.9	0
ENSG00000131981	LGALS3	protein_coding	2.97	3	0	2.98	3	0	2.92	2.9	0
ENSG00000108679	LGALS3BP	protein_coding	2.63	2.6	0	2.81	2.8	0	NS	NS	-
ENSG00000171747	LGALS4	protein_coding	NS	NS	-	NS	NS	-	0.14	-7.3	0
ENSG00000168961	LGALS9	protein_coding	2.63	2.6	0	2.72	2.7	0	NS	NS	-
ENSG00000170298	LGALS9B	protein_coding	NS	NS	-	2.39	2.4	0	NS	NS	-
ENSG00000171916	LGALS9C	protein_coding	0.36	-2.8	0	0.38	-2.6	0	NS	NS	-
ENSG00000100600	LGMN	protein_coding	1.99	2	0	2.03	2	0	NS	NS	-
ENSG00000145685	LHFPL2	protein_coding	2.03	2	0	NS	NS	-	3.36	3.4	0
ENSG00000197753	LHFPL5	protein_coding	0.37	-2.7	0	0.37	-2.7	0	NS	NS	-
ENSG00000113594	LIFR	protein_coding	0.21	-4.7	0	0.22	-4.6	0	NS	NS	-
ENSG00000204577	LILRB3	protein_coding	NS	NS	-	1.97	2	0	NS	NS	-
ENSG00000050405	LIMA1	protein_coding	2.4	2.4	0	2.24	2.2	0	3.68	3.7	0
ENSG00000106683	LIMK1	protein_coding	2.32	2.3	0	2.12	2.1	0	3.96	4	0
ENSG00000107798	LIPA	protein_coding	2.4	2.4	0	2.37	2.4	0	NS	NS	-
ENSG00000101670	LIPG	protein_coding	NS	NS	-	3.22	3.2	0	NS	NS	-
ENSG00000163898	LIPH	protein_coding	NS	NS	-	2.63	2.6	0	0.07	-14.4	0
ENSG00000131899	LLGL1	protein_coding	NS	NS	-	NS	NS	-	2.07	2.1	0
ENSG00000073350	LLGL2	protein_coding	NS	NS	-	NS	NS	-	0.31	-3.2	0
ENSG00000100258	LMF2	protein_coding	0.44	-2.3	0	0.4	-2.5	0	NS	NS	-
ENSG00000176619	LMNB2	protein_coding	NS	NS	-	NS	NS	-	2.07	2.1	0
ENSG00000048540	LMO3	protein_coding	0.19	-5.4	0	0.2	-5	0	NS	NS	-
ENSG00000143013	LMO4	protein_coding	2.45	2.5	0	2.53	2.5	0	NS	NS	-
ENSG00000136153	LMO7	protein_coding	3.11	3.1	0	3	3	0	3.96	4	0
ENSG00000072201	LNX1	protein_coding	0.48	-2.1	0	0.48	-2.1	0	NS	NS	-

ENSG00000139517	LNX2	protein_coding	NS	NS	-	NS	NS	-	0.28	-3.6	0
ENSG00000170500	LONRF2	protein_coding	0.42	-2.4	0	0.44	-2.3	0	0.26	-3.8	0
ENSG00000113083	LOX	protein_coding	10.78	10.8	0	7.11	7.1	0	40.1	40.1	0
ENSG00000129038	LOXL1	protein_coding	NS	NS	-	NS	NS	-	3.16	3.2	0
ENSG00000134013	LOXL2	protein_coding	4.06	4.1	0	NS	NS	-	8.77	8.8	0
ENSG00000198121	LPAR1	protein_coding	NS	NS	-	NS	NS	-	3.29	3.3	0
ENSG00000171517	LPAR3	protein_coding	0.19	-5.4	0	0.19	-5.3	0	NS	NS	-
ENSG00000139679	LPAR6	protein_coding	NS	NS	-	2.87	2.9	0	NS	NS	-
ENSG00000087253	LPCAT2	protein_coding	2.67	2.7	0	2.84	2.8	0	NS	NS	-
ENSG00000176454	LPCAT4	protein_coding	3.12	3.1	0	3.24	3.2	0	2.15	2.1	0
ENSG00000110031	LPXN	protein_coding	2.33	2.3	0	NS	NS	-	4.08	4.1	0
ENSG00000198589	LRBA	protein_coding	NS	NS	-	NS	NS	-	0.41	-2.4	0
ENSG00000136141	LRCH1	protein_coding	2.22	2.2	0	2.34	2.3	0	NS	NS	-
ENSG00000130224	LRCH2	protein_coding	2.03	2	0	2.04	2	0	NS	NS	-
ENSG00000144749	LRIG1	protein_coding	0.32	-3.1	0	0.34	-3	0	NS	NS	-
ENSG00000197324	LRP10	protein_coding	2.6	2.6	0	2.28	2.3	0	5.14	5.1	0
ENSG00000147650	LRP12	protein_coding	NS	NS	-	NS	NS	-	4.72	4.7	0
ENSG00000137269	LRRC1	protein_coding	NS	NS	-	NS	NS	-	0.36	-2.8	0
ENSG00000172061	LRRC15	protein_coding	NS	NS	-	NS	NS	-	6.47	6.5	0
ENSG00000137507	LRRC32	protein_coding	3.3	3.3	0	2.88	2.9	0	6.7	6.7	0
ENSG00000033122	LRRC7	protein_coding	0.51	-2	0	0.51	-2	0	NS	NS	-
ENSG00000136802	LRRC8A	protein_coding	NS	NS	-	NS	NS	-	2.13	2.1	0
ENSG00000197147	LRRC8B	protein_coding	NS	NS	-	NS	NS	-	0.19	-5.2	0
ENSG00000177363	LRRN4CL	protein_coding	NS	NS	-	NS	NS	-	5.37	5.4	0
ENSG00000160285	LSS	protein_coding	NS	NS	-	NS	NS	-	4.22	4.2	0
ENSG00000049323	LTBP1	protein_coding	4.72	4.7	0	4.84	4.8	0	NS	NS	-
ENSG00000119681	LTBP2	protein_coding	3.55	3.5	0	3.3	3.3	0	5.51	5.5	0
ENSG00000139329	LUM	protein_coding	2.93	2.9	0	3	3	0	NS	NS	-
ENSG00000169641	LUZP1	protein_coding	2.24	2.2	0	2.14	2.1	0	2.99	3	0
ENSG00000079257	LXN	protein_coding	NS	NS	-	4.47	4.5	0	NS	NS	-
ENSG00000160932	LY6E	protein_coding	3.44	3.4	0	3.32	3.3	0	NS	NS	-
ENSG00000054219	LY75	protein_coding	5.69	5.7	0	6.3	6.3	0	NS	NS	-
ENSG00000154589	LY96	protein_coding	3.41	3.4	0	NS	NS	-	7.95	7.9	0
ENSG00000133800	LYVE1	protein_coding	NS	NS	-	NS	NS	-	0.14	-7.1	0
ENSG00000090382	LYZ	protein_coding	NS	NS	-	2.51	2.5	0	0.01	-108.1	0
ENSG00000197063	MAFG	protein_coding	NS	NS	-	NS	NS	-	2.3	2.3	0
ENSG00000102316	MAGED2	protein_coding	NS	NS	-	NS	NS	-	2.32	2.3	0
ENSG00000187391	MAGI2	protein_coding	NS	NS	-	NS	NS	-	2.07	2.1	0
ENSG00000081026	MAGI3	protein_coding	NS	NS	-	NS	NS	-	0.38	-2.6	0
ENSG00000147676	MAL2	processed_transcript	NS	NS	-	NS	NS	-	0.04	-26.4	0
ENSG00000144063	MALL	protein_coding	NS	NS	-	4.57	4.6	0	NS	NS	-
ENSG00000177239	MAN1B1	protein_coding	NS	NS	-	NS	NS	-	2.19	2.2	0

ENSG00000117643	MAN1C1	protein_coding	NS	NS	-	NS	NS	-	2.81	2.8	0
ENSG00000166963	MAP1A	protein_coding	4.09	4.1	0	2.79	2.8	0	14.49	14.5	0
ENSG00000131711	MAP1B	protein_coding	2.86	2.9	0	2.48	2.5	0	5.88	5.9	0
ENSG00000171471	MAP1LC3B2	protein_coding	NS	NS	-	NS	NS	-	2.76	2.8	0
ENSG00000034152	MAP2K3	protein_coding	NS	NS	-	NS	NS	-	3.42	3.4	0
ENSG00000095015	MAP3K1	protein_coding	NS	NS	-	NS	NS	-	0.2	-4.9	0
ENSG00000073803	MAP3K13	protein_coding	NS	NS	-	NS	NS	-	0.19	-5.2	0
ENSG00000143674	MAP3K19	protein_coding	0.36	-2.8	0	0.37	-2.7	0	0.21	-4.7	0
ENSG00000197442	MAP3K5	protein_coding	NS	NS	-	NS	NS	-	0.21	-4.8	0
ENSG00000157625	MAP3K7IP3	protein_coding	NS	NS	-	NS	NS	-	0.48	-2.1	0
ENSG00000071054	MAP4K4	protein_coding	3.42	3.4	0	3.25	3.3	0	4.79	4.8	0
ENSG00000135525	MAP7	protein_coding	NS	NS	-	NS	NS	-	0.33	-3	0
ENSG00000129680	MAP7D3	protein_coding	NS	NS	-	NS	NS	-	4.12	4.1	0
ENSG00000156711	MAPK13	protein_coding	NS	NS	-	NS	NS	-	0.38	-2.6	0
ENSG00000069956	MAPK6	protein_coding	NS	NS	-	NS	NS	-	2.23	2.2	0
ENSG00000121653	MAPK8IP1	protein_coding	0.43	-2.3	0	0.43	-2.3	0	0.48	-2.1	0
ENSG00000155130	MARCKS	protein_coding	NS	NS	-	NS	NS	-	2.02	2	0
ENSG00000155254	MARVELD1	protein_coding	2.61	2.6	0	2.38	2.4	0	4.43	4.4	0
ENSG00000140832	MARVELD3	protein_coding	NS	NS	-	NS	NS	-	0.39	-2.6	0
ENSG00000127241	MASP1	protein_coding	NS	NS	-	NS	NS	-	4.12	4.1	0
ENSG00000086015	MAST2	protein_coding	2.3	2.3	0	2.25	2.2	0	2.68	2.7	0
ENSG00000151224	MAT1A	protein_coding	0.2	-5	0	0.2	-5	0	NS	NS	-
ENSG00000132031	MATN3	protein_coding	5.99	6	0	6.53	6.5	0	NS	NS	-
ENSG00000143797	MBOAT2	protein_coding	4.19	4.2	0	4.35	4.4	0	NS	NS	-
ENSG00000078070	MCCC1	protein_coding	0.46	-2.2	0	0.47	-2.1	0	0.37	-2.7	0
ENSG00000112118	MCM3	protein_coding	2.13	2.1	0	2.21	2.2	0	NS	NS	-
ENSG00000076003	MCM6	protein_coding	2.44	2.4	0	2.4	2.4	0	NS	NS	-
ENSG00000090674	MCOLN1	protein_coding	NS	NS	-	NS	NS	-	3.46	3.5	0
ENSG00000055732	MCOLN3	protein_coding	0.2	-5	0	0.2	-5.1	0	NS	NS	-
ENSG00000140563	MCTP2	protein_coding	NS	NS	-	NS	NS	-	0.28	-3.6	0
ENSG00000112139	MDGA1	protein_coding	NS	NS	-	NS	NS	-	2.48	2.5	0
ENSG00000085276	MDS1	protein_coding	NS	NS	-	NS	NS	-	0.12	-8.4	0
ENSG00000108590	MED31	protein_coding	NS	NS	-	NS	NS	-	2.52	2.5	0
ENSG00000143995	MEIS1	protein_coding	NS	NS	-	NS	NS	-	0.26	-3.9	0
ENSG00000165304	MELK	protein_coding	3.13	3.1	0	NS	NS	-	4.11	4.1	0
ENSG00000112818	MEP1A	protein_coding	0.25	-3.9	0	0.26	-3.9	0	NS	NS	-
ENSG00000153208	MERTK	protein_coding	NS	NS	-	NS	NS	-	0.14	-7.2	0
ENSG00000105976	MET	protein_coding	4.73	4.7	0	5.08	5.1	0	NS	NS	-
ENSG00000176845	METRNL	protein_coding	NS	NS	-	NS	NS	-	2.11	2.1	0
ENSG00000117122	MFAP2	protein_coding	3.8	3.8	0	3.35	3.4	0	7.41	7.4	0
ENSG00000197614	MFAP5	protein_coding	NS	NS	-	NS	NS	-	33.01	33	0
ENSG00000140545	MFGE8	protein_coding	3.52	3.5	0	2.85	2.8	0	8.91	8.9	0

ENSG00000147324	MFHAS1	protein_coding	1.99	2	0	2.02	2	0	NS	NS	-
ENSG00000168389	MFSD2	protein_coding	2.32	2.3	0	NS	NS	-	2.31	2.3	0
ENSG00000182544	MFSD5	protein_coding	NS	NS	-	NS	NS	-	5.75	5.7	0
ENSG00000071073	MGAT4A	protein_coding	NS	NS	-	NS	NS	-	0.04	-26.8	0
ENSG00000161013	MGAT4B	protein_coding	2.4	2.4	0	2.17	2.2	0	4.2	4.2	0
ENSG00000074416	MGLL	protein_coding	4.06	4.1	0	4.12	4.1	0	3.62	3.6	0
ENSG00000143198	MGST3	protein_coding	2.6	2.6	0	2.33	2.3	0	4.76	4.8	0
ENSG00000135596	MICAL1	protein_coding	2.3	2.3	0	2.15	2.2	0	3.49	3.5	0
ENSG00000133816	MICAL2	protein_coding	5.02	5	0	5.17	5.2	0	NS	NS	-
ENSG00000204516	MICB	protein_coding	NS	NS	-	NS	NS	-	3.18	3.2	0
ENSG00000101871	MID1	protein_coding	2	2	0	NS	NS	-	3.15	3.2	0
ENSG00000215417	MIRHG1	processed_transcript	0.28	-3.6	0	0.3	-3.4	0	NS	NS	-
ENSG00000148773	MKI67	protein_coding	4.13	4.1	0	4.37	4.4	0	NS	NS	-
ENSG00000079277	MKNK1	protein_coding	0.3	-3.4	0	0.3	-3.3	0	0.26	-3.8	0
ENSG00000099875	MKNK2	protein_coding	NS	NS	-	NS	NS	-	0.5	-2	0
ENSG00000150051	MKX	protein_coding	NS	NS	-	NS	NS	-	7.91	7.9	0
ENSG00000130382	MLLT1	protein_coding	NS	NS	-	NS	NS	-	2.1	2.1	0
ENSG00000171843	MLLT3	protein_coding	2.28	2.3	0	2.36	2.4	0	NS	NS	-
ENSG00000103150	MLYCD	protein_coding	0.48	-2.1	0	0.49	-2	0	0.39	-2.5	0
ENSG00000196549	MME	protein_coding	NS	NS	-	NS	NS	-	65.87	65.9	0
ENSG00000196611	MMP1	protein_coding	NS	NS	-	NS	NS	-	42.85	42.9	0
ENSG00000099953	MMP11	protein_coding	14.67	14.7	0	15.73	15.7	0	6.2	6.2	0
ENSG00000157227	MMP14	protein_coding	6.61	6.6	0	5.96	6	0	11.84	11.8	0
ENSG00000087245	MMP2	protein_coding	7.46	7.5	0	6.11	6.1	0	18.24	18.2	0
ENSG00000149968	MMP3	protein_coding	NS	NS	-	NS	NS	-	66.31	66.3	0
ENSG00000137673	MMP7	protein_coding	NS	NS	-	2.95	3	0	0.03	-37.3	0
ENSG00000138722	MMRN1	protein_coding	NS	NS	-	NS	NS	-	0.4	-2.5	0
ENSG00000114978	MOBKL1B	protein_coding	2.64	2.6	0	2.66	2.7	0	NS	NS	-
ENSG00000172081	MOBKL2A	protein_coding	NS	NS	-	NS	NS	-	2.16	2.2	0
ENSG00000101928	MOSPD1	protein_coding	NS	NS	-	NS	NS	-	3.11	3.1	0
ENSG00000079931	MOXD1	protein_coding	8.05	8	0	8.13	8.1	0	7.34	7.3	0
ENSG00000135698	MPHOSPH6	protein_coding	2.14	2.1	0	2.13	2.1	0	NS	NS	-
ENSG00000105926	MPP6	protein_coding	0.46	-2.2	0	0.47	-2.1	0	NS	NS	-
ENSG00000150054	MPP7	protein_coding	0.43	-2.3	0	0.46	-2.2	0	0.14	-7.2	0
ENSG00000115204	MPV17	protein_coding	NS	NS	-	NS	NS	-	3.74	3.7	0
ENSG00000156968	MPV17L	protein_coding	0.23	-4.4	0	0.23	-4.3	0	NS	NS	-
ENSG00000197965	MPZL1	protein_coding	2.6	2.6	0	2.47	2.5	0	3.69	3.7	0
ENSG00000149573	MPZL2	protein_coding	NS	NS	-	NS	NS	-	0.05	-18.3	0
ENSG00000160588	MPZL3	protein_coding	NS	NS	-	NS	NS	-	0.24	-4.2	0
ENSG00000153029	MR1	protein_coding	2.67	2.7	0	2.55	2.6	0	3.58	3.6	0
ENSG00000011028	MRC2	protein_coding	2.94	2.9	0	2.57	2.6	0	5.84	5.8	0
ENSG00000172935	MRGPRF	protein_coding	NS	NS	-	NS	NS	-	4.54	4.5	0

ENSG00000172172	MRPL13	protein_coding	NS	NS	-	NS	NS	-	2.04	2	0
ENSG00000180992	MRPL14	protein_coding	NS	NS	-	NS	NS	-	2.02	2	0
ENSG00000137547	MRPL15	protein_coding	NS	NS	-	NS	NS	-	2.6	2.6	0
ENSG00000128626	MRPS12	protein_coding	NS	NS	-	NS	NS	-	2.39	2.4	0
ENSG00000072952	MRVI1	protein_coding	2.48	2.5	0	2.53	2.5	0	NS	NS	-
ENSG00000110077	MS4A6A	protein_coding	NS	NS	-	2.88	2.9	0	NS	NS	-
ENSG00000166927	MS4A7	protein_coding	NS	NS	-	NS	NS	-	0.15	-6.8	0
ENSG00000178860	MSC	protein_coding	NS	NS	-	1.95	2	0	5.95	5.9	0
ENSG00000147065	MSN	protein_coding	3.44	3.4	0	3.42	3.4	0	3.55	3.6	0
ENSG00000148450	MSRB2	protein_coding	NS	NS	-	NS	NS	-	2.54	2.5	0
ENSG00000174099	MSRB3	protein_coding	2.69	2.7	0	2.49	2.5	0	4.29	4.3	0
ENSG00000173531	MST1	protein_coding	0.47	-2.1	0	0.48	-2.1	0	NS	NS	-
ENSG00000186715	MST1P9	pseudogene	0.46	-2.2	0	0.47	-2.1	0	NS	NS	-
ENSG00000198417	MT1F	protein_coding	0.34	-2.9	0	0.38	-2.7	0	0.08	-12.7	0
ENSG00000125144	MT1G	protein_coding	0.33	-3	0	0.34	-3	0	0.27	-3.8	0
ENSG00000205358	MT1H	protein_coding	0.11	-8.9	0	0.12	-8.6	0	0.08	-12.3	0
ENSG00000205364	MT1M	protein_coding	0.25	-4	0	0.27	-3.8	0	0.12	-8.6	0
ENSG00000187193	MT1X	protein_coding	0.45	-2.2	0	0.46	-2.2	0	NS	NS	-
ENSG00000014914	MTMR11	protein_coding	4.36	4.4	0	4.74	4.7	0	NS	NS	-
ENSG00000150712	MTMR12	protein_coding	0.46	-2.2	0	0.47	-2.1	0	NS	NS	-
ENSG00000132613	MTSS1L	protein_coding	NS	NS	-	NS	NS	-	6.81	6.8	0
ENSG00000129422	MTUS1	protein_coding	NS	NS	-	NS	NS	-	0.2	-4.9	0
ENSG00000173702	MUC13	protein_coding	NS	NS	-	13.39	13.4	0	NS	NS	-
ENSG00000169550	MUC15	protein_coding	0.2	-4.9	0	0.22	-4.6	0	NS	NS	-
ENSG00000169894	MUC3A	protein_coding	NS	NS	-	9.47	9.5	0	NS	NS	-
ENSG00000184956	MUC6	protein_coding	NS	NS	-	NS	NS	-	0.05	-19.1	0
ENSG00000157502	MUM1L1	protein_coding	0.21	-4.7	0	0.22	-4.5	0	NS	NS	-
ENSG00000013364	MVP	protein_coding	2.32	2.3	0	2.3	2.3	0	NS	NS	-
ENSG00000183486	MX2	protein_coding	NS	NS	-	3.08	3.1	0	NS	NS	-
ENSG00000059728	MXD1	protein_coding	NS	NS	-	2.72	2.7	0	NS	NS	-
ENSG00000101825	MXRA5	protein_coding	3.79	3.8	0	3.77	3.8	0	NS	NS	-
ENSG00000118513	MYB	protein_coding	NS	NS	-	2.26	2.3	0	NS	NS	-
ENSG00000136997	MYC	protein_coding	NS	NS	-	0.49	-2	0	NS	NS	-
ENSG00000116990	MYCL1	protein_coding	0.39	-2.5	0	0.38	-2.6	0	NS	NS	-
ENSG00000172936	MYD88	protein_coding	1.97	2	0	1.96	2	0	NS	NS	-
ENSG00000104177	MYEF2	protein_coding	0.42	-2.4	0	0.44	-2.3	0	0.21	-4.9	0
ENSG00000100345	MYH9	protein_coding	2	2	0	2.03	2	0	NS	NS	-
ENSG00000196465	MYL6B	protein_coding	NS	NS	-	NS	NS	-	2.22	2.2	0
ENSG00000229596	MYL8P	pseudogene	NS	NS	-	NS	NS	-	2.25	2.2	0
ENSG00000101335	MYL9	protein_coding	3.02	3	0	2.65	2.7	0	5.97	6	0
ENSG00000065534	MYLK	protein_coding	3.27	3.3	0	2.92	2.9	0	NS	NS	-
ENSG00000176658	MYO1D	protein_coding	2.54	2.5	0	2.35	2.4	0	4.09	4.1	0

ENSG00000157483	MYO1E	protein_coding	3.69	3.7	0	3.68	3.7	0	3.8	3.8	0
ENSG00000142347	MYO1F	protein_coding	NS	NS	-	2.27	2.3	0	NS	NS	-
ENSG00000167306	MYO5B	protein_coding	NS	NS	-	NS	NS	-	0.19	-5.4	0
ENSG00000128833	MYO5C	protein_coding	NS	NS	-	NS	NS	-	0.05	-21.6	0
ENSG00000099331	MYO9B	protein_coding	2.11	2.1	0	2.11	2.1	0	NS	NS	-
ENSG00000138119	MYOF	protein_coding	4.85	4.8	0	4.59	4.6	0	NS	NS	-
ENSG00000101605	MYOM1	protein_coding	NS	NS	-	NS	NS	-	0.13	-7.6	0
ENSG00000170011	MYRIP	protein_coding	0.34	-2.9	0	0.33	-3	0	NS	NS	-
ENSG00000160877	NACC1	protein_coding	NS	NS	-	NS	NS	-	2.06	2.1	0
ENSG00000198951	NAGA	protein_coding	2.3	2.3	0	2.28	2.3	0	2.44	2.4	0
ENSG00000172766	NARG1L	protein_coding	0.33	-3	0	0.35	-2.8	0	0.17	-5.8	0
ENSG00000067798	NAV3	protein_coding	NS	NS	-	NS	NS	-	7.95	7.9	0
ENSG00000158747	NBL1	protein_coding	2.01	2	0	2.04	2	0	NS	NS	-
ENSG00000146918	NCAPG2	protein_coding	2.1	2.1	0	2.13	2.1	0	NS	NS	-
ENSG00000020129	NCDN	protein_coding	NS	NS	-	NS	NS	-	2.33	2.3	0
ENSG00000116701	NCF2	protein_coding	NS	NS	-	2.92	2.9	0	0.31	-3.2	0
ENSG00000100365	NCF4	protein_coding	NS	NS	-	2.41	2.4	0	NS	NS	-
ENSG00000123338	NCKAP1L	protein_coding	NS	NS	-	4.3	4.3	0	NS	NS	-
ENSG00000080986	NDC80	protein_coding	2.22	2.2	0	2.3	2.3	0	NS	NS	-
ENSG00000072864	NDE1	protein_coding	2.11	2.1	0	2.13	2.1	0	NS	NS	-
ENSG00000182636	NDN	protein_coding	NS	NS	-	NS	NS	-	1.97	2	0
ENSG00000165795	NDRG2	protein_coding	NS	NS	-	NS	NS	-	0.14	-7.4	0
ENSG00000070614	NDST1	protein_coding	NS	NS	-	NS	NS	-	2.74	2.7	0
ENSG00000174886	NDUFA11	protein_coding	NS	NS	-	NS	NS	-	2.03	2	0
ENSG00000164258	NDUFS4	protein_coding	NS	NS	-	NS	NS	-	2.88	2.9	0
ENSG00000078114	NEBL	protein_coding	NS	NS	-	NS	NS	-	0.19	-5.4	0
ENSG00000157191	NECAP2	protein_coding	2.59	2.6	0	2.51	2.5	0	3.24	3.2	0
ENSG00000069869	NEDD4	protein_coding	2.23	2.2	0	2.09	2.1	0	3.41	3.4	0
ENSG00000049759	NEDD4L	protein_coding	0.39	-2.6	0	0.38	-2.6	0	NS	NS	-
ENSG00000129559	NEDD8	protein_coding	NS	NS	-	NS	NS	-	3.51	3.5	0
ENSG00000163491	NEK10	protein_coding	NS	NS	-	NS	NS	-	4.08	4.1	0
ENSG00000173848	NET1	protein_coding	NS	NS	-	2.11	2.1	0	NS	NS	-
ENSG00000107954	NEURL	protein_coding	0.41	-2.4	0	0.41	-2.4	0	NS	NS	-
ENSG00000186575	NF2	protein_coding	NS	NS	-	NS	NS	-	2.62	2.6	0
ENSG00000100968	NFATC4	protein_coding	NS	NS	-	NS	NS	-	3.11	3.1	0
ENSG00000050344	NFE2L3	protein_coding	NS	NS	-	3.12	3.1	0	NS	NS	-
ENSG00000008441	NFIX	protein_coding	NS	NS	-	NS	NS	-	4.29	4.3	0
ENSG00000188158	NHS	protein_coding	2	2	0	2.04	2	0	NS	NS	-
ENSG00000116962	NID1	protein_coding	2.78	2.8	0	2.49	2.5	0	5.1	5.1	0
ENSG00000087303	NID2	protein_coding	3.38	3.4	0	3.06	3.1	0	5.97	6	0
ENSG00000197885	NKIRAS1	protein_coding	NS	NS	-	NS	NS	-	4.79	4.8	0
ENSG00000169992	NLGN2	protein_coding	NS	NS	-	NS	NS	-	2.18	2.2	0

ENSG00000140853	NLRC5	protein_coding	1.96	2	0	2.03	2	0	NS	NS	-
ENSG00000123609	NMI	protein_coding	3.46	3.5	0	3.6	3.6	0	NS	NS	-
ENSG00000163864	NMNAT3	protein_coding	0.49	-2.1	0	0.51	-2	0	0.33	-3.1	0
ENSG00000166741	NNMT	protein_coding	3.51	3.5	0	3.13	3.1	0	6.59	6.6	0
ENSG00000106100	NOD1	protein_coding	2.54	2.5	0	2.59	2.6	0	NS	NS	-
ENSG00000182117	NOP10	protein_coding	2.38	2.4	0	2.15	2.1	0	4.23	4.2	0
ENSG00000089250	NOS1	protein_coding	0.41	-2.5	0	0.4	-2.5	0	NS	NS	-
ENSG00000163072	NOSTRIN	protein_coding	NS	NS	-	NS	NS	-	0.06	-15.7	0
ENSG00000074181	NOTCH3	protein_coding	NS	NS	-	1.96	2	0	NS	NS	-
ENSG00000136999	NOV	protein_coding	NS	NS	-	NS	NS	-	5.43	5.4	0
ENSG00000086991	NOX4	protein_coding	8.79	8.8	0	9.54	9.5	0	NS	NS	-
ENSG00000198805	NP	protein_coding	2.19	2.2	0	2.26	2.3	0	NS	NS	-
ENSG00000141458	NPC1	protein_coding	NS	NS	-	NS	NS	-	5.31	5.3	0
ENSG00000119655	NPC2	protein_coding	2.47	2.5	0	2.3	2.3	0	3.84	3.8	0
ENSG00000135838	NPL	protein_coding	NS	NS	-	2.73	2.7	0	NS	NS	-
ENSG00000241935	NPL2	protein_coding	0.38	-2.6	0	0.38	-2.7	0	0.44	-2.2	0
ENSG00000168743	NPNT	protein_coding	NS	NS	-	NS	NS	-	0.16	-6.4	0
ENSG00000159899	NPR2	protein_coding	NS	NS	-	NS	NS	-	3.86	3.9	0
ENSG00000164128	NPY1R	protein_coding	0.27	-3.7	0	NS	NS	-	0.08	-12.2	0
ENSG00000181019	NQO1	protein_coding	8.1	8.1	0	7.75	7.8	0	NS	NS	-
ENSG00000151623	NR3C2	protein_coding	NS	NS	-	NS	NS	-	0.27	-3.7	0
ENSG00000116833	NR5A2	protein_coding	0.14	-7.4	0	0.15	-6.6	0	0.01	-98.2	0
ENSG00000148572	NRBF2	protein_coding	1.99	2	0	NS	NS	-	3.01	3	0
ENSG00000091129	NRCAM	protein_coding	0.23	-4.3	0	0.25	-4.1	0	0.11	-8.7	0
ENSG00000169752	NRG4	protein_coding	0.09	-10.7	0	0.1	-10	0	NS	NS	-
ENSG00000175352	NRIP3	protein_coding	NS	NS	-	NS	NS	-	2.5	2.5	0
ENSG00000124785	NRN1	protein_coding	NS	NS	-	NS	NS	-	13.18	13.2	0
ENSG00000118257	NRP2	protein_coding	5.14	5.1	0	5.22	5.2	0	4.54	4.5	0
ENSG00000147383	NSDHL	protein_coding	NS	NS	-	NS	NS	-	3.51	3.5	0
ENSG00000241058	NSUN6	protein_coding	0.5	-2	0	NS	NS	-	0.27	-3.7	0
ENSG00000179299	NSUN7	protein_coding	0.39	-2.5	0	0.42	-2.4	0	0.18	-5.5	0
ENSG00000116981	NT5C1A	protein_coding	0.49	-2	0	0.48	-2.1	0	NS	NS	-
ENSG00000168268	NT5DC2	protein_coding	2.44	2.4	0	2.23	2.2	0	4.07	4.1	0
ENSG00000135318	NT5E	protein_coding	6.71	6.7	0	5.59	5.6	0	15.65	15.7	0
ENSG00000157045	NTAN1	protein_coding	NS	NS	-	NS	NS	-	4.68	4.7	0
ENSG00000185652	NTF3	protein_coding	NS	NS	-	NS	NS	-	2.91	2.9	0
ENSG00000074527	NTN4	protein_coding	0.48	-2.1	0	0.49	-2	0	NS	NS	-
ENSG00000074590	NUAK1	protein_coding	3.53	3.5	0	3.53	3.5	0	NS	NS	-
ENSG00000163545	NUAK2	protein_coding	0.5	-2	0	0.48	-2.1	0	NS	NS	-
ENSG00000070081	NUCB2	protein_coding	0.23	-4.3	0	0.23	-4.3	0	NS	NS	-
ENSG00000132182	NUP210	protein_coding	NS	NS	-	NS	NS	-	0.33	-3	0
ENSG00000213024	NUP62	protein_coding	2.4	2.4	0	2.38	2.4	0	NS	NS	-

ENSG00000137804	NUSAP1	protein_coding	2.92	2.9	0	3.05	3	0	NS	NS	-
ENSG00000111335	OAS2	protein_coding	2.87	2.9	0	3.08	3.1	0	NS	NS	-
ENSG00000173559	OBFC2A	protein_coding	2.24	2.2	0	2.03	2	0	NS	NS	-
ENSG00000145247	OCIAD2	protein_coding	NS	NS	-	2.92	2.9	0	NS	NS	-
ENSG00000197822	OCLN	protein_coding	NS	NS	-	NS	NS	-	0.05	-21.7	0
ENSG00000122417	ODF2L	protein_coding	2.22	2.2	0	2.24	2.2	0	NS	NS	-
ENSG00000145934	ODZ2	protein_coding	NS	NS	-	NS	NS	-	28.92	28.9	0
ENSG00000087263	OGFOD1	protein_coding	NS	NS	-	NS	NS	-	2.89	2.9	0
ENSG00000162745	OLFML2B	protein_coding	10.13	10.1	0	9.96	10	0	11.52	11.5	0
ENSG00000169856	ONECUT1	protein_coding	0.32	-3.1	0	0.33	-3	0	0.24	-4.2	0
ENSG00000182667	OPCML	protein_coding	3.05	3.1	0	3.29	3.3	0	NS	NS	-
ENSG00000123240	OPTN	protein_coding	NS	NS	-	NS	NS	-	2.62	2.6	0
ENSG00000234560	OR10G4	protein_coding	0.47	-2.1	0	0.47	-2.1	0	NS	NS	-
ENSG00000236981	OR10G9	protein_coding	0.51	-2	0	0.51	-2	0	NS	NS	-
ENSG00000171014	OR4D5	protein_coding	0.21	-4.8	0	0.21	-4.8	0	NS	NS	-
ENSG00000181518	OR8D4	protein_coding	0.2	-5.1	0	0.2	-5.1	0	NS	NS	-
ENSG00000160991	ORAI2	protein_coding	NS	NS	-	NS	NS	-	2.83	2.8	0
ENSG00000123353	ORMDL2	protein_coding	2.16	2.2	0	2.03	2	0	NS	NS	-
ENSG00000144645	OSBPL10	protein_coding	5.08	5.1	0	5.49	5.5	0	NS	NS	-
ENSG00000070882	OSBPL3	protein_coding	5.28	5.3	0	5.17	5.2	0	6.15	6.2	0
ENSG00000091039	OSBPL8	protein_coding	1.98	2	0	NS	NS	-	2.78	2.8	0
ENSG00000145623	OSMR	protein_coding	2.33	2.3	0	2.27	2.3	0	NS	NS	-
ENSG00000143867	OSR1	protein_coding	NS	NS	-	NS	NS	-	8.33	8.3	0
ENSG00000164920	OSR2	protein_coding	NS	NS	-	NS	NS	-	8.2	8.2	0
ENSG00000134996	OSTF1	protein_coding	2.19	2.2	0	2.25	2.3	0	NS	NS	-
ENSG00000036473	OTC	protein_coding	0.36	-2.8	0	0.36	-2.8	0	NS	NS	-
ENSG00000083720	OXCT1	protein_coding	2.5	2.5	0	2.47	2.5	0	2.69	2.7	0
ENSG00000164830	OXR1	protein_coding	NS	NS	-	2.02	2	0	NS	NS	-
ENSG00000108405	P2RX1	protein_coding	0.14	-7.1	0	0.15	-6.8	0	0.1	-10.1	0
ENSG00000122884	P4HA1	protein_coding	3.72	3.7	0	3.61	3.6	0	4.6	4.6	0
ENSG00000149380	P4HA3	protein_coding	NS	NS	-	NS	NS	-	2.81	2.8	0
ENSG00000090621	PABPC4	protein_coding	NS	NS	-	0.51	-2	0	NS	NS	-
ENSG00000175115	PACS1	protein_coding	NS	NS	-	NS	NS	-	2.38	2.4	0
ENSG00000100266	PACSIN2	protein_coding	0.51	-2	0	NS	NS	-	0.3	-3.3	0
ENSG00000124374	PAIP2B	protein_coding	0.08	-12.7	0	0.09	-11.7	0	0.03	-35.3	0
ENSG00000149269	PAK1	protein_coding	2.49	2.5	0	2.61	2.6	0	NS	NS	-
ENSG00000180370	PAK2	protein_coding	2.01	2	0	1.98	2	0	NS	NS	-
ENSG00000077264	PAK3	protein_coding	0.19	-5.4	0	0.2	-5	0	0.07	-15.3	0
ENSG00000129116	PALLD	protein_coding	3.49	3.5	0	3.55	3.6	0	NS	NS	-
ENSG00000135473	PAN2	protein_coding	NS	NS	-	NS	NS	-	0.33	-3	0
ENSG00000182752	PAPPA	protein_coding	NS	NS	-	NS	NS	-	18.11	18.1	0
ENSG00000170915	PAQR8	protein_coding	2.42	2.4	0	2.59	2.6	0	NS	NS	-

ENSG00000102981	PARD6A	protein_coding	0.44	-2.3	0	0.45	-2.2	0	NS	NS	-
ENSG00000124171	PARD6B	protein_coding	NS	NS	-	NS	NS	-	0.35	-2.9	0
ENSG00000169116	PARM1	protein_coding	NS	NS	-	NS	NS	-	0.08	-13.2	0
ENSG00000173193	PARP14	protein_coding	2.59	2.6	0	2.8	2.8	0	NS	NS	-
ENSG00000173200	PARP15	protein_coding	4.12	4.1	0	4.49	4.5	0	NS	NS	-
ENSG00000041880	PARP3	protein_coding	3.07	3.1	0	2.84	2.8	0	4.93	4.9	0
ENSG00000102699	PARP4	protein_coding	2.11	2.1	0	2.2	2.2	0	NS	NS	-
ENSG00000151883	PARP8	protein_coding	NS	NS	-	2.02	2	0	NS	NS	-
ENSG00000138496	PARP9	protein_coding	2.22	2.2	0	2.37	2.4	0	NS	NS	-
ENSG00000138964	PARVG	protein_coding	NS	NS	-	2.42	2.4	0	NS	NS	-
ENSG00000108187	PBLD	protein_coding	0.29	-3.5	0	0.3	-3.3	0	0.17	-6	0
ENSG00000183570	PCBP3	protein_coding	NS	NS	-	NS	NS	-	2.33	2.3	0
ENSG00000090097	PCBP4	protein_coding	NS	NS	-	NS	NS	-	2.02	2	0
ENSG00000118946	PCDH17	protein_coding	NS	NS	-	NS	NS	-	0.32	-3.2	0
ENSG00000189184	PCDH18	protein_coding	4.78	4.8	0	3.44	3.4	0	15.47	15.5	0
ENSG00000169851	PCDH7	protein_coding	2.72	2.7	0	2.89	2.9	0	NS	NS	-
ENSG00000239389	PCDHA13	protein_coding	0.49	-2	0	0.49	-2.1	0	NS	NS	-
ENSG00000204965	PCDHA5	protein_coding	0.45	-2.2	0	0.46	-2.2	0	NS	NS	-
ENSG00000146001	PCDHB18	pseudogene	NS	NS	-	NS	NS	-	0.26	-3.8	0
ENSG00000081818	PCDHB4	protein_coding	NS	NS	-	NS	NS	-	0.29	-3.5	0
ENSG00000240184	PCDHGC3	protein_coding	NS	NS	-	NS	NS	-	3.79	3.8	0
ENSG00000115289	PCGF1	protein_coding	NS	NS	-	NS	NS	-	2.16	2.2	0
ENSG00000106333	PCOLCE	protein_coding	NS	NS	-	NS	NS	-	5.41	5.4	0
ENSG00000102225	PCTK1	protein_coding	NS	NS	-	NS	NS	-	2.31	2.3	0
ENSG00000161217	PCYT1A	protein_coding	NS	NS	-	NS	NS	-	2.46	2.5	0
ENSG00000102230	PCYT1B	protein_coding	0.48	-2.1	0	0.48	-2.1	0	0.47	-2.1	0
ENSG00000197646	PDCD1LG2	protein_coding	NS	NS	-	NS	NS	-	8.63	8.6	0
ENSG00000150593	PDCD4	protein_coding	0.23	-4.3	0	0.25	-4	0	0.11	-9	0
ENSG00000115252	PDE1A	protein_coding	NS	NS	-	2.45	2.5	0	NS	NS	-
ENSG00000152270	PDE3B	protein_coding	NS	NS	-	NS	NS	-	0.18	-5.6	0
ENSG00000113231	PDE8B	protein_coding	0.32	-3.1	0	0.34	-2.9	0	NS	NS	-
ENSG00000145431	PDGFC	protein_coding	2.62	2.6	0	2.74	2.7	0	NS	NS	-
ENSG00000113721	PDGFRB	protein_coding	4.27	4.3	0	3.99	4	0	NS	NS	-
ENSG00000185615	PDIA2	protein_coding	0.04	-23.4	0	0.05	-22.1	0	0.02	-43.3	0
ENSG00000005882	PDK2	protein_coding	NS	NS	-	0.51	-2	0	NS	NS	-
ENSG00000004799	PDK4	protein_coding	0.16	-6.3	0	0.18	-5.7	0	0.02	-50.8	0
ENSG00000131435	PDLIM4	protein_coding	NS	NS	-	NS	NS	-	3.04	3	0
ENSG00000163110	PDLIM5	protein_coding	NS	NS	-	1.98	2	0	NS	NS	-
ENSG00000196923	PDLIM7	protein_coding	3.54	3.5	0	3.42	3.4	0	4.49	4.5	0
ENSG00000162493	PDPN	protein_coding	4.31	4.3	0	3.99	4	0	6.85	6.8	0
ENSG00000174827	PDZK1	protein_coding	0.22	-4.6	0	0.23	-4.4	0	NS	NS	-
ENSG00000162366	PDZK1IP1	protein_coding	NS	NS	-	NS	NS	-	0.2	-5	0

ENSG00000162734	PEA15	protein_coding	2.31	2.3	0	1.99	2	0	4.83	4.8	0
ENSG00000134020	PEBP4	protein_coding	0.33	-3	0	0.32	-3.1	0	NS	NS	-
ENSG00000198721	PECI	protein_coding	0.4	-2.5	0	0.38	-2.7	0	NS	NS	-
ENSG00000197329	PELI1	protein_coding	NS	NS	-	2.39	2.4	0	NS	NS	-
ENSG00000139946	PELI2	protein_coding	0.44	-2.3	0	0.46	-2.2	0	0.27	-3.8	0
ENSG00000179094	PER1	protein_coding	0.44	-2.3	0	0.43	-2.3	0	NS	NS	-
ENSG00000049246	PER3	protein_coding	NS	NS	-	NS	NS	-	0.33	-3	0
ENSG00000114757	PEX5L	protein_coding	0.2	-5.1	0	0.19	-5.2	0	NS	NS	-
ENSG00000067057	PFKP	protein_coding	2.61	2.6	0	2.64	2.6	0	NS	NS	-
ENSG00000229183	PGA4	protein_coding	0.45	-2.2	0	0.43	-2.3	0	NS	NS	-
ENSG00000171314	PGAM1	protein_coding	NS	NS	-	NS	NS	-	2.76	2.8	0
ENSG00000177614	PGBD5	protein_coding	0.42	-2.4	0	0.43	-2.3	0	0.31	-3.2	0
ENSG00000102144	PGK1	protein_coding	2.12	2.1	0	2.13	2.1	0	NS	NS	-
ENSG00000130313	PGLS	protein_coding	NS	NS	-	NS	NS	-	2.19	2.2	0
ENSG00000169299	PGM2	protein_coding	2.61	2.6	0	2.71	2.7	0	NS	NS	-
ENSG00000165434	PGM2L1	protein_coding	NS	NS	-	2.2	2.2	0	NS	NS	-
ENSG00000112419	PHACTR2	protein_coding	2.47	2.5	0	2.46	2.5	0	NS	NS	-
ENSG00000077684	PHF17	protein_coding	0.27	-3.7	0	0.28	-3.6	0	NS	NS	-
ENSG00000040633	PHF23	protein_coding	NS	NS	-	NS	NS	-	2.73	2.7	0
ENSG00000118482	PHF3	protein_coding	NS	NS	-	NS	NS	-	0.51	-2	0
ENSG00000092621	PHGDH	protein_coding	0.28	-3.6	0	0.23	-4.4	0	NS	NS	-
ENSG00000044446	PHKA2	protein_coding	NS	NS	-	NS	NS	-	0.33	-3.1	0
ENSG00000006576	PHTF2	protein_coding	1.96	2	0	2	2	0	NS	NS	-
ENSG00000175287	PHYHD1	protein_coding	0.43	-2.3	0	0.41	-2.4	0	NS	NS	-
ENSG00000165443	PHYHIP	protein_coding	0.16	-6.3	0	0.17	-6.1	0	NS	NS	-
ENSG00000131788	PIAS3	protein_coding	NS	NS	-	NS	NS	-	2.38	2.4	0
ENSG00000162896	PIGR	protein_coding	NS	NS	-	NS	NS	-	0.14	-7.1	0
ENSG00000119227	PIGZ	protein_coding	NS	NS	-	NS	NS	-	2.27	2.3	0
ENSG00000155629	PIK3AP1	protein_coding	NS	NS	-	NS	NS	-	0.19	-5.4	0
ENSG00000133056	PIK3C2B	protein_coding	NS	NS	-	2.06	2.1	0	NS	NS	-
ENSG00000105851	PIK3CG	protein_coding	NS	NS	-	3.06	3.1	0	NS	NS	-
ENSG00000100100	PIK3IP1	protein_coding	2.83	2.8	0	2.92	2.9	0	NS	NS	-
ENSG00000105647	PIK3R2	protein_coding	NS	NS	-	NS	NS	-	2.71	2.7	0
ENSG00000137193	PIM1	protein_coding	NS	NS	-	NS	NS	-	2.81	2.8	0
ENSG00000186111	PIP5K1C	protein_coding	NS	NS	-	NS	NS	-	2.39	2.4	0
ENSG00000150867	PIP5K2A	protein_coding	2.72	2.7	0	2.75	2.8	0	NS	NS	-
ENSG00000170927	PKHD1	protein_coding	0.22	-4.6	0	0.24	-4.2	0	0.04	-23.5	0
ENSG00000067225	PKM2	protein_coding	4.28	4.3	0	4.04	4	0	6.13	6.1	0
ENSG00000057294	PKP2	protein_coding	NS	NS	-	NS	NS	-	0.14	-6.9	0
ENSG00000069764	PLA2G10	protein_coding	NS	NS	-	7.83	7.8	0	NS	NS	-
ENSG00000103066	PLA2G15	protein_coding	NS	NS	-	NS	NS	-	4.97	5	0
ENSG00000176485	PLA2G16	protein_coding	NS	NS	-	2.55	2.6	0	NS	NS	-

ENSG00000170890	PLA2G1B	protein_coding	0.14	-7.2	0	0.16	-6.4	0	0.01	-132	0
ENSG00000146070	PLA2G7	protein_coding	NS	NS	-	5.81	5.8	0	NS	NS	-
ENSG00000153246	PLA2R1	protein_coding	5.57	5.6	0	5.48	5.5	0	6.3	6.3	0
ENSG00000145287	PLAC8	protein_coding	15.7	15.7	0	17.59	17.6	0	NS	NS	-
ENSG00000181690	PLAG1	protein_coding	NS	NS	-	NS	NS	-	2.79	2.8	0
ENSG00000104368	PLAT	protein_coding	5.12	5.1	0	5.65	5.6	0	NS	NS	-
ENSG00000122861	PLAU	protein_coding	5.92	5.9	0	6.33	6.3	0	NS	NS	-
ENSG00000011422	PLAUR	protein_coding	4.15	4.2	0	4.09	4.1	0	4.65	4.6	0
ENSG00000151176	PLBD2	protein_coding	NS	NS	-	NS	NS	-	3.18	3.2	0
ENSG00000182621	PLCB1	protein_coding	0.39	-2.6	0	0.42	-2.4	0	0.13	-7.5	0
ENSG00000137841	PLCB2	protein_coding	NS	NS	-	2.45	2.4	0	NS	NS	-
ENSG00000149782	PLCB3	protein_coding	NS	NS	-	NS	NS	-	2.31	2.3	0
ENSG00000187091	PLCD1	protein_coding	NS	NS	-	NS	NS	-	2.41	2.4	0
ENSG00000161714	PLCD3	protein_coding	2.71	2.7	0	2.37	2.4	0	5.4	5.4	0
ENSG00000180287	PLD5	protein_coding	0.51	-2	0	0.51	-2	0	NS	NS	-
ENSG00000178209	PLEC1	protein_coding	2.42	2.4	0	2.47	2.5	0	NS	NS	-
ENSG00000100558	PLEK2	protein_coding	NS	NS	-	4.44	4.4	0	NS	NS	-
ENSG00000143850	PLEKHA6	protein_coding	NS	NS	-	NS	NS	-	0.51	-2	0
ENSG00000166689	PLEKHA7	protein_coding	NS	NS	-	NS	NS	-	0.26	-3.9	0
ENSG00000073712	PLEKHC1	protein_coding	NS	NS	-	NS	NS	-	2.66	2.7	0
ENSG00000120278	PLEKHG1	protein_coding	3.21	3.2	0	3.39	3.4	0	NS	NS	-
ENSG00000090924	PLEKHG2	protein_coding	NS	NS	-	NS	NS	-	2.22	2.2	0
ENSG00000054690	PLEKHH1	protein_coding	0.45	-2.2	0	0.47	-2.1	0	0.23	-4.3	0
ENSG00000068137	PLEKHH3	protein_coding	0.44	-2.2	0	0.44	-2.3	0	NS	NS	-
ENSG00000225190	PLEKHM1	protein_coding	NS	NS	-	NS	NS	-	3.55	3.6	0
ENSG00000116786	PLEKHM2	protein_coding	NS	NS	-	NS	NS	-	2.82	2.8	0
ENSG00000023902	PLEKHO1	protein_coding	NS	NS	-	NS	NS	-	2.29	2.3	0
ENSG00000105355	PLIN3	protein_coding	2.04	2	0	NS	NS	-	4.45	4.4	0
ENSG00000102934	PLLP	protein_coding	NS	NS	-	NS	NS	-	0.13	-7.8	0
ENSG00000083444	PLOD1	protein_coding	NS	NS	-	NS	NS	-	4.01	4	0
ENSG00000152952	PLOD2	protein_coding	3.12	3.1	0	3.14	3.1	0	NS	NS	-
ENSG00000106397	PLOD3	protein_coding	NS	NS	-	NS	NS	-	3.23	3.2	0
ENSG00000120756	PLS1	protein_coding	NS	NS	-	3.4	3.4	0	0.22	-4.6	0
ENSG00000102024	PLS3	protein_coding	2.71	2.7	0	2.63	2.6	0	3.35	3.4	0
ENSG00000187838	PLSCR3	protein_coding	2.05	2	0	NS	NS	-	3.97	4	0
ENSG00000130300	PLVAP	protein_coding	NS	NS	-	NS	NS	-	0.28	-3.6	0
ENSG00000161381	PLXDC1	protein_coding	2.31	2.3	0	2.41	2.4	0	NS	NS	-
ENSG00000120594	PLXDC2	protein_coding	3.18	3.2	0	3.36	3.4	0	NS	NS	-
ENSG00000114554	PLXNA1	protein_coding	NS	NS	-	NS	NS	-	3.78	3.8	0
ENSG00000076356	PLXNA2	protein_coding	0.32	-3.1	0	0.34	-2.9	0	0.13	-7.5	0
ENSG00000130827	PLXNA3	protein_coding	NS	NS	-	NS	NS	-	2.64	2.6	0
ENSG00000136040	PLXNC1	protein_coding	4.34	4.3	0	4.35	4.4	0	NS	NS	-

ENSG00000004399	PLXND1	protein_coding	2.3	2.3	0	2.08	2.1	0	3.99	4	0
ENSG00000162877	PM20D1	protein_coding	0.05	-18.6	0	0.06	-17.8	0	NS	NS	-
ENSG00000146281	PM20D2	protein_coding	NS	NS	-	NS	NS	-	0.4	-2.5	0
ENSG00000118557	PMFBP1	protein_coding	0.44	-2.3	0	0.45	-2.2	0	NS	NS	-
ENSG00000140464	PML	protein_coding	2.23	2.2	0	2.2	2.2	0	NS	NS	-
ENSG00000109099	PMP22	protein_coding	3.26	3.3	0	2.78	2.8	0	7.11	7.1	0
ENSG00000175535	PNLIP	protein_coding	0.1	-10.4	0	0.11	-9.2	0	0	-527.8	0
ENSG00000187021	PNLIPRP1	protein_coding	0.06	-16.8	0	0.07	-15.1	0	0	-236.4	0
ENSG00000165862	PNLIPRP2	polymorphic_pseudogene	0.09	-11.4	0	0.1	-10.2	0	0	-230.5	0
ENSG00000032444	PNPLA6	protein_coding	NS	NS	-	NS	NS	-	2.24	2.2	0
ENSG00000174348	PODN	protein_coding	NS	NS	-	NS	NS	-	5.92	5.9	0
ENSG00000132000	PODNL1	protein_coding	2.74	2.7	0	2.3	2.3	0	6.25	6.2	0
ENSG00000124429	POF1B	protein_coding	NS	NS	-	3.39	3.4	0	0.2	-4.9	0
ENSG00000099817	POLR2E	protein_coding	NS	NS	-	NS	NS	-	2.71	2.7	0
ENSG00000105854	PON2	protein_coding	2.22	2.2	0	2.31	2.3	0	NS	NS	-
ENSG00000132429	POPDC3	protein_coding	NS	NS	-	NS	NS	-	14.77	14.8	0
ENSG00000133110	POSTN	protein_coding	46.82	46.8	0	48.72	48.7	0	31.62	31.6	0
ENSG00000106536	POU6F2	protein_coding	0.47	-2.1	0	0.48	-2.1	0	NS	NS	-
ENSG00000162407	PPAP2B	protein_coding	NS	NS	-	NS	NS	-	3.12	3.1	0
ENSG00000203805	PPAPDC1A	protein_coding	5.02	5	0	5.19	5.2	0	3.68	3.7	0
ENSG00000147535	PPAPDC1B	protein_coding	0.4	-2.5	0	0.41	-2.4	0	NS	NS	-
ENSG00000112033	PPARD	protein_coding	NS	NS	-	NS	NS	-	2.25	2.3	0
ENSG00000132170	PPARG	protein_coding	3.01	3	0	3.14	3.1	0	NS	NS	-
ENSG00000139220	PPFIA2	protein_coding	NS	NS	-	2.59	2.6	0	NS	NS	-
ENSG00000196262	PPIA	protein_coding	NS	NS	-	NS	NS	-	2.24	2.2	0
ENSG00000168938	PPIC	protein_coding	2.56	2.6	0	2.64	2.6	0	NS	NS	-
ENSG00000088808	PPP1R13B	protein_coding	NS	NS	-	NS	NS	-	0.35	-2.8	0
ENSG00000087074	PPP1R15A	protein_coding	NS	NS	-	NS	NS	-	3.59	3.6	0
ENSG00000173281	PPP1R3B	protein_coding	NS	NS	-	NS	NS	-	2.13	2.1	0
ENSG00000158528	PPP1R9A	protein_coding	NS	NS	-	NS	NS	-	0.18	-5.5	0
ENSG00000137713	PPP2R1B	protein_coding	0.46	-2.2	0	0.47	-2.1	0	NS	NS	-
ENSG00000175470	PPP2R2D	protein_coding	0.26	-3.9	0	0.26	-3.8	0	NS	NS	-
ENSG00000198901	PRC1	protein_coding	2.48	2.5	0	2.49	2.5	0	NS	NS	-
ENSG00000057657	PRDM1	protein_coding	3.95	4	0	4.16	4.2	0	NS	NS	-
ENSG00000138738	PRDM5	protein_coding	0.48	-2.1	0	0.48	-2.1	0	NS	NS	-
ENSG00000117450	PRDX1	protein_coding	2.14	2.1	0	2.06	2.1	0	NS	NS	-
ENSG00000123131	PRDX4	protein_coding	0.29	-3.5	0	0.27	-3.7	0	NS	NS	-
ENSG00000124126	PREX1	protein_coding	NS	NS	-	2.02	2	0	NS	NS	-
ENSG00000005249	PRKAR2B	protein_coding	0.34	-2.9	0	0.37	-2.7	0	0.13	-8	0
ENSG00000170955	PRKCDBP	protein_coding	2.41	2.4	0	2.2	2.2	0	NS	NS	-
ENSG00000027075	PRKCH	protein_coding	NS	NS	-	NS	NS	-	0.25	-4	0
ENSG00000065675	PRKCQ	protein_coding	NS	NS	-	NS	NS	-	0.41	-2.5	0

ENSG00000067606	PRKCZ	protein_coding	NS	NS	-	NS	NS	-	0.43	-2.3	0
ENSG00000113494	PRLR	protein_coding	0.24	-4.2	0	0.19	-5.3	0	NS	NS	-
ENSG00000171867	PRNP	protein_coding	NS	NS	-	NS	NS	-	2.94	2.9	0
ENSG00000007062	PROM1	protein_coding	NS	NS	-	NS	NS	-	0.07	-13.6	0
ENSG00000184500	PROS1	protein_coding	2.61	2.6	0	2.57	2.6	0	NS	NS	-
ENSG00000117707	PROX1	protein_coding	0.21	-4.8	0	0.22	-4.5	0	0.09	-11.3	0
ENSG00000101911	PRPS2	protein_coding	0.5	-2	0	NS	NS	-	0.26	-3.8	0
ENSG00000068489	PRR11	protein_coding	2.76	2.8	0	2.79	2.8	0	NS	NS	-
ENSG00000167183	PRR15L	protein_coding	NS	NS	-	NS	NS	-	0.08	-12.8	0
ENSG00000184838	PRR16	protein_coding	NS	NS	-	NS	NS	-	6.27	6.3	0
ENSG00000135362	PRR5L	protein_coding	2.41	2.4	0	2.48	2.5	0	NS	NS	-
ENSG00000130962	PRRG1	protein_coding	3.39	3.4	0	3.26	3.3	0	4.4	4.4	0
ENSG00000116132	PRRX1	protein_coding	5.28	5.3	0	3.92	3.9	0	16.1	16.1	0
ENSG00000204983	PRSS1	protein_coding	0.16	-6.4	0	NS	NS	-	0	-241.8	0
ENSG00000010438	PRSS3	protein_coding	0.13	-7.9	0	0.14	-7.3	0	0.04	-24.3	0
ENSG00000052344	PRSS8	protein_coding	NS	NS	-	NS	NS	-	0.18	-5.7	0
ENSG00000106772	PRUNE2	protein_coding	NS	NS	-	NS	NS	-	6.69	6.7	0
ENSG00000135069	PSAT1	protein_coding	NS	NS	-	0.15	-6.7	0	NS	NS	-
ENSG00000221826	PSG3	protein_coding	NS	NS	-	NS	NS	-	28.5	28.5	0
ENSG00000204941	PSG5	protein_coding	NS	NS	-	NS	NS	-	62.55	62.6	0
ENSG00000170848	PSG6	protein_coding	NS	NS	-	NS	NS	-	2.66	2.7	0
ENSG00000108294	PSMB3	protein_coding	2.31	2.3	0	2.22	2.2	0	2.99	3	0
ENSG00000204264	PSMB8	protein_coding	2.51	2.5	0	2.6	2.6	0	NS	NS	-
ENSG00000240065	PSMB9	protein_coding	2.48	2.5	0	2.64	2.6	0	NS	NS	-
ENSG00000173692	PSMD1	protein_coding	2.23	2.2	0	2.2	2.2	0	2.42	2.4	0
ENSG00000175166	PSMD2	protein_coding	NS	NS	-	NS	NS	-	2.57	2.6	0
ENSG00000163636	PSMD6	protein_coding	0.33	-3	0	0.32	-3.2	0	NS	NS	-
ENSG00000110801	PSMD9	protein_coding	NS	NS	-	NS	NS	-	1.99	2	0
ENSG00000152229	PSTPIP2	protein_coding	2.55	2.6	0	2.6	2.6	0	NS	NS	-
ENSG00000185920	PTCH1	protein_coding	NS	NS	-	NS	NS	-	0.4	-2.5	0
ENSG00000168267	PTF1A	protein_coding	0.35	-2.9	0	0.35	-2.9	0	0.36	-2.8	0
ENSG00000171522	PTGER4	protein_coding	0.31	-3.2	0	0.33	-3	0	NS	NS	-
ENSG00000148344	PTGES	protein_coding	NS	NS	-	NS	NS	-	7.2	7.2	0
ENSG00000134247	PTGFRN	protein_coding	NS	NS	-	1.99	2	0	NS	NS	-
ENSG00000124212	PTGIS	protein_coding	7.09	7.1	0	6.79	6.8	0	NS	NS	-
ENSG00000095303	PTGS1	protein_coding	2.7	2.7	0	2.7	2.7	0	2.74	2.7	0
ENSG00000073756	PTGS2	protein_coding	NS	NS	-	NS	NS	-	10.72	10.7	0
ENSG00000112655	PTK7	protein_coding	2.38	2.4	0	2.23	2.2	0	3.55	3.5	0
ENSG00000159335	PTMS	protein_coding	NS	NS	-	NS	NS	-	3.43	3.4	0
ENSG00000165996	PTPLA	protein_coding	NS	NS	-	NS	NS	-	4.24	4.2	0
ENSG00000188921	PTPLAD2	protein_coding	3.59	3.6	0	3.26	3.3	0	6.22	6.2	0
ENSG00000127947	PTPN12	protein_coding	2.49	2.5	0	2.58	2.6	0	NS	NS	-

ENSG00000163629	PTPN13	protein_coding	NS	NS	-	NS	NS	-	2.64	2.6	0
ENSG00000152104	PTPN14	protein_coding	NS	NS	-	NS	NS	-	3.47	3.5	0
ENSG00000134242	PTPN22	protein_coding	NS	NS	-	2.87	2.9	0	NS	NS	-
ENSG00000070159	PTPN3	protein_coding	NS	NS	-	NS	NS	-	0.11	-9.4	0
ENSG00000111679	PTPN6	protein_coding	NS	NS	-	2.17	2.2	0	NS	NS	-
ENSG00000127329	PTPRB	protein_coding	NS	NS	-	NS	NS	-	0.31	-3.2	0
ENSG00000081237	PTPRC	protein_coding	NS	NS	-	4.1	4.1	0	NS	NS	-
ENSG00000132334	PTPRE	protein_coding	NS	NS	-	2.32	2.3	0	NS	NS	-
ENSG00000155093	PTPRN2	protein_coding	0.33	-3	0	0.35	-2.8	0	0.17	-5.8	0
ENSG00000177469	PTRF	protein_coding	NS	NS	-	NS	NS	-	5.34	5.3	0
ENSG00000183255	PTTG1IP	protein_coding	2.05	2	0	2.04	2	0	NS	NS	-
ENSG00000163661	PTX3	protein_coding	NS	NS	-	NS	NS	-	12.31	12.3	0
ENSG00000130508	PXDN	protein_coding	5.91	5.9	0	5.04	5	0	12.91	12.9	0
ENSG00000168297	PXK	protein_coding	NS	NS	-	NS	NS	-	4.65	4.6	0
ENSG00000100994	PYGB	protein_coding	2.47	2.5	0	2.54	2.5	0	NS	NS	-
ENSG00000100504	PYGL	protein_coding	2.65	2.6	0	NS	NS	-	5.18	5.2	0
ENSG00000171016	PYGO1	protein_coding	NS	NS	-	NS	NS	-	2.62	2.6	0
ENSG00000213339	QTRT1	protein_coding	NS	NS	-	NS	NS	-	0.48	-2.1	0
ENSG00000156675	RAB11FIP1	protein_coding	NS	NS	-	NS	NS	-	0.18	-5.5	0
ENSG00000131242	RAB11FIP4	protein_coding	NS	NS	-	NS	NS	-	0.49	-2	0
ENSG00000135631	RAB11FIP5	protein_coding	NS	NS	-	NS	NS	-	2.28	2.3	0
ENSG00000124839	RAB17	protein_coding	0.43	-2.3	0	0.45	-2.2	0	0.26	-3.9	0
ENSG00000112210	RAB23	protein_coding	5.54	5.5	0	4.56	4.6	0	13.4	13.4	0
ENSG00000132698	RAB25	protein_coding	NS	NS	-	NS	NS	-	0.18	-5.5	0
ENSG00000167964	RAB26	protein_coding	0.36	-2.8	0	0.37	-2.7	0	0.31	-3.3	0
ENSG00000168461	RAB31	protein_coding	4.06	4.1	0	4.06	4.1	0	4.07	4.1	0
ENSG00000118508	RAB32	protein_coding	NS	NS	-	NS	NS	-	4.34	4.3	0
ENSG00000134594	RAB33A	protein_coding	NS	NS	-	NS	NS	-	2.71	2.7	0
ENSG00000109113	RAB34	protein_coding	2.91	2.9	0	2.65	2.6	0	5.01	5	0
ENSG00000169213	RAB3B	protein_coding	NS	NS	-	NS	NS	-	8.82	8.8	0
ENSG00000105514	RAB3D	protein_coding	0.39	-2.6	0	0.39	-2.6	0	NS	NS	-
ENSG00000167994	RAB3IL1	protein_coding	NS	NS	-	NS	NS	-	2.66	2.7	0
ENSG00000111540	RAB5B	protein_coding	NS	NS	-	NS	NS	-	2.29	2.3	0
ENSG00000117280	RAB7L1	protein_coding	2.04	2	0	NS	NS	-	3.26	3.3	0
ENSG00000166128	RAB8B	protein_coding	2.38	2.4	0	2.31	2.3	0	2.92	2.9	0
ENSG00000183155	RABIF	protein_coding	NS	NS	-	NS	NS	-	2.54	2.5	0
ENSG00000136238	RAC1	protein_coding	2.28	2.3	0	2.31	2.3	0	2.01	2	0
ENSG00000161800	RACGAP1	protein_coding	3.55	3.6	0	3.52	3.5	0	3.85	3.8	0
ENSG00000164754	RAD21	protein_coding	1.96	2	0	2.02	2	0	NS	NS	-
ENSG00000179262	RAD23A	protein_coding	NS	NS	-	NS	NS	-	2.12	2.1	0
ENSG00000051180	RAD51	protein_coding	NS	NS	-	NS	NS	-	2.03	2	0
ENSG00000039560	RAI14	protein_coding	3.17	3.2	0	3.12	3.1	0	NS	NS	-

ENSG00000017797	RALBP1	protein_coding	2.18	2.2	0	2.14	2.1	0	NS	NS	-
ENSG00000132329	RAMP1	protein_coding	0.47	-2.1	0	0.47	-2.1	0	NS	NS	-
ENSG00000076864	RAP1GAP	protein_coding	0.5	-2	0	NS	NS	-	0.24	-4.2	0
ENSG00000132359	RAP1GAP2	protein_coding	0.4	-2.5	0	0.43	-2.3	0	0.17	-6	0
ENSG00000181467	RAP2B	protein_coding	2.71	2.7	0	2.81	2.8	0	NS	NS	-
ENSG00000136237	RAPGEF5	protein_coding	NS	NS	-	NS	NS	-	0.11	-8.7	0
ENSG00000173166	RAPH1	protein_coding	2.14	2.1	0	2.17	2.2	0	NS	NS	-
ENSG00000077092	RARB	protein_coding	NS	NS	-	2.47	2.5	0	NS	NS	-
ENSG00000172819	RARG	protein_coding	NS	NS	-	NS	NS	-	3.73	3.7	0
ENSG00000118849	RARRES1	protein_coding	4.27	4.3	0	4.66	4.7	0	NS	NS	-
ENSG00000106538	RARRES2	protein_coding	0.29	-3.5	0	0.29	-3.4	0	NS	NS	-
ENSG00000133321	RARRES3	protein_coding	4.67	4.7	0	5.06	5.1	0	NS	NS	-
ENSG00000185989	RASA3	protein_coding	1.96	2	0	NS	NS	-	2.16	2.2	0
ENSG00000075391	RASAL2	protein_coding	2.37	2.4	0	2.41	2.4	0	NS	NS	-
ENSG00000152689	RASGRP3	protein_coding	NS	NS	-	2.15	2.2	0	NS	NS	-
ENSG00000105538	RASIP1	protein_coding	0.48	-2.1	0	0.48	-2.1	0	NS	NS	-
ENSG00000101265	RASSF2	protein_coding	2.92	2.9	0	3.04	3	0	NS	NS	-
ENSG00000153179	RASSF3	protein_coding	2.18	2.2	0	2.24	2.2	0	NS	NS	-
ENSG00000123094	RASSF8	protein_coding	NS	NS	-	NS	NS	-	3.21	3.2	0
ENSG00000112183	RBM24	protein_coding	NS	NS	-	NS	NS	-	5.13	5.1	0
ENSG00000163694	RBM47	protein_coding	NS	NS	-	NS	NS	-	0.13	-7.7	0
ENSG00000153250	RBMS1	protein_coding	2.67	2.7	0	2.54	2.5	0	3.76	3.8	0
ENSG00000114115	RBP1	protein_coding	0.27	-3.7	0	0.3	-3.4	0	0.08	-12.5	0
ENSG00000124232	RBPJL	protein_coding	0.05	-21	0	0.05	-20.2	0	0.03	-31	0
ENSG00000049449	RCN1	protein_coding	2.96	3	0	2.5	2.5	0	6.56	6.6	0
ENSG00000142552	RCN3	protein_coding	2.88	2.9	0	2.39	2.4	0	6.76	6.8	0
ENSG00000122707	RECK	protein_coding	NS	NS	-	NS	NS	-	11.9	11.9	0
ENSG00000004700	RECQL	protein_coding	2.7	2.7	0	2.75	2.8	0	2.26	2.3	0
ENSG00000165476	REEP3	protein_coding	2.41	2.4	0	2.38	2.4	0	2.67	2.7	0
ENSG00000115386	REG1A	protein_coding	NS	NS	-	NS	NS	-	0.01	-187.4	0
ENSG00000172023	REG1B	protein_coding	NS	NS	-	NS	NS	-	0.01	-131.1	0
ENSG00000172016	REG3A	protein_coding	NS	NS	-	NS	NS	-	0.02	-59.5	0
ENSG00000169891	REPS2	protein_coding	NS	NS	-	NS	NS	-	0.18	-5.6	0
ENSG00000134533	RERG	protein_coding	NS	NS	-	NS	NS	-	0.16	-6.4	0
ENSG00000076043	REXO2	protein_coding	NS	NS	-	NS	NS	-	4.98	5	0
ENSG00000131378	RFTN1	protein_coding	3.22	3.2	0	3.13	3.1	0	3.98	4	0
ENSG00000242732	RGAG4	protein_coding	NS	NS	-	NS	NS	-	3.03	3	0
ENSG00000174136	RGMB	protein_coding	NS	NS	-	NS	NS	-	5.33	5.3	0
ENSG00000130988	RGN	protein_coding	0.19	-5.1	0	0.19	-5.2	0	0.21	-4.7	0
ENSG00000090104	RGS1	protein_coding	NS	NS	-	4.34	4.3	0	NS	NS	-
ENSG00000116741	RGS2	protein_coding	0.28	-3.6	0	0.29	-3.4	0	0.13	-8	0
ENSG00000143248	RGS5	protein_coding	NS	NS	-	NS	NS	-	0.03	-30.9	0

ENSG00000164292	RHOBTB3	protein_coding	0.35	-2.9	0	0.33	-3	0	NS	NS	-
ENSG00000155366	RHOC	protein_coding	2.22	2.2	0	2.13	2.1	0	2.89	2.9	0
ENSG00000139725	RHOF	protein_coding	NS	NS	-	3.29	3.3	0	NS	NS	-
ENSG00000177105	RHOG	protein_coding	NS	NS	-	NS	NS	-	2.77	2.8	0
ENSG00000116574	RHOU	protein_coding	NS	NS	-	NS	NS	-	0.19	-5.3	0
ENSG00000166405	RIC3	protein_coding	0.33	-3	0	0.35	-2.9	0	0.2	-5.1	0
ENSG00000177963	RIC8A	protein_coding	NS	NS	-	NS	NS	-	2.73	2.7	0
ENSG00000188026	RILPL1	protein_coding	NS	NS	-	NS	NS	-	2.51	2.5	0
ENSG00000129538	RNASE1	protein_coding	NS	NS	-	0.44	-2.3	0	0.02	-60.5	0
ENSG00000219200	RNASEK	protein_coding	NS	NS	-	NS	NS	-	3.36	3.4	0
ENSG00000135828	RNASEL	protein_coding	2	2	0	2.07	2.1	0	NS	NS	-
ENSG00000115963	RND3	protein_coding	NS	NS	-	NS	NS	-	13.54	13.5	0
ENSG00000123091	RNF11	protein_coding	NS	NS	-	NS	NS	-	2.86	2.9	0
ENSG00000121848	RNF115	protein_coding	NS	NS	-	NS	NS	-	2.31	2.3	0
ENSG00000145860	RNF145	protein_coding	2.06	2.1	0	2.14	2.1	0	NS	NS	-
ENSG00000116514	RNF19B	protein_coding	2.3	2.3	0	2.37	2.4	0	NS	NS	-
ENSG00000178222	RNF212	protein_coding	0.37	-2.7	0	0.35	-2.8	0	NS	NS	-
ENSG00000173821	RNF213	protein_coding	2.3	2.3	0	2.39	2.4	0	NS	NS	-
ENSG00000163481	RNF25	protein_coding	NS	NS	-	NS	NS	-	2.58	2.6	0
ENSG00000173456	RNF26	protein_coding	NS	NS	-	NS	NS	-	3.32	3.3	0
ENSG00000108375	RNF43	protein_coding	NS	NS	-	NS	NS	-	0.35	-2.9	0
ENSG00000023191	RNH1	protein_coding	NS	NS	-	NS	NS	-	3.37	3.4	0
ENSG00000169855	ROBO1	protein_coding	2.66	2.7	0	2.53	2.5	0	3.71	3.7	0
ENSG00000119314	ROD1	protein_coding	2.12	2.1	0	2.26	2.3	0	NS	NS	-
ENSG00000145491	ROPN1L	protein_coding	NS	NS	-	0.51	-2	0	NS	NS	-
ENSG00000169071	ROR2	protein_coding	2.34	2.3	0	2.29	2.3	0	NS	NS	-
ENSG00000143365	RORC	protein_coding	0.32	-3.1	0	0.33	-3	0	0.24	-4.1	0
ENSG00000229939	RP11-111F16.1	pseudogene	0.46	-2.2	0	0.44	-2.3	0	NS	NS	-
ENSG00000197067	RP11-132G10.1	pseudogene	0.48	-2.1	0	0.48	-2.1	0	NS	NS	-
ENSG00000232527	RP11-14N7.2	lincRNA	4.97	5	0	3.33	3.3	0	18.08	18.1	0
ENSG00000230358	RP11-166O4.2	pseudogene	0.43	-2.3	0	0.42	-2.4	0	NS	NS	-
ENSG00000229932	RP11-215C7.2	pseudogene	2.17	2.2	0	2.2	2.2	0	NS	NS	-
ENSG00000236444	RP11-223E19.2	pseudogene	NS	NS	-	NS	NS	-	2.29	2.3	0
ENSG00000235060	RP11-254N18.1	pseudogene	1.99	2	0	1.99	2	0	NS	NS	-
ENSG00000228547	RP11-271M1.2	pseudogene	0.44	-2.3	0	0.4	-2.5	0	NS	NS	-
ENSG00000171889	RP11-354P17.1	processed_transcript	NS	NS	-	NS	NS	-	12.34	12.3	0
ENSG00000185074	RP11-389K14.2	pseudogene	NS	NS	-	0.45	-2.2	0	NS	NS	-
ENSG00000234790	RP11-426L16.2	pseudogene	NS	NS	-	NS	NS	-	2.02	2	0
ENSG00000205861	RP11-45B20.2	antisense	NS	NS	-	NS	NS	-	4.37	4.4	0
ENSG00000241627	RP11-464E15.3	pseudogene	0.48	-2.1	0	0.49	-2	0	NS	NS	-
ENSG00000232203	RP11-572H4.1	pseudogene	NS	NS	-	0.5	-2	0	NS	NS	-
ENSG00000236316	RP11-61N16.1	pseudogene	0.51	-2	0	0.48	-2.1	0	NS	NS	-

ENSG00000232699	RP11-758C21.1	pseudogene	NS	NS	-	NS	NS	-	2.18	2.2	0
ENSG00000230655	RP11-803B1.4	pseudogene	NS	NS	-	0.49	-2	0	NS	NS	-
ENSG00000226970	RP11-82H13.1	pseudogene	NS	NS	-	NS	NS	-	2.37	2.4	0
ENSG00000218510	RP1-224A6.2	lincRNA	0.35	-2.8	0	0.35	-2.9	0	NS	NS	-
ENSG00000102218	RP2	protein_coding	2.52	2.5	0	2.45	2.4	0	3.06	3.1	0
ENSG00000216853	RP3-399J4.1	pseudogene	0.44	-2.3	0	0.43	-2.3	0	NS	NS	-
ENSG00000226084	RP4-706A16.1	pseudogene	0.5	-2	0	0.48	-2.1	0	NS	NS	-
ENSG00000226415	RP5-1102E8.1	pseudogene	NS	NS	-	NS	NS	-	2.86	2.9	0
ENSG00000189372	RP6-149D17.1	pseudogene	0.32	-3.2	0	0.32	-3.2	0	NS	NS	-
ENSG00000205763	RP9P	pseudogene	NS	NS	-	NS	NS	-	2.52	2.5	0
ENSG00000103494	RPGRIP1L	protein_coding	2.12	2.1	0	2.05	2	0	2.67	2.7	0
ENSG00000181031	RPH3AL	protein_coding	0.37	-2.7	0	0.37	-2.7	0	NS	NS	-
ENSG00000108107	RPL28	protein_coding	NS	NS	-	NS	NS	-	2.24	2.2	0
ENSG00000100316	RPL3	protein_coding	0.5	-2	0	0.51	-2	0	NS	NS	-
ENSG00000145425	RPS3A	protein_coding	0.35	-2.9	0	0.34	-3	0	NS	NS	-
ENSG00000117676	RPS6KA1	protein_coding	NS	NS	-	NS	NS	-	0.41	-2.4	0
ENSG00000166592	RRAD	protein_coding	NS	NS	-	NS	NS	-	3.84	3.8	0
ENSG00000116954	RRAGC	protein_coding	2.16	2.2	0	NS	NS	-	3.86	3.9	0
ENSG00000126458	RRAS	protein_coding	2.72	2.7	0	2.4	2.4	0	5.3	5.3	0
ENSG00000125844	RRBP1	protein_coding	0.4	-2.5	0	0.41	-2.5	0	0.34	-2.9	0
ENSG00000081019	RSBN1	protein_coding	NS	NS	-	NS	NS	-	0.43	-2.3	0
ENSG00000146374	RSPO3	protein_coding	NS	NS	-	NS	NS	-	6.72	6.7	0
ENSG00000136514	RTP4	protein_coding	NS	NS	-	2.1	2.1	0	NS	NS	-
ENSG00000159216	RUNX1	protein_coding	4.23	4.2	0	4.29	4.3	0	NS	NS	-
ENSG00000079102	RUNX1T1	protein_coding	2.83	2.8	0	NS	NS	-	4.69	4.7	0
ENSG00000124813	RUNX2	protein_coding	6.54	6.5	0	6.75	6.7	0	4.93	4.9	0
ENSG00000175792	RUVBL1	protein_coding	2.58	2.6	0	2.52	2.5	0	3.03	3	0
ENSG00000171509	RXFP1	protein_coding	NS	NS	-	NS	NS	-	13.23	13.2	0
ENSG00000163602	RYBP	protein_coding	NS	NS	-	NS	NS	-	2.81	2.8	0
ENSG00000198626	RYR2	protein_coding	0.26	-3.9	0	0.27	-3.6	0	0.12	-8.2	0
ENSG00000163191	S100A11	protein_coding	4.28	4.3	0	4.14	4.1	0	5.45	5.5	0
ENSG00000163221	S100A12	protein_coding	0.38	-2.6	0	0.39	-2.6	0	NS	NS	-
ENSG00000189171	S100A13	protein_coding	NS	NS	-	NS	NS	-	2.89	2.9	0
ENSG00000188643	S100A16	protein_coding	4.17	4.2	0	3.93	3.9	0	6.03	6	0
ENSG00000188015	S100A3	protein_coding	NS	NS	-	NS	NS	-	4.28	4.3	0
ENSG00000196154	S100A4	protein_coding	NS	NS	-	NS	NS	-	12.02	12	0
ENSG00000197956	S100A6	protein_coding	3.11	3.1	0	3.15	3.1	0	2.83	2.8	0
ENSG00000163993	S100P	protein_coding	7.02	7	0	7.78	7.8	0	NS	NS	-
ENSG00000151835	SACS	protein_coding	NS	NS	-	NS	NS	-	2.78	2.8	0
ENSG00000177570	SAMD12	protein_coding	NS	NS	-	NS	NS	-	0.32	-3.1	0
ENSG00000205413	SAMD9	protein_coding	4.85	4.8	0	5.22	5.2	0	NS	NS	-
ENSG00000177409	SAMD9L	protein_coding	3.44	3.4	0	3.6	3.6	0	NS	NS	-

ENSG00000155307	SAMSN1	protein_coding	NS	NS	-	3.24	3.2	0	NS	NS	-
ENSG00000079332	SAR1A	protein_coding	NS	NS	-	NS	NS	-	2.2	2.2	0
ENSG00000123453	SARDH	protein_coding	0.48	-2.1	0	0.49	-2.1	0	NS	NS	-
ENSG00000130066	SAT1	protein_coding	2	2	0	2.08	2.1	0	NS	NS	-
ENSG00000052802	SC4MOL	protein_coding	2.89	2.9	0	2.57	2.6	0	NS	NS	-
ENSG00000227500	SCAMP4	protein_coding	NS	NS	-	NS	NS	-	2.32	2.3	0
ENSG00000140386	SCAPER	protein_coding	NS	NS	-	NS	NS	-	0.44	-2.3	0
ENSG00000145284	SCD5	protein_coding	0.43	-2.3	0	0.41	-2.4	0	NS	NS	-
ENSG00000184178	SCFD2	protein_coding	NS	NS	-	NS	NS	-	2.46	2.5	0
ENSG00000205209	SCGBL	protein_coding	0.42	-2.4	0	0.41	-2.4	0	NS	NS	-
ENSG00000079689	SCGN	protein_coding	0.21	-4.8	0	0.23	-4.4	0	0.06	-16.8	0
ENSG00000006747	SCIN	protein_coding	NS	NS	-	NS	NS	-	4.05	4.1	0
ENSG00000136546	SCN7A	protein_coding	NS	NS	-	NS	NS	-	0.16	-6.1	0
ENSG00000196876	SCN8A	protein_coding	NS	NS	-	NS	NS	-	2.5	2.5	0
ENSG00000163156	SCNM1	protein_coding	NS	NS	-	NS	NS	-	3.73	3.7	0
ENSG00000111319	SCNN1A	protein_coding	NS	NS	-	NS	NS	-	0.14	-6.9	0
ENSG00000121064	SCPEP1	protein_coding	2.59	2.6	0	2.37	2.4	0	4.37	4.4	0
ENSG00000080293	SCTR	protein_coding	0.29	-3.4	0	0.31	-3.2	0	0.15	-6.9	0
ENSG00000162512	SDC3	protein_coding	NS	NS	-	NS	NS	-	4.27	4.3	0
ENSG00000132581	SDF2	protein_coding	NS	NS	-	NS	NS	-	2.23	2.2	0
ENSG00000146555	SDK1	protein_coding	0.35	-2.8	0	0.36	-2.8	0	NS	NS	-
ENSG00000184860	SDR42E1	protein_coding	NS	NS	-	NS	NS	-	0.4	-2.5	0
ENSG00000166562	SEC11C	protein_coding	0.29	-3.5	0	0.31	-3.2	0	NS	NS	-
ENSG00000120341	SEC16B	protein_coding	NS	NS	-	NS	NS	-	0.43	-2.3	0
ENSG00000100934	SEC23A	protein_coding	2.56	2.6	0	2.42	2.4	0	3.66	3.7	0
ENSG00000187742	SECISBP2	protein_coding	NS	NS	-	NS	NS	-	0.45	-2.2	0
ENSG00000141574	SECTM1	protein_coding	NS	NS	-	NS	NS	-	3.4	3.4	0
ENSG00000071537	SEL1L	protein_coding	0.23	-4.3	0	0.24	-4.2	0	NS	NS	-
ENSG00000110876	SELPLG	protein_coding	3.09	3.1	0	3.31	3.3	0	NS	NS	-
ENSG00000075213	SEMA3A	protein_coding	5.17	5.2	0	4.43	4.4	0	11.08	11.1	0
ENSG00000075223	SEMA3C	protein_coding	12.24	12.2	0	11.09	11.1	0	21.41	21.4	0
ENSG00000153993	SEMA3D	protein_coding	NS	NS	-	NS	NS	-	11.19	11.2	0
ENSG00000095539	SEMA4G	protein_coding	NS	NS	-	NS	NS	-	0.46	-2.2	0
ENSG00000112902	SEMA5A	protein_coding	NS	NS	-	NS	NS	-	3.39	3.4	0
ENSG00000137872	SEMA6D	protein_coding	0.41	-2.5	0	0.4	-2.5	0	NS	NS	-
ENSG00000138623	SEMA7A	protein_coding	3.62	3.6	0	3.56	3.6	0	4.07	4.1	0
ENSG00000162430	SEPN1	protein_coding	NS	NS	-	NS	NS	-	2.18	2.2	0
ENSG00000125354	SEPT06	protein_coding	2.6	2.6	0	2.69	2.7	0	NS	NS	-
ENSG00000184640	SEPT09	protein_coding	2.47	2.5	0	2.34	2.3	0	3.55	3.5	0
ENSG00000186522	SEPT10	protein_coding	2.25	2.2	0	2.15	2.1	0	NS	NS	-
ENSG00000138758	SEPT11	protein_coding	2.45	2.5	0	2.22	2.2	0	4.34	4.3	0
ENSG00000168528	SERINC2	protein_coding	2.53	2.5	0	2.53	2.5	0	NS	NS	-

ENSG00000197249	SERPINA1	protein_coding	NS	NS	-	2.58	2.6	0	0.07	-13.6	0
ENSG00000196136	SERPINA3	protein_coding	NS	NS	-	NS	NS	-	0.02	-59.9	0
ENSG00000188488	SERPINA5	protein_coding	0.28	-3.5	0	NS	NS	-	0.06	-17.4	0
ENSG00000170099	SERPINA6	protein_coding	NS	NS	-	NS	NS	-	0.28	-3.6	0
ENSG00000206075	SERPINB5	protein_coding	9.14	9.1	0	10.18	10.2	0	NS	NS	-
ENSG00000170542	SERPINB9	protein_coding	NS	NS	-	2.64	2.6	0	NS	NS	-
ENSG00000135919	SERPINE2	protein_coding	NS	NS	-	NS	NS	-	39.13	39.1	0
ENSG00000149257	SERPINH1	protein_coding	5.11	5.1	0	4.56	4.6	0	9.57	9.6	0
ENSG00000163536	SERPINI1	protein_coding	0.24	-4.2	0	0.24	-4.2	0	NS	NS	-
ENSG00000114204	SERPINI2	protein_coding	0.06	-16.2	0	0.07	-14.8	0	0.01	-72	0
ENSG00000179833	SERTAD2	protein_coding	NS	NS	-	NS	NS	-	4.28	4.3	0
ENSG00000082497	SERTAD4	protein_coding	2.84	2.8	0	NS	NS	-	4.01	4	0
ENSG00000130766	SESN2	protein_coding	NS	NS	-	NS	NS	-	2.38	2.4	0
ENSG00000149212	SESN3	protein_coding	NS	NS	-	2.67	2.7	0	NS	NS	-
ENSG00000187231	SESTD1	protein_coding	NS	NS	-	1.95	2	0	NS	NS	-
ENSG00000198879	SFMBT2	protein_coding	NS	NS	-	2.52	2.5	0	NS	NS	-
ENSG00000175793	SFN	protein_coding	4.99	5	0	5.47	5.5	0	NS	NS	-
ENSG00000106483	SFRP4	protein_coding	NS	NS	-	12.68	12.7	0	NS	NS	-
ENSG00000120057	SFRP5	protein_coding	0.29	-3.5	0	0.3	-3.3	0	0.17	-5.8	0
ENSG00000107819	SFXN3	protein_coding	2.12	2.1	0	2.04	2	0	2.74	2.7	0
ENSG00000183605	SFXN4	protein_coding	NS	NS	-	NS	NS	-	0.43	-2.3	0
ENSG00000163069	SGCB	protein_coding	2.03	2	0	NS	NS	-	4.2	4.2	0
ENSG00000170624	SGCD	protein_coding	NS	NS	-	NS	NS	-	7.79	7.8	0
ENSG00000118473	SGIP1	protein_coding	4.52	4.5	0	4.22	4.2	0	6.91	6.9	0
ENSG00000118515	SGK1	protein_coding	NS	NS	-	0.5	-2	0	NS	NS	-
ENSG00000162878	SGK493	protein_coding	NS	NS	-	2.29	2.3	0	NS	NS	-
ENSG00000163082	SGPP2	protein_coding	NS	NS	-	3.5	3.5	0	NS	NS	-
ENSG00000197860	SGTB	protein_coding	1.98	2	0	NS	NS	-	3.59	3.6	0
ENSG00000198478	SH3BGRL2	protein_coding	NS	NS	-	NS	NS	-	0.11	-9	0
ENSG00000141985	SH3GL1	protein_coding	NS	NS	-	NS	NS	-	2.32	2.3	0
ENSG00000147010	SH3KBP1	protein_coding	2.86	2.9	0	2.76	2.8	0	NS	NS	-
ENSG00000107957	SH3PXD2A	protein_coding	2.49	2.5	0	2.41	2.4	0	3.19	3.2	0
ENSG00000174705	SH3PXD2B	protein_coding	2.49	2.5	0	2.33	2.3	0	3.76	3.8	0
ENSG00000035115	SH3YL1	protein_coding	0.44	-2.3	0	0.47	-2.1	0	0.2	-4.9	0
ENSG00000162105	SHANK2	protein_coding	0.43	-2.3	0	0.46	-2.2	0	0.2	-5	0
ENSG00000160691	SHC1	protein_coding	NS	NS	-	NS	NS	-	2.42	2.4	0
ENSG00000148082	SHC3	protein_coding	NS	NS	-	0.34	-3	0	NS	NS	-
ENSG00000198892	SHISA4	protein_coding	NS	NS	-	NS	NS	-	3.01	3	0
ENSG00000164054	SHISA5	protein_coding	2.12	2.1	0	2	2	0	NS	NS	-
ENSG00000168779	SHOX2	protein_coding	NS	NS	-	NS	NS	-	4.62	4.6	0
ENSG00000149577	SIDT2	protein_coding	0.38	-2.6	0	0.37	-2.7	0	NS	NS	-
ENSG00000142178	SIK1	protein_coding	0.37	-2.7	0	0.37	-2.7	0	NS	NS	-

ENSG00000112246	SIM1	protein_coding	NS	NS	-	0.4	-2.5	0	NS	NS	-
ENSG00000198053	SIRPA	protein_coding	NS	NS	-	NS	NS	-	2.58	2.6	0
ENSG00000005020	SKAP2	protein_coding	4	4	0	4.15	4.1	0	NS	NS	-
ENSG00000136603	SKIL	protein_coding	2.75	2.7	0	2.89	2.9	0	NS	NS	-
ENSG00000139737	SLAIN1	protein_coding	NS	NS	-	NS	NS	-	0.13	-7.4	0
ENSG00000158714	SLAMF8	protein_coding	NS	NS	-	3.41	3.4	0	NS	NS	-
ENSG00000064651	SLC12A2	protein_coding	NS	NS	-	3.11	3.1	0	NS	NS	-
ENSG00000140199	SLC12A6	protein_coding	2.11	2.1	0	2.14	2.1	0	NS	NS	-
ENSG00000088386	SLC15A1	protein_coding	NS	NS	-	NS	NS	-	0.27	-3.7	0
ENSG00000110446	SLC15A3	protein_coding	2.59	2.6	0	2.69	2.7	0	NS	NS	-
ENSG00000155380	SLC16A1	protein_coding	4.64	4.6	0	4.59	4.6	0	4.98	5	0
ENSG00000112394	SLC16A10	protein_coding	0.17	-5.7	0	0.19	-5.2	0	0.04	-26.8	0
ENSG00000152779	SLC16A12	protein_coding	0.09	-11.1	0	0.1	-10.5	0	0.05	-20.8	0
ENSG00000174327	SLC16A13	protein_coding	NS	NS	-	NS	NS	-	2.58	2.6	0
ENSG00000168679	SLC16A4	protein_coding	4.04	4	0	4.25	4.3	0	NS	NS	-
ENSG00000146039	SLC17A4	protein_coding	0.18	-5.6	0	0.2	-5.1	0	0.05	-19.5	0
ENSG00000119899	SLC17A5	protein_coding	NS	NS	-	NS	NS	-	3	3	0
ENSG00000110436	SLC1A2	protein_coding	0.19	-5.3	0	0.18	-5.7	0	NS	NS	-
ENSG00000185052	SLC24A3	protein_coding	2.43	2.4	0	2.58	2.6	0	NS	NS	-
ENSG00000115840	SLC25A12	protein_coding	2.2	2.2	0	2.16	2.2	0	2.5	2.5	0
ENSG00000102743	SLC25A15	protein_coding	0.13	-7.5	0	0.13	-7.7	0	NS	NS	-
ENSG00000177542	SLC25A22	protein_coding	NS	NS	-	0.5	-2	0	NS	NS	-
ENSG00000085491	SLC25A24	protein_coding	2.39	2.4	0	2.43	2.4	0	NS	NS	-
ENSG00000148339	SLC25A25	protein_coding	0.26	-3.9	0	0.25	-4.1	0	NS	NS	-
ENSG00000160785	SLC25A44	protein_coding	NS	NS	-	NS	NS	-	2.09	2.1	0
ENSG00000162241	SLC25A45	protein_coding	0.36	-2.8	0	0.35	-2.9	0	NS	NS	-
ENSG00000164638	SLC29A4	protein_coding	0.45	-2.2	0	0.46	-2.2	0	NS	NS	-
ENSG00000117394	SLC2A1	protein_coding	6.42	6.4	0	6.71	6.7	0	NS	NS	-
ENSG00000158014	SLC30A2	protein_coding	0.21	-4.9	0	0.21	-4.8	0	0.19	-5.4	0
ENSG00000205060	SLC35B4	protein_coding	NS	NS	-	NS	NS	-	2.69	2.7	0
ENSG00000181830	SLC35C1	protein_coding	NS	NS	-	NS	NS	-	2.37	2.4	0
ENSG00000123643	SLC36A1	protein_coding	2.05	2.1	0	2.02	2	0	NS	NS	-
ENSG00000160190	SLC37A1	protein_coding	NS	NS	-	2.4	2.4	0	NS	NS	-
ENSG00000137700	SLC37A4	processed_transcript	0.38	-2.7	0	0.39	-2.5	0	NS	NS	-
ENSG00000188338	SLC38A3	processed_transcript	0.16	-6.2	0	0.17	-5.9	0	NS	NS	-
ENSG00000139209	SLC38A4	protein_coding	0.24	-4.2	0	0.23	-4.4	0	NS	NS	-
ENSG00000017483	SLC38A5	protein_coding	0.25	-4	0	0.26	-3.8	0	NS	NS	-
ENSG00000139974	SLC38A6	protein_coding	2.09	2.1	0	NS	NS	-	3.69	3.7	0
ENSG00000103042	SLC38A7	protein_coding	NS	NS	-	NS	NS	-	3.02	3	0
ENSG00000196950	SLC39A10	protein_coding	2.92	2.9	0	2.95	3	0	NS	NS	-
ENSG00000165915	SLC39A13	protein_coding	NS	NS	-	NS	NS	-	3.44	3.4	0
ENSG00000104635	SLC39A14	protein_coding	0.44	-2.3	0	0.43	-2.3	0	NS	NS	-

ENSG00000139540	SLC39A5	protein_coding	0.07	-13.4	0	0.08	-12.9	0	0.05	-20.3	0
ENSG00000141424	SLC39A6	protein_coding	NS	NS	-	NS	NS	-	2.23	2.2	0
ENSG00000138821	SLC39A8	protein_coding	0.39	-2.6	0	0.36	-2.8	0	NS	NS	-
ENSG00000138079	SLC3A1	protein_coding	0.28	-3.6	0	NS	NS	-	0.07	-13.4	0
ENSG00000138449	SLC40A1	protein_coding	NS	NS	-	2.5	2.5	0	NS	NS	-
ENSG00000133065	SLC41A1	protein_coding	0.31	-3.2	0	0.28	-3.5	0	NS	NS	-
ENSG00000149150	SLC43A1	protein_coding	0.18	-5.5	0	0.18	-5.7	0	NS	NS	-
ENSG00000134802	SLC43A3	protein_coding	NS	NS	-	NS	NS	-	3.98	4	0
ENSG00000070214	SLC44A1	protein_coding	2.32	2.3	0	2.36	2.4	0	NS	NS	-
ENSG00000143036	SLC44A3	protein_coding	NS	NS	-	NS	NS	-	0.19	-5.3	0
ENSG00000204385	SLC44A4	protein_coding	NS	NS	-	7.19	7.2	0	0.23	-4.3	0
ENSG00000139508	SLC46A3	protein_coding	2.75	2.8	0	2.96	3	0	NS	NS	-
ENSG00000142494	SLC47A1	protein_coding	NS	NS	-	NS	NS	-	3.56	3.6	0
ENSG00000080493	SLC4A4	protein_coding	0.22	-4.5	0	NS	NS	-	0.02	-52	0
ENSG00000033867	SLC4A7	protein_coding	2.79	2.8	0	2.68	2.7	0	3.71	3.7	0
ENSG00000198743	SLC5A3	protein_coding	3.05	3	0	2.77	2.8	0	5.25	5.2	0
ENSG00000117834	SLC5A9	protein_coding	NS	NS	-	NS	NS	-	0.49	-2	0
ENSG00000087916	SLC6A14	protein_coding	30.88	30.9	0	34.64	34.6	0	NS	NS	-
ENSG00000063127	SLC6A16	protein_coding	0.43	-2.3	0	0.44	-2.3	0	NS	NS	-
ENSG00000131389	SLC6A6	protein_coding	5.64	5.6	0	5.89	5.9	0	3.67	3.7	0
ENSG00000003989	SLC7A2	protein_coding	0.14	-6.9	0	0.16	-6.2	0	0.02	-48.8	0
ENSG00000103064	SLC7A6	protein_coding	2.4	2.4	0	2.37	2.4	0	2.68	2.7	0
ENSG00000155465	SLC7A7	protein_coding	3.96	4	0	4.23	4.2	0	NS	NS	-
ENSG00000183023	SLC8A1	protein_coding	2.21	2.2	0	2.31	2.3	0	NS	NS	-
ENSG00000090020	SLC9A1	protein_coding	2.69	2.7	0	2.68	2.7	0	2.74	2.7	0
ENSG00000137491	SLCO2B1	protein_coding	NS	NS	-	2.05	2.1	0	0.26	-3.9	0
ENSG00000172716	SLFN11	protein_coding	2.57	2.6	0	2.67	2.7	0	NS	NS	-
ENSG00000154760	SLFN13	protein_coding	NS	NS	-	2.84	2.8	0	NS	NS	-
ENSG00000166750	SLFN5	protein_coding	2.64	2.6	0	2.62	2.6	0	NS	NS	-
ENSG00000184347	SLIT3	protein_coding	NS	NS	-	NS	NS	-	12.32	12.3	0
ENSG00000124107	SLPI	protein_coding	NS	NS	-	10.31	10.3	0	NS	NS	-
ENSG00000136824	SMC2	protein_coding	2.29	2.3	0	2.31	2.3	0	NS	NS	-
ENSG00000113810	SMC4	protein_coding	2.05	2	0	2.08	2.1	0	NS	NS	-
ENSG00000163029	SMC6	protein_coding	2.45	2.4	0	2.53	2.5	0	NS	NS	-
ENSG00000166311	SMPD1	protein_coding	NS	NS	-	NS	NS	-	4.87	4.9	0
ENSG00000130768	SMPDL3B	protein_coding	0.38	-2.6	0	0.41	-2.4	0	NS	NS	-
ENSG00000185420	SMYD3	protein_coding	NS	NS	-	NS	NS	-	3.08	3.1	0
ENSG00000124216	SNAI1	protein_coding	NS	NS	-	NS	NS	-	9.86	9.9	0
ENSG00000019549	SNAI2	protein_coding	NS	NS	-	NS	NS	-	11.2	11.2	0
ENSG00000023608	SNAPC1	protein_coding	NS	NS	-	NS	NS	-	2.59	2.6	0
ENSG00000143553	SNAPIN	protein_coding	NS	NS	-	NS	NS	-	2.38	2.4	0
ENSG00000159210	SNF8	protein_coding	NS	NS	-	NS	NS	-	2.16	2.2	0

ENSG00000163788	SNRK	protein_coding	NS	NS	-	NS	NS	-	0.4	-2.5	0
ENSG00000125870	SNRPB2	protein_coding	NS	NS	-	NS	NS	-	2.01	2	0
ENSG00000227624	SNRPEL1	pseudogene	NS	NS	-	NS	NS	-	2.04	2	0
ENSG00000172554	SNTG2	protein_coding	0.46	-2.2	0	0.46	-2.2	0	NS	NS	-
ENSG00000104497	SNX16	protein_coding	NS	NS	-	NS	NS	-	2.26	2.3	0
ENSG00000064652	SNX24	protein_coding	NS	NS	-	NS	NS	-	2.32	2.3	0
ENSG00000048471	SNX29	protein_coding	NS	NS	-	NS	NS	-	2.95	3	0
ENSG00000173548	SNX33	protein_coding	NS	NS	-	NS	NS	-	2.07	2.1	0
ENSG00000130340	SNX9	protein_coding	NS	NS	-	NS	NS	-	3.34	3.3	0
ENSG00000171150	SOCS5	protein_coding	NS	NS	-	NS	NS	-	2.31	2.3	0
ENSG00000142168	SOD1	protein_coding	2.06	2.1	0	2.06	2.1	0	NS	NS	-
ENSG00000154556	SORBS2	protein_coding	0.29	-3.5	0	0.31	-3.2	0	NS	NS	-
ENSG00000120896	SORBS3	protein_coding	NS	NS	-	NS	NS	-	2.18	2.2	0
ENSG00000137642	SORL1	protein_coding	NS	NS	-	NS	NS	-	0.11	-9.3	0
ENSG00000135899	SP110	protein_coding	2.54	2.5	0	2.66	2.7	0	NS	NS	-
ENSG00000079263	SP140	protein_coding	NS	NS	-	2.4	2.4	0	NS	NS	-
ENSG00000185404	SP140L	protein_coding	2.6	2.6	0	2.71	2.7	0	NS	NS	-
ENSG00000061656	SPAG4	protein_coding	0.4	-2.5	0	0.41	-2.4	0	NS	NS	-
ENSG00000076382	SPAG5	protein_coding	2.16	2.2	0	2.18	2.2	0	NS	NS	-
ENSG00000113140	SPARC	protein_coding	3.89	3.9	0	3.89	3.9	0	3.89	3.9	0
ENSG00000152583	SPARCL1	protein_coding	NS	NS	-	NS	NS	-	0.03	-36	0
ENSG00000163071	SPATA18	protein_coding	NS	NS	-	NS	NS	-	3.97	4	0
ENSG00000133104	SPG20	protein_coding	NS	NS	-	NS	NS	-	3.04	3	0
ENSG00000164266	SPINK1	protein_coding	NS	NS	-	NS	NS	-	0	-312	0
ENSG00000166145	SPINT1	protein_coding	NS	NS	-	NS	NS	-	0.22	-4.6	0
ENSG00000169682	SPNS1	protein_coding	NS	NS	-	NS	NS	-	2.39	2.4	0
ENSG00000152377	SPOCK1	protein_coding	4	4	0	3.14	3.1	0	10.89	10.9	0
ENSG00000107742	SPOCK2	protein_coding	NS	NS	-	2.06	2.1	0	NS	NS	-
ENSG00000152268	SPON1	processed_transcript	4.18	4.2	0	4.31	4.3	0	NS	NS	-
ENSG00000159674	SPON2	protein_coding	4.12	4.1	0	3.39	3.4	0	9.98	10	0
ENSG00000118785	SPP1	protein_coding	NS	NS	-	NS	NS	-	0.03	-28.6	0
ENSG00000166068	SPRED1	protein_coding	2.59	2.6	0	2.66	2.7	0	NS	NS	-
ENSG00000229035	SPRR2C	pseudogene	0.49	-2	0	0.48	-2.1	0	NS	NS	-
ENSG00000187678	SPRY4	protein_coding	2.05	2	0	2.05	2	0	NS	NS	-
ENSG00000100596	SPTLC2	protein_coding	2.72	2.7	0	2.87	2.9	0	NS	NS	-
ENSG00000104549	SQLE	protein_coding	4.92	4.9	0	4.75	4.7	0	6.34	6.3	0
ENSG00000137767	SQRDL	protein_coding	2.07	2.1	0	2.01	2	0	2.54	2.5	0
ENSG00000213523	SRA1	protein_coding	NS	NS	-	NS	NS	-	2.34	2.3	0
ENSG00000198911	SREBF2	protein_coding	NS	NS	-	NS	NS	-	2.18	2.2	0
ENSG00000196935	SRGAP1	protein_coding	NS	NS	-	NS	NS	-	2.38	2.4	0
ENSG00000163486	SRGAP2	protein_coding	2.4	2.4	0	2.33	2.3	0	NS	NS	-
ENSG00000149418	ST14	protein_coding	NS	NS	-	NS	NS	-	0.12	-8.3	0

ENSG00000157350	ST3GAL2	protein_coding	NS	NS	-	NS	NS	-	2.21	2.2	0
ENSG00000110080	ST3GAL4	protein_coding	2.03	2	0	2.04	2	0	NS	NS	-
ENSG00000166444	ST5	protein_coding	NS	NS	-	NS	NS	-	2.06	2.1	0
ENSG00000073849	ST6GAL1	protein_coding	NS	NS	-	3.18	3.2	0	NS	NS	-
ENSG00000070526	ST6GALNAC1	protein_coding	NS	NS	-	4.42	4.4	0	NS	NS	-
ENSG00000140557	ST8SIA2	protein_coding	NS	NS	-	NS	NS	-	2.23	2.2	0
ENSG00000113532	ST8SIA4	protein_coding	NS	NS	-	3.11	3.1	0	NS	NS	-
ENSG00000144681	STAC	protein_coding	NS	NS	-	NS	NS	-	5.8	5.8	0
ENSG00000138134	STAMBPL1	protein_coding	2.28	2.3	0	2.33	2.3	0	NS	NS	-
ENSG00000214530	STARD10	protein_coding	NS	NS	-	NS	NS	-	0.28	-3.6	0
ENSG00000164211	STARD4	protein_coding	2.32	2.3	0	2.14	2.1	0	NS	NS	-
ENSG00000172345	STARD5	protein_coding	NS	NS	-	NS	NS	-	3.91	3.9	0
ENSG00000115415	STAT1	protein_coding	2.23	2.2	0	2.14	2.1	0	NS	NS	-
ENSG00000170581	STAT2	protein_coding	2.36	2.4	0	2.22	2.2	0	NS	NS	-
ENSG00000113739	STC2	protein_coding	NS	NS	-	0.31	-3.2	0	NS	NS	-
ENSG00000164647	STEAP1	protein_coding	5.11	5.1	0	4.54	4.5	0	9.69	9.7	0
ENSG00000157214	STEAP2	protein_coding	2.03	2	0	2.06	2.1	0	NS	NS	-
ENSG00000123473	STIL	protein_coding	3.28	3.3	0	3.29	3.3	0	3.19	3.2	0
ENSG00000072786	STK10	protein_coding	2.14	2.1	0	2.08	2.1	0	NS	NS	-
ENSG00000134602	STK3	protein_coding	2.32	2.3	0	2.49	2.5	0	NS	NS	-
ENSG00000130413	STK33	protein_coding	NS	NS	-	NS	NS	-	0.3	-3.4	0
ENSG00000112079	STK38	protein_coding	3	3	0	2.92	2.9	0	3.62	3.6	0
ENSG00000211455	STK38L	protein_coding	NS	NS	-	2.23	2.2	0	NS	NS	-
ENSG00000101109	STK4	protein_coding	2.11	2.1	0	2.22	2.2	0	NS	NS	-
ENSG00000117632	STMN1	protein_coding	2.36	2.4	0	2.36	2.4	0	NS	NS	-
ENSG00000243244	STON1	protein_coding	2.27	2.3	0	2.36	2.4	0	NS	NS	-
ENSG00000140022	STON2	protein_coding	NS	NS	-	NS	NS	-	3.73	3.7	0
ENSG00000173320	STOX2	protein_coding	NS	NS	-	NS	NS	-	0.39	-2.6	0
ENSG00000101846	STS	protein_coding	2.57	2.6	0	NS	NS	-	4.15	4.2	0
ENSG00000135823	STX6	protein_coding	2.37	2.4	0	2.39	2.4	0	NS	NS	-
ENSG00000079950	STX7	protein_coding	NS	NS	-	1.99	2	0	NS	NS	-
ENSG00000060140	STYK1	protein_coding	NS	NS	-	2.7	2.7	0	NS	NS	-
ENSG00000137573	SULF1	protein_coding	20.46	20.5	0	20.85	20.9	0	17.31	17.3	0
ENSG00000196562	SULF2	protein_coding	5.5	5.5	0	5.58	5.6	0	NS	NS	-
ENSG00000106868	SUSD1	protein_coding	2.24	2.2	0	2.23	2.2	0	NS	NS	-
ENSG00000143502	SUSD4	protein_coding	NS	NS	-	NS	NS	-	0.44	-2.3	0
ENSG00000185518	SV2B	protein_coding	0.3	-3.3	0	NS	NS	-	0.1	-9.6	0
ENSG00000133789	SWAP70	protein_coding	NS	NS	-	NS	NS	-	2.43	2.4	0
ENSG00000179751	SYCN	protein_coding	0.04	-23	0	0.05	-21.1	0	0.01	-84.6	0
ENSG00000162520	SYNC	protein_coding	NS	NS	-	NS	NS	-	7.71	7.7	0
ENSG00000054654	SYNE2	protein_coding	NS	NS	-	NS	NS	-	0.15	-6.9	0
ENSG00000132718	SYT11	protein_coding	2.54	2.5	0	2.3	2.3	0	NS	NS	-

ENSG00000137501	SYTL2	protein_coding	5	5	0	5.36	5.4	0	NS	NS	-
ENSG00000171148	TADA3L	protein_coding	NS	NS	-	NS	NS	-	2.09	2.1	0
ENSG00000197780	TAF13	protein_coding	NS	NS	-	NS	NS	-	5.88	5.9	0
ENSG00000106290	TAF6	protein_coding	NS	NS	-	NS	NS	-	2.1	2.1	0
ENSG00000149591	TAGLN	protein_coding	4.11	4.1	0	3.66	3.7	0	NS	NS	-
ENSG00000170921	TANC2	protein_coding	2.13	2.1	0	2.16	2.2	0	NS	NS	-
ENSG00000168394	TAP1	protein_coding	3.67	3.7	0	3.89	3.9	0	NS	NS	-
ENSG00000204267	TAP2	protein_coding	2.15	2.1	0	2.27	2.3	0	NS	NS	-
ENSG00000213977	TAX1BP3	protein_coding	NS	NS	-	NS	NS	-	3.21	3.2	0
ENSG00000095383	TBC1D2	protein_coding	3.3	3.3	0	3.07	3.1	0	NS	NS	-
ENSG00000214946	TBC1D26	protein_coding	0.5	-2	0	0.48	-2.1	0	NS	NS	-
ENSG00000111490	TBC1D30	protein_coding	0.29	-3.4	0	0.31	-3.2	0	0.13	-7.8	0
ENSG00000105254	TBCB	protein_coding	NS	NS	-	NS	NS	-	3.1	3.1	0
ENSG00000183735	TBK1	protein_coding	2.29	2.3	0	2.28	2.3	0	2.36	2.4	0
ENSG00000092607	TBX15	protein_coding	NS	NS	-	NS	NS	-	11.76	11.8	0
ENSG00000112837	TBX18	protein_coding	NS	NS	-	NS	NS	-	10.99	11	0
ENSG00000135111	TBX3	protein_coding	NS	NS	-	NS	NS	-	3.69	3.7	0
ENSG00000059377	TBXAS1	protein_coding	NS	NS	-	2.39	2.4	0	0.37	-2.7	0
ENSG00000165929	TC2N	protein_coding	NS	NS	-	0.36	-2.7	0	0.01	-121.6	0
ENSG00000204219	TCEA3	protein_coding	0.26	-3.8	0	0.27	-3.7	0	0.19	-5.3	0
ENSG00000154582	TCEB1	protein_coding	2.09	2.1	0	NS	NS	-	3.71	3.7	0
ENSG00000196628	TCF4	protein_coding	1.98	2	0	1.98	2	0	NS	NS	-
ENSG00000148737	TCF7L2	protein_coding	NS	NS	-	NS	NS	-	2.13	2.1	0
ENSG00000154316	TDH	pseudogene	0.23	-4.4	0	0.22	-4.6	0	NS	NS	-
ENSG00000151790	TDO2	protein_coding	5.92	5.9	0	6.08	6.1	0	NS	NS	-
ENSG00000129566	TEP1	protein_coding	2.26	2.3	0	2.37	2.4	0	NS	NS	-
ENSG00000135269	TES	protein_coding	NS	NS	-	1.99	2	0	NS	NS	-
ENSG00000088992	TESC	protein_coding	NS	NS	-	NS	NS	-	0.1	-9.7	0
ENSG00000087510	TFAP2C	protein_coding	NS	NS	-	NS	NS	-	6.99	7	0
ENSG00000115112	TFCP2L1	protein_coding	NS	NS	-	NS	NS	-	0.2	-5.1	0
ENSG00000198176	TFDP1	protein_coding	NS	NS	-	NS	NS	-	2.49	2.5	0
ENSG00000068323	TFE3	protein_coding	NS	NS	-	NS	NS	-	2.74	2.7	0
ENSG00000105967	TFEC	protein_coding	NS	NS	-	3.73	3.7	0	NS	NS	-
ENSG00000003436	TFPI	protein_coding	2.4	2.4	0	2.5	2.5	0	NS	NS	-
ENSG00000105329	TGFB1	protein_coding	3.33	3.3	0	3.19	3.2	0	4.51	4.5	0
ENSG00000140682	TGFB1I1	protein_coding	NS	NS	-	NS	NS	-	3.3	3.3	0
ENSG00000120708	TGFBI	protein_coding	5.3	5.3	0	4.83	4.8	0	9.06	9.1	0
ENSG00000106799	TGFBR1	protein_coding	3.08	3.1	0	3.08	3.1	0	3.02	3	0
ENSG00000198959	TGM2	protein_coding	5.32	5.3	0	5.8	5.8	0	NS	NS	-
ENSG00000186340	THBS2	protein_coding	12.27	12.3	0	12.1	12.1	0	13.6	13.6	0
ENSG00000154096	THY1	protein_coding	7.67	7.7	0	6.26	6.3	0	18.93	18.9	0
ENSG00000145365	TIFA	protein_coding	0.41	-2.5	0	0.41	-2.4	0	NS	NS	-

ENSG00000134375	TIMM17A	protein_coding	NS	NS	-	NS	NS	-	2.77	2.8	0
ENSG00000102265	TIMP1	protein_coding	4.79	4.8	0	4.62	4.6	0	NS	NS	-
ENSG00000035862	TIMP2	protein_coding	3.15	3.1	0	2.74	2.7	0	6.36	6.4	0
ENSG00000106829	TLE4	protein_coding	NS	NS	-	NS	NS	-	2.91	2.9	0
ENSG00000038295	TLL1	protein_coding	NS	NS	-	NS	NS	-	0.42	-2.4	0
ENSG00000137076	TLN1	protein_coding	2.41	2.4	0	2.3	2.3	0	NS	NS	-
ENSG00000136869	TLR4	protein_coding	2.54	2.5	0	2.65	2.6	0	NS	NS	-
ENSG00000174130	TLR6	protein_coding	2.9	2.9	0	3.12	3.1	0	NS	NS	-
ENSG00000169903	TM4SF4	protein_coding	NS	NS	-	NS	NS	-	0.03	-33.9	0
ENSG00000136404	TM6SF1	protein_coding	NS	NS	-	2.61	2.6	0	NS	NS	-
ENSG00000149809	TM7SF2	protein_coding	0.37	-2.7	0	0.36	-2.8	0	NS	NS	-
ENSG00000103534	TMC5	protein_coding	NS	NS	-	10.51	10.5	0	NS	NS	-
ENSG00000170537	TMC7	protein_coding	5.17	5.2	0	5.55	5.5	0	NS	NS	-
ENSG00000057704	TMCC3	protein_coding	NS	NS	-	NS	NS	-	0.36	-2.8	0
ENSG00000157315	TMED6	protein_coding	0.06	-15.9	0	0.07	-14.8	0	NS	NS	-
ENSG00000241697	TMEFF1	protein_coding	NS	NS	-	NS	NS	-	5.43	5.4	0
ENSG00000109066	TMEM104	protein_coding	NS	NS	-	NS	NS	-	3.2	3.2	0
ENSG00000178307	TMEM11	protein_coding	NS	NS	-	NS	NS	-	3.39	3.4	0
ENSG00000183160	TMEM119	protein_coding	NS	NS	-	NS	NS	-	8.16	8.2	0
ENSG00000179178	TMEM125	protein_coding	0.41	-2.4	0	0.43	-2.3	0	NS	NS	-
ENSG00000170647	TMEM133	protein_coding	3.8	3.8	0	4.04	4	0	NS	NS	-
ENSG00000149483	TMEM138	protein_coding	NS	NS	-	NS	NS	-	3.68	3.7	0
ENSG00000170006	TMEM154	protein_coding	5.07	5.1	0	5.36	5.4	0	NS	NS	-
ENSG00000152128	TMEM163	protein_coding	NS	NS	-	NS	NS	-	0.21	-4.7	0
ENSG00000174695	TMEM167A	protein_coding	2.67	2.7	0	2.57	2.6	0	NS	NS	-
ENSG00000157111	TMEM171	protein_coding	NS	NS	-	NS	NS	-	4.35	4.3	0
ENSG00000184584	TMEM173	protein_coding	1.97	2	0	NS	NS	-	2.62	2.6	0
ENSG00000002933	TMEM176A	protein_coding	2.71	2.7	0	2.76	2.8	0	NS	NS	-
ENSG00000198792	TMEM184B	protein_coding	2.36	2.4	0	2.12	2.1	0	4.26	4.3	0
ENSG00000240849	TMEM189	protein_coding	2.89	2.9	0	2.59	2.6	0	5.31	5.3	0
ENSG00000139291	TMEM19	protein_coding	2.07	2.1	0	2.06	2.1	0	NS	NS	-
ENSG00000176273	TMEM20	protein_coding	0.43	-2.3	0	0.41	-2.4	0	NS	NS	-
ENSG00000164484	TMEM200A	protein_coding	2.11	2.1	0	2.04	2	0	2.7	2.7	0
ENSG00000186501	TMEM222	protein_coding	NS	NS	-	NS	NS	-	2.32	2.3	0
ENSG00000182107	TMEM30B	protein_coding	NS	NS	-	NS	NS	-	0.22	-4.6	0
ENSG00000121775	TMEM39B	protein_coding	NS	NS	-	NS	NS	-	2.25	2.3	0
ENSG00000151715	TMEM45B	protein_coding	NS	NS	-	7.25	7.2	0	NS	NS	-
ENSG00000147027	TMEM47	protein_coding	NS	NS	-	NS	NS	-	6.12	6.1	0
ENSG00000058804	TMEM48	protein_coding	NS	NS	-	2.01	2	0	NS	NS	-
ENSG00000171729	TMEM51	protein_coding	NS	NS	-	NS	NS	-	0.27	-3.7	0
ENSG00000178821	TMEM52	protein_coding	0.47	-2.1	0	0.46	-2.2	0	NS	NS	-
ENSG00000121900	TMEM54	protein_coding	2.05	2	0	2.1	2.1	0	NS	NS	-

ENSG00000155099	TMEM55A	protein_coding	2.07	2.1	0	NS	NS	-	3.76	3.8	0
ENSG00000152078	TMEM56	protein_coding	0.42	-2.4	0	NS	NS	-	0.15	-6.8	0
ENSG00000137216	TMEM63B	protein_coding	2.09	2.1	0	1.97	2	0	3.07	3.1	0
ENSG00000187783	TMEM72	protein_coding	0.43	-2.3	0	0.42	-2.4	0	NS	NS	-
ENSG00000153214	TMEM87B	protein_coding	2.49	2.5	0	2.61	2.6	0	NS	NS	-
ENSG00000136842	TMOD1	protein_coding	NS	NS	-	NS	NS	-	0.1	-9.9	0
ENSG00000138594	TMOD3	protein_coding	1.97	2	0	1.99	2	0	NS	NS	-
ENSG00000184012	TMPRSS2	protein_coding	NS	NS	-	NS	NS	-	0.05	-21.2	0
ENSG00000137648	TMPRSS4	protein_coding	18.69	18.7	0	20.91	20.9	0	NS	NS	-
ENSG00000034510	TMSB10	protein_coding	2.34	2.3	0	2.31	2.3	0	2.52	2.5	0
ENSG00000179104	TMTC2	protein_coding	1.98	2	0	2.04	2	0	NS	NS	-
ENSG00000139324	TMTC3	protein_coding	2.2	2.2	0	2.02	2	0	3.63	3.6	0
ENSG00000166479	TMX3	protein_coding	NS	NS	-	NS	NS	-	2.58	2.6	0
ENSG00000041982	TNC	protein_coding	7	7	0	4.75	4.8	0	24.99	25	0
ENSG00000185215	TNFAIP2	protein_coding	2.44	2.4	0	2.59	2.6	0	NS	NS	-
ENSG00000123610	TNFAIP6	protein_coding	7.43	7.4	0	6.27	6.3	0	NS	NS	-
ENSG00000173530	TNFRSF10D	protein_coding	NS	NS	-	NS	NS	-	3.62	3.6	0
ENSG00000164761	TNFRSF11B	protein_coding	NS	NS	-	NS	NS	-	31.84	31.8	0
ENSG00000028137	TNFRSF1B	protein_coding	NS	NS	-	1.96	2	0	NS	NS	-
ENSG00000146072	TNFRSF21	protein_coding	2.88	2.9	0	3.13	3.1	0	NS	NS	-
ENSG00000102524	TNFSF13B	protein_coding	NS	NS	-	2.26	2.3	0	NS	NS	-
ENSG00000181634	TNFSF15	protein_coding	2.73	2.7	0	2.92	2.9	0	NS	NS	-
ENSG00000125657	TNFSF9	protein_coding	NS	NS	-	NS	NS	-	2.54	2.5	0
ENSG00000154310	TNIK	protein_coding	2.38	2.4	0	2.56	2.6	0	NS	NS	-
ENSG00000131746	TNS4	protein_coding	NS	NS	-	10.71	10.7	0	NS	NS	-
ENSG00000100284	TOM1	protein_coding	NS	NS	-	NS	NS	-	2.6	2.6	0
ENSG00000131747	TOP2A	protein_coding	5.12	5.1	0	5.41	5.4	0	NS	NS	-
ENSG00000136827	TOR1A	protein_coding	NS	NS	-	NS	NS	-	2.19	2.2	0
ENSG00000136816	TOR1B	protein_coding	NS	NS	-	NS	NS	-	2.8	2.8	0
ENSG00000141510	TP53	protein_coding	NS	NS	-	NS	NS	-	2.76	2.8	0
ENSG00000164938	TP53INP1	protein_coding	NS	NS	-	0.5	-2	0	NS	NS	-
ENSG00000076554	TPD52	protein_coding	NS	NS	-	NS	NS	-	0.18	-5.6	0
ENSG00000111669	TPI1	protein_coding	2.33	2.3	0	2.3	2.3	0	NS	NS	-
ENSG00000198467	TPM2	protein_coding	4.96	5	0	4.28	4.3	0	10.47	10.5	0
ENSG00000167460	TPM4	protein_coding	3.34	3.3	0	3.24	3.2	0	4.17	4.2	0
ENSG00000166340	TPP1	protein_coding	NS	NS	-	NS	NS	-	3.46	3.5	0
ENSG00000169902	TPST1	protein_coding	NS	NS	-	NS	NS	-	2.54	2.5	0
ENSG00000128294	TPST2	protein_coding	0.22	-4.5	0	0.21	-4.7	0	NS	NS	-
ENSG00000088325	TPX2	protein_coding	4.91	4.9	0	4.94	4.9	0	4.63	4.6	0
ENSG00000056972	TRAF3IP2	protein_coding	NS	NS	-	NS	NS	-	2.59	2.6	0
ENSG00000131653	TRAF7	protein_coding	NS	NS	-	NS	NS	-	2.28	2.3	0
ENSG00000182606	TRAK1	protein_coding	2.33	2.3	0	2.33	2.3	0	NS	NS	-

ENSG00000065308	TRAM2	protein_coding	3.03	3	0	2.31	2.3	0	8.79	8.8	0
ENSG00000170043	TRAPPC1	protein_coding	NS	NS	-	NS	NS	-	3.5	3.5	0
ENSG00000095970	TREM2	protein_coding	NS	NS	-	3.36	3.4	0	NS	NS	-
ENSG00000072657	TRHDE	protein_coding	0.06	-16.7	0	0.06	-16.5	0	0.06	-18.1	0
ENSG00000071575	TRIB2	protein_coding	2.82	2.8	0	2.91	2.9	0	NS	NS	-
ENSG00000106785	TRIM14	protein_coding	NS	NS	-	2.04	2	0	NS	NS	-
ENSG00000221926	TRIM16	protein_coding	3.63	3.6	0	3.6	3.6	0	3.85	3.8	0
ENSG00000132274	TRIM22	protein_coding	2.26	2.3	0	2.23	2.2	0	NS	NS	-
ENSG00000204616	TRIM31	protein_coding	NS	NS	-	6.07	6.1	0	NS	NS	-
ENSG00000108395	TRIM37	protein_coding	NS	NS	-	NS	NS	-	2.24	2.2	0
ENSG00000112343	TRIM38	protein_coding	2.25	2.3	0	2.28	2.3	0	NS	NS	-
ENSG00000213186	TRIM59	protein_coding	4.04	4	0	4.01	4	0	4.24	4.2	0
ENSG00000038382	TRIO	protein_coding	2.03	2	0	1.99	2	0	NS	NS	-
ENSG00000071539	TRIP13	protein_coding	NS	NS	-	NS	NS	-	2	2	0
ENSG00000104447	TRPS1	protein_coding	3.82	3.8	0	3.69	3.7	0	4.82	4.8	0
ENSG00000165125	TRPV6	protein_coding	0.23	-4.4	0	0.24	-4.1	0	NS	NS	-
ENSG00000182463	TSHZ2	protein_coding	2.96	3	0	3.17	3.2	0	NS	NS	-
ENSG00000121297	TSHZ3	protein_coding	3.26	3.3	0	2.96	3	0	5.68	5.7	0
ENSG00000182704	TSKU	protein_coding	2.9	2.9	0	2.8	2.8	0	NS	NS	-
ENSG00000206549	TSP50	protein_coding	0.49	-2	0	0.49	-2.1	0	NS	NS	-
ENSG00000117472	TSPAN1	protein_coding	20.06	20.1	0	22.51	22.5	0	NS	NS	-
ENSG00000106537	TSPAN13	protein_coding	NS	NS	-	NS	NS	-	0.15	-6.5	0
ENSG00000099282	TSPAN15	protein_coding	NS	NS	-	2.6	2.6	0	0.27	-3.8	0
ENSG00000214063	TSPAN4	protein_coding	NS	NS	-	NS	NS	-	2.71	2.7	0
ENSG00000168785	TSPAN5	protein_coding	3.97	4	0	3.09	3.1	0	10.95	11	0
ENSG00000156298	TSPAN7	protein_coding	NS	NS	-	NS	NS	-	0.08	-12.2	0
ENSG00000127324	TSPAN8	protein_coding	NS	NS	-	4.76	4.8	0	0.05	-19	0
ENSG00000100300	TSPO	protein_coding	2.22	2.2	0	2.02	2	0	3.85	3.9	0
ENSG00000052841	TTC17	protein_coding	NS	NS	-	NS	NS	-	0.46	-2.2	0
ENSG00000155158	TTC39B	protein_coding	2.1	2.1	0	2.21	2.2	0	NS	NS	-
ENSG00000114999	TTL	protein_coding	NS	NS	-	NS	NS	-	2.85	2.9	0
ENSG00000100271	TTLL1	protein_coding	NS	NS	-	NS	NS	-	3.06	3.1	0
ENSG00000137941	TTLL7	protein_coding	0.4	-2.5	0	0.39	-2.6	0	NS	NS	-
ENSG00000155657	TTN	protein_coding	0.28	-3.6	0	0.29	-3.4	0	0.15	-6.5	0
ENSG00000124120	TTPAL	protein_coding	NS	NS	-	NS	NS	-	3.38	3.4	0
ENSG00000118271	TTR	protein_coding	NS	NS	-	NS	NS	-	0.01	-77.2	0
ENSG00000167614	TTYH1	protein_coding	0.41	-2.4	0	0.42	-2.4	0	0.4	-2.5	0
ENSG00000136295	TTYH3	protein_coding	2.69	2.7	0	2.53	2.5	0	3.98	4	0
ENSG00000127824	TUBA1	protein_coding	NS	NS	-	1.98	2	0	NS	NS	-
ENSG00000167552	TUBA1A	protein_coding	2.65	2.7	0	2.37	2.4	0	NS	NS	-
ENSG00000123416	TUBA1B	protein_coding	NS	NS	-	NS	NS	-	2.01	2	0
ENSG00000167553	TUBA1C	protein_coding	4.16	4.2	0	3.71	3.7	0	7.74	7.7	0

ENSG00000196230	TUBB	protein_coding	NS	NS	-	NS	NS	-	2.62	2.6	0
ENSG00000137267	TUBB2A	protein_coding	NS	NS	-	NS	NS	-	6.09	6.1	0
ENSG00000176014	TUBB6	protein_coding	NS	NS	-	NS	NS	-	2.65	2.6	0
ENSG00000037042	TUBG2	protein_coding	0.5	-2	0	0.44	-2.3	0	NS	NS	-
ENSG00000078246	TULP3	protein_coding	NS	NS	-	NS	NS	-	2.32	2.3	0
ENSG00000173366	TWF2	protein_coding	2.25	2.3	0	2.18	2.2	0	2.81	2.8	0
ENSG00000122691	TWIST1	protein_coding	NS	NS	-	NS	NS	-	5.94	5.9	0
ENSG00000128791	TWSG1	protein_coding	2.72	2.7	0	2.29	2.3	0	6.14	6.1	0
ENSG00000136810	TXN	protein_coding	3.06	3.1	0	2.89	2.9	0	4.48	4.5	0
ENSG00000115514	TXNDC9	protein_coding	1.99	2	0	1.96	2	0	NS	NS	-
ENSG00000117289	TXNIP	protein_coding	NS	NS	-	NS	NS	-	0.31	-3.3	0
ENSG00000198431	TXNRD1	protein_coding	3.05	3	0	2.72	2.7	0	5.69	5.7	0
ENSG00000176890	TYMS	protein_coding	2.8	2.8	0	NS	NS	-	4.31	4.3	0
ENSG00000137831	UACA	protein_coding	2.13	2.1	0	2.23	2.2	0	NS	NS	-
ENSG00000134882	UBAC2	protein_coding	NS	NS	-	NS	NS	-	2.06	2.1	0
ENSG00000143569	UBAP2L	protein_coding	NS	NS	-	NS	NS	-	2.08	2.1	0
ENSG00000154127	UBASH3B	protein_coding	2.31	2.3	0	2.16	2.2	0	NS	NS	-
ENSG00000077721	UBE2A	protein_coding	NS	NS	-	NS	NS	-	2.72	2.7	0
ENSG00000072401	UBE2D1	protein_coding	3.02	3	0	2.83	2.8	0	4.56	4.6	0
ENSG00000182247	UBE2E2	protein_coding	NS	NS	-	NS	NS	-	4.39	4.4	0
ENSG00000185651	UBE2L3	protein_coding	NS	NS	-	NS	NS	-	2.45	2.5	0
ENSG00000156587	UBE2L6	protein_coding	1.98	2	0	1.96	2	0	NS	NS	-
ENSG00000077152	UBE2T	protein_coding	4.9	4.9	0	4.65	4.7	0	6.86	6.9	0
ENSG00000177414	UBE2U	protein_coding	0.47	-2.1	0	0.47	-2.1	0	NS	NS	-
ENSG00000175567	UCP2	protein_coding	NS	NS	-	2.58	2.6	0	NS	NS	-
ENSG00000148154	UGCG	protein_coding	2.08	2.1	0	2.05	2	0	NS	NS	-
ENSG00000242515	UGT1A10	protein_coding	43.58	43.6	0	48.88	48.9	0	NS	NS	-
ENSG00000135220	UGT2A3	protein_coding	NS	NS	-	NS	NS	-	0.12	-8.1	0
ENSG00000196620	UGT2B15	protein_coding	NS	NS	-	NS	NS	-	0.23	-4.3	0
ENSG00000174607	UGT8	protein_coding	NS	NS	-	NS	NS	-	0.4	-2.5	0
ENSG00000152332	UHMK1	protein_coding	2.19	2.2	0	2.27	2.3	0	NS	NS	-
ENSG00000169344	UMOD	protein_coding	NS	NS	-	0.51	-2	0	NS	NS	-
ENSG00000107731	UNC5B	protein_coding	2.03	2	0	2.09	2.1	0	NS	NS	-
ENSG00000036672	USP2	protein_coding	0.35	-2.8	0	0.29	-3.4	0	NS	NS	-
ENSG00000136014	USP44	protein_coding	0.49	-2	0	0.5	-2	0	NS	NS	-
ENSG00000111962	UST	protein_coding	NS	NS	-	0.5	-2	0	NS	NS	-
ENSG00000103043	VAC14	protein_coding	NS	NS	-	NS	NS	-	2.16	2.2	0
ENSG00000139190	VAMP1	protein_coding	2.06	2.1	0	NS	NS	-	3.67	3.7	0
ENSG00000118640	VAMP8	protein_coding	NS	NS	-	NS	NS	-	0.07	-14.6	0
ENSG00000168140	VASN	protein_coding	NS	NS	-	NS	NS	-	4.3	4.3	0
ENSG00000108828	VAT1	protein_coding	NS	NS	-	NS	NS	-	5.44	5.4	0
ENSG00000141968	VAV1	protein_coding	NS	NS	-	2.04	2	0	NS	NS	-

ENSG00000160293	VAV2	protein_coding	NS	NS	-	NS	NS	-	0.4	-2.5	0
ENSG00000038427	VCAN	protein_coding	11.75	11.7	0	12.25	12.3	0	NS	NS	-
ENSG00000035403	VCL	protein_coding	2.45	2.5	0	2.32	2.3	0	3.53	3.5	0
ENSG00000111424	VDR	protein_coding	4.13	4.1	0	3.71	3.7	0	7.43	7.4	0
ENSG00000150630	VEGFC	protein_coding	NS	NS	-	NS	NS	-	5.1	5.1	0
ENSG00000197415	VEPH1	protein_coding	0.2	-5	0	0.19	-5.3	0	0.28	-3.6	0
ENSG00000206538	VGLL3	protein_coding	NS	NS	-	NS	NS	-	10.8	10.8	0
ENSG00000127831	VIL1	protein_coding	NS	NS	-	NS	NS	-	0.3	-3.4	0
ENSG00000026025	VIM	protein_coding	NS	NS	-	NS	NS	-	2.88	2.9	0
ENSG00000106018	VIPR2	protein_coding	0.21	-4.7	0	0.21	-4.8	0	0.23	-4.3	0
ENSG00000205221	VIT	protein_coding	NS	NS	-	NS	NS	-	5.35	5.3	0
ENSG00000147852	VLDLR	protein_coding	0.27	-3.7	0	0.25	-4	0	NS	NS	-
ENSG00000104142	VPS18	protein_coding	NS	NS	-	NS	NS	-	2.33	2.3	0
ENSG00000122958	VPS26A	protein_coding	NS	NS	-	NS	NS	-	2	2	0
ENSG00000139719	VPS33A	protein_coding	NS	NS	-	NS	NS	-	1.96	2	0
ENSG00000101842	VSIG1	protein_coding	NS	NS	-	13.54	13.5	0	NS	NS	-
ENSG00000134258	VTCN1	protein_coding	0.28	-3.6	0	0.3	-3.3	0	0.07	-13.4	0
ENSG00000112290	WASF1	protein_coding	NS	NS	-	NS	NS	-	4.13	4.1	0
ENSG00000132970	WASF3	protein_coding	NS	NS	-	NS	NS	-	2.5	2.5	0
ENSG00000132471	WBP2	protein_coding	NS	NS	-	NS	NS	-	2.19	2.2	0
ENSG00000185222	WBP5	protein_coding	2.15	2.1	0	1.96	2	0	3.68	3.7	0
ENSG00000185274	WBSCR17	protein_coding	0.47	-2.1	0	0.48	-2.1	0	0.41	-2.5	0
ENSG00000139668	WDFY2	protein_coding	0.46	-2.2	0	0.47	-2.1	0	0.37	-2.7	0
ENSG00000198554	WDHD1	protein_coding	2.32	2.3	0	2.23	2.2	0	NS	NS	-
ENSG00000071127	WDR1	protein_coding	2.42	2.4	0	2.31	2.3	0	3.36	3.4	0
ENSG00000139323	WDR51B	protein_coding	NS	NS	-	2.22	2.2	0	NS	NS	-
ENSG00000162643	WDR63	protein_coding	NS	NS	-	NS	NS	-	3.65	3.7	0
ENSG00000115935	WIPF1	protein_coding	3.69	3.7	0	3.77	3.8	0	NS	NS	-
ENSG00000104415	WISP1	protein_coding	3.1	3.1	0	3.19	3.2	0	NS	NS	-
ENSG00000165238	WNK2	protein_coding	0.32	-3.1	0	0.33	-3.1	0	0.31	-3.3	0
ENSG00000114251	WNT5A	protein_coding	4.16	4.2	0	3.13	3.1	0	NS	NS	-
ENSG00000132530	XAF1	protein_coding	3.02	3	0	3.25	3.3	0	NS	NS	-
ENSG00000100219	XBP1	protein_coding	0.46	-2.2	0	0.47	-2.1	0	NS	NS	-
ENSG00000124343	XG	protein_coding	NS	NS	-	NS	NS	-	13.44	13.4	0
ENSG00000169180	XPO6	protein_coding	NS	NS	-	NS	NS	-	2.71	2.7	0
ENSG00000152422	XRCC4	protein_coding	3.36	3.4	0	3.51	3.5	0	NS	NS	-
ENSG00000163872	YEATS2	protein_coding	2	2	0	2.01	2	0	NS	NS	-
ENSG00000091436	ZAK	protein_coding	NS	NS	-	NS	NS	-	2.79	2.8	0
ENSG00000126804	ZBTB1	protein_coding	1.98	2	0	1.97	2	0	NS	NS	-
ENSG00000109906	ZBTB16	protein_coding	0.22	-4.5	0	0.24	-4.2	0	0.09	-11.1	0
ENSG00000174282	ZBTB4	protein_coding	NS	NS	-	NS	NS	-	2.34	2.3	0
ENSG00000165424	ZCCHC24	protein_coding	NS	NS	-	NS	NS	-	2.6	2.6	0

ENSG00000204186	ZDBF2	protein_coding	0.4	-2.5	0	0.38	-2.6	0	NS	NS	-
ENSG00000188818	ZDHHC11	protein_coding	0.5	-2	0	0.5	-2	0	NS	NS	-
ENSG00000204160	ZDHHC18	protein_coding	NS	NS	-	NS	NS	-	1.96	2	0
ENSG00000180776	ZDHHC20	protein_coding	2.13	2.1	0	2.23	2.2	0	NS	NS	-
ENSG00000163812	ZDHHC3	protein_coding	2	2	0	1.97	2	0	NS	NS	-
ENSG00000153786	ZDHHC7	protein_coding	2.48	2.5	0	2.41	2.4	0	3.02	3	0
ENSG00000148516	ZEB1	protein_coding	2.76	2.8	0	2.63	2.6	0	NS	NS	-
ENSG00000169554	ZEB2	protein_coding	2.43	2.4	0	2.43	2.4	0	NS	NS	-
ENSG00000091656	ZFHX4	protein_coding	3.23	3.2	0	3.04	3	0	4.76	4.8	0
ENSG00000103994	ZFP106	protein_coding	NS	NS	-	NS	NS	-	3.01	3	0
ENSG00000185650	ZFP36L1	protein_coding	NS	NS	-	NS	NS	-	2.11	2.1	0
ENSG00000165861	ZFYVE1	protein_coding	NS	NS	-	NS	NS	-	2.56	2.6	0
ENSG00000039319	ZFYVE16	protein_coding	2	2	0	2.02	2	0	NS	NS	-
ENSG00000166432	ZMAT1	protein_coding	NS	NS	-	NS	NS	-	0.14	-7.2	0
ENSG00000172667	ZMAT3	protein_coding	2.37	2.4	0	1.97	2	0	5.55	5.5	0
ENSG00000136870	ZNF189	protein_coding	0.43	-2.3	0	0.44	-2.3	0	0.33	-3.1	0
ENSG00000204789	ZNF204P	pseudogene	NS	NS	-	NS	NS	-	0.14	-7.4	0
ENSG00000165512	ZNF22	protein_coding	NS	NS	-	NS	NS	-	0.48	-2.1	0
ENSG00000185947	ZNF267	protein_coding	2.35	2.4	0	2.49	2.5	0	NS	NS	-
ENSG00000189180	ZNF33A	protein_coding	NS	NS	-	NS	NS	-	0.29	-3.4	0
ENSG00000196693	ZNF33B	protein_coding	0.48	-2.1	0	NS	NS	-	0.17	-5.8	0
ENSG00000148143	ZNF462	protein_coding	2.24	2.2	0	2.31	2.3	0	NS	NS	-
ENSG00000196268	ZNF493	protein_coding	NS	NS	-	NS	NS	-	0.4	-2.5	0
ENSG00000165655	ZNF503	protein_coding	0.49	-2	0	0.45	-2.2	0	NS	NS	-
ENSG00000177853	ZNF518A	processed_transcript	NS	NS	-	NS	NS	-	0.31	-3.3	0
ENSG00000198795	ZNF521	protein_coding	3.96	4	0	3.64	3.6	0	6.51	6.5	0
ENSG00000074657	ZNF532	protein_coding	3.05	3.1	0	3.1	3.1	0	NS	NS	-
ENSG00000120963	ZNF706	protein_coding	NS	NS	-	NS	NS	-	0.49	-2	0
ENSG00000140548	ZNF710	protein_coding	0.33	-3.1	0	0.33	-3.1	0	NS	NS	-
ENSG00000147180	ZNF711	protein_coding	NS	NS	-	NS	NS	-	0.48	-2.1	0
ENSG00000196597	ZNF782	protein_coding	0.48	-2.1	0	0.5	-2	0	NS	NS	-
ENSG00000151612	ZNF827	protein_coding	NS	NS	-	NS	NS	-	1.96	2	0
ENSG00000198824	ZNF828	protein_coding	NS	NS	-	NS	NS	-	2.14	2.1	0
ENSG00000178917	ZNF852	protein_coding	NS	NS	-	NS	NS	-	0.47	-2.1	0
ENSG00000197385	ZNF860	protein_coding	3.45	3.4	0	3.74	3.7	0	NS	NS	-
ENSG00000167232	ZNF91	protein_coding	NS	NS	-	NS	NS	-	0.3	-3.3	0
ENSG00000124201	ZNFX1	protein_coding	2.26	2.3	0	2.24	2.2	0	NS	NS	-
ENSG00000174442	ZWILCH	protein_coding	2.23	2.2	0	2.23	2.2	0	NS	NS	-
ENSG00000159840	ZYX	protein_coding	2.23	2.2	0	1.96	2	0	4.43	4.4	0

NS: non-statistically significative; T: tumor samples; GEP-A/B: gene expression profile subgroups A and B of PDAC tumors as assessed by unsupervised principal component (PCA) and hierarchical clustering (HCA) analyses; Non-T: non-tumoral samples.
