# Supplementary material for: Identification and characterization of the gene expression profiles for protein coding and non-coding RNAs of pancreatic ductal adenocarcinomas
Source: Oncotarget. 2015 May 22;6(22):19070–86. doi: 10.18632/oncotarget.4233 (PMC4662476; doi:10.18632/oncotarget.4233)
Supplement: Supplementary file 4 [file oncotarget-06-19070-s004.pdf]

SUPPLEMENTARY TABLE 3. Gene transcripts differentially expressed in PDAC tumor tissues (n=27), and both the GEP-A (n=24) and GEP-B (n=3) subgroups of PDACs vs non- tumoral pancreatic tissues (n=5) analyzed with the Affymetrix miRNA 2.0 Expression array.

Gene ID	Gene name (by Affymetrix array)	Transcript description	Ratio T vs Non-T	Fold Change T vs Non-T	q-value (%)	Ratio T GEP-A vs Non-T	Fold Change GEP-A vs Non-T	q-value (%)	Ratio T GEP-B vs Non-T	Fold Change GEP-B vs Non-T	q-value (%)
ENSG00000254279	*14qII-1*	CDBox	0,11	-8,8	0	0,13	-7,8	0	0,01	-127,0	0
ENSG00000202270	*14qII-12*	CDBox	0,26	-3,8	0	0,28	-3,5	0	NS	NS	-
ENSG00000199593	*14qII-14*	CDBox	NS	NS	-	NS	NS	-	0,06	-16,5	0
ENSG00000200406	*14qII-23*	CDBox	0,42	-2,4	0	0,43	-2,3	0	NS	NS	-
ENSG00000206811	*ACA10*	HAcaBox	0,23	-4,3	0	0,25	-4,1	0	0,11	-9,4	0
ENSG00000207168	*ACA15*	HAcaBox	0,42	-2,4	0	0,43	-2,3	0	NS	NS	-
ENSG00000199293	*ACA21*	HAcaBox	0,29	-3,4	0	0,32	-3,1	0	0,08	-12,1	0
ENSG00000269893	*ACA24*	HAcaBox	0,08	-13,1	0	0,08	-11,9	0	0,02	-58,2	0
ENSG00000252808	*ACA26*	HAcaBox	0,39	-2,5	0	0,41	-2,4	0	0,23	-4,4	0
ENSG00000206799	*ACA32*	HAcaBox	0,47	-2,1	0	0,48	-2,1	0	NS	NS	-
ENSG00000200816	*ACA38*	HAcaBox	0,42	-2,4	0	0,41	-2,5	0	NS	NS	-
ENSG00000207406	*ACA41*	HAcaBox	0,36	-2,8	0	0,37	-2,7	0	0,27	-3,7	0
ENSG00000207475	*ACA42*	HAcaBox	0,47	-2,1	0	0,48	-2,1	0	NS	NS	-
ENSG00000207493	*ACA46*	HAcaBox	0,29	-3,5	0	0,30	-3,4	0	NS	NS	-
ENSG00000271798	*ACA51*	HAcaBox	0,33	-3,0	0	0,34	-2,9	0	NS	NS	-
ENSG00000199785	*ACA52*	HAcaBox	0,39	-2,5	0	0,42	-2,4	0	NS	NS	-
ENSG00000207008	*ACA54*	HAcaBox	NS	NS	-	NS	NS	-	0,17	-5,9	0
ENSG00000201457	*ACA55*	HAcaBox	0,31	-3,3	0	0,32	-3,2	0	NS	NS	-
ENSG00000251898	*ACA57*	HAcaBox	0,11	-9,0	0	0,12	-8,3	0	0,04	-26,5	0
ENSG00000197989	*ACA61*	HAcaBox	NS	NS	-	NS	NS	-	0,27	-3,7	0
ENSG00000266402	*ACA62*	HAcaBox	0,44	-2,3	0	0,47	-2,1	0	NS	NS	-
ENSG00000207496	*ACA7*	HAcaBox	NS	NS	-	NS	NS	-	0,08	-11,8	0
ENSG00000207088	*ACA7B*	HAcaBox	0,38	-2,7	0	NS	NS	-	0,18	-5,7	0
ENSG00000207304	*ACA8*	HAcaBox	NS	NS	-	NS	NS	-	0,25	-4,1	0
NA	*E3*	NA	0,29	-3,5	0	0,30	-3,4	0	0,20	-4,9	0
ENSG00000212588	*HBI-6*	CDBox	NS	NS	-	NS	NS	-	0,27	-3,7	0
ENSG00000221803	*HBII-115*	CDBox	0,27	-3,7	0	0,29	-3,4	0	0,10	-10,0	0
ENSG00000270704	*HBII-13*	CDBox	0,28	-3,6	0	0,30	-3,3	0	0,11	-8,9	0

ENSG00000212158	*HBII-142*	CDBox	0,17	-5,7	0	0,19	-5,3	0	0,06	-17,3	0
ENSG00000221241	*HBII-180A*	CDBox	0,20	-4,9	0	0,21	-4,7	0	0,13	-7,8	0
ENSG00000220988	*HBII-180C*	CDBox	0,33	-3,1	0	0,32	-3,1	0	NS	NS	-
ENSG00000212452	*HBII-210*	CDBox	0,13	-7,7	0	0,14	-7,2	0	0,07	-15,1	0
ENSG00000200181	*HBII-251*	CDBox	0,29	-3,5	0	0,30	-3,4	0	0,24	-4,2	0
ENSG00000212283	*HBII-289*	CDBox	NS	NS	-	NS	NS	-	0,28	-3,5	0
ENSG00000212552	*HBII-296B*	snoRNA	0,46	-2,2	0	0,47	-2,1	0	NS	NS	-
ENSG00000264994	*HBII-316*	CDBox	0,37	-2,7	0	0,39	-2,6	0	NS	NS	-
ENSG00000221740	*HBII-336*	CDBox	0,17	-5,9	0	0,18	-5,5	0	0,06	-15,8	0
ENSG00000221500	*HBII-429*	CDBox	0,23	-4,4	0	0,23	-4,4	0	0,22	-4,5	0
ENSG00000207001	*HBII-85-2*	CDBox	0,24	-4,2	0	0,25	-4,0	0	0,13	-7,9	0
ENSG00000207279	*HBII-85-24*	CDBox	0,30	-3,4	0	0,30	-3,3	0	NS	NS	-
ENSG00000252326	*HBII-85-25*	CDBox	0,39	-2,6	0	0,40	-2,5	0	0,29	-3,4	0
ENSG00000251815	*HBII-85-26*	CDBox	0,20	-5,0	0	0,19	-5,3	0	NS	NS	-
ENSG00000207245	*HBII-85-29*	CDBox	0,17	-5,9	0	0,16	-6,1	0	NS	NS	-
ENSG00000207442	*HBII-85-6*	CDBox	0,26	-3,8	0	0,28	-3,6	0	NS	NS	-
ENSG00000207133	*HBII-85-7*	CDBox	0,38	-2,6	0	0,38	-2,6	0	NS	NS	-
ENSG00000207093	*HBII-85-8*	CDBox	0,32	-3,2	0	0,33	-3,0	0	NS	NS	-
ENSG00000206727	*HBII-85-9*	CDBox	0,27	-3,7	0	0,29	-3,4	0	0,13	-7,9	0
MI0000253	*hp_hsa-miR-148a*	miRNA	0,40	-2,5	0	0,36	-2,8	0	NS	NS	-
MI0000290	*hp_hsa-miR-214*	miRNA	2,10	2,1	0	2,14	2,1	0	NS	NS	-
MI0000254	*hp_hsa-miR-30c-2*	miRNA	0,41	-2,5	0	0,42	-2,4	0	NS	NS	-
MI0001445	*hp_hsa-miR-423*	miRNA	NS	NS	-	NS	NS	-	0,30	-3,3	0
MI0005764	*hp_hsa-miR-941-2*	miRNA	2,90	2,9	0	3,14	3,1	0	NS	NS	-
MI0000066	*hsa-let-7e*	miRNA	2,57	2,6	0	2,56	2,6	0	NS	NS	-
MI0000434	*hsa-let-7i*	miRNA	2,82	2,8	0	2,85	2,8	0	NS	NS	-
MI0000102	*hsa-miR-100*	miRNA	3,30	3,3	0	3,38	3,4	0	NS	NS	-
MI0000266	*hsa-miR-10a*	miRNA	10,77	10,8	0	10,52	10,5	0	12,71	12,7	0
MI0000267	*hsa-miR-10b*	miRNA	NS	NS	-	3,66	3,7	0	NS	NS	-
MI0006273	*hsa-miR-1180*	miRNA	NS	NS	-	NS	NS	-	0,14	-7,3	0
MI0006334	*hsa-miR-1202*	miRNA	0,37	-2,7	0	0,37	-2,7	0	NS	NS	-
*	*hsa-miR-1244*	miRNA	NS	NS	-	NS	NS	-	47,47	47,5	0
MI0000469	*hsa-miR-125a-5p*	miRNA	4,40	4,4	0	4,54	4,5	0	NS	NS	-
MI0014197	*hsa-miR-1260b*	miRNA	0,23	-4,4	0	0,22	-4,5	0	NS	NS	-
MI0006405	*hsa-miR-1268*	miRNA	0,43	-2,3	0	0,40	-2,5	0	NS	NS	-
MI0006415	*hsa-miR-1275*	miRNA	NS	NS	-	NS	NS	-	0,09	-11,0	0

MI0000748	*hsa-miR-130b*	miRNA	0,08	-13,0	0	0,08	-12,2	0	0,03	-31,1	0
MI0000748	*hsa-miR-130b-star*	miRNA	NS	NS	-	0,48	-2,1	0	NS	NS	-
MI0000449	*hsa-miR-132*	miRNA	NS	NS	-	3,59	3,6	0	NS	NS	-
MI0003786	*hsa-miR-1323*	miRNA	0,37	-2,7	0	0,37	-2,7	0	NS	NS	-
MI0000459	*hsa-miR-143*	miRNA	3,47	3,5	0	3,50	3,5	0	NS	NS	-
MI0000461	*hsa-miR-145*	miRNA	2,12	2,1	0	2,17	2,2	0	NS	NS	-
MI0007074	*hsa-miR-1469*	miRNA	0,37	-2,7	0	0,38	-2,6	0	NS	NS	-
MI0000477	*hsa-miR-146a*	miRNA	5,63	5,6	0	5,78	5,8	0	NS	NS	-
MI0000253	*hsa-miR-148a*	miRNA	0,07	-15,3	0	0,07	-13,7	0	0,01	-189,4	0
MI0000253	*hsa-miR-148a-star*	miRNA	0,10	-10,0	0	0,10	-10,0	0	0,10	-9,9	0
MI0000478	*hsa-miR-149*	miRNA	NS	NS	-	NS	NS	-	0,09	-11,3	0
MI0000478	*hsa-miR-149-star*	miRNA	0,39	-2,6	0	0,36	-2,7	0	NS	NS	-
MI0000479	*hsa-miR-150*	miRNA	5,34	5,3	0	5,31	5,3	0	NS	NS	-
MI0000681	*hsa-miR-155*	miRNA	21,20	21,2	0	21,29	21,3	0	20,54	20,5	0
MI0000438	*hsa-miR-15b*	miRNA	6,24	6,2	0	6,04	6,0	0	NS	NS	-
*	*hsa-miR-181a*	miRNA	2,88	2,9	0	3,04	3,0	0	NS	NS	-
MI0000271	*hsa-miR-181c*	miRNA	NS	NS	-	7,17	7,2	0	NS	NS	-
MI0000072	*hsa-miR-18a*	miRNA	7,78	7,8	0	7,66	7,7	0	NS	NS	-
MI0003137	*hsa-miR-193b*	miRNA	NS	NS	-	NS	NS	-	0,04	-22,7	0
MI0003137	*hsa-miR-193b-star*	miRNA	NS	NS	-	NS	NS	-	0,08	-13,0	0
MI0009983	*hsa-miR-1973*	miRNA	5,59	5,6	0	5,01	5,0	0	NS	NS	-
*	*hsa-miR-199a-3p*	miRNA	4,10	4,1	0	4,18	4,2	0	3,51	3,5	0
*	*hsa-miR-199a-5p*	miRNA	NS	NS	-	2,39	2,4	0	NS	NS	-
MI0000282	*hsa-miR-199b-3p*	miRNA	3,92	3,9	0	4,01	4,0	0	3,21	3,2	0
*	*hsa-miR-19b*	miRNA	0,24	-4,1	0	0,24	-4,2	0	NS	NS	-
MI0000283	*hsa-miR-203*	miRNA	43,03	43,0	0	39,03	39,0	0	75,04	75,0	0
MI0000076	*hsa-miR-20a*	miRNA	2,15	2,2	0	2,11	2,1	0	NS	NS	-
MI0001519	*hsa-miR-20b*	miRNA	2,82	2,8	0	2,86	2,9	0	NS	NS	-
MI0000077	*hsa-miR-21*	miRNA	13,10	13,1	0	12,86	12,9	0	NS	NS	-
MI0000290	*hsa-miR-214*	miRNA	2,18	2,2	0	2,26	2,3	0	NS	NS	-
MI0000292	*hsa-miR-216a*	miRNA	0,01	-68,1	0	0,02	-60,9	0	0,00	-1250,2	0
MI0005569	*hsa-miR-216b*	miRNA	0,04	-25,9	0	0,04	-23,2	0	0,00	-664,4	0
MI0000293	*hsa-miR-217*	miRNA	0,03	-31,1	0	0,04	-27,7	0	0,00	-1834,4	0
MI0000078	*hsa-miR-22*	miRNA	NS	NS	-	NS	NS	-	0,33	-3,1	0
MI0000298	*hsa-miR-221*	miRNA	2,46	2,5	0	2,56	2,6	0	NS	NS	-
MI0000299	*hsa-miR-222*	miRNA	2,97	3,0	0	3,07	3,1	0	NS	NS	-

MI0000079	*hsa-miR-23a*	miRNA	2,48	2,5	0	2,54	2,5	0	NS	NS	-
MI0000439	*hsa-miR-23b-star*	miRNA	NS	NS	-	NS	NS	-	0,17	-5,7	0
MI0000085	*hsa-miR-27a*	miRNA	4,95	4,9	0	5,02	5,0	0	NS	NS	-
MI0000086	*hsa-miR-28-5p*	miRNA	2,45	2,4	0	2,51	2,5	0	NS	NS	-
MI0000107	*hsa-miR-29b-2-star*	miRNA	NS	NS	-	NS	NS	-	0,12	-8,0	0
MI0000735	*hsa-miR-29c*	miRNA	0,32	-3,1	0	0,35	-2,8	0	0,07	-14,8	0
MI0000088	*hsa-miR-30a*	miRNA	0,32	-3,1	0	0,33	-3,0	0	0,26	-3,8	0
MI0000088	*hsa-miR-30a-star*	miRNA	0,19	-5,2	0	0,21	-4,8	0	0,07	-14,9	0
*	*hsa-miR-30c*	miRNA	0,36	-2,7	0	0,38	-2,6	0	0,24	-4,1	0
MI0000254	*hsa-miR-30c-2-star*	miRNA	0,41	-2,5	0	0,28	-3,5	0	0,05	-21,6	0
MI0000255	*hsa-miR-30d*	miRNA	0,41	-2,4	0	0,42	-2,4	0	NS	NS	-
MI0000089	*hsa-miR-31*	miRNA	23,41	23,4	0	25,87	25,9	0	NS	NS	-
*	*hsa-miR-3156*	miRNA	0,33	-3,1	0	0,30	-3,4	0	NS	NS	-
MI0014210	*hsa-miR-3176*	miRNA	0,27	-3,7	0	0,28	-3,6	0	NS	NS	-
MI0014232	*hsa-miR-3188*	miRNA	NS	NS	-	NS	NS	-	0,16	-6,4	0
MI0014240	*hsa-miR-3195*	miRNA	NS	NS	-	NS	NS	-	0,09	-11,2	0
MI0000813	*hsa-miR-324-3p*	miRNA	NS	NS	-	NS	NS	-	0,12	-8,5	0
MI0000803	*hsa-miR-330-3p*	miRNA	NS	NS	-	NS	NS	-	0,20	-5,1	0
MI0000806	*hsa-miR-337-5p*	miRNA	NS	NS	-	3,35	3,4	0	NS	NS	-
MI0000815	*hsa-miR-339-3p*	miRNA	NS	NS	-	NS	NS	-	0,02	-46,3	0
MI0000805	*hsa-miR-342-3p*	miRNA	1,98	2,0	0	2,02	2,0	0	NS	NS	-
MI0000826	*hsa-miR-346*	miRNA	NS	NS	-	2,27	2,3	0	NS	NS	-
MI0000268	*hsa-miR-34a*	miRNA	2,95	2,9	0	2,98	3,0	0	NS	NS	-
MI0000762	*hsa-miR-362-5p*	miRNA	NS	NS	-	NS	NS	-	0,08	-13,3	0
MI0000783	*hsa-miR-375*	miRNA	0,21	-4,7	0	0,22	-4,5	0	0,13	-7,8	0
MI0001446	*hsa-miR-424-star*	miRNA	9,01	9,0	0	9,71	9,7	0	NS	NS	-
MI0015885	*hsa-miR-4281*	miRNA	0,33	-3,0	0	0,30	-3,3	0	NS	NS	-
MI0015893	*hsa-miR-4284*	miRNA	NS	NS	-	0,43	-2,3	0	NS	NS	-
MI0015894	*hsa-miR-4286*	miRNA	0,07	-13,7	0	0,08	-12,7	0	0,03	-38,5	0
MI0015830	*hsa-miR-4298*	miRNA	21,43	21,4	0	14,06	14,1	0	80,35	80,3	0
MI0015850	*hsa-miR-4317*	miRNA	NS	NS	-	2,59	2,6	0	NS	NS	-
MI0001729	*hsa-miR-451*	miRNA	0,31	-3,2	0	0,34	-2,9	0	0,06	-16,9	0
MI0003126	*hsa-miR-491-5p*	miRNA	NS	NS	-	NS	NS	-	0,13	-7,6	0
MI0003134	*hsa-miR-494*	miRNA	0,38	-2,6	0	0,36	-2,8	0	NS	NS	-
MI0003188	*hsa-miR-503*	miRNA	11,55	11,6	0	12,41	12,4	0	NS	NS	-
*	*hsa-miR-513a-5p*	miRNA	0,22	-4,5	0	0,23	-4,4	0	NS	NS	-

MI0005539	*hsa-miR-541-star*	miRNA	0,50	-2,0	0	0,50	-2,0	0	NS	NS	-
MI0003581	*hsa-miR-574-3p*	miRNA	3,42	3,4	0	3,45	3,5	0	NS	NS	-
MI0003653	*hsa-miR-638*	miRNA	0,44	-2,3	0	0,45	-2,2	0	NS	NS	-
MI0006442	*hsa-miR-664-star*	miRNA	NS	NS	-	NS	NS	-	0,09	-11,5	0
MI0005543	*hsa-miR-708*	miRNA	25,27	25,3	0	26,75	26,7	0	NS	NS	-
MI0003892	*hsa-miR-762*	miRNA	0,35	-2,9	0	0,35	-2,9	0	NS	NS	-
MI0005761	*hsa-miR-939*	miRNA	0,33	-3,1	0	0,35	-2,9	0	NS	NS	-
ENSG00000238578	*mgh18S-121*	snoRNA	0,27	-3,7	0	0,29	-3,4	0	0,08	-12,9	0
ENSG00000270066	*mgU2-25-61*	lincRNA	0,18	-5,6	0	0,19	-5,3	0	NS	NS	-
ENSG00000207062	*SNORA15*	snoRNA	0,29	-3,5	0	0,30	-3,3	0	0,18	-5,7	0
ENSG00000206785	*SNORA15*	snoRNA	0,30	-3,3	0	0,31	-3,2	0	NS	NS	-
ENSG00000206903	*SNORA24*	snoRNA	0,09	-11,7	0	0,09	-10,7	0	0,02	-48,2	0
ENSG00000207130	*SNORA24*	snoRNA	0,07	-13,5	0	0,08	-12,6	0	0,03	-32,6	0
ENSG00000201042	*SNORA38*	snoRNA	0,19	-5,4	0	0,20	-5,0	0	NS	NS	-
ENSG00000200394	*SNORA38B*	snoRNA	0,12	-8,5	0	0,12	-8,1	0	0,08	-13,0	0
ENSG00000207187	*SNORA64*	snoRNA	0,31	-3,2	0	0,32	-3,1	0	0,23	-4,4	0
ENSG00000206958	*SNORA70*	snoRNA	NS	NS	-	0,49	-2,0	0	NS	NS	-
ENSG00000252853	*SNORD112*	snoRNA	NS	NS	-	NS	NS	-	2,27	2,3	0
ENSG00000202498	*SNORD116*	snoRNA	0,21	-4,8	0	0,22	-4,5	0	NS	NS	-
ENSG00000252277	*SNORD116-30*	snoRNA	0,34	-2,9	0	0,36	-2,8	0	NS	NS	-
ENSG00000239112	*SNORD123*	snoRNA	0,24	-4,1	0	0,25	-3,9	0	0,16	-6,2	0
ENSG00000202252	*SNORD14C*	snoRNA	0,18	-5,5	0	0,19	-5,3	0	NS	NS	-
ENSG00000207118	*SNORD14D*	snoRNA	0,32	-3,1	0	0,34	-2,9	0	NS	NS	-
ENSG00000201009	*SNORD46*	snoRNA	NS	NS	-	NS	NS	-	0,09	-11,7	0
ENSG00000199411	*SNORD62*	snoRNA	0,21	-4,8	0	0,22	-4,5	0	NS	NS	-
ENSG00000238936	*SNORD65*	snoRNA	0,42	-2,4	0	0,41	-2,4	0	NS	NS	-
ENSG00000212532	*SNORD66*	snoRNA	0,27	-3,7	0	0,27	-3,7	0	NS	NS	-
ENSG00000238807	*snoU3*	snoRNA	NS	NS	-	NS	NS	-	3,67	3,7	0
ENSG00000238581	*snoU3*	snoRNA	NS	NS	-	0,50	-2,0	0	NS	NS	-
ENSG00000163597	*snR38C*	processed_transcript	0,29	-3,5	0	0,30	-3,3	0	0,19	-5,3	0
ENSG00000207500	*U102*	CDBox	0,45	-2,2	0	0,43	-2,3	0	NS	NS	-
ENSG00000199753	*U104*	CDBox	0,12	-8,1	0	0,13	-7,5	0	0,05	-20,6	0
ENSG00000201348	*U105B*	CDBox	0,31	-3,3	0	0,33	-3,0	0	0,12	-8,4	0
ENSG00000221716	*U107*	CDBox	0,32	-3,1	0	0,33	-3,0	0	NS	NS	-
ENSG00000238294	*U13*	CDBox	0,34	-3,0	0	0,34	-2,9	0	NS	NS	-
ENSG00000199673	*U16*	CDBox	0,34	-2,9	0	0,34	-3,0	0	NS	NS	-

ENSG00000206780	*U23*	CDBox	0,28	-3,6	0	0,29	-3,5	0	NS	NS	-
ENSG00000206611	*U24*	CDBox	0,23	-4,3	0	0,24	-4,1	0	0,17	-5,8	0
ENSG00000255717	*U26*	CDBox	0,28	-3,6	0	0,29	-3,5	0	0,20	-4,9	0
ENSG00000255717	*U27*	CDBox	NS	NS	-	NS	NS	-	0,43	-2,3	0
ENSG00000255717	*U29*	CDBox	0,30	-3,3	0	0,31	-3,2	0	NS	NS	-
NA	*U3*	CDBox	0,30	-3,3	0	0,31	-3,2	0	NS	NS	-
ENSG00000207119	*U3*	snoRNA	0,48	-2,1	0	0,46	-2,2	0	NS	NS	-
ENSG00000201847	*U31*	CDBox	0,14	-6,9	0	0,16	-6,4	0	0,06	-18,2	0
*	*U3-2B*	CDBox	0,36	-2,8	0	0,35	-2,8	0	NS	NS	-
ENSG00000199631	*U33*	CDBox	0,32	-3,1	0	0,34	-2,9	0	0,20	-5,0	0
ENSG00000202503	*U34*	CDBox	0,27	-3,8	0	0,28	-3,6	0	NS	NS	-
ENSG00000262202	*U3-4*	CDBox	0,39	-2,6	0	0,38	-2,7	0	NS	NS	-
NA	*U35A*	CDBox	0,24	-4,2	0	0,25	-4,0	0	0,12	-8,6	0
ENSG00000200530	*U35B*	CDBox	0,45	-2,2	0	0,46	-2,2	0	NS	NS	-
ENSG00000200831	*U36B*	CDBox	0,26	-3,8	0	0,28	-3,6	0	0,11	-8,8	0
ENSG00000252542	*U36C*	CDBox	0,16	-6,4	0	0,16	-6,2	0	0,11	-8,8	0
ENSG00000206775	*U37*	CDBox	NS	NS	-	NS	NS	-	0,42	-2,4	0
ENSG00000202031	*U38A*	CDBox	0,28	-3,6	0	0,30	-3,3	0	0,07	-13,5	0
ENSG00000207421	*U38B*	CDBox	0,16	-6,2	0	0,18	-5,7	0	0,04	-25,0	0
ENSG00000263764	*U43*	CDBox	0,34	-2,9	0	0,36	-2,8	0	0,19	-5,2	0
ENSG00000201823	*U48*	CDBox	0,18	-5,4	0	0,19	-5,4	0	NS	NS	-
ENSG00000175061	*U49A*	CDBox	0,37	-2,7	0	0,39	-2,6	0	NS	NS	-
ENSG00000203875	*U50B*	CDBox	0,11	-9,0	0	0,12	-8,5	0	0,06	-15,8	0
ENSG00000207047	*U51*	CDBox	0,21	-4,7	0	0,22	-4,5	0	0,11	-8,9	0
ENSG00000265145	*U53*	CDBox	NS	NS	-	NS	NS	-	0,14	-7,0	0
ENSG00000264294	*U55*	CDBox	NS	NS	-	NS	NS	-	0,22	-4,6	0
ENSG00000229686	*U56*	CDBox	0,30	-3,3	0	0,30	-3,3	0	NS	NS	-
ENSG00000226572	*U57*	CDBox	0,36	-2,8	0	0,37	-2,7	0	0,27	-3,7	0
ENSG00000206989	*U63*	CDBox	0,21	-4,7	0	0,22	-4,5	0	0,14	-6,9	0
ENSG00000207166	*U68*	CDBox	0,23	-4,3	0	0,24	-4,1	0	0,16	-6,4	0
ENSG00000208797	*U73a*	CDBox	0,43	-2,3	0	0,45	-2,2	0	0,23	-4,3	0
ENSG00000234741	*U74*	CDBox	0,23	-4,4	0	0,24	-4,1	0	0,10	-10,5	0
ENSG00000234741	*U76*	CDBox	0,30	-3,3	0	0,31	-3,2	0	0,21	-4,7	0
ENSG00000234741	*U79*	CDBox	0,39	-2,5	0	0,38	-2,6	0	NS	NS	-
*	*U8*	CDBox	0,39	-2,6	0	0,41	-2,4	0	NS	NS	-
ENSG00000202400	*U82*	CDBox	0,25	-3,9	0	0,26	-3,9	0	NS	NS	-

ENSG00000209482	*U83A*	CDBox	0,34	-2,9	0	0,36	-2,8	0	0,23	-4,3	0
ENSG00000252945	*U83B*	CDBox	0,12	-8,3	0	0,12	-8,5	0	0,15	-6,9	0
ENSG00000265236	*U84*	CDBox	0,21	-4,8	0	0,20	-4,9	0	NS	NS	-
ENSG00000264549	*U95*	CDBox	0,38	-2,6	0	0,38	-2,6	0	0,36	-2,8	0
ENSG00000272296	*U96a*	CDBox	0,35	-2,9	0	0,35	-2,8	0	NS	NS	-
ENSG00000238622	*U97*	CDBox	0,41	-2,4	0	0,43	-2,3	0	NS	NS	-

*miRNA transcripts with various annotated stem-loop sequences or other small RNA names with various associated ENSEMBLE ID; NA: information not available; NS: non-statistically significative; GEP-A/B: gene expression profile subgroups A and B of PDAC tumors as assessed by unsupervised PCA and HCA; T: tumor samples; Non-T: non-tumor samples; snoRNA: small nucleolar RNA; HAcaBox: H/ACA box small nucleolar RNA.
