# Supplementary material for: Identification and characterization of the gene expression profiles for protein coding and non-coding RNAs of pancreatic ductal adenocarcinomas
Source: Oncotarget. 2015 May 22;6(22):19070–86. doi: 10.18632/oncotarget.4233 (PMC4662476; doi:10.18632/oncotarget.4233)
Supplement: Supplementary file 5 [file oncotarget-06-19070-s005.pdf]

SUPPLEMENTARY TABLE 4. Most representative canonical pathways involved in PDAC tumors as identified through analysis of the GEP of coding and non-coding RNAs (n=27) which were shared by the GEP-A and GEP-B subgroups of PDAC tumors.

	Canonical Pathways	N. of genes	Gene %*	Corrected p-value‡	Gene ID
Organismal Growth and Development	Axonal Guidance Signaling	66	13.8	<.001	PLCB2, GLI2, UNC5B, KRAS, ADAMTS2, LIMK1, GNB4, PLCD3, MICAL1, PAK1, ITGA3, SEMA6D, GNA15, ECE2, ADAM28, PIK3CG, ABLIM3, PLCB1, TUBA1C, SRGAP2, GNA13, ITGA4, PIK3C2B, ACTR2, CFL1, RRAS, RAC1, ITGA5, MMP2, MYL9, ARPC1A, ADAM12, FYN, ADAM17, MMP7, ARPC1B, EGF, EPHA4, PLXNA2, PDGFC, ROBO1, EFNB2, ACTR3, GLI3, EFNA5, MKNK1, ADAM19, MMP11, SHANK2, ITGB1, PLXNC1, NRP2, CXCR4, ITGA2, EPHA3, PLXND1, SEMA3A, WIPF1, PRKAR2B, TUBA1A, PAK3, ADAM10, SEMA3C, ADAM9, SEMA7A, WNT5A
	Axonal Guidance Signaling	67	14	<.001	SLIT3, TUBB, ADAMTS2, LIMK1, GNB4, PLCD3, MICAL1, SEMA3D, CFL2, ABLIM3, PLCB1, TUBA1C, GSK3B, ADAMTS5, GNG12, PAPPA, PRKCQ, CFL1, RRAS, SEMA5A, PTCH1, TUBB2A, RAC1, ITGA5, VEGFC, MMP2, TUBA1B, MYL9, ADAMTS6, ADAM12, PLCB3, PRKCH, FZD5, NTF3, MMP7, PLXNA3, MYL6B, EGF, PLXNA2, ABLIM1, ROBO1, PRKCZ, PLCD1, SHC1, GNG11, ACTR3, GLI3, SRGAP1, EFNA5, MKNK1, MMP11, PIK3R2, SHANK2, BMP1, ITGB1, PLXNA1, NRP2, NFATC4, PLXND1, SEMA3A, PRKAR2B, TUBB6, PAK3, SEMA4G, SEMA3C, ADAM9, SEMA7A
	Actin Cytoskeleton Signaling	39	16.4	<.001	FN1, ARPC1B, F2R, ACTA2, EGF, KRAS, TLN1, MYLK, SLC9A1, PDGFC, LIMK1, PAK1, ITGA3, ACTR3, FLNA, PIK3CG, DIAPH2, GNA13, VCL, TMSB10/TMSB4X, ACTN1, ITGA4, ITGB1, PIK3C2B, ACTR2, CFL1, RRAS, ITGA2, RAC1, ITGA5, TTN, MYL9, ARPC1A, PAK3, MYH9, VAV1, NCKAP1L, PIP4K2A, MSN
	Actin Cytoskeleton Signaling	33	13.9	.003	FN1, MYL6B, DIAPH3, EGF, SLC9A1, LIMK1, SHC1, IQGAP2, ACTR3, CFL2, PIK3R2, VCL, TMSB10/TMSB4X, FGF7, ACTC1, GNG12, ACTN1, VAV2, ITGB1, CFL1, RRAS, RAC1, ITGA5, FGD1, WASF1, TTN, FGF1, MYL9, PAK3, PIP5K1C, ARHGAP35, MSN, FGF5
	Virus Entry via Endocytic Pathways	25	24.8	<.001	ITGB1, B2M, PIK3C2B, AP2B1, FYN, AP2M1, RRAS, HLA-A, HLA-B, ITGA2, CLTB, ACTA2, RAC1, ITGA5, KRAS, ITGB2, CD55, ITGA3, FLNA, PIK3CG, ITGA1, ITGB4, ITGB6, ITGB5, ITGA4
	Virus Entry via Endocytic Pathways	20	19.8	.001	ITGB1, AP2B1, AP2A1, AP2M1, PRKCQ, RRAS, CLTC, RAC1, ITGA5, AP2S1, PRKCZ, FLNC, CLTA, PRKCH, PIK3R2, ITGB4, ITGB6, CXADR, ACTC1, ITGB5
	Clathrin-mediated Endocytosis Signaling	31	15.8	<.001	ARPC1B, F2R, CLTB, ACTA2, EGF, PDGFC, LYZ, ACTR3, PIK3CG, SERPINA1, ITGB4, SH3KBP1, ITGB5, ITGB1, ACTR2, AP2B1, PIK3C2B, AP2M1, RAC1, ITGA5, HIP1, APOC1, APOL1, MET, ITGB2, ALB, ARPC1A, CBL, ITGB6, CTTN, MYO1E
	Clathrin-mediated Endocytosis Signaling	29	14.8	.004	AP2A1, STON2, EGF, RAB5B, LYZ, ACTR3, SNX9, SERPINA1, ITGB4, PIK3R2, FGF7, ACTC1, ITGB5, ITGB1, AP2B1, AP2M1, CLTC, RAC1, ITGA5, VEGFC, AP2S1, FGF1, ALB, PIP5K1C, CLTA, ITGB6, CTTN, MYO1E, FGF5
	Macropinocytosis Signaling	16	2.8	<.001	ITGB1, PIK3C2B, RRAS, RAC1, EGF, ITGA5, KRAS, RAB34, PDGFC, MET, ITGB2, PAK1, PIK3CG, ITGB4, ITGB6, ITGB5
	Macropinocytosis Signaling	13	16.9	.025	ITGB1, PRKCQ, RRAS, RAC1, ITGA5, EGF, RAB34, PRKCZ, PRKCH, PIK3R2, ITGB4, ITGB6, ITGB5
	Inhibition of Matrix Metalloproteases	12	3.8	<.001	HSPG2, ADAM17, MMP7, ADAM12, TIMP1, MMP14, THBS2, ADAM10, MMP2, MMP11, LRP1, TIMP2
	Inhibition of Matrix Metalloproteases	11	28.2	.003	MMP7, ADAM12, MMP3, MMP14, RECK, THBS2, MMP2, MMP11, LRP1, MMP1, TIMP2
Cellular and/or Humoral Immune Response	Leukocyte Extravasation Signaling	37	18	<.001	MMP7, ICAM1, MMP14, ACTA2, ITGA3, TIMP1, PIK3CG, CYBB, MMP11, VCL, ACTN1, ITGA4, TIMP2, ITGB1, PIK3C2B, CXCR4, CLDN18, ITGA2, RAC1, THY1, ITGA5, MMP2, NCF4, SELPLG, BTK, ITGB2, WIPF1, ITGAM, EDIL3, JAM3, NCF2, ITGA1, VAV1, ARHGAP1, CTTN, CLDN3, MSN
	Leukocyte Extravasation Signaling	36	17.6	<.001	MMP7, CLDN11, MMP3, MMP14, CLDN7, MAPK13, PRKCZ, CLDN4, CYBB, MMP11, PIK3R2, VCL, ACTC1, MMP1, ACTN1, TIMP2, VAV2, ITGB1, CLDN10, PRKCQ, RAC1, THY1, ITGA5, MMP2, F11R, CDH5, JAM3, RAP1GAP, CLDN1, NCF2, ARHGAP35, PRKCH, ARHGAP1, CTTN, CLDN3, MSN
	IL-8 Signaling	31	14	<.001	PLCB2, ICAM1, DIRAS3, EGF, KRAS, PDGFC, EIF4EBP1, LIMK1, GNB4, PIK3CG, CYBB, ITGAV, GNA13, RHOF, ITGB5, PIK3C2B, NOX4, RHOC, RRAS, RAC1, MMP2, IRAK3, CSTB, MYL9, ITGB2, ITGAM, NCF2, MAP4K4, FNBP1, IRAK2, ITGAX

	IL-8 Signaling	29	13.1	.005	EGF, PRKCZ, LIMK1, HMOX1, GNB4, GNG11, RHOG, RHOU, CYBB, PIK3R2, ITGB5, GNG12, LASP1, PRKCQ, RHOC, RRAS, RAC1, VEGFC, MMP2, BAX, CSTB, MYL9, CDH1, RND3, NCF2, PRKCH, PTGS2, KDR, MAP4K4
	Agranulocyte Adhesion and Diapedesis	28	15.5	.002	MMP7, ICAM1, FN1, MMP14, ACTA2, CXCL10, ITGA3, MMP11, ITGA4, ITGB1, CXCR4, CLDN18, ITGA2, CKLF, ITGA5, C5, MMP2, SELPLG, MYL9, CXCL16, ITGB2, IL18, JAM3, IL1RN, MYH9, ITGA1, CLDN3, MSN
	Agranulocyte Adhesion and Diapedesis	25	13.8	.025	ITGB1, CLDN10, MMP7, CLDN11, FN1, MMP3, MYL6B, MMP14, ITGA5, MMP2, CLDN7, MYL9, ICAM2, CDH5, CLDN4, JAM3, CLDN1, CCL21, MMP11, CXCL17, CD34, ACTC1, CLDN3, MMP1, MSN
	Granulocyte Adhesion and Diapedesis	27	15.7	.002	MMP7, ICAM1, MMP14, CXCL10, ITGA3, MMP11, IL1RAP, ITGA4, ITGB1, CXCR4, ITGA2, CLDN18, CKLF, ITGA5, THY1, C5, MMP2, SELPLG, ITGB2, CXCL16, IL18, ITGAM, JAM3, IL1RN, ITGA1, CLDN3, MSN
	Granulocyte Adhesion and Diapedesis	23	13.4	.043	ITGB1, CLDN10, CLDN11, MMP7, MMP3, MMP14, THY1, ITGA5, MMP2, SDC3, CLDN7, ICAM2, CLDN4, CDH5, CLDN1, JAM3, CCL21, MMP11, CXCL17, MMP1, CLDN3, TNFRSF11B, MSN
**Intracellular and Second Messenger Signaling**	Signaling by Rho Family GTPases	45	17	<.001	ARPC1B, SEPT9, DIRAS3, ACTA2, MYLK, SEPT11, SLC9A1, PARD6A, CDH11, LIMK1, GNB4, PAK1, ITGA3, STMN1, ACTR3, GNA15, CDH3, PIK3CG, CYBB, ARHGEF2, GNA13, RHOF, ITGA4, ITGB1, PIK3C2B, ACTR2, NOX4, CFL1, RHOC, ITGA2, RAC1, CDH6, ITGA5, KIAA1804, MYL9, WIPF1, NEDD4, ARPC1A, PAK3, SEPT10, NCF2, PIP4K2A, SEPT6, FNBP1, MSN
	Signaling by Rho Family GTPases	41	15.5	<.001	SEPT9, MYL6B, DIAPH3, WASF3, CDC42EP2, SEPT11, SLC9A1, CDH11, PRKCZ, LIMK1, GNB4, GNG11, ACTR3, RHOG, CFL2, RHOU, CYBB, PIK3R2, CDH13, ACTC1, GNG12, ITGB1, CFL1, RHOC, RAC1, VIM, ITGA5, CDC42EP3, WASF1, KIAA1804, MYL9, CDH1, CDH2, NEDD4, CDH5, RND3, PAK3, PIP5K1C, NCF2, ARHGEF10, MSN
	RhoGDI Signaling	32	16	<.001	ARPC1B, ARHGDIG, DIRAS3, ACTA2, CDH11, LIMK1, GNB4, ITGA3, PAK1, ACTR3, GNA15, CDH3, ARHGEF2, GNA13, RHOF, ITGA4, ITGB1, ACTR2, CFL1, RHOC, ITGA2, RAC1, CDH6, ITGA5, ARHGDIB, MYL9, ARPC1A, PAK3, PIP4K2A, ARHGAP1, FNBP1, MSN
	RhoGDI Signaling	30	15	.001	MYL6B, ARHGDIG, CDH11, LIMK1, GNB4, RHOG, ACTR3, GNG11, CFL2, RHOU, ACTC1, CDH13, GNG12, ITGB1, CFL1, RHOC, RAC1, ITGA5, WASF1, MYL9, CDH2, CDH1, RND3, PAK3, CDH5, PIP5K1C, ARHGAP35, ARHGEF10, ARHGAP1, MSN
	RhoA Signaling	23	18.4	<.001	ACTR2, CFL1, SEPT9, NRP2, ARPC1B, ANLN, ACTA2, MYLK, SEPT11, LPAR3, TTN, LIMK1, MYL9, NEDD4, LPAR6, ACTR3, ARPC1A, SEPT10, GNA13, ARHGAP1, PIP4K2A, SEPT6, MSN
	RhoA Signaling	23	18.4	.002	PLXNA1, CFL1, SEPT9, NRP2, ANLN, MYL6B, CDC42EP3, WASF1, SEPT11, CDC42EP2, TTN, LIMK1, MYL9, NEDD4, ACTR3, LPAR1, CFL2, RND3, PIP5K1C, ARHGAP35, ARHGAP1, ACTC1, MSN
**Cellular Growth, Proliferation and Development**	Epithelial Adherens Junction Signaling	22	14.5	.016	ACTR2, TGFBR1, LMO7, ARPC1B, RRAS, FGFR1, ACTA2, ACVR1, RAC1, EGF, BMPR2, KRAS, MET, MYL9, ACTR3, ARPC1A, TUBA1A, MYH9, LEF1, TUBA1C, VCL, ACTN1
	Epithelial Adherens Junction Signaling	27	17.8	<.001	SNAI2, TGFBR1, MYL6B, EGF, TUBB, ACTR3, TUBA1C, VCL, ACTC1, ACTN1, VAV2, LMO7, RRAS, SNAI1, TUBB2A, RAC1, ACVR1, WASF1, TUBA1B, FGF1, MYL9, CDH1, CDH2, TUBB6, ZYX, MAGI2, TCF7L2
	Semaphorin Signaling in Neurons	16	29.6	<.001	ITGB1, FYN, CFL1, RHOC, DPYSL3, DIRAS3, RAC1, LIMK1, MET, SEMA3A, PAK1, PAK3, RHOF, ARHGAP1, FNBP1, SEMA7A
	Semaphorin Signaling in Neurons	15	27.8	<.001	ITGB1, PLXNA1, CFL1, RHOC, DPYSL3, RAC1, LIMK1, SEMA3A, RHOG, PAK3, CFL2, RND3, RHOU, ARHGAP1, SEMA7A
**Cancer**	Role of Tissue Factor in Cancer	20	15.9	.003	ITGB1, PDIA2, PIK3C2B, FYN, CFL1, CASP3, RRAS, RAC1, PLAUR, KRAS, JAK2, LIMK1, ITGA3, PAK1, GNA15, PIK3CG, ITGAV, PLCB1, GNA13, ITGB5
	Role of Tissue Factor in Cancer	19	15.1	.011	TP53, ITGB1, PDIA2, CFL1, CASP3, RRAS, RAC1, PLAUR, VEGFC, MAPK13, FRK, LIMK1, CFL2, PLCB1, PIK3R2, RPS6KA1, ITGB5, MMP1, FGF5
**Apoptosis, cancer**	PTEN Signaling	20	14.4	.008	MAST2, ITGB1, TGFBR1, CASP3, RRAS, FGFR1, ITGA2, RAC1, BMPR2, ITGA5, KRAS, CNKSR3, INPP5D, FGFR3, ITGA3, CBL, INPP5F, PIK3CG, ITGA4, PDGFRB
	PTEN Signaling	20	14.4	.013	MAST2, ITGB1, TGFBR1, CASP3, RRAS, ILK, RAC1, ITGA5, FGFR2, CNKSR3, IGF2R, PRKCZ, SHC1, CDKN1A, PIK3R2, INSR, GSK3B, KDR, MAGI2, MAGI3

*The percentage of genes within a functional category is presented as the ratio between the number of genes differentially expressed in the GEP assigned to a canonical pathway and the total number of genes which are annotated for that same pathway. ‡p-value corrected for multiple hypothesis testing using the false discovery rate method of Benjamini and Hochberg. Data regarding canonical pathways involved in GEP-A subgroup of tumors are shaded in grey.
