# Supplementary material for: Identification and characterization of the gene expression profiles for protein coding and non-coding RNAs of pancreatic ductal adenocarcinomas
Source: Oncotarget. 2015 May 22;6(22):19070–86. doi: 10.18632/oncotarget.4233 (PMC4662476; doi:10.18632/oncotarget.4233)
Supplement: Supplementary file 6 [file oncotarget-06-19070-s006.pdf]

SUPPLEMENTARY TABLE 5. Most representative canonical pathways involved in the GEP-A subgroup of PDAC tumors (n=24) as identified through analysis of the GEP of coding and non-coding RNAs.

	Canonical Pathways	N. of genes	Gene %*	Corrected p-value‡	Gene ID
Cellular and/or Humoral Immune Response	Dendritic Cell Maturation	34	16.9	<.001	B2M, PLCB2, ICAM1, HLA-A, HLA-DMB, JAK2, HLA-DQB1, TREM2, FCGR2B, COL1A2, PLCD3, PIK3CG, HLA-DRA, COL10A1, PLCB1, LY75, STAT1, HLA-DMA, PIK3C2B, FCGR2A, HLA-B, CD58, TLR9, COL1A1, TLR4, IL18, CD80, IL1RN, FSCN1, IGHG4, FCER1G, STAT2, IRF8, COL3A1
	NF-kB Signaling	25	14.1	.011	PIK3C2B, TGFBR1, RRAS, FGFR1, HDAC1, BMPR2, EGF, TBK1, KRAS, IRAK3, TLR9, FGFR3, TLR4, IL18, BCL10, CARD11, IL1RN, PIK3CG, TLR6, PELI1, FCER1G, CASP8, MAP4K4, TNFSF13B, PDGFRB
	CD28 Signaling in T Helper Cells	22	16.5	.001	HLA-DMA, ACTR2, FYN, PIK3C2B, PTPN6, ARPC1B, RAC1, HLA-DMB, HLA-DQB1, PTPRC, PAK1, ACTR3, ARPC1A, CD80, CARD11, BCL10, PIK3CG, HLA-DRA, ITPR3, FCER1G, VAV1, LCP2
	CTLA4 Signaling in Cytotoxic T Lymphocytes	17	17.9	.003	B2M, AP2B1, FYN, PIK3C2B, PTPN6, AP2M1, HLA-A, HLA-B, CLTB, JAK2, CD80, PIK3CG, FCER1G, AP1S3, PPP2R1B, LCP2, PTPN22
	Antigen Presentation Pathway	17	4.5	<.001	B2M, HLA-DMA, PSMB9, HLA-A, HLA-B, CIITA, HLA- DMB, PSMB8, CD74, TAP1, HLA-DPA1, MR1, NLRC5, HLA-DRA, HLA-F, TAP2, HLA-E
	Communication between Immune Cells†	16	15.7	.005	B2M, HLA-A, HLA-B, TLR9, CXCL10, TLR4, IL18, CD80, IL1RN, TLR6, HLA-DRA, IGHG4, FCER1G, HLA-F, HLA-E, TNFSF13B
	Crosstalk between Dendritic Cells and Natural Killer Cells	16	15.2	.012	IL2RG, HLA-A, ACTA2, HLA-B, TLN1, TLR9, TREM2, FAS, TLR4, IL18, CAMK2D, CD80, FSCN1, HLA-DRA, HLA-F, HLA-E
	T Helper Cell Differentiation	15	21.4	.002	HLA-DMA, IL2RG, TGFBR1, IFNGR2, HLA-DMB, HLA-DQB1, RORC, IL18, CD80, TGFB1, HLA-DRA, IL10RA, FCER1G, IL2RA, STAT1
	Allograft Rejection Signaling	14	23	<.001	B2M, HLA-DMA, HLA-A, HLA-B, HLA-DMB, HLA-DQB1, HLA-DPA1, FAS, CD80, HLA-DRA, IGHG4, FCER1G, HLA-F, HLA-E
	TREM1 Signaling	14	16.1	.011	ITGB1, ICAM1, NLRP1, CIITA, ITGA5, JAK2, FCGR2B, TLR9, TLR4, NLRC5, IL18, TLR6, NOD1, ITGAX
	Graft-versus-Host Disease Signaling	13	26.5	<.001	HLA-DMA, HLA-A, HLA-B, HLA-DMB, HLA-DQB1, FAS, IL18, CD80, IL1RN, HLA-DRA, FCER1G, HLA-F, HLA-E
	Autoimmune Thyroid Disease Signaling	12	21.4	.001	HLA-DMA, CD80, HLA-A, HLA-DRA, HLA-B, IGHG4, FCER1G, HLA-DMB, HLA-DQB1, HLA-F, FAS, HLA-E
	MSP-RON Signaling Pathway	11	21.6	.006	F11, ITGB2, TLR4, PIK3C2B, KLKB1, KLK1, ITGAM, PIK3CG, ACTA2, MST1, JAK2
	Complement System	9	26.5	.007	C1R, CD55, C1S, CFB, C1QC, C1QA, C5, C1QB, CFH
	Interferon Signaling	9	25	.008	IFNGR2, STAT2, PSMB8, JAK2, STAT1, IFNAR2, TAP1, BAK1, IRF1
Intracellular and Second Messenger Signaling	ERK/MAPK Signaling	28	13.5	.006	FYN, PLA2G10, KRAS, TLN1, KSR1, EIF4EBP1, MYC, ELF4, PAK1, ITGA3, ETS2, PIK3CG, MKNK1, STAT1, ITGA4, ITGB1, PPARG, ETS1, PIK3C2B, RRAS, ITGA2, PLA2G1B, RAC1, ITGA5, PRKAR2B, PAK3, PPP2R1B, ELK3
	Tec Kinase Signaling	27	14.8	<.001	FYN, DIRAS3, ACTA2, JAK2, FAS, GNB4, PAK1, ITGA3, GNA15, PIK3CG, GNA13, STAT1, RHOF, ITGA4, ITGB1, TNFRSF21, PIK3C2B, RHOC, ITGA2, ITGA5, BTK, TLR4, PAK3, FCER1G, STAT2, VAV1, FNBP1
	Rac Signaling	23	18.1	<.001	ITGB1, ACTR2, PIK3C2B, NOX4, CFL1, ARPC1B, RRAS, ITGA2, RAC1, ITGA5, KRAS, PARD6A, LIMK1, ITGA3, PAK1, ACTR3, ARPC1A, PAK3, PIK3CG, NCF2, CYBB, PIP4K2A, ITGA4
	PAK Signaling	20	18.2	<.001	ITGB1, PIK3C2B, CFL1, CASP3, RRAS, ITGA2, RAC1, ITGA5, MYLK, KRAS, EPHA3, PDGFC, LIMK1, MYL9, ITGA3, PAK1, PAK3, PIK3CG, ITGA4, PDGFRB
	Regulation of Actin-based Motility by Rho	18	19.8	<.001	ACTR2, CFL1, ARPC1B, RHOC, DIRAS3, ACTA2, RAC1, MYLK, LIMK1, MYL9, PAK1, WIPF1, ARPC1A, ACTR3, PAK3, RHOF, PIP4K2A, FNBP1
	Actin Nucleation by ARP-WASP Complex	17	25.4	<.001	ITGB1, ACTR2, ARPC1B, RHOC, RRAS, DIRAS3, ITGA2, RAC1, ITGA5, KRAS, ITGA3, WIPF1, ACTR3, ARPC1A, RHOF, FNBP1, ITGA4
Organismal Growth and Development, and/or Neurotransmitters and Other NS Signaling	Ephrin Receptor Signaling	32	15.5	<.001	FYN, ARPC1B, EGF, EPHA4, KRAS, JAK2, PDGFC, LIMK1, EFNB2, GNB4, ITGA3, PAK1, ACTR3, GNA15, PIK3CG, EFNA5, GNA13, ITGA4, ITGB1, ACTR2, CFL1, CXCR4, RRAS, ITGA2, RAC1, ITGA5, EPHA3, WIPF1, ARPC1A, PAK3, ADAM10, MAP4K4
	Paxillin Signaling	26	22.6	<.001	ACTA2, KRAS, TLN1, PTPN12, PAK1, ITGA3, ITGA11, PIK3CG, ITGAV, ITGB4, VCL, ITGB5, ACTN1, ITGA4, ITGB1, PIK3C2B, RRAS, ITGA2, RAC1, ITGA5, ITGB2, ITGAM, PAK3, ITGA1, ITGB6, ITGAX
	Caveolar-mediated Endocytosis Signaling	23	27.7	<.001	B2M, ITGB1, FYN, HLA-A, HLA-B, ITGA2, ACTA2, EGF, ITGA5, ITGB2, CD55, ITGA3, ALB, ITGAM, FLNA, ITGA11, ITGAV, ITGA1, ITGB4, ITGB6, ITGB5, ITGA4, ITGAX
	Agrin Interactions at Neuromuscular Junction	18	26.1	<.001	ITGB1, RRAS, ACTA2, ITGA2, RAC1, ITGA5, NRG4, KRAS, LAMC1, ITGB2, PAK1, ITGA3, PAK3, ERBB4, LAMB1, ITGA1, CTTN, ITGA4
	Ephrin A Signaling	12	22.2	.004	FYN, PIK3C2B, PAK1, CFL1, EFNA5, PIK3CG, ADAM10, RAC1, VAV1, EPHA4,

					EPHA3, LIMK1
Cellular Growth, Proliferation and Development	Integrin Signaling	45	22	<.001	RAP2B, FYN, ARHGAP26, ARPC1B, DIRAS3, ACTA2, KRAS, TLN1, MYLK, ITGA3, PAK1, ACTR3, ITGA11, PIK3CG, ITGAV, VCL, ITGB4, RHOF, ACTN1, ITGB5, ITGA4, ITGB1, ACTR2, PIK3C2B, TSPAN5, ASAP1, RRAS, RHOC, ITGA2, RAC1, ITGA5, TTN, MYL9, ITGB2, WIPF1, ITGAM, ARPC1A, PAK3, TSPAN1, ITGA1, CAPN2, ITGB6, CTTN, FNBP1, ITGAX
	Cdc42 Signaling	29	18.8	<.001	B2M, ARPC1B, HLA-A, HLA-DMB, MYLK, HLA-DQB1, PARD6A, HLA-DPA1, LIMK1, PAK1, ITGA3, ACTR3, HLA-DRA, HLA-F, ITGA4, ITGB1, HLA-DMA, ACTR2, CFL1, ITGA2, HLA-B, ITGA5, MYL9, WIPF1, ARPC1A, PAK3, FCER1G, VAV1, HLA-E
	FAK Signaling	20	19	<.001	ITGB1, PIK3C2B, FYN, ARHGAP26, ASAP1, RRAS, ACTA2, ITGA2, RAC1, EGF, ITGA5, KRAS, TLN1, ITGA3, PAK1, PAK3, PIK3CG, CAPN2, VCL, ITGA4
	Regulation of Cellular Mechanics by Calpain Protease	13	18.3	.003	ITGB1, CCNA2, ITGA3, RRAS, ITGA2, EGF, ITGA5, CAPN2, KRAS, TLN1, VCL, ACTN1, ITGA4
Cellular Stress and Injury	Hepatic Fibrosis / Hepatic Stellate Cell Activation	30	19.6	<.001	TGFBR1, ICAM1, FN1, ACTA2, EGF, PDGFC, FAS, COL1A2, TGFB1, CYP2E1, TIMP1, STAT1, IL1RAP, TIMP2, AGT, PDGFRB, FGFR1, IFNGR2, IGFBP5, MMP2, IFNAR2, MET, MYL9, COL1A1, TLR4, IGFBP3, IL10RA, MYH9, EDNRA, COL3A1
	Atherosclerosis Signaling	25	18.4	<.001	ICAM1, PLA2G10, PLA2R1, PDGFC, PLA2G7, COL1A2, LYZ, TGFB1, COL10A1, SERPINA1, ITGA4, PLA2G16, CXCR4, PLA2G1B, SELPLG, APOL1, APOC1, ITGB2, COL1A1, ALB, IL18, IL1RN, RARRES3, ALOX5, COL3A1
	Coagulation System	10	26.3	.003	F11, KLKB1, F2R, F8, PROS1, PLAUR, SERPINA1, PLAU, TFPI, PLAT
	Intrinsic Prothrombin Activation Pathway	9	25	.003	F11, COL1A2, COL1A1, KLKB1, KLK1, F8, PROS1, COL10A1, COL3A1
Cancer	Glioma Invasiveness Signaling	16	24.6	<.001	PIK3C2B, F2R, RHOC, RRAS, DIRAS3, PLAUR, KRAS, MMP2, TIMP1, PIK3CG, ITGAV, PLAU, RHOF, FNBP1, ITGB5, TIMP2
Chronic inflammatory autoinmune disease	Role of Macrophages, Fibroblasts and Endothelial Cells in Rheumatoid Arthritis	39	12	.006	PLCB2, ICAM1, FN1, CSNK1A1, KRAS, JAK2, PDGFC, PRSS3, MYC, PLCD3, ROR2, CAMK2D, TGFB1, DKK3, PIK3CG, DKK2, SFRP5, PLCB1, IL1RAP, TNFSF13B, PIK3C2B, SFRP4, RRAS, RAC1, C5, IRAK3, TLR9, IL7, IL16, TLR4, IL18, IL1RN, TLR6, IGHG4, LEF1, DKK1, LRP1, WNT5A, IRAK2

Specific GEP-A pathways were defined as those pathways which were significantly associated with GEP-A tumors but not GEP-B tumors, and/or those which showed at least two-fold more significantly association in GEP-A vs. GEP-B; *the percentage of genes within a pathway is presented as the ratio between the number of genes differentially expressed in the GEP assigned to a canonical pathway and the total number of genes which are annotated for that same pathway. ‡ p-value corrected for multiple hypothesis testing using the false discovery rate method of Benjamini and Hochberg; NS: Nervous System; † refers to innate and adaptive immune cells.
