# Supplementary material for: Identification and characterization of the gene expression profiles for protein coding and non-coding RNAs of pancreatic ductal adenocarcinomas
Source: Oncotarget. 2015 May 22;6(22):19070–86. doi: 10.18632/oncotarget.4233 (PMC4662476; doi:10.18632/oncotarget.4233)
Supplement: Supplementary file 7 [file oncotarget-06-19070-s007.pdf]

SUPPLEMENTARY TABLE 6. Most representative canonical pathways involved in the GEP-B subgroup of PDAC tumors (n=3) as identified through analysis of the GEP of coding and non-coding RNAs.

	Canonical Pathways	N. of genes	Gene %*	Corrected p-value[‡]	Gene ID
Cellular Growth, Proliferation and Development	Sertoli Cell-Sertoli Cell Junction Signaling	36	18.6	<.001	CLDN11, ILK, MAP3K5, MAPK13, CLDN7, TUBB, OCLN, CLDN4, PPAP2B, CGN, TUBA1C, GSK3B, ACTC1, RAB8B, ACTN1, ITGB1, PLS1, CLDN10, GUCY1A3, RRAS, MAP3K13, TUBB2A, MAP3K1, RAC1, ITGA5, TUBA1B, F11R, CDH1, PRKAR2B, TUBB6, JAM3, CLDN1, ZAK, MAP2K3, MAGI2, CLDN3
	Interleukin Signaling	34	17.1	<.001	FN1, SNAI2, MYL6B, ILK, HIF1A, TGFB1I1, RHOG, CFL2, PPAP2B, RHOU, PIK3R2, GSK3B, ITGB4, TMSB10/TMSB4X, ACTC1, DSP, ITGB5, ACTN1, ITGB1, CFL1, CASP3, RHOC, SNAI1, FERMT2, VIM, VEGFC, MYL9, CDH1, RND3, FLNC, IRS1, KRT18, PTGS2, ITGB6
	Germ Cell-Sertoli Cell Junction Signaling	30	18	<.001	TGFBR1, ILK, MAP3K5, TUBB, LIMK1, RHOG, TGFB1, PPAP2B, RHOU, TUBA1C, PIK3R2, ACTC1, RAB8B, ACTN1, ITGB1, PLS1, RHOC, RRAS, MAP3K13, TUBB2A, MAP3K1, RAC1, TUBA1B, CDH2, CDH1, RND3, TUBB6, PAK3, ZYX, MAP2K3
Apoptosis, cancer	Tight Junction Signaling	25	15.1	.005	CLDN10, F2RL2, CLDN11, TGFBR1, MYL6B, RAC1, CLDN7, CNKSR3, PRKCZ, OCLN, MYL9, F11R, PRKAR2B, CLDN4, JAM3, CLDN1, LLGL1, TGFB1, CGN, VCL, MAGI2, INADL, ACTC1, CLDN3, TNFRSF11B
Cancer	HER-2 Signaling in Breast Cancer	17	2.7	.002	TP53, ITGB1, PRKCQ, RRAS, EGF, PARD6B, ERBB3, MMP2, MAP3K5, PRKCZ, CDKN1A, PRKCH, ITGB4, PIK3R2, GSK3B, ITGB6, ITGB5
	Bladder Cancer Signaling	16	17	.013	TP53, MMP7, TFDP1, MMP3, RRAS, MMP14, EGF, VEGFC, MMP2, FGF1, CDH1, CDKN1A, MMP11, FGF7, MMP1, FGF5

Specific GEP-B pathways were defined as those pathways which were significantly associated with GEP-B tumors but not GEP-A tumors, and/or those which showed at least two-fold more significantly association in GEP-B vs. GEP-A. *the percentage of genes within a pathway is presented as the ratio between the number of genes differentially expressed in the GEP assigned to a canonical pathway and the total number of genes which are annotated for that same pathway. [‡] p-value corrected for multiple hypothesis testing using the false discovery rate method of Benjamini and Hochberg.
