# Supplementary material for: Identification and characterization of the gene expression profiles for protein coding and non-coding RNAs of pancreatic ductal adenocarcinomas
Source: Oncotarget. 2015 May 22;6(22):19070–86. doi: 10.18632/oncotarget.4233 (PMC4662476; doi:10.18632/oncotarget.4233)
Supplement: Supplementary file 8 [file oncotarget-06-19070-s008.pdf]

SUPPLEMENTARY TABLE 7: Receiver operating characteristic (ROC) curve analysis specifically performed for genes previously selected based on the predictive algorithms which contributed most to the discrimination of the GEP subgroups A (24 tumors vs 3 tumors and 5 non-tumoral tissues) and B (3 tumors vs 24 tumors and 5 non-tumoral tissues) of PDAC tumor tissues.

Gene name	Gene ID	Selected marker for GEP subgroup*	Fold change GEP-A vs Non-T	Fold Change GEP-B vs Non-T	AUC (GEP-A)	p-value	CI (95%)		AUC (GEP-B)	p-value	CI (95%)	
ABCF2	ENSG00000033050		1.4	2.8	0.63	0.294	0.290	0.960	1.00	0.005	1.000	1.000
ABR	ENSG00000159842		2.1	NS	0.64	0.240	0.316	0.964	0.97	0.009	0.902	1.031
AC0071821	ENSG00000119686		2.6	NS	1.00	0.000	0.999	1.000	0.10	0.024	-0.013	0.213
**AC0093111**	ENSG00000242113	**B**	**NS**	**5.8**	0.31	**0.110**	**0.100**	**0.520**	**1.00**	**0.005**	**1.000**	**1.000**
AC0114792	ENSG00000167642		NS	-6.3	0.93	0.000	0.845	1.015	1.00	0.005	0.000	0.000
AC0245752	ENSG00000105520		NS	3.3	0.47	0.801	0.192	0.748	1.00	0.005	1.000	1.000
AC0788893	ENSG00000225422		3.7	7.2	0.62	0.313	0.290	0.953	1.00	0.005	1.000	1.000
AC0793371	ENSG00000235734		-2.1	NS	1,00	0.000	-0.019	0.049	0.87	0.039	0.742	0.992
AC0797801	ENSG00000236654		4.0	9.8	0.63	0.294	0.308	0.942	0.97	0.009	0.902	1.031
AC0995221	ENSG00000183900		NS	3.9	0.21	0.015	0.026	0.394	1.00	0.005	1.000	1.000
**AC0997591**	ENSG00000105889	**B**	**NS**	**11.0**	0.59	**0.450**	**0.269**	**0.911**	**1.00**	**0.005**	**1.000**	**1.000**
AC1078831	ENSG00000173517		2.0	4.3	0.62	0.334	0.284	0.946	1.00	0.005	1.000	1.000
AC1080651	ENSG00000138640		-2.2	-3.8	0.63	0.240	0.036	0.684	1.00	0.009	-0.031	0.098
AC1151021	ENSG00000247116		NS	-2.7	0.52	0.900	0.226	0.804	1.00	0.005	0.000	0.000
ACP5	ENSG00000102575		2.8	NS	1.00	0.000	0.979	1.011	0.12	0.033	-0.017	0.262
**ACSL5**	ENSG00000197142	**A**	**7.0**	**NS**	0.97	**0.000**	**0.897**	**1.033**	**0.02**	**0.007**	**-0.027**	**0.072**
**ACTC1**	ENSG00000159251	**B**	**NS**	**12.5**	0.31	**0.110**	**0.094**	**0.526**	**0.97**	**0.009**	**0.902**	**1.031**
ACTR1A	ENSG00000138107		1.6	2.9	0.61	0.355	0.281	0.939	1.00	0.005	1.000	1.000
ADAM12	ENSG00000148848		5.9	28.6	0.58	0.529	0.259	0.891	1.00	0.005	1.000	1.000
**ADAM28**	ENSG00000042980	**A**	**7.2**	**NS**	0.98	**0.000**	**0.923**	**1.027**	**0.00**	**0.005**	**0.000**	**0.000**
**ADAMTS5**	ENSG00000154736	**B**	**NS**	**9.5**	0.48	**0.834**	**0.197**	**0.753**	**1.00**	**0.005**	**1.000**	**1.000**
ADRBK1	ENSG00000173020		2.3	NS	1.00	0.000	0.999	1.000	0.11	0.028	-0.002	0.225
AEN	ENSG00000181026		NS	2.3	0.15	0.003	0.016	0.284	0.97	0.009	0.902	1.031
AGPAT1	ENSG00000204310		1.7	3.2	0.62	0.313	0.293	0.947	0.99	0.006	0.956	1.022
AGR2	ENSG00000106541		5.3	NS	0.95	0.000	0.875	1.025	0.02	0.007	-0.027	0.072
AHR	ENSG00000106546		3.4	NS	0.96	0.000	0.883	1.037	0.18	0.069	0.041	0.314
AK1	ENSG00000106992		NS	3.1	0.56	0.614	0.248	0.872	1.00	0.005	1.000	1.000
**AK5**	**ENSG00000154027**	**B**	**NS**	**48.4**	0.43	**0.529**	**0.161**	**0.689**	**1.00**	**0.005**	**1.000**	**1.000**

AKAP9	ENSG00000127914		NS	-2.6	0.50	1.000	0.223	0.777	1.00	0.009	-0.031	0.098
ALDH1L2	ENSG00000136010		-3.4	NS	0.98	0.000	-0.020	0.060	0.91	0.021	0.811	1.011
ALOX5	ENSG00000012779		2.5	-6.2	0.98	0.000	0.923	1.027	1.00	0.005	0.000	0.000
ALOX5AP	ENSG00000132965		4.0	NS	0.97	0.000	0.907	1.023	0.00	0.005	0.000	0.000
**ALPK2**	ENSG00000198796	**B**	**NS**	**12.0**	0.56	**0.614**	**0.251**	**0.869**	**1.00**	**0.005**	**1.000**	**1.000**
ALS2CR4	ENSG00000155755		NS	2.8	0.55	0.674	0.250	0.850	0.98	0.007	0.928	1.027
AMT	ENSG00000145020		-1.7	-3.0	0.62	0.378	0.068	0.722	1.00	0.005	0.000	0.000
ANAPC11	ENSG00000141552		NS	2.4	0.62	0.334	0.290	0.940	0.99	0.006	0.956	1.022
ANK3	ENSG00000151150		NS	-7.8	0.59	0.450	0.319	0.861	1.00	0.005	0.000	0.000
**ANO1**	ENSG00000131620	**A**	**6.8**	**NS**	0.98	**0.000**	**0.937**	**1.023**	**0.00**	**0.005**	**0.000**	**0.000**
ANXA5	ENSG00000164111		1.9	3.2	0.62	0.334	0.284	0.946	1.00	0.005	1.000	1.000
AOX1	ENSG00000138356		-9.2	NS	1,00	0.000	0.000	0.000	0.87	0.039	0.742	0.992
AP0006542	ENSG00000150687		2.4	8.8	0.60	0.425	0.272	0.919	1.00	0.005	1.000	1.000
**AP0009262**	ENSG00000166250	**B**	**NS**	**9.3**	0.59	**0.475**	**0.265**	**0.905**	**1.00**	**0.005**	**1.000**	**1.000**
AP1M1	ENSG00000072958		1.5	3.1	0.62	0.334	0.284	0.946	1.00	0.005	1.000	1.000
AP1S3	ENSG00000152056		3.4	NS	1.00	0.000	0.979	1.011	0.06	0.012	-0.024	0.136
AP2A1	ENSG00000196961		1.8	4.0	0.63	0.294	0.290	0.960	1.00	0.005	1.000	1.000
AP2M1	ENSG00000161203		2.2	4.7	0.63	0.294	0.290	0.960	1.00	0.005	1.000	1.000
AP2S1	ENSG00000042753		NS	2.7	0.57	0.556	0.254	0.886	1.00	0.005	1.000	1.000
APOBEC3C	ENSG00000244509		2.2	5.4	0.62	0.334	0.284	0.946	1.00	0.005	1.000	1.000
**APOL1**	ENSG00000100342	**A**	**10.1**	**NS**	0.96	**0.000**	**0.883**	**1.037**	**0.16**	**0.052**	**0.025**	**0.286**
ARHGAP15	ENSG00000075884		NS	-3.0	0.97	0.000	0.910	1.030	1.00	0.006	-0.022	0.044
ARHGAP23	ENSG00000225485		NS	2.2	0.36	0.240	0.123	0.597	1.00	0.005	1.000	1.000
ARHGAP26	ENSG00000145819		2.7	NS	0.98	0.000	0.928	1.022	0.11	0.028	-0.001	0.223
**ARHGDIB**	ENSG00000111348	**A**	**4.3**	**NS**	0.96	**0.000**	**0.890**	**1.030**	**0.02**	**0.007**	**-0.027**	**0.072**
**ARL4D**	ENSG00000175906	**B**	**NS**	**4.4**	0.23	**0.021**	**0.039**	**0.411**	**1.00**	**0.005**	**1.000**	**1.000**
ASAP1	ENSG00000153317		2.1	NS	0.61	0.378	0.281	0.932	1.00	0.005	1.000	1.000
ASPHD2	ENSG00000128203		2.9	NS	1.00	0.000	0.999	1.000	0.17	0.060	0.033	0.300
ATF7IP2	ENSG00000166669		NS	-6.0	0.45	0.644	0.137	0.753	1.00	0.005	0.000	0.000
ATP6V0D1	ENSG00000159720		1.7	3.7	0.62	0.334	0.284	0.946	1.00	0.005	1.000	1.000
ATP6V1F	ENSG00000128524		1.7	3.7	0.62	0.313	0.290	0.953	1.00	0.005	1.000	1.000
ATP8A1	ENSG00000124406		NS	-11.5	0.40	0.401	0.075	0.725	1.00	0.005	0.000	0.000
ATP8B2	ENSG00000143515		2.0	5.4	0.63	0.294	0.290	0.960	1.00	0.005	1.000	1.000
AXL	ENSG00000167601		2.3	5.0	0.62	0.313	0.290	0.953	1.00	0.005	1.000	1.000
BAT2	ENSG00000204469		NS	2.0	0.60	0.401	0.292	0.908	0.97	0.009	0.902	1.031
BAX	ENSG00000087088		NS	2.6	0.60	0.425	0.272	0.918	1.00	0.005	1.000	1.000

BCAR3	ENSG00000137936		1.7	3.1	0.61	0.355	0.281	0.939	1.00	0.005	1.000	1.000
BCL9L	ENSG00000186174		1.4	2.6	0.61	0.378	0.278	0.933	1.00	0.005	1.000	1.000
BIRC3	ENSG00000023445		4.0	NS	0.96	0.000	0.883	1.037	0.03	0.009	-0.031	0.098
BLNK	ENSG00000095585		NS	-4.4	0.71	0.078	0.502	0.918	1.00	0.006	-0.022	0.044
**BMPER**	ENSG00000164619	**B**	**NS**	**6.8**	0.09	**0.000**	**-0.014**	**0.184**	**1.00**	**0.005**	**1.000**	**1.000**
**BNC1**	ENSG00000169594	**B**	**NS**	**10.3**	0.25	**0.032**	**0.032**	**0.458**	**1.00**	**0.005**	**1.000**	**1.000**
BRI3	ENSG00000164713		NS	2.6	0.42	0.501	0.165	0.675	1.00	0.005	1.000	1.000
BTN3A1	ENSG00000026950		2.1	NS	0.96	0.000	0.883	1.037	0.17	0.060	0.033	0.300
BUD31	ENSG00000106245		NS	2.1	0.62	0.313	0.290	0.953	1.00	0.005	1.000	1.000
**BVES**	ENSG00000112276	**B**	**NS**	**8.0**	0.43	**0.556**	**0.162**	**0.698**	**1.00**	**0.005**	**1.000**	**1.000**
**C10orf72**	ENSG00000165633	**B**	**NS**	**5.1**	0.55	**0.705**	**0.241**	**0.849**	**1.00**	**0.005**	**1.000**	**1.000**
C10orf81	ENSG00000148735		NS	-10.4	0.64	0.257	0.415	0.855	1.00	0.009	-0.031	0.098
C11orf24	ENSG00000171067		1.9	3.6	0.61	0.378	0.278	0.933	1.00	0.005	1.000	1.000
C11orf59	ENSG00000149357		1.8	4.6	0.62	0.334	0.284	0.946	1.00	0.005	1.000	1.000
C11orf68	ENSG00000175573		NS	2.2	0.44	0.614	0.171	0.709	1.00	0.005	1.000	1.000
C12orf24	ENSG00000204856		NS	3.1	0.58	0.529	0.274	0.876	0.97	0.009	0.902	1.031
C13orf27	ENSG00000151287		NS	2.8	0.57	0.585	0.259	0.871	0.99	0.006	0.956	1.022
C14orf105	ENSG00000100557		NS	-17.9	0.41	0.450	0.089	0.731	1.00	0.005	0.000	0.000
C14orf37	ENSG00000139971		1.9	3.5	0.61	0.355	0.281	0.939	1.00	0.005	1.000	1.000
C16orf35	ENSG00000103148		NS	2.2	0.48	0.834	0.181	0.769	1.00	0.005	1.000	1.000
C17orf28	ENSG00000167861		-2.1	-7.5	0.59	0.401	0.075	0.725	1.00	0.005	0.000	0.000
C19orf40	ENSG00000131944		NS	2.0	0.48	0.834	0.179	0.771	1.00	0.005	1.000	1.000
C1orf126	ENSG00000175147		-2.7	-8.3	0.63	0.294	0.040	0.710	1.00	0.005	0.000	0.000
C1QA	ENSG00000173372		3.4	NS	0.95	0.000	0.874	1.026	0.02	0.007	-0.027	0.072
C1QB	ENSG00000173369		4.0	NS	0.96	0.000	0.882	1.028	0.02	0.007	-0.027	0.072
C1QC	ENSG00000159189		3.7	NS	0.96	0.000	0.889	1.021	0.00	0.005	0.000	0.000
**C20orf103**	ENSG00000125869	**A**	**7.9**	**NS**	0.97	**0.000**	**0.903**	**1.027**	**0.10**	**0.024**	**-0.007**	**0.207**
C20orf74	ENSG00000188559		NS	-4.6	0.73	0.053	0.493	0.967	1.00	0.005	0.000	0.000
C2orf18	ENSG00000213699		NS	2.5	0.56	0.644	0.245	0.865	1.00	0.005	1.000	1.000
C3orf52	ENSG00000114529		-3.7	-12.5	0.61	0.334	0.054	0.716	1.00	0.005	0.000	0.000
**C3orf55**	ENSG00000174899	**B**	**NS**	**5.6**	0.30	**0.093**	**0.067**	**0.533**	**1.00**	**0.005**	**1.000**	**1.000**
C6	ENSG00000039537		NS	-23.6	0.41	0.450	0.111	0.709	0.97	0.010	-0.027	0.116
C6orf130	ENSG00000124596		NS	-2.4	0.54	0.737	0.260	0.820	1.00	0.005	0.000	0.000
C7	ENSG00000112936		NS	-165.9	0.55	0.705	0.273	0.817	1.00	0.005	0.000	0.000
C7orf46	ENSG00000188732		NS	-6.5	0.43	0.529	0.110	0.740	1.00	0.005	0.000	0.000
C9orf119	ENSG00000175854		NS	2.3	0.42	0.475	0.162	0.668	1.00	0.005	1.000	1.000

C9orf72	ENSG00000147894		NS	-3.6	0.53	0.834	0.246	0.804	1.00	0.005	0.000	0.000
CA2	ENSG00000104267		NS	-12.4	0.66	0.193	0.426	0.884	1.00	0.005	0.000	0.000
CACNB3	ENSG00000167535		2.3	3.5	0.63	0.294	0.290	0.960	1.00	0.005	1.000	1.000
CADPS2	ENSG00000081803		NS	-6.3	0.44	0.585	0.122	0.748	1.00	0.005	0.000	0.000
CALHM2	ENSG00000138172		NS	3.4	0.48	0.834	0.193	0.757	1.00	0.005	1.000	1.000
CALU	ENSG00000128595		2.7	6.4	0.62	0.313	0.290	0.953	1.00	0.005	1.000	1.000
CAMK2D	ENSG00000145349		2.4	3.7	0.60	0.401	0.274	0.926	1.00	0.005	1.000	1.000
CARD11	ENSG00000198286		3.5	NS	1.00	0.000	0.999	1.000	0.04	0.010	-0.031	0.120
CARM1	ENSG00000142453		NS	2.6	0.60	0.425	0.290	0.901	0.97	0.009	0.902	1.031
CASP8	ENSG00000064012		2.1	NS	0.97	0.000	0.910	1.030	0.07	0.015	-0.027	0.161
CBFB	ENSG00000067955		2.1	NS	0.97	0.000	0.910	1.030	0.17	0.060	0.033	0.300
**CCBE1**	ENSG00000183287	**B**	**NS**	**13.6**	0.05	**0.000**	**-0.021**	**0.111**	**1.00**	**0.005**	**1.000**	**1.000**
CCDC7	ENSG00000216937		NS	-2.1	0.70	0.093	0.494	0.906	1.00	0.009	-0.031	0.098
CCL21	ENSG00000137077		NS	-4.9	0.77	0.023	0.597	0.943	1.00	0.005	0.000	0.000
CD248	ENSG00000174807		2.6	18.3	0.58	0.501	0.262	0.898	1.00	0.005	1.000	1.000
CD24L4	ENSG00000185275		NS	-136.1	0.49	0.900	0.192	0.778	1.00	0.005	0.000	0.000
CD276	ENSG00000103855		2.1	5.8	0.63	0.294	0.290	0.960	1.00	0.005	1.000	1.000
CD53	ENSG00000143119		3.4	-9.3	0.96	0.000	0.897	1.023	1.00	0.005	0.000	0.000
CD58	ENSG00000116815		2.7	NS	0.96	0.000	0.883	1.037	0.20	0.091	0.057	0.343
CD63	ENSG00000135404		1.7	2.3	0.60	0.401	0.274	0.926	1.00	0.005	1.000	1.000
CD74	ENSG00000019582		3.3	NS	0.98	0.000	0.923	1.027	0.00	0.005	0.000	0.000
CDC25B	ENSG00000101224		2.5	6.3	0.63	0.294	0.290	0.960	1.00	0.005	1.000	1.000
CDH1	ENSG00000039068		NS	-32.3	0.86	0.003	0.720	0.990	1.00	0.005	0.000	0.000
**CDH13**	ENSG00000140945	**B**	**NS**	**14.1**	0.57	**0.585**	**0.253**	**0.877**	**1.00**	**0.005**	**1.000**	**1.000**
**CDH3**	**ENSG00000062038**	**A**	**8.2**	**NS**	1.00	**0.000**	**0.999**	**1.000**	**0.03**	**0.009**	**-0.037**	**0.104**
**CEACAM1**	ENSG00000079385	**A**	**7.0**	**NS**	0.97	**0.000**	**0.910**	**1.030**	**0.07**	**0.015**	**-0.057**	**0.190**
**CEACAM5**	ENSG00000105388	**A**	**58.1**	**NS**	0.96	**0.000**	**0.882**	**1.028**	**0.07**	**0.015**	**-0.027**	**0.161**
CEP70	ENSG00000114107		NS	-5.1	0.41	0.425	0.081	0.729	1.00	0.005	0.000	0.000
**CERCAM**	ENSG00000167123	**B**	**NS**	**4.6**	0.57	**0.556**	**0.256**	**0.884**	**1.00**	**0.005**	**1.000**	**1.000**
CFH	ENSG00000000971		3.5	NS	0.96	0.000	0.883	1.037	0.14	0.045	0.019	0.270
CFHR1	ENSG00000244414		2.0	NS	0.96	0.000	0.883	1.037	0.17	0.060	0.035	0.299
CFTR	ENSG00000001626		NS	-131.0	0.39	0.355	0.067	0.713	1.00	0.006	-0.022	0.044
CHKA	ENSG00000110721		-1.7	-3.2	0.62	0.313	0.047	0.713	1.00	0.005	0.000	0.000
**CHST11**	ENSG00000171310	**A**	**4.3**	**NS**	0.99	**0.000**	**0.965**	**1.016**	**0.19**	**0.080**	**0.050**	**0.328**
CIITA	ENSG00000179583		3.0	NS	0.97	0.000	0.896	1.033	0.07	0.015	-0.023	0.156
CKLF	ENSG00000217555		4.0	NS	0.97	0.000	0.910	1.030	0.20	0.091	0.057	0.343

CLDN1	ENSG00000163347		NS	-10.8	0.76	0.029	0.574	0.946	1.00	0.005	0.000	0.000
CLDN10	ENSG00000134873		NS	-20.6	0.41	0.450	0.105	0.715	0.97	0.009	-0.031	0.098
**CLDN11**	ENSG00000013297	**B**	**NS**	**21.0**	0.56	**0.614**	**0.250**	**0.870**	**1.00**	**0.005**	**1.000**	**1.000**
CLDN7	ENSG00000181885		NS	-14.2	0.77	0.026	0.581	0.949	1.00	0.005	0.000	0.000
CLEC2D	ENSG00000069493		3.3	NS	0.96	0.000	0.883	1.037	0.10	0.024	-0.007	0.207
CLIC4	ENSG00000169504		1.9	3.1	0.63	0.294	0.290	0.960	1.00	0.005	1.000	1.000
**CLIP3**	ENSG00000105270	**B**	**NS**	**4.3**	0.48	**0.867**	**0.191**	**0.769**	**1.00**	**0.005**	**1.000**	**1.000**
CLMN	ENSG00000165959		NS	-19.3	0.46	0.705	0.147	0.763	1.00	0.005	0.000	0.000
CMTM6	ENSG00000091317		2.2	NS	0.97	0.000	0.910	1.030	0.18	0.069	0.042	0.313
CMTM8	ENSG00000170293		-2.1	-4.3	0.63	0.294	0.040	0.710	1.00	0.005	0.000	0.000
CNIH4	ENSG00000143771		1.8	3.5	0.64	0.257	0.314	0.956	0.97	0.009	0.902	1.031
CNKSR1	ENSG00000142675		NS	-3.7	0.60	0.401	0.350	0.850	1.00	0.005	0.000	0.000
CNRIP1	ENSG00000119865		NS	2.8	0.56	0.614	0.251	0.869	1.00	0.005	1.000	1.000
CNTNAP1	ENSG00000108797		NS	2.7	0.27	0.048	0.058	0.472	1.00	0.005	1.000	1.000
**COL6A1**	ENSG00000142156	**B**	**NS**	**5.6**	0.61	**0.378**	**0.278**	**0.932**	**1.00**	**0.005**	**1.000**	**1.000**
COL6A2	ENSG00000142173		NS	3.3	0.55	0.674	0.244	0.856	1.00	0.005	1.000	1.000
**COLEC12**	ENSG00000158270	**B**	**NS**	**8.4**	0.51	**0.933**	**0.219**	**0.801**	**1.00**	**0.005**	**1.000**	**1.000**
CORO1C	ENSG00000110880		2.4	4.4	0.63	0.294	0.290	0.960	1.00	0.005	1.000	1.000
**CORO2A**	ENSG00000106789	**A**	**4.4**	**NS**	1.00	**0.000**	**0.999**	**1.000**	**0.06**	**0.012**	**-0.024**	**0.136**
CPA4	ENSG00000128510		-2.1	NS	0.99	0.000	-0.017	0.047	0.98	0.007	0.928	1.027
CRAT	ENSG00000095321		-2.1	NS	0.96	0.000	-0.027	0.097	0.88	0.033	0.762	0.994
CSF2RA	ENSG00000198223		2.5	NS	0.97	0.000	0.910	1.021	0.00	0.005	0.000	0.000
CSGLCAT	ENSG00000033100		NS	2.4	0.59	0.475	0.265	0.905	1.00	0.005	1.000	1.000
**CST1**	**ENSG00000170373**	**A**	**23.3**	**NS**	1.00	**0.000**	**0.999**	**1.000**	**0.07**	**0.015**	**-0.023**	**0.156**
CTSA	ENSG00000064601		2.1	4.0	0.62	0.334	0.284	0.946	1.00	0.005	1.000	1.000
**CTSE**	ENSG00000196188	**A**	**13.0**	**NS**	0.97	**0.000**	**0.897**	**1.033**	**0.02**	**0.007**	**-0.027**	**0.072**
CTSK	ENSG00000143387		6.4	29.5	0.60	0.425	0.272	0.919	1.00	0.005	1.000	1.000
**CTSL1**	ENSG00000135047	**B**	**NS**	**6.2**	0.58	**0.501**	**0.262**	**0.898**	**1.00**	**0.005**	**1.000**	**1.000**
CTSS	ENSG00000163131		3.8	NS	0.96	0.000	0.877	1.033	0.03	0.009	-0.031	0.098
CTTNBP2	ENSG00000077063		-2.3	-4.9	0.63	0.334	0.054	0.716	1.00	0.005	0.000	0.000
CXADR	ENSG00000154639		NS	-16.8	0.74	0.048	0.543	0.927	1.00	0.005	0.000	0.000
**CXCL10**	ENSG00000169245	**A**	**9.7**	**NS**	0.97	**0.000**	**0.907**	**1.023**	**0.00**	**0.005**	**0.000**	**0.000**
CXCL16	ENSG00000161921		3.3	NS	0.97	0.000	0.897	1.033	0.02	0.007	-0.027	0.072
**CXCR4**	ENSG00000121966	**A**	**4.5**	**NS**	0.98	**0.000**	**0.923**	**1.027**	**0.00**	**0.005**	**0.000**	**0.000**
CXorf38	ENSG00000185753		2.1	NS	0.98	0.000	0.937	1.023	0.14	0.045	0.002	0.287
CYB5R3	ENSG00000100243		1.7	4.5	0.62	0.334	0.284	0.946	1.00	0.005	1.000	1.000

CYBB	ENSG00000165168		4.5	-7.6	0.95	0.000	0.875	1.025	1.00	0.006	-0.022	0.044
CYTH3	ENSG00000008256		2.4	4.4	0.61	0.355	0.281	0.939	1.00	0.005	1.000	1.000
CYTH4	ENSG00000100055		2.2	NS	0.98	0.000	0.941	1.019	0.00	0.005	0.000	0.000
**DAPP1**	**ENSG00000070190**	**A**	**5.8**	**NS**	1.00	**0.000**	**0.999**	**1.000**	**0.03**	**0.009**	**-0.028**	**0.095**
DBN1	ENSG00000113758		NS	2.6	0.63	0.275	0.311	0.949	0.97	0.009	0.902	1.031
DCP2	ENSG00000172795		2.1	NS	1.00	0.000	0.999	1.000	0.06	0.012	-0.035	0.146
DCTN1	ENSG00000204843		1.3	2.3	0.62	0.334	0.284	0.946	1.00	0.005	1.000	1.000
DDX60	ENSG00000137628		4.2	NS	0.94	0.000	0.843	1.027	0.08	0.017	-0.019	0.175
**DGKH**	ENSG00000102780	**A**	**4.3**	**NS**	0.96	**0.000**	**0.883**	**1.037**	**0.20**	**0.091**	**0.057**	**0.343**
DKK2	ENSG00000155011		3.1	24.2	0.63	0.294	0.290	0.960	1.00	0.005	1.000	1.000
DKK3	ENSG00000050165		3.1	6.1	0.61	0.355	0.290	0.933	0.99	0.006	0.956	1.022
DNAJC13	ENSG00000138246		2.0	2.7	0.62	0.313	0.290	0.953	1.00	0.005	1.000	1.000
**DOCK2**	ENSG00000134516	**A**	**4.1**	**NS**	1.00	**0.000**	**0.979**	**1.011**	**0.00**	**0.005**	**0.000**	**0.000**
DOCK7	ENSG00000116641		NS	2.3	0.61	0.355	0.300	0.922	0.97	0.009	0.902	1.031
DRAM1	ENSG00000136048		2.0	12.4	0.60	0.401	0.274	0.926	1.00	0.005	1.000	1.000
DSC2	ENSG00000134755		NS	-10.2	0.91	0.001	0.801	1.009	1.00	0.005	0.000	0.000
**DSEL**	ENSG00000171451	**B**	**NS**	**9.4**	0.40	**0.378**	**0.128**	**0.662**	**1.00**	**0.005**	**1.000**	**1.000**
DSG2	ENSG00000046604		NS	-15.0	0.94	0.000	0.847	1.024	1.00	0.005	0.000	0.000
DSP	ENSG00000096696		NS	-6.6	0.40	0.401	0.075	0.725	1.00	0.005	0.000	0.000
DULLARD	ENSG00000175826		NS	2.1	0.62	0.313	0.290	0.953	1.00	0.005	1.000	1.000
**DUSP14**	ENSG00000161326	**B**	**NS**	**9.0**	0.37	**0.275**	**0.109**	**0.631**	**1.00**	**0.005**	**1.000**	**1.000**
DUSP16	ENSG00000111266		NS	-3.1	0.49	0.933	0.196	0.784	1.00	0.005	0.000	0.000
DYNLL1	ENSG00000088986		1.6	2.6	0.62	0.334	0.290	0.940	0.99	0.006	0.956	1.022
**DYRK3**	ENSG00000143479	**B**	**NS**	**4.2**	0.21	**0.015**	**0.044**	**0.376**	**1.00**	**0.005**	**1.000**	**1.000**
**ECM1**	ENSG00000143369	**B**	**NS**	**11.6**	0.63	**0.294**	**0.308**	**0.942**	**0.97**	**0.009**	**0.902**	**1.031**
EFEMP2	ENSG00000172638		2.0	6.7	0.61	0.378	0.278	0.932	1.00	0.005	1.000	1.000
EFS	ENSG00000100842		NS	2.1	0.56	0.614	0.251	0.869	1.00	0.005	1.000	1.000
**EGLN3**	ENSG00000129521	**A**	**5.4**	**NS**	1.00	**0.000**	**0.999**	**1.000**	**0.00**	**0.005**	**0.000**	**0.000**
EHD1	ENSG00000110047		1.6	2.8	0.63	0.294	0.290	0.960	1.00	0.005	1.000	1.000
EHD2	ENSG00000024422		1.6	5.8	0.62	0.313	0.290	0.953	1.00	0.005	1.000	1.000
EHF	ENSG00000135373		NS	-32.9	0.85	0.003	0.720	0.990	1.00	0.005	0.000	0.000
EIF1B	ENSG00000114784		NS	2.1	0.58	0.501	0.281	0.880	0.97	0.009	0.902	1.031
EIF4A2	ENSG00000156976		NS	-2.9	0.51	0.966	0.219	0.791	1.00	0.005	0.000	0.000
ELOVL1	ENSG00000066322		2.0	NS	0.59	0.475	0.263	0.907	1.00	0.005	1.000	1.000
ELOVL7	ENSG00000164181		NS	-8.1	0.48	0.834	0.179	0.771	1.00	0.005	0.000	0.000
EMILIN2	ENSG00000132205		NS	3.7	0.56	0.614	0.251	0.869	1.00	0.005	1.000	1.000

EMP3	ENSG00000142227		2.5	10.4	0.63	0.294	0.290	0.960	1.00	0.005	1.000	1.000
**EMX2**	ENSG00000170370	**B**	**NS**	**4.8**	0.08	**0.000**	**-0.015**	**0.175**	**1.00**	**0.005**	**1.000**	**1.000**
ENTPD1	ENSG00000138185		3.9	NS	0.97	0.000	0.910	1.030	0.01	0.006	-0.022	0.044
ENTPD3	ENSG00000168032		-3.2	NS	0.60	0.313	0.065	0.695	0.03	0.009	-0.031	0.098
EPB41L4B	ENSG00000095203		-4.3	-7.8	0.63	0.294	0.040	0.710	1.00	0.005	0.000	0.000
EPCAM	ENSG00000119888		NS	-60.8	0.85	0.004	0.710	0.983	1.00	0.005	0.000	0.000
**EPSTI1**	ENSG00000133106	**A**	**4.3**	**NS**	0.97	**0.000**	**0.903**	**1.027**	**0.16**	**0.052**	**0.027**	**0.284**
ERAP1	ENSG00000164307		2.2	NS	0.96	0.000	0.883	1.037	0.16	0.052	0.027	0.284
ERBB3	ENSG00000065361		NS	-5.0	0.83	0.006	0.672	0.978	1.00	0.005	0.000	0.000
ESRP1	ENSG00000104413		NS	-22.8	0.68	0.130	0.455	0.905	1.00	0.005	0.000	0.000
ETNK1	ENSG00000139163		2.1	-1.4	0.99	0.000	0.951	1.019	1.00	0.005	0.000	0.000
F11R	ENSG00000158769		NS	-11.3	0.96	0.000	0.890	1.021	1.00	0.005	0.000	0.000
FABP3	ENSG00000121769		4.4	46.4	0.62	0.334	0.284	0.946	1.00	0.005	1.000	1.000
**FADS1**	ENSG00000149485	**B**	**NS**	**10.4**	0.50	**1.000**	**0.207**	**0.793**	**1.00**	**0.005**	**1.000**	**1.000**
FADS2	ENSG00000134824		3.1	15.9	0.61	0.355	0.281	0.939	1.00	0.005	1.000	1.000
FAM105A	ENSG00000145569		NS	-12.0	0.49	0.900	0.177	0.793	1.00	0.005	0.000	0.000
FAM114A1	ENSG00000197712		2.0	3.1	0.60	0.425	0.272	0.919	1.00	0.005	1.000	1.000
FAM127A	ENSG00000134590		1.8	4.8	0.63	0.294	0.290	0.960	1.00	0.005	1.000	1.000
FAM127B	ENSG00000203950		NS	3.1	0.60	0.425	0.272	0.918	1.00	0.005	1.000	1.000
FAM160A1	ENSG00000164142		-3.0	-17.0	0.63	0.334	0.054	0.716	1.00	0.005	0.000	0.000
**FAM180A**	ENSG00000189320	**B**	**NS**	**13.2**	0.40	**0.401**	**0.152**	**0.648**	**1.00**	**0.005**	**1.000**	**1.000**
FAM26E	ENSG00000178033		3.4	7.1	0.60	0.425	0.272	0.918	1.00	0.005	1.000	1.000
FAM3B	ENSG00000183844		NS	-47.8	0.39	0.355	0.061	0.719	1.00	0.005	0.000	0.000
FAM49B	ENSG00000153310		2.6	NS	1.00	0.000	0.999	1.000	0.08	0.017	-0.019	0.175
FAM55C	ENSG00000144815		NS	3.4	0.61	0.378	0.281	0.932	1.00	0.005	1.000	1.000
FAM57A	ENSG00000167695		NS	3.4	0.57	0.585	0.254	0.876	1.00	0.005	1.000	1.000
FAM69A	ENSG00000154511		3.2	10.7	0.63	0.294	0.290	0.960	1.00	0.005	1.000	1.000
FAP	ENSG00000078098		7.6	NS	0.60	0.401	0.275	0.925	1.00	0.005	1.000	1.000
**FBLN2**	ENSG00000163520	**B**	**NS**	**8.2**	0.57	**0.585**	**0.254**	**0.876**	**1.00**	**0.005**	**1.000**	**1.000**
FBXO34	ENSG00000178974		2.1	NS	0.96	0.000	0.883	1.037	0.10	0.024	-0.007	0.207
**FCER1G**	ENSG00000158869	**A**	**4.0**	**NS**	0.97	**0.000**	**0.907**	**1.023**	**0.00**	**0.005**	**0.000**	**0.000**
FECH	ENSG00000066926		NS	2.2	0.57	0.585	0.251	0.879	1.00	0.005	1.000	1.000
**FERMT1**	**ENSG00000101311**	**A**	**6.7**	**NS**	1.00	**0.000**	**0.999**	**1.000**	**0.02**	**0.007**	**-0.030**	**0.075**
FERMT3	ENSG00000149781		2.2	NS	0.97	0.000	0.910	1.030	0.01	0.006	-0.022	0.044
**FEZ1**	ENSG00000149557	**B**	**NS**	**6.2**	0.60	**0.401**	**0.274**	**0.926**	**1.00**	**0.005**	**1.000**	**1.000**
FGD6	ENSG00000180263		2.8	NS	1.00	0.000	0.999	1.000	0.01	0.006	-0.022	0.044

**FGF5**	ENSG00000138675	**B**	**NS**	**13.9**	0.28	**0.065**	**0.041**	**0.519**	**1.00**	**0.005**	**1.000**	**1.000**
FGFR1	ENSG00000077782		-2.2	NS	0.99	0.000	-0.021	0.081	0.89	0.028	0.770	1.008
FIBP	ENSG00000172500		1.5	2.6	0.61	0.355	0.290	0.934	0.99	0.006	0.956	1.022
FICD	ENSG00000198855		-2.5	NS	0.99	0.000	-0.011	0.021	0.87	0.039	0.745	0.988
FKBP5	ENSG00000096060		NS	-14.4	0.50	0.966	0.207	0.783	1.00	0.005	0.000	0.000
**FLNC**	ENSG00000128591	**B**	**NS**	**6.7**	0.61	**0.378**	**0.278**	**0.932**	**1.00**	**0.005**	**1.000**	**1.000**
FMNL3	ENSG00000161791		3.5	NS	0.99	0.000	0.964	1.016	0.19	0.080	0.050	0.328
**FREQ**	ENSG00000107130	**B**	**NS**	**5.0**	0.51	**0.966**	**0.214**	**0.796**	**1.00**	**0.005**	**1.000**	**1.000**
FRK	ENSG00000111816		NS	-3.3	0.88	0.001	0.765	0.995	1.00	0.007	-0.027	0.072
FRMD6	ENSG00000139926		3.3	13.1	0.62	0.334	0.284	0.946	1.00	0.005	1.000	1.000
**FST**	ENSG00000134363	**B**	**NS**	**15.5**	0.37	**0.257**	**0.123**	**0.607**	**1.00**	**0.005**	**1.000**	**1.000**
FUNDC2	ENSG00000165775		-2.0	NS	0.97	0.000	-0.022	0.082	0.90	0.024	0.793	1.007
**FXYD3**	ENSG00000089356	**A**	**4.6**	**NS**	0.96	**0.000**	**0.897**	**1.023**	**0.00**	**0.005**	**0.000**	**0.000**
FXYD6	ENSG00000137726		NS	-3.7	0.78	0.019	0.583	0.977	1.00	0.005	0.000	0.000
**FYB**	ENSG00000082074	**A**	**4.9**	**NS**	0.96	**0.000**	**0.883**	**1.037**	**0.03**	**0.009**	**-0.031**	**0.098**
G6PD	ENSG00000160211		1.8	5.1	0.63	0.294	0.290	0.960	1.00	0.005	1.000	1.000
GADD45A	ENSG00000116717		2.1	3.5	0.61	0.378	0.278	0.932	1.00	0.005	1.000	1.000
GALC	ENSG00000054983		2.2	NS	0.93	0.000	0.823	1.027	0.13	0.039	0.012	0.255
GALNT3	ENSG00000115339		NS	-8.6	0.92	0.000	0.828	1.012	1.00	0.005	0.000	0.000
**GALNTL2**	ENSG00000131386	**B**	**NS**	**4.3**	0.40	**0.401**	**0.137**	**0.663**	**1.00**	**0.005**	**1.000**	**1.000**
GATM	ENSG00000171766		-5.0	-181.4	0.60	0.378	0.068	0.722	1.00	0.005	0.000	0.000
**GBAP**	ENSG00000160766	**B**	**NS**	**4.0**	0.62	**0.334**	**0.284**	**0.946**	**1.00**	**0.005**	**1.000**	**1.000**
**GBE1**	ENSG00000114480	**B**	**NS**	**5.5**	0.37	**0.275**	**0.120**	**0.620**	**1.00**	**0.005**	**1.000**	**1.000**
**GBP4**	ENSG00000162654	**A**	**4.5**	**NS**	0.98	**0.000**	**0.923**	**1.027**	**0.00**	**0.005**	**0.000**	**0.000**
GDAP1	ENSG00000104381		-2.2	-3.4	0.62	0.294	0.052	0.698	0.98	0.007	-0.027	0.072
GDPD3	ENSG00000102886		2.9	NS	1.00	0.000	0.999	1.000	0.14	0.045	0.019	0.270
GEMIN6	ENSG00000152147		1.5	2.5	0.64	0.257	0.314	0.956	0.97	0.009	0.902	1.031
GGT5	ENSG00000099998		2.1	NS	0.96	0.000	0.895	1.025	0.01	0.006	-0.022	0.044
GIMAP2	ENSG00000106560		2.7	NS	0.94	0.000	0.843	1.027	0.10	0.024	-0.006	0.206
GJC1	ENSG00000182963		2.3	4.9	0.63	0.275	0.311	0.949	0.97	0.009	0.902	1.031
GLI3	ENSG00000106571		3.0	11.0	0.60	0.425	0.272	0.918	1.00	0.005	1.000	1.000
GMPR	ENSG00000137198		-3.0	NS	0.99	0.000	-0.011	0.021	0.90	0.024	0.793	1.007
GNA15	ENSG00000060558		2.8	NS	0.96	0.000	0.883	1.037	0.04	0.010	-0.027	0.116
GNG5P2	ENSG00000133136		2.1	NS	0.63	0.275	0.305	0.955	0.98	0.007	0.928	1.027
GNPDA1	ENSG00000113552		1.7	4.5	0.60	0.425	0.270	0.920	1.00	0.005	1.000	1.000
GNPTAB	ENSG00000111670		1.9	3.8	0.61	0.355	0.281	0.939	1.00	0.005	1.000	1.000

GOLGA8B	ENSG00000215252		-4.7	-23.9	0.63	0.294	0.040	0.710	1.00	0.005	0.000	0.000
GOLM1	ENSG00000135052		2.6	NS	0.97	0.000	0.910	1.030	0.11	0.028	-0.008	0.230
**GPR1**	ENSG00000183671	**B**	**NS**	**5.1**	0.27	**0.048**	**0.051**	**0.479**	**1.00**	**0.005**	**1.000**	**1.000**
GPR124	ENSG00000020181		2.1	4.7	0.57	0.556	0.257	0.883	1.00	0.005	1.000	1.000
GPR176	ENSG00000166073		1.6	8.3	0.64	0.257	0.308	0.962	0.98	0.007	0.928	1.027
**GPX2**	ENSG00000176153	**A**	**13.8**	**NS**	0.98	**0.000**	**0.923**	**1.027**	**0.00**	**0.005**	**0.000**	**0.000**
GPX8	ENSG00000164294		4.9	11.7	0.63	0.294	0.290	0.960	1.00	0.005	1.000	1.000
GRAMD3	ENSG00000155324		2.0	5.1	0.59	0.475	0.265	0.905	1.00	0.005	1.000	1.000
GREM1	ENSG00000166923		13.3	50.9	0.63	0.294	0.290	0.960	1.00	0.005	1.000	1.000
GRHL2	ENSG00000083307		NS	-7.1	0.47	0.769	0.165	0.765	1.00	0.005	0.000	0.000
GRINA	ENSG00000178719		1.5	3.2	0.62	0.334	0.284	0.946	1.00	0.005	1.000	1.000
GRLF1	ENSG00000160007		NS	2.1	0.51	0.933	0.214	0.806	1.00	0.005	1.000	1.000
GRN	ENSG00000030582		2.1	3.9	0.62	0.334	0.284	0.946	1.00	0.005	1.000	1.000
GSK3B	ENSG00000082701		1.8	2.9	0.63	0.294	0.290	0.960	1.00	0.005	1.000	1.000
GUCY1A3	ENSG00000164116		NS	-8.1	0.73	0.059	0.528	0.922	1.00	0.009	-0.031	0.098
HABP4	ENSG00000130956		NS	2.7	0.48	0.834	0.188	0.762	1.00	0.005	1.000	1.000
HCFC1	ENSG00000172534		1.5	2.4	0.63	0.294	0.307	0.943	0.97	0.009	0.902	1.031
HCLS1	ENSG00000180353		2.5	NS	0.97	0.000	0.910	1.030	0.01	0.006	-0.022	0.044
HCP5	ENSG00000206337		3.2	NS	0.97	0.000	0.916	1.024	0.00	0.005	0.000	0.000
HEATR5A	ENSG00000129493		1.8	2.4	0.63	0.294	0.290	0.960	1.00	0.005	1.000	1.000
HEXA	ENSG00000213614		1.9	4.0	0.60	0.401	0.274	0.926	1.00	0.005	1.000	1.000
HGD	ENSG00000113924		NS	-18.8	0.41	0.450	0.106	0.714	1.00	0.009	-0.031	0.098
HLAB	ENSG00000234745		2.7	NS	0.96	0.000	0.883	1.037	0.19	0.080	0.050	0.328
HLADMA	ENSG00000204257		3.0	NS	0.95	0.000	0.862	1.028	0.08	0.017	-0.018	0.173
HLADMB	ENSG00000242574		3.8	NS	0.97	0.000	0.916	1.024	0.00	0.005	0.000	0.000
HLADPA1	ENSG00000231389		3.1	NS	0.96	0.000	0.895	1.025	0.01	0.006	-0.022	0.044
HLADRA	ENSG00000204287		3.5	NS	0.97	0.000	0.910	1.030	0.01	0.006	-0.022	0.044
HLAF	ENSG00000204642		2.9	NS	0.96	0.000	0.883	1.037	0.10	0.024	-0.011	0.211
HLF	ENSG00000108924		NS	-4.6	0.40	0.378	0.091	0.699	0.97	0.010	-0.027	0.116
**HMOX1**	ENSG00000100292	**B**	**NS**	**11.0**	0.51	**0.933**	**0.212**	**0.808**	**1.00**	**0.005**	**1.000**	**1.000**
HNF1B	ENSG00000108753		NS	-8.7	0.46	0.705	0.156	0.754	1.00	0.007	-0.027	0.072
HNF4G	ENSG00000164749		NS	-3.6	0.83	0.006	0.686	0.974	1.00	0.005	0.000	0.000
HOMER2	ENSG00000103942		-5.5	-14.0	0.63	0.275	0.039	0.701	1.00	0.006	-0.022	0.044
HOOK1	ENSG00000134709		-2.3	-12.4	0.62	0.313	0.047	0.713	1.00	0.005	0.000	0.000
HOXA9	ENSG00000078399		NS	3.5	0.29	0.078	0.071	0.509	1.00	0.005	1.000	1.000
HOXC5	ENSG00000172789		NS	2.5	0.49	0.933	0.204	0.776	1.00	0.005	1.000	1.000

**HOXC6**	ENSG00000197757	**B**	**NS**	**8.6**	0.53	**0.801**	**0.232**	**0.828**	**1.00**	**0.005**	**1.000**	**1.000**
HSD17B14	ENSG00000087076		NS	3.8	0.39	0.334	0.141	0.629	1.00	0.005	1.000	1.000
HSPB3	ENSG00000169271		NS	2.6	0.19	0.008	0.026	0.344	1.00	0.005	1.000	1.000
HTRA1	ENSG00000166033		2.5	6.1	0.58	0.529	0.259	0.891	1.00	0.005	1.000	1.000
ICA1	ENSG00000003147		NS	-8.7	0.46	0.737	0.158	0.762	1.00	0.005	0.000	0.000
IFI27	ENSG00000165949		7.3	NS	0.95	0.000	0.874	1.026	0.07	0.015	-0.039	0.173
IFI30	ENSG00000216490		3.6	NS	0.99	0.000	0.950	1.020	0.03	0.009	-0.037	0.104
IFITM3	ENSG00000142089		2.3	4.4	0.61	0.355	0.281	0.939	1.00	0.005	1.000	1.000
**IGHG4**	ENSG00000211892	**A**	**7.7**	**NS**	0.96	**0.000**	**0.890**	**1.021**	**0.00**	**0.005**	**0.000**	**0.000**
IKBIP	ENSG00000166130		1.9	5.9	0.62	0.313	0.290	0.953	1.00	0.005	1.000	1.000
**IL13RA2**	**ENSG00000123496**	**B**	**NS**	**21.9**	0.44	**0.614**	**0.165**	**0.714**	**1.00**	**0.005**	**1.000**	**1.000**
IL18	ENSG00000150782		3.2	NS	0.95	0.000	0.867	1.023	0.03	0.009	-0.028	0.095
**IL2RG**	ENSG00000147168	**A**	**8.2**	**NS**	0.97	**0.000**	**0.897**	**1.033**	**0.02**	**0.007**	**-0.027**	**0.072**
INADL	ENSG00000132849		NS	-5.4	0.60	0.425	0.341	0.849	1.00	0.005	0.000	0.000
INPP5D	ENSG00000168918		3.3	NS	0.99	0.000	0.951	1.019	0.00	0.005	0.000	0.000
IPO13	ENSG00000117408		1.5	3.5	0.61	0.378	0.278	0.933	1.00	0.005	1.000	1.000
IQGAP2	ENSG00000145703		NS	-40.4	0.47	0.769	0.163	0.767	1.00	0.005	0.000	0.000
IRF6	ENSG00000117595		NS	-6.6	0.77	0.026	0.586	0.944	1.00	0.005	0.000	0.000
IRF8	ENSG00000140968		3.2	NS	0.96	0.000	0.885	1.025	0.01	0.006	-0.022	0.044
**ITGA1**	ENSG00000213949	**A**	**4.4**	**NS**	0.96	**0.000**	**0.883**	**1.037**	**0.17**	**0.060**	**0.030**	**0.303**
**ITGA2**	ENSG00000164171	**A**	**13.9**	**NS**	0.98	**0.000**	**0.937**	**1.023**	**0.16**	**0.052**	**0.017**	**0.294**
ITGA5	ENSG00000161638		2.4	6.4	0.61	0.378	0.281	0.932	1.00	0.005	1.000	1.000
ITGAX	ENSG00000140678		2.6	NS	0.95	0.000	0.862	1.028	0.03	0.009	-0.031	0.098
ITGB2	ENSG00000160255		2.9	NS	0.97	0.000	0.916	1.024	0.00	0.005	0.000	0.000
ITGB4	ENSG00000132470		4.8	-3.0	0.98	0.000	0.923	1.027	1.00	0.005	0.000	0.000
ITGB5	ENSG00000082781		3.6	6.6	0.63	0.294	0.290	0.960	1.00	0.005	1.000	1.000
**ITGB6**	ENSG00000115221	**A**	**9.7**	**-6.6**	0.97	**0.000**	**0.910**	**1.030**	1.00	**0.006**	**-0.022**	**0.044**
ITPR2	ENSG00000123104		NS	-3.7	0.62	0.313	0.364	0.876	1.00	0.005	0.000	0.000
ITPRIP	ENSG00000148841		NS	3.7	0.53	0.834	0.228	0.822	1.00	0.005	1.000	1.000
ITSN2	ENSG00000198399		-1.6	-3.5	0.61	0.294	0.058	0.692	1.00	0.009	-0.031	0.098
JAM3	ENSG00000166086		2.3	4.3	0.62	0.334	0.284	0.946	1.00	0.005	1.000	1.000
KANK2	ENSG00000197256		NS	2.5	0.54	0.769	0.235	0.835	1.00	0.005	1.000	1.000
**KCNK2**	**ENSG00000082482**	**B**	**NS**	**33.4**	0.50	**1.000**	**0.213**	**0.786**	**1.00**	**0.005**	**1.000**	**1.000**
KCNQ1	ENSG00000053918		NS	-2.8	0.41	0.450	0.089	0.731	1.00	0.005	0.000	0.000
KCTD10	ENSG00000110906		1.9	3.7	0.63	0.294	0.290	0.960	1.00	0.005	1.000	1.000
KIAA1199	ENSG00000103888		6.1	64.3	0.63	0.294	0.290	0.960	1.00	0.005	1.000	1.000

KIAA1324	ENSG00000116299		-4.2	-45.5	0.61	0.294	0.052	0.698	1.00	0.007	-0.027	0.072
KIAA1539	ENSG00000005238		1.6	2.9	0.63	0.294	0.290	0.960	1.00	0.005	1.000	1.000
KIAA1949	ENSG00000146112		2.1	4.1	0.63	0.294	0.290	0.960	1.00	0.005	1.000	1.000
KIRREL	ENSG00000183853		2.6	5.7	0.62	0.334	0.284	0.946	1.00	0.005	1.000	1.000
KLHL6	ENSG00000172578		3.0	NS	0.96	0.000	0.895	1.025	0.07	0.015	-0.029	0.162
KPNA2	ENSG00000182481		3.6	8.5	0.64	0.257	0.314	0.956	0.97	0.009	0.902	1.031
KRT18	ENSG00000111057		NS	-16.2	0.75	0.040	0.558	0.932	1.00	0.005	0.000	0.000
**KRT19**	**ENSG00000171345**	**A**	**9.4**	**NS**	1.00	**0.000**	**0.999**	**1.000**	**0.00**	**0.005**	**0.000**	**0.000**
KRT8	ENSG00000170421		NS	-25.9	0.82	0.007	0.670	0.970	1.00	0.005	0.000	0.000
**KYNU**	ENSG00000115919	**A**	**6.1**	**NS**	0.96	**0.000**	**0.883**	**1.037**	**0.03**	**0.009**	**-0.031**	**0.098**
LAD1	ENSG00000159166		NS	-3.3	0.95	0.000	0.876	1.024	1.00	0.005	0.000	0.000
LAMA1	ENSG00000101680		NS	2.8	0.50	0.966	0.222	0.768	0.97	0.009	0.902	1.031
**LAMB3**	ENSG00000196878	**A**	**10.1**	**NS**	0.99	**0.000**	**0.951**	**1.019**	**0.12**	**0.033**	**-0.005**	**0.249**
LAMC1	ENSG00000135862		2.1	5.1	0.63	0.294	0.290	0.960	1.00	0.005	1.000	1.000
**LAMC2**	**ENSG00000058085**	**A**	**21.0**	**NS**	**1.00**	**0.000**	**0.979**	**1.011**	**0.18**	**0.069**	**0.042**	**0.313**
LAPTM5	ENSG00000162511		3.3	-8.5	0.96	0.000	0.882	1.028	1.00	0.007	-0.027	0.072
LARP1B	ENSG00000138709		-2.6	-4.6	0.63	0.294	0.040	0.710	1.00	0.005	0.000	0.000
LASP1	ENSG00000002834		1.9	3.5	0.63	0.294	0.290	0.960	1.00	0.005	1.000	1.000
LASS6	ENSG00000172292		2.1	NS	1.00	0.000	0.999	1.000	0.04	0.010	-0.044	0.133
LAYN	ENSG00000204381		3.8	18.7	0.63	0.294	0.290	0.960	1.00	0.005	1.000	1.000
**LCN2**	ENSG00000148346	**A**	**14.3**	**NS**	0.97	**0.000**	**0.916**	**1.024**	**0.00**	**0.005**	**0.000**	**0.000**
LCP2	ENSG00000043462		3.1	NS	0.96	0.000	0.877	1.033	0.03	0.009	-0.031	0.098
**LDOC1**	ENSG00000182195	**B**	**NS**	**4.1**	0.23	**0.021**	**0.040**	**0.410**	**1.00**	**0.005**	**1.000**	**1.000**
LENG4	ENSG00000125505		NS	2.5	0.52	0.900	0.217	0.813	1.00	0.005	1.000	1.000
LGALS1	ENSG00000100097		3.6	6.9	0.63	0.294	0.290	0.960	1.00	0.005	1.000	1.000
LGALS3BP	ENSG00000108679		2.8	NS	0.96	0.000	0.883	1.037	0.14	0.045	0.013	0.276
LGALS4	ENSG00000171747		NS	-7.3	0.92	0.000	0.818	1.013	1.00	0.009	-0.031	0.098
LGALS9B	ENSG00000170298		2.4	NS	1.00	0.000	0.999	1.000	0.07	0.015	-0.023	0.156
LGALS9C	ENSG00000171916		-2.6	NS	0.57	0.556	0.112	0.748	0.00	0.005	0.000	0.000
LIPG	ENSG00000101670		3.2	NS	1.00	0.000	0.979	1.011	0.06	0.012	-0.026	0.137
LIPH	ENSG00000163898		2.6	-14.4	0.93	0.000	0.838	1.022	1.00	0.009	-0.031	0.098
LLGL2	ENSG00000073350		NS	-3.2	0.66	0.179	0.431	0.889	1.00	0.005	0.000	0.000
LMF2	ENSG00000100258		-2.5	NS	0.99	0.000	-0.016	0.036	0.87	0.039	0.745	0.988
LNX2	ENSG00000139517		NS	-3.6	0.38	0.313	0.047	0.713	1.00	0.005	0.000	0.000
LOX	ENSG00000113083		7.1	40.1	0.60	0.425	0.272	0.918	1.00	0.005	1.000	1.000
LPAR1	ENSG00000198121		NS	3.3	0.22	0.019	0.046	0.394	1.00	0.005	1.000	1.000

LPAR6	ENSG00000139679		2.9	NS	0.96	0.000	0.877	1.033	0.03	0.009	-0.031	0.098
LPCAT2	ENSG00000087253		2.8	NS	0.95	0.000	0.870	1.030	0.19	0.080	0.041	0.337
LRBA	ENSG00000198589		NS	-2.4	0.85	0.004	0.710	0.982	1.00	0.005	0.000	0.000
LRP10	ENSG00000197324		2.3	5.1	0.60	0.401	0.274	0.926	1.00	0.005	1.000	1.000
LRRC1	ENSG00000137269		NS	-2.8	0.92	0.000	0.819	1.011	1.00	0.005	0.000	0.000
LRRC32	ENSG00000137507		2.9	6.7	0.60	0.401	0.274	0.926	1.00	0.005	1.000	1.000
**LRRN4CL**	ENSG00000177363	**B**	**NS**	**5.4**	0.11	**0.001**	**-0.019**	**0.229**	**1.00**	**0.005**	**1.000**	**1.000**
**LSS**	ENSG00000160285	**B**	**NS**	**4.2**	0.22	**0.017**	**0.040**	**0.390**	**1.00**	**0.005**	**1.000**	**1.000**
**LXN**	ENSG00000079257	**A**	**4.5**	**NS**	0.98	**0.000**	**0.923**	**1.027**	**0.00**	**0.005**	**0.000**	**0.000**
**LY75**	ENSG00000054219	**A**	**6.3**	**NS**	0.97	**0.000**	**0.897**	**1.033**	**0.03**	**0.009**	**-0.028**	**0.095**
LYZ	ENSG00000090382		2.5	-108.1	0.94	0.000	0.848	1.022	1.00	0.007	-0.027	0.072
MAFG	ENSG00000197063		NS	2.3	0.56	0.644	0.248	0.862	1.00	0.005	1.000	1.000
MAL2	ENSG00000147676		NS	-26.4	0.96	0.000	0.890	1.021	1.00	0.005	0.000	0.000
**MALL**	ENSG00000144063	**A**	**4.6**	**NS**	0.97	**0.000**	**0.910**	**1.030**	**0.01**	**0.006**	**-0.022**	**0.044**
MAN1B1	ENSG00000177239		NS	2.2	0.46	0.705	0.185	0.725	1.00	0.005	1.000	1.000
MAP1A	ENSG00000166963		2.8	14.5	0.61	0.355	0.281	0.939	1.00	0.005	1.000	1.000
MAP1LC3B2	ENSG00000171471		NS	2.8	0.40	0.378	0.139	0.651	1.00	0.005	1.000	1.000
MAP3K1	ENSG00000095015		NS	-4.9	0.71	0.085	0.483	0.927	1.00	0.005	0.000	0.000
MAP3K13	ENSG00000073803		NS	-5.2	0.41	0.425	0.099	0.711	1.00	0.009	-0.031	0.098
MAP3K5	ENSG00000197442		NS	-4.8	0.41	0.450	0.105	0.715	1.00	0.009	-0.031	0.098
**MAP7D3**	ENSG00000129680	**B**	**NS**	**4.1**	0.58	**0.529**	**0.259**	**0.891**	**1.00**	**0.005**	**1.000**	**1.000**
MARVELD1	ENSG00000155254		2.4	4.4	0.63	0.294	0.290	0.960	1.00	0.005	1.000	1.000
MDGA1	ENSG00000112139		NS	2.5	0.37	0.275	0.127	0.613	1.00	0.005	1.000	1.000
MED31	ENSG00000108590		NS	2.5	0.34	0.166	0.092	0.578	1.00	0.005	1.000	1.000
MEIS1	ENSG00000143995		NS	-3.9	0.48	0.834	0.193	0.757	1.00	0.009	-0.031	0.098
MFAP2	ENSG00000117122		3.4	7.4	0.63	0.294	0.290	0.960	1.00	0.005	1.000	1.000
**MFAP5**	**ENSG00000197614**	**B**	**NS**	**33.0**	0.52	**0.867**	**0.226**	**0.814**	**1.00**	**0.005**	**1.000**	**1.000**
MFSD5	ENSG00000182544		1.7	5.7	0.60	0.425	0.272	0.918	1.00	0.005	1.000	1.000
MGAT4A	ENSG00000071073		NS	-26.8	0.49	0.933	0.199	0.781	1.00	0.005	0.000	0.000
MICB	ENSG00000204516		NS	3.2	0.54	0.737	0.234	0.846	1.00	0.005	1.000	1.000
MLLT1	ENSG00000130382		NS	2.1	0.18	0.007	0.018	0.342	1.00	0.005	1.000	1.000
**MME**	**ENSG00000196549**	**B**	**NS**	**65.9**	0.50	**0.966**	**0.200**	**0.790**	**1.00**	**0.005**	**1.000**	**1.000**
MMP14	ENSG00000157227		6.0	11.8	0.63	0.294	0.290	0.960	1.00	0.005	1.000	1.000
MMP2	ENSG00000087245		6.1	18.2	0.62	0.334	0.284	0.946	1.00	0.005	1.000	1.000
MMP7	ENSG00000137673		3.0	-37.3	0.95	0.000	0.870	1.020	1.00	0.006	-0.022	0.044
MOBKL2A	ENSG00000172081		NS	2.2	0.57	0.556	0.255	0.885	1.00	0.005	1.000	1.000

MPP7	ENSG00000150054		-2.2	-7.2	0.61	0.334	0.054	0.716	1.00	0.005	0.000	0.000
MPZL1	ENSG00000197965		2.5	3.7	0.63	0.294	0.290	0.960	1.00	0.005	1.000	1.000
MPZL2	ENSG00000149573		NS	-18.3	0.93	0.000	0.845	1.015	1.00	0.005	0.000	0.000
**MRGPRF**	ENSG00000172935	**B**	**NS**	**4.5**	0.30	**0.093**	**0.060**	**0.540**	**1.00**	**0.005**	**1.000**	**1.000**
MSC	ENSG00000178860		2.0	5.9	0.58	0.501	0.262	0.898	1.00	0.005	1.000	1.000
MSRB2	ENSG00000148450		1.4	2.5	0.62	0.334	0.284	0.946	1.00	0.005	1.000	1.000
MT1F	ENSG00000198417		-2.7	-12.7	0.62	0.313	0.047	0.713	1.00	0.005	0.000	0.000
**MTMR11**	ENSG00000014914	**A**	**4.7**	**NS**	0.97	**0.000**	**0.897**	**1.033**	**0.19**	**0.080**	**0.050**	**0.328**
**MTSS1L**	ENSG00000132613	**B**	**NS**	**6.8**	0.41	**0.425**	**0.141**	**0.669**	**1.00**	**0.005**	**1.000**	**1.000**
MTUS1	ENSG00000129422		NS	-4.9	0.44	0.585	0.135	0.735	1.00	0.007	-0.027	0.072
**MUC13**	ENSG00000173702	**A**	**13.4**	**NS**	0.96	**0.000**	**0.883**	**1.037**	**0.03**	**0.009**	**-0.031**	**0.098**
**MUC3A**	**ENSG00000169894**	**A**	**9.5**	**NS**	1.00	**0.000**	**0.999**	**1.000**	**0.03**	**0.009**	**-0.028**	**0.095**
MYO1F	ENSG00000142347		2.3	NS	0.98	0.000	0.941	1.020	0.03	0.009	-0.037	0.104
MYO5B	ENSG00000167306		NS	-5.4	0.68	0.141	0.456	0.894	1.00	0.005	0.000	0.000
MYO5C	ENSG00000128833		NS	-21.6	0.41	0.425	0.082	0.728	1.00	0.005	0.000	0.000
NARG1L	ENSG00000172766		-2.8	-5.8	0.63	0.294	0.040	0.710	1.00	0.005	0.000	0.000
**NAV3**	ENSG00000067798	**B**	**NS**	**7.9**	0.57	**0.556**	**0.256**	**0.884**	**1.00**	**0.005**	**1.000**	**1.000**
NCF2	ENSG00000116701		2.9	-3.2	0.97	0.000	0.907	1.023	1.00	0.005	0.000	0.000
NCF4	ENSG00000100365		2.4	NS	0.95	0.000	0.878	1.022	0.02	0.007	-0.030	0.075
**NCKAP1L**	ENSG00000123338	**A**	**4.3**	**NS**	0.98	**0.000**	**0.923**	**1.027**	**0.00**	**0.005**	**0.000**	**0.000**
NDRG2	ENSG00000165795		NS	-7.4	0.50	1.000	0.200	0.800	1.00	0.005	0.000	0.000
NDUFA11	ENSG00000174886		NS	2.0	0.42	0.475	0.145	0.685	1.00	0.005	1.000	1.000
NDUFS4	ENSG00000164258		NS	2.9	0.55	0.705	0.249	0.841	0.98	0.007	0.928	1.027
NEBL	ENSG00000078114		NS	-5.4	0.58	0.529	0.315	0.835	1.00	0.005	0.000	0.000
NEDD8	ENSG00000129559		1.6	3.5	0.62	0.334	0.290	0.940	0.99	0.006	0.956	1.022
**NEK10**	ENSG00000163491	**B**	**NS**	**4.1**	0.44	**0.585**	**0.166**	**0.704**	**1.00**	**0.005**	**1.000**	**1.000**
NET1	ENSG00000173848		2.1	NS	0.99	0.000	0.965	1.016	0.01	0.006	-0.022	0.044
NFATC4	ENSG00000100968		NS	3.1	0.46	0.705	0.178	0.732	1.00	0.005	1.000	1.000
NFE2L3	ENSG00000050344		3.1	NS	1.00	0.000	0.999	1.000	0.00	0.005	0.000	0.000
**NFIX**	ENSG00000008441	**B**	**NS**	**4.3**	0.42	**0.501**	**0.146**	**0.694**	**1.00**	**0.005**	**1.000**	**1.000**
NID1	ENSG00000116962		2.5	5.1	0.62	0.334	0.284	0.946	1.00	0.005	1.000	1.000
**NKIRAS1**	ENSG00000197885	**B**	**NS**	**4.8**	0.44	**0.614**	**0.146**	**0.734**	**1.00**	**0.005**	**1.000**	**1.000**
NLGN2	ENSG00000169992		NS	2.2	0.39	0.334	0.156	0.614	0.97	0.009	0.902	1.031
NMNAT3	ENSG00000163864		-2.0	-3.1	0.63	0.294	0.040	0.710	1.00	0.005	0.000	0.000
NNMT	ENSG00000166741		3.1	6.6	0.60	0.425	0.278	0.912	0.99	0.006	0.956	1.022
NOP10	ENSG00000182117		2.1	4.2	0.64	0.257	0.308	0.962	0.98	0.007	0.928	1.027

NOSTRIN	ENSG00000163072		NS	-15.7	0.41	0.450	0.089	0.731	1.00	0.005	0.000	0.000
NPC2	ENSG00000119655		2.3	3.8	0.61	0.378	0.281	0.932	1.00	0.005	1.000	1.000
NPL	ENSG00000135838		2.7	NS	0.95	0.000	0.862	1.028	0.08	0.017	-0.018	0.173
NPR2	ENSG00000159899		NS	3.9	0.60	0.425	0.272	0.919	1.00	0.005	1.000	1.000
NR5A2	ENSG00000116833		-6.6	-98.2	0.63	0.294	0.040	0.710	1.00	0.005	0.000	0.000
**NRN1**	ENSG00000124785	**B**	**NS**	**13.2**	0.16	**0.004**	**0.017**	**0.304**	**1.00**	**0.005**	**1.000**	**1.000**
NSUN7	ENSG00000179299		-2.4	-5.5	0.63	0.294	0.040	0.710	1.00	0.005	0.000	0.000
NT5DC2	ENSG00000168268		2.2	4.1	0.62	0.313	0.305	0.935	0.97	0.009	0.902	1.031
NTF3	ENSG00000185652		NS	2.9	0.49	0.900	0.193	0.777	1.00	0.005	1.000	1.000
NUP210	ENSG00000132182		NS	-3.0	0.83	0.006	0.686	0.974	1.00	0.005	0.000	0.000
OCLN	ENSG00000197822		NS	-21.7	0.51	0.933	0.223	0.797	1.00	0.005	0.000	0.000
OGFOD1	ENSG00000087263		1.7	2.9	0.63	0.275	0.300	0.961	0.99	0.006	0.956	1.022
OPTN	ENSG00000123240		1.5	2.6	0.58	0.529	0.260	0.890	1.00	0.005	1.000	1.000
ORAI2	ENSG00000160991		NS	2.8	0.62	0.313	0.290	0.953	1.00	0.005	1.000	1.000
**OSBPL10**	**ENSG00000144645**	**A**	**5.5**	**NS**	1.00	**0.000**	**0.999**	**1.000**	**0.17**	**0.060**	**0.033**	**0.300**
**OSR1**	ENSG00000143867	**B**	**NS**	**8.3**	0.07	**0.000**	**-0.016**	**0.146**	**1.00**	**0.005**	**1.000**	**1.000**
**OSR2**	ENSG00000164920	**B**	**NS**	**8.2**	0.36	**0.223**	**0.120**	**0.590**	**1.00**	**0.005**	**1.000**	**1.000**
PACSIN2	ENSG00000100266		-1.9	-3.3	0.61	0.355	0.061	0.719	1.00	0.005	0.000	0.000
PAIP2B	ENSG00000124374		-11.7	-35.3	0.63	0.294	0.040	0.710	1.00	0.005	0.000	0.000
PAN2	ENSG00000135473		-1.5	-3.0	0.59	0.425	0.080	0.730	1.00	0.005	0.000	0.000
**PAPPA**	ENSG00000182752	**B**	**NS**	**18.1**	0.54	**0.769**	**0.234**	**0.836**	**1.00**	**0.005**	**1.000**	**1.000**
PARM1	ENSG00000169116		NS	-13.2	0.43	0.529	0.110	0.740	1.00	0.005	0.000	0.000
PARP14	ENSG00000173193		2.8	NS	0.96	0.000	0.877	1.033	0.11	0.028	-0.001	0.223
**PARP15**	ENSG00000173200	**A**	**4.5**	**NS**	0.98	**0.000**	**0.937**	**1.023**	**0.14**	**0.045**	**0.018**	**0.271**
PARP3	ENSG00000041880		2.8	4.9	0.60	0.401	0.274	0.926	1.00	0.005	1.000	1.000
PARP8	ENSG00000151883		2.0	NS	0.97	0.000	0.907	1.023	0.00	0.005	0.000	0.000
PARVG	ENSG00000138964		2.4	NS	0.95	0.000	0.862	1.028	0.03	0.009	-0.031	0.098
PCDH18	ENSG00000189184		3.4	15.5	0.61	0.355	0.281	0.939	1.00	0.005	1.000	1.000
PCDHB18	ENSG00000146001		-1.8	-3.8	0.61	0.294	0.052	0.698	1.00	0.007	-0.027	0.072
PCGF1	ENSG00000115289		NS	2.2	0.51	0.933	0.195	0.825	1.00	0.005	1.000	1.000
PCTK1	ENSG00000102225		NS	2.3	0.59	0.450	0.270	0.911	1.00	0.005	1.000	1.000
**PDCD1LG2**	ENSG00000197646	**B**	**NS**	**8.6**	0.57	**0.556**	**0.257**	**0.883**	**1.00**	**0.005**	**1.000**	**1.000**
PDCD4	ENSG00000150593		-4.0	-9.0	0.63	0.240	0.036	0.684	1.00	0.009	-0.031	0.098
PDE3B	ENSG00000152270		NS	-5.6	0.51	0.966	0.218	0.792	1.00	0.005	0.000	0.000
PDE8B	ENSG00000113231		-2.9	NS	0.60	0.378	0.067	0.723	0.00	0.005	0.000	0.000
PDK4	ENSG00000004799		-5.7	-50.8	0.63	0.257	0.038	0.692	1.00	0.007	-0.027	0.072

PEA15	ENSG00000162734		2.0	4.8	0.62	0.313	0.290	0.953	1.00	0.005	1.000	1.000
PELI1	ENSG00000197329		2.4	NS	0.98	0.000	0.941	1.019	0.00	0.005	0.000	0.000
PGLS	ENSG00000130313		NS	2.2	0.46	0.737	0.187	0.733	1.00	0.005	1.000	1.000
PHKA2	ENSG00000044446		-1.5	-3.1	0.60	0.378	0.067	0.723	1.00	0.005	0.000	0.000
PIGZ	ENSG00000119227		NS	2.3	0.12	0.001	-0.001	0.231	1.00	0.005	1.000	1.000
PIK3AP1	ENSG00000155629		NS	-5.4	0.56	0.644	0.293	0.817	1.00	0.006	-0.022	0.044
PIK3CG	ENSG00000105851		3.1	NS	0.97	0.000	0.910	1.030	0.01	0.006	-0.022	0.044
PIP5K1C	ENSG00000186111		NS	2.4	0.51	0.933	0.215	0.805	1.00	0.005	1.000	1.000
PKHD1	ENSG00000170927		-4.2	-23.5	0.60	0.401	0.075	0.725	1.00	0.005	0.000	0.000
PKP2	ENSG00000057294		NS	-6.9	0.60	0.401	0.352	0.848	1.00	0.005	0.000	0.000
**PLA2G15**	ENSG00000103066	**B**	**NS**	**5.0**	0.60	**0.425**	**0.272**	**0.918**	**1.00**	**0.005**	**1.000**	**1.000**
**PLAC8**	ENSG00000145287	**A**	**17.6**	**NS**	0.97	**0.000**	**0.897**	**1.033**	**0.04**	**0.010**	**-0.031**	**0.120**
**PLAT**	ENSG00000104368	**A**	**5.6**	**NS**	0.96	**0.000**	**0.883**	**1.037**	**0.09**	**0.021**	**-0.013**	**0.191**
PLCB1	ENSG00000182621		-2.4	-7.5	0.62	0.313	0.047	0.713	1.00	0.005	0.000	0.000
PLCB2	ENSG00000137841		2.4	NS	0.98	0.000	0.941	1.019	0.00	0.005	0.000	0.000
PLCD3	ENSG00000161714		2.4	5.4	0.63	0.294	0.290	0.960	1.00	0.005	1.000	1.000
**PLEK2**	ENSG00000100558	**A**	**4.4**	**NS**	0.98	**0.000**	**0.928**	**1.022**	**0.00**	**0.005**	**0.000**	**0.000**
PLEKHO1	ENSG00000023902		NS	2.3	0.62	0.313	0.305	0.935	0.97	0.009	0.902	1.031
PLIN3	ENSG00000105355		1.7	4.4	0.63	0.294	0.290	0.960	1.00	0.005	1.000	1.000
PLS1	ENSG00000120756		3.4	-4.6	1.00	0.000	0.979	1.011	1.00	0.005	0.000	0.000
PLSCR3	ENSG00000187838		1.8	4.0	0.63	0.294	0.290	0.960	1.00	0.005	1.000	1.000
PLVAP	ENSG00000130300		NS	-3.6	0.42	0.475	0.096	0.734	1.00	0.005	0.000	0.000
PLXDC2	ENSG00000120594		3.4	NS	0.96	0.000	0.897	1.023	0.22	0.117	0.075	0.370
PLXNA2	ENSG00000076356		-2.9	-7.5	0.62	0.294	0.046	0.704	1.00	0.006	-0.022	0.044
PLXND1	ENSG00000004399		2.1	4.0	0.62	0.334	0.284	0.946	1.00	0.005	1.000	1.000
PMP22	ENSG00000109099		2.8	7.1	0.59	0.450	0.269	0.911	1.00	0.005	1.000	1.000
PODNL1	ENSG00000132000		2.3	6.2	0.60	0.425	0.272	0.918	1.00	0.005	1.000	1.000
POF1B	ENSG00000124429		3.4	-4.9	0.95	0.000	0.877	1.024	1.00	0.006	-0.022	0.044
POLR2E	ENSG00000099817		NS	2.7	0.55	0.674	0.244	0.856	0.98	0.007	0.928	1.027
**POPDC3**	ENSG00000132429	**B**	**NS**	**14.8**	0.35	**0.208**	**0.103**	**0.597**	**1.00**	**0.005**	**1.000**	**1.000**
PPP1R15A	ENSG00000087074		NS	3.6	0.31	0.101	0.094	0.516	1.00	0.005	1.000	1.000
PRKCDBP	ENSG00000170955		2.2	NS	0.60	0.425	0.272	0.918	1.00	0.005	1.000	1.000
PRKCQ	ENSG00000065675		NS	-2.5	0.59	0.475	0.333	0.837	1.00	0.005	0.000	0.000
PRPS2	ENSG00000101911		-1.9	-3.8	0.63	0.294	0.040	0.710	1.00	0.005	0.000	0.000
PRR15L	ENSG00000167183		NS	-12.8	0.60	0.401	0.348	0.852	1.00	0.006	-0.022	0.044
**PRR16**	ENSG00000184838	**B**	**NS**	**6.3**	0.57	**0.556**	**0.257**	**0.883**	**1.00**	**0.005**	**1.000**	**1.000**

PRRX1	ENSG00000116132		3.9	16.1	0.60	0.425	0.272	0.919	1.00	0.005	1.000	1.000
PRSS1	ENSG00000204983		NS	-241.8	0.38	0.313	0.047	0.713	1.00	0.005	0.000	0.000
PRSS8	ENSG00000052344		NS	-5.7	0.77	0.023	0.595	0.945	1.00	0.005	0.000	0.000
PSAT1	ENSG00000135069		-6.7	NS	0.91	0.001	-0.043	0.283	0.88	0.033	0.762	0.994
**PSG3**	**ENSG00000221826**	**B**	**NS**	**28.5**	0.35	**0.193**	**0.080**	**0.610**	**1.00**	**0.005**	**1.000**	**1.000**
**PSG5**	**ENSG00000204941**	**B**	**NS**	**62.6**	0.02	**0.000**	**-0.023**	**0.063**	**1.00**	**0.005**	**1.000**	**1.000**
PSMB9	ENSG00000240065		2.6	NS	0.94	0.000	0.847	1.023	0.20	0.091	0.057	0.343
PTCH1	ENSG00000185920		NS	-2.5	0.74	0.044	0.554	0.926	1.00	0.005	0.000	0.000
PTMS	ENSG00000159335		1.6	3.4	0.63	0.294	0.290	0.960	1.00	0.005	1.000	1.000
PTPN14	ENSG00000152104		1.6	3.5	0.61	0.355	0.281	0.939	1.00	0.005	1.000	1.000
PTPN22	ENSG00000134242		2.9	NS	0.95	0.000	0.870	1.030	0.04	0.010	-0.027	0.116
PTPN3	ENSG00000070159		NS	-9.4	0.41	0.450	0.089	0.731	1.00	0.005	0.000	0.000
PTPN6	ENSG00000111679		2.2	NS	0.98	0.000	0.930	1.020	0.00	0.005	0.000	0.000
PTPRE	ENSG00000132334		2.3	NS	0.96	0.000	0.877	1.033	0.07	0.015	-0.021	0.154
**PTRF**	ENSG00000177469	**B**	**NS**	**5.3**	0.57	**0.585**	**0.254**	**0.876**	**1.00**	**0.005**	**1.000**	**1.000**
RAB11FIP1	ENSG00000156675		NS	-5.5	0.68	0.141	0.452	0.898	1.00	0.005	0.000	0.000
RAB23	ENSG00000112210		4.6	13.4	0.63	0.294	0.290	0.960	1.00	0.005	1.000	1.000
RAB25	ENSG00000132698		NS	-5.5	0.97	0.000	0.907	1.023	1.00	0.005	0.000	0.000
**RAB32**	ENSG00000118508	**B**	**NS**	**4.3**	0.55	**0.705**	**0.242**	**0.848**	**1.00**	**0.005**	**1.000**	**1.000**
RAB34	ENSG00000109113		2.6	5.0	0.64	0.240	0.316	0.964	0.97	0.009	0.902	1.031
**RAB3B**	ENSG00000169213	**B**	**NS**	**8.8**	0.55	**0.674**	**0.249**	**0.851**	**0.99**	**0.006**	**0.956**	**1.022**
RAB3IL1	ENSG00000167994		NS	2.7	0.51	0.933	0.218	0.802	1.00	0.005	1.000	1.000
RAD23A	ENSG00000179262		NS	2.1	0.47	0.801	0.194	0.746	1.00	0.005	1.000	1.000
RAP1GAP2	ENSG00000132359		-2.3	-6.0	0.63	0.294	0.040	0.710	1.00	0.005	0.000	0.000
RAPGEF5	ENSG00000136237		NS	-8.7	0.48	0.867	0.183	0.777	1.00	0.005	0.000	0.000
RARG	ENSG00000172819		1.7	3.7	0.63	0.294	0.290	0.960	1.00	0.005	1.000	1.000
**RARRES1**	ENSG00000118849	**A**	**4.7**	**NS**	0.97	**0.000**	**0.910**	**1.030**	**0.17**	**0.060**	**0.035**	**0.299**
**RBM24**	ENSG00000112183	**B**	**NS**	**5.1**	0.15	**0.003**	**0.015**	**0.275**	**1.00**	**0.005**	**1.000**	**1.000**
RBM47	ENSG00000163694		NS	-7.7	0.71	0.078	0.497	0.923	1.00	0.005	0.000	0.000
RCN1	ENSG00000049449		2.5	6.6	0.62	0.313	0.290	0.953	1.00	0.005	1.000	1.000
RCN3	ENSG00000142552		2.4	6.8	0.63	0.294	0.290	0.960	1.00	0.005	1.000	1.000
**RECK**	ENSG00000122707	**B**	**NS**	**11.9**	0.55	**0.674**	**0.244**	**0.856**	**1.00**	**0.005**	**1.000**	**1.000**
REXO2	ENSG00000076043		1.5	5.0	0.59	0.450	0.266	0.914	1.00	0.005	1.000	1.000
**RGMB**	ENSG00000174136	**B**	**NS**	**5.3**	0.09	**0.000**	**-0.025**	**0.195**	**1.00**	**0.005**	**1.000**	**1.000**
**RGS1**	ENSG00000090104	**A**	**4.3**	**NS**	0.97	**0.000**	**0.907**	**1.023**	**0.00**	**0.005**	**0.000**	**0.000**
RGS5	ENSG00000143248		NS	-30.9	0.72	0.065	0.490	0.950	1.00	0.005	0.000	0.000

RHOG	ENSG00000177105		1.8	2.8	0.63	0.294	0.290	0.960	1.00	0.005	1.000	1.000
RHOU	ENSG00000116574		NS	-5.3	0.46	0.705	0.148	0.762	1.00	0.005	0.000	0.000
RIC8A	ENSG00000177963		NS	2.7	0.62	0.334	0.284	0.946	1.00	0.005	1.000	1.000
RILPL1	ENSG00000188026		NS	2.5	0.43	0.556	0.166	0.694	1.00	0.005	1.000	1.000
RNASE1	ENSG00000129538		-2.3	-60.5	0.58	0.475	0.095	0.735	1.00	0.005	0.000	0.000
RNF11	ENSG00000123091		1.5	2.9	0.62	0.334	0.284	0.946	1.00	0.005	1.000	1.000
RNF115	ENSG00000121848		NS	2.3	0.31	0.110	0.077	0.543	1.00	0.005	1.000	1.000
RNF25	ENSG00000163481		NS	2.6	0.58	0.501	0.261	0.899	1.00	0.005	1.000	1.000
RNH1	ENSG00000023191		NS	3.4	0.52	0.867	0.219	0.821	1.00	0.005	1.000	1.000
ROD1	ENSG00000119314		2.3	NS	1.00	0.000	0.979	1.011	0.11	0.028	0.000	0.222
RP1114N72	ENSG00000232527		3.3	18.1	0.59	0.450	0.270	0.911	1.00	0.005	1.000	1.000
RP11223E192	ENSG00000236444		1.5	2.3	0.61	0.355	0.292	0.928	0.98	0.007	0.928	1.027
RP11426L162	ENSG00000234790		NS	2.0	0.63	0.294	0.308	0.942	0.97	0.009	0.902	1.031
RP9P	ENSG00000205763		NS	2.5	0.51	0.933	0.220	0.800	1.00	0.005	1.000	1.000
RPS6KA1	ENSG00000117676		NS	-2.4	0.96	0.000	0.899	1.021	1.00	0.005	0.000	0.000
RRAD	ENSG00000166592		NS	3.8	0.49	0.900	0.188	0.782	1.00	0.005	1.000	1.000
RSBN1	ENSG00000081019		NS	-2.3	0.48	0.867	0.180	0.780	1.00	0.005	0.000	0.000
**RXFP1**	ENSG00000171509	**B**	**NS**	**13.2**	0.16	**0.004**	**-0.006**	**0.316**	**1.00**	**0.005**	**1.000**	**1.000**
RYBP	ENSG00000163602		1.7	2.8	0.63	0.294	0.290	0.960	1.00	0.005	1.000	1.000
RYR2	ENSG00000198626		-3.6	-8.2	0.63	0.294	0.040	0.710	1.00	0.005	0.000	0.000
**S100A3**	ENSG00000188015	**B**	**NS**	**4.3**	0.51	**0.966**	**0.216**	**0.794**	**1.00**	**0.005**	**1.000**	**1.000**
**S100P**	ENSG00000163993	**A**	**7.8**	**NS**	0.96	**0.000**	**0.883**	**1.037**	**0.12**	**0.033**	**0.006**	**0.238**
**SAMD9**	ENSG00000205413	**A**	**5.2**	**NS**	0.96	**0.000**	**0.883**	**1.037**	**0.17**	**0.060**	**0.033**	**0.300**
SCN8A	ENSG00000196876		NS	2.5	0.10	0.001	-0.008	0.208	1.00	0.005	1.000	1.000
SCNM1	ENSG00000163156		1.6	3.7	0.63	0.275	0.300	0.961	0.99	0.006	0.956	1.022
**SDC3**	ENSG00000162512	**B**	**NS**	**4.3**	0.60	**0.425**	**0.272**	**0.918**	**1.00**	**0.005**	**1.000**	**1.000**
**SEMA3D**	ENSG00000153993	**B**	**NS**	**11.2**	0.50	**1.000**	**0.210**	**0.790**	**1.00**	**0.005**	**1.000**	**1.000**
SEMA5A	ENSG00000112902		NS	3.4	0.37	0.275	0.136	0.604	1.00	0.005	1.000	1.000
SEPN1	ENSG00000162430		NS	2.2	0.39	0.355	0.140	0.640	1.00	0.005	1.000	1.000
SEPT09	ENSG00000184640		2.3	3.5	0.63	0.294	0.290	0.960	1.00	0.005	1.000	1.000
SEPT11	ENSG00000138758		2.2	4.3	0.61	0.355	0.281	0.939	1.00	0.005	1.000	1.000
SERPINA1	ENSG00000197249		2.6	-13.6	0.96	0.000	0.886	1.024	1.00	0.006	-0.022	0.044
SERPINA3	ENSG00000196136		NS	-59.9	0.54	0.769	0.259	0.811	1.00	0.007	-0.027	0.072
**SERPINB5**	**ENSG00000206075**	**A**	**10.2**	**NS**	1.00	**0.000**	**0.999**	**1.000**	**0.01**	**0.006**	**-0.022**	**0.044**
**SERPINE2**	**ENSG00000135919**	**B**	**NS**	**39.1**	0.61	**0.378**	**0.278**	**0.932**	**1.00**	**0.005**	**1.000**	**1.000**
SERPINH1	ENSG00000149257		4.6	9.6	0.63	0.294	0.290	0.960	1.00	0.005	1.000	1.000

SESN2	ENSG00000130766		-1.6	2.4	0.98	0.000	-0.022	0.072	1.00	0.005	1.000	1.000
SFMBT2	ENSG00000198879		2.5	NS	0.98	0.000	0.930	1.020	0.00	0.005	0.000	0.000
**SFN**	ENSG00000175793	**A**	**5.5**	**NS**	1.00	**0.000**	**0.999**	**1.000**	**0.11**	**0.028**	**0.000**	**0.222**
SGCB	ENSG00000163069		1.8	4.2	0.61	0.378	0.278	0.933	1.00	0.005	1.000	1.000
**SGCD**	ENSG00000170624	**B**	**NS**	**7.8**	0.56	**0.644**	**0.247**	**0.863**	**1.00**	**0.005**	**1.000**	**1.000**
SGPP2	ENSG00000163082		3.5	NS	0.95	0.000	0.870	1.030	0.03	0.009	-0.031	0.098
SGTB	ENSG00000197860		1.8	3.6	0.63	0.294	0.290	0.960	1.00	0.005	1.000	1.000
SH3YL1	ENSG00000035115		-2.1	-4.9	0.63	0.257	0.038	0.692	1.00	0.007	-0.027	0.072
SHC1	ENSG00000160691		NS	2.4	0.63	0.294	0.290	0.960	1.00	0.005	1.000	1.000
SHC3	ENSG00000148082		-3.0	NS	0.99	0.000	-0.016	0.036	0.99	0.006	0.956	1.022
SHISA4	ENSG00000198892		NS	3.0	0.39	0.355	0.130	0.650	1.00	0.005	1.000	1.000
SHISA5	ENSG00000164054		2.0	NS	0.63	0.294	0.290	0.960	1.00	0.005	1.000	1.000
**SHOX2**	ENSG00000168779	**B**	**NS**	**4.6**	0.20	**0.012**	**0.025**	**0.375**	**1.00**	**0.005**	**1.000**	**1.000**
SLAMF8	ENSG00000158714		3.4	NS	0.97	0.000	0.910	1.030	0.04	0.010	-0.029	0.118
SLC16A10	ENSG00000112394		-5.2	-26.8	0.61	0.334	0.054	0.716	1.00	0.005	0.000	0.000
SLC37A1	ENSG00000160190		2.4	NS	1.00	0.000	0.999	1.000	0.00	0.005	0.000	0.000
SLC38A6	ENSG00000139974		1.9	3.7	0.60	0.425	0.272	0.919	1.00	0.005	1.000	1.000
SLC38A7	ENSG00000103042		NS	3.0	0.51	0.966	0.207	0.803	1.00	0.005	1.000	1.000
SLC39A13	ENSG00000165915		NS	3.4	0.54	0.769	0.235	0.835	1.00	0.005	1.000	1.000
**SLC44A4**	ENSG00000204385	**A**	**7.2**	**-4.3**	0.98	**0.000**	**0.923**	**1.027**	1.00	**0.005**	**0.000**	**0.000**
SLC47A1	ENSG00000142494		NS	3.6	0.53	0.834	0.231	0.819	0.99	0.006	0.956	1.022
**SLC6A14**	ENSG00000087916	**A**	**34.6**	**NS**	0.99	**0.000**	**0.951**	**1.019**	**0.03**	**0.009**	**-0.028**	**0.095**
SLC7A2	ENSG00000003989		-6.2	-48.8	0.63	0.294	0.040	0.710	1.00	0.005	0.000	0.000
SLFN13	ENSG00000154760		2.8	NS	1.00	0.000	0.999	1.000	0.01	0.006	-0.022	0.044
**SLPI**	ENSG00000124107	**A**	**10.3**	**NS**	0.97	**0.000**	**0.910**	**1.030**	**0.01**	**0.006**	**-0.022**	**0.044**
**SMPD1**	ENSG00000166311	**B**	**NS**	**4.9**	0.45	**0.674**	**0.166**	**0.734**	**1.00**	**0.005**	**1.000**	**1.000**
**SNAI1**	ENSG00000124216	**B**	**NS**	**9.9**	0.52	**0.867**	**0.224**	**0.816**	**1.00**	**0.005**	**1.000**	**1.000**
**SNAI2**	ENSG00000019549	**B**	**NS**	**11.2**	0.55	**0.705**	**0.241**	**0.849**	**1.00**	**0.005**	**1.000**	**1.000**
SNAPIN	ENSG00000143553		NS	2.4	0.58	0.501	0.263	0.897	1.00	0.005	1.000	1.000
SNX9	ENSG00000130340		1.6	3.3	0.60	0.401	0.274	0.926	1.00	0.005	1.000	1.000
SORL1	ENSG00000137642		1.9	-9.3	0.97	0.000	0.910	1.032	1.00	0.005	0.000	0.000
SPARCL1	ENSG00000152583		NS	-36.0	0.94	0.000	0.854	1.016	1.00	0.005	0.000	0.000
SPATA18	ENSG00000163071		NS	4.0	0.47	0.769	0.191	0.739	1.00	0.005	1.000	1.000
SPINK1	ENSG00000164266		NS	-312.0	0.38	0.313	0.047	0.713	1.00	0.005	0.000	0.000
SPINT1	ENSG00000166145		NS	-4.6	0.93	0.000	0.834	1.016	1.00	0.005	0.000	0.000
SPNS1	ENSG00000169682		NS	2.4	0.55	0.705	0.235	0.855	1.00	0.005	1.000	1.000

SPON2	ENSG00000159674		3.4	10.0	0.60	0.401	0.274	0.926	1.00	0.005	1.000	1.000
SREBF2	ENSG00000198911		NS	2.2	0.55	0.674	0.255	0.845	0.98	0.007	0.928	1.027
SRGAP1	ENSG00000196935		NS	2.4	0.51	0.966	0.217	0.793	1.00	0.005	1.000	1.000
ST14	ENSG00000149418		NS	-8.3	0.90	0.001	0.795	1.005	1.00	0.005	0.000	0.000
**ST6GALNAC1**	ENSG00000070526	**A**	**4.4**	**NS**	1.00	**0.000**	**0.979**	**1.011**	**0.06**	**0.012**	**-0.035**	**0.146**
ST8SIA2	ENSG00000140557		NS	2.2	0.44	0.614	0.167	0.713	1.00	0.005	1.000	1.000
ST8SIA4	ENSG00000113532		3.1	NS	0.98	0.000	0.923	1.027	0.00	0.005	0.000	0.000
**STAC**	ENSG00000144681	**B**	**NS**	**5.8**	0.56	**0.644**	**0.247**	**0.863**	**1.00**	**0.005**	**1.000**	**1.000**
STC2	ENSG00000113739		-3.2	NS	0.97	0.000	-0.022	0.082	1.00	0.005	1.000	1.000
STK3	ENSG00000134602		2.5	NS	1.00	0.000	0.979	1.011	0.08	0.017	-0.016	0.172
STK4	ENSG00000101109		2.2	NS	0.99	0.000	0.965	1.016	0.19	0.080	0.050	0.328
STON2	ENSG00000140022		NS	3.7	0.55	0.705	0.239	0.851	1.00	0.005	1.000	1.000
SWAP70	ENSG00000133789		NS	2.4	0.55	0.674	0.244	0.856	1.00	0.005	1.000	1.000
**SYDE1**	ENSG00000105137	**B**	**NS**	**4.1**	0.58	**0.529**	**0.259**	**0.891**	**1.00**	**0.005**	**1.000**	**1.000**
**SYNC**	ENSG00000162520	**B**	**NS**	**7.7**	0.54	**0.737**	**0.238**	**0.842**	**1.00**	**0.005**	**1.000**	**1.000**
SYT11	ENSG00000132718		2.3	NS	0.61	0.355	0.281	0.939	1.00	0.005	1.000	1.000
TAF6	ENSG00000106290		NS	2.1	0.62	0.334	0.302	0.928	0.97	0.009	0.902	1.031
TAP1	ENSG00000168394		3.9	NS	0.96	0.000	0.883	1.037	0.20	0.091	0.057	0.343
TAP2	ENSG00000204267		2.3	NS	0.96	0.000	0.889	1.021	0.18	0.069	0.042	0.313
TBC1D26	ENSG00000214946		-2.1	NS	1,00	0.000	0.000	0.000	0.83	0.060	0.700	0.967
TBC1D30	ENSG00000111490		-3.2	-7.8	0.63	0.294	0.040	0.710	1.00	0.005	0.000	0.000
TBCB	ENSG00000105254		1.8	3.1	0.63	0.275	0.305	0.955	0.98	0.007	0.928	1.027
**TBX15**	ENSG00000092607	**B**	**NS**	**11.8**	0.48	**0.867**	**0.120**	**0.760**	**1.00**	**0.005**	**1.000**	**1.000**
**TBX18**	ENSG00000112837	**B**	**NS**	**11.0**	0.53	**0.834**	**0.228**	**0.822**	**1.00**	**0.005**	**1.000**	**1.000**
TBXAS1	ENSG00000059377		2.4	-2.7	0.96	0.000	0.895	1.025	1.00	0.006	-0.022	0.044
TC2N	ENSG00000165929		-2.7	-121.6	0.62	0.313	0.047	0.713	1.00	0.005	0.000	0.000
TESC	ENSG00000088992		NS	-9.7	0.61	0.378	0.361	0.849	1.00	0.005	0.000	0.000
TFCP2L1	ENSG00000115112		NS	-5.1	0.61	0.355	0.368	0.852	1.00	0.005	0.000	0.000
TFDP1	ENSG00000198176		1.7	2.5	0.61	0.378	0.281	0.932	1.00	0.005	1.000	1.000
TFE3	ENSG00000068323		1.5	2.7	0.62	0.313	0.290	0.953	1.00	0.005	1.000	1.000
TFEC	ENSG00000105967		3.7	NS	0.98	0.000	0.941	1.020	0.00	0.005	0.000	0.000
**TGM2**	ENSG00000198959	**A**	**5.8**	**NS**	0.96	**0.000**	**0.883**	**1.037**	**0.18**	**0.069**	**0.041**	**0.314**
THY1	ENSG00000154096		6.3	18.9	0.62	0.313	0.290	0.953	1.00	0.005	1.000	1.000
TIMP2	ENSG00000035862		2.7	6.4	0.60	0.425	0.272	0.919	1.00	0.005	1.000	1.000
TLE4	ENSG00000106829		NS	2.9	0.51	0.933	0.219	0.801	1.00	0.005	1.000	1.000
TLR6	ENSG00000174130		3.1	NS	0.96	0.000	0.883	1.037	0.14	0.045	0.002	0.287

TM4SF4	ENSG00000169903		NS	-33.9	0.66	0.193	0.432	0.878	1.00	0.006	-0.022	0.044
**TMC5**	ENSG00000103534	**A**	**10.5**	**NS**	0.98	**0.000**	**0.923**	**1.027**	**0.00**	**0.005**	**0.000**	**0.000**
**TMEFF1**	ENSG00000241697	**B**	**NS**	**5.4**	0.64	**0.257**	**0.314**	**0.956**	**0.97**	**0.009**	**0.902**	**1.031**
TMEM104	ENSG00000109066		NS	3.2	0.54	0.737	0.217	0.863	1.00	0.005	1.000	1.000
**TMEM119**	ENSG00000183160	**B**	**NS**	**8.2**	0.54	**0.769**	**0.240**	**0.835**	**1.00**	**0.005**	**1.000**	**1.000**
TMEM138	ENSG00000149483		1.4	3.7	0.59	0.450	0.266	0.914	1.00	0.005	1.000	1.000
TMEM184B	ENSG00000198792		2.1	4.3	0.63	0.294	0.290	0.960	1.00	0.005	1.000	1.000
TMEM189	ENSG00000240849		2.6	5.3	0.63	0.294	0.290	0.960	1.00	0.005	1.000	1.000
TMEM30B	ENSG00000182107		NS	-4.6	0.58	0.529	0.317	0.833	1.00	0.005	0.000	0.000
TMEM39B	ENSG00000121775		NS	2.3	0.49	0.933	0.198	0.782	1.00	0.005	1.000	1.000
**TMEM45B**	ENSG00000151715	**A**	**7.2**	**NS**	0.96	**0.000**	**0.886**	**1.024**	**0.01**	**0.006**	**-0.022**	**0.044**
TMEM51	ENSG00000171729		NS	-3.7	0.52	0.900	0.233	0.797	1.00	0.005	0.000	0.000
TMEM55A	ENSG00000155099		1.9	3.8	0.61	0.378	0.278	0.933	1.00	0.005	1.000	1.000
TMEM56	ENSG00000152078		NS	-6.8	0.41	0.425	0.082	0.728	1.00	0.005	0.000	0.000
TMOD1	ENSG00000136842		NS	-9.9	0.39	0.355	0.061	0.719	1.00	0.005	0.000	0.000
TMPRSS2	ENSG00000184012		NS	-21.2	0.41	0.450	0.088	0.732	1.00	0.005	0.000	0.000
**TMPRSS4**	**ENSG00000137648**	**A**	**20.9**	**NS**	1.00	**0.000**	**0.979**	**1.011**	**0.07**	**0.015**	**-0.021**	**0.154**
TMTC3	ENSG00000139324		2.0	3.6	0.63	0.275	0.300	0.961	0.99	0.006	0.956	1.022
TNC	ENSG00000041982		4.8	25.0	0.61	0.355	0.281	0.939	1.00	0.005	1.000	1.000
**TNFRSF11B**	**ENSG00000164761**	**B**	**NS**	**31.8**	0.58	**0.529**	**0.259**	**0.890**	**1.00**	**0.005**	**1.000**	**1.000**
TNFRSF21	ENSG00000146072		3.1	NS	1.00	0.000	0.999	1.000	0.08	0.017	-0.018	0.173
TNFSF13B	ENSG00000102524		2.3	NS	0.93	0.000	0.833	1.027	0.03	0.009	-0.031	0.098
TNFSF15	ENSG00000181634		2.9	NS	0.99	0.000	0.964	1.016	0.14	0.045	0.002	0.287
TNFSF9	ENSG00000125657		NS	2.5	0.18	0.006	0.019	0.331	1.00	0.005	1.000	1.000
TNIK	ENSG00000154310		2.6	NS	1.00	0.000	0.999	1.000	0.08	0.017	-0.019	0.175
TOM1	ENSG00000100284		1.6	2.6	0.60	0.401	0.275	0.925	1.00	0.005	1.000	1.000
TOR1A	ENSG00000136827		1.5	2.2	0.63	0.294	0.290	0.960	1.00	0.005	1.000	1.000
TPD52	ENSG00000076554		NS	-5.6	0.74	0.044	0.552	0.928	1.00	0.005	0.000	0.000
TPP1	ENSG00000166340		1.7	3.5	0.59	0.450	0.269	0.911	1.00	0.005	1.000	1.000
TRAM2	ENSG00000065308		2.3	8.8	0.63	0.294	0.290	0.960	1.00	0.005	1.000	1.000
TRIM14	ENSG00000106785		2.0	NS	1.00	0.000	0.999	1.000	0.10	0.024	-0.006	0.206
**TSPAN1**	ENSG00000117472	**A**	**22.5**	**NS**	0.97	**0.000**	**0.897**	**1.033**	**0.02**	**0.007**	**-0.027**	**0.072**
TSPAN15	ENSG00000099282		2.6	-3.8	0.97	0.000	0.916	1.024	1.00	0.005	0.000	0.000
TSPAN5	ENSG00000168785		3.1	11.0	0.62	0.313	0.290	0.953	1.00	0.005	1.000	1.000
TSPAN7	ENSG00000156298		NS	-12.2	0.48	0.867	0.183	0.777	1.00	0.005	0.000	0.000
TSPAN8	ENSG00000127324		4.8	-19.0	0.95	0.000	0.862	1.028	1.00	0.009	-0.031	0.098

TTC17	ENSG00000052841		-1.5	-2.2	0.62	0.257	0.044	0.686	1.00	0.009	-0.031	0.098
TTL	ENSG00000114999		1.8	2.9	0.63	0.294	0.290	0.960	1.00	0.005	1.000	1.000
TTLL1	ENSG00000100271		NS	3.1	0.56	0.644	0.246	0.864	1.00	0.005	1.000	1.000
TTYH3	ENSG00000136295		2.5	4.0	0.63	0.294	0.290	0.960	1.00	0.005	1.000	1.000
TUBA1A	ENSG00000167552		2.4	NS	0.62	0.334	0.284	0.946	1.00	0.005	1.000	1.000
TUBA1C	ENSG00000167553		3.7	7.7	0.64	0.240	0.316	0.964	0.97	0.009	0.902	1.031
**TUBB2A**	ENSG00000137267	**B**	**NS**	**6.1**	0.26	**0.040**	**0.069**	**0.441**	**1.00**	**0.005**	**1.000**	**1.000**
TUBG2	ENSG00000037042		-2.3	NS	1,00	0.000	0.000	0.000	0.93	0.015	0.844	1.023
TULP3	ENSG00000078246		NS	2.3	0.34	0.166	0.123	0.547	0.97	0.009	0.902	1.031
TWSG1	ENSG00000128791		2.3	6.1	0.61	0.355	0.281	0.939	1.00	0.005	1.000	1.000
TXNIP	ENSG00000117289		NS	-3.3	0.88	0.002	0.757	0.993	1.00	0.009	-0.031	0.098
TXNRD1	ENSG00000198431		2.7	5.7	0.63	0.294	0.290	0.960	1.00	0.005	1.000	1.000
UBAC2	ENSG00000134882		1.5	2.1	0.63	0.294	0.290	0.960	1.00	0.005	1.000	1.000
UBAP2L	ENSG00000143569		NS	2.1	0.58	0.529	0.274	0.876	0.97	0.009	0.902	1.031
**UBE2E2**	ENSG00000182247	**B**	**NS**	**4.4**	0.58	**0.529**	**0.266**	**0.884**	**0.99**	**0.006**	**0.956**	**1.022**
UBE2L3	ENSG00000185651		1.5	2.5	0.61	0.355	0.293	0.927	0.98	0.007	0.928	1.027
UGT8	ENSG00000174607		NS	-2.5	0.81	0.010	0.649	0.961	1.00	0.005	0.000	0.000
VAMP8	ENSG00000118640		NS	-14.6	0.88	0.002	0.713	1.037	1.00	0.005	0.000	0.000
**VASN**	ENSG00000168140	**B**	**NS**	**4.3**	0.48	**0.867**	**0.202**	**0.758**	**1.00**	**0.005**	**1.000**	**1.000**
VAT1	ENSG00000108828		1.5	5.4	0.60	0.425	0.272	0.919	1.00	0.005	1.000	1.000
VDR	ENSG00000111424		3.7	7.4	0.61	0.355	0.281	0.939	1.00	0.005	1.000	1.000
**VEGFC**	ENSG00000150630	**B**	**NS**	**5.1**	0.52	**0.900**	**0.213**	**0.817**	**1.00**	**0.005**	**1.000**	**1.000**
**VGLL3**	ENSG00000206538	**B**	**NS**	**10.8**	0.53	**0.801**	**0.232**	**0.828**	**1.00**	**0.005**	**1.000**	**1.000**
VIM	ENSG00000026025		NS	2.9	0.58	0.529	0.259	0.891	1.00	0.005	1.000	1.000
**VIT**	ENSG00000205221	**B**	**NS**	**5.3**	0.11	**0.001**	**-0.004**	**0.224**	**1.00**	**0.005**	**1.000**	**1.000**
VTCN1	ENSG00000134258		-3.3	-13.4	0.61	0.355	0.061	0.719	1.00	0.005	0.000	0.000
**WASF1**	ENSG00000112290	**B**	**NS**	**4.1**	0.57	**0.556**	**0.265**	**0.875**	**0.98**	**0.007**	**0.928**	**1.027**
WASF3	ENSG00000132970		NS	2.5	0.05	0.000	-0.019	0.119	1.00	0.005	1.000	1.000
WBP2	ENSG00000132471		NS	2.2	0.55	0.674	0.244	0.856	1.00	0.005	1.000	1.000
XAF1	ENSG00000132530		3.3	NS	0.92	0.000	0.800	1.030	0.17	0.060	0.033	0.300
**XG**	ENSG00000124343	**B**	**NS**	**13.4**	0.14	**0.002**	**0.009**	**0.261**	**1.00**	**0.005**	**1.000**	**1.000**
XPO6	ENSG00000169180		1.6	2.7	0.62	0.334	0.300	0.934	0.98	0.007	0.928	1.027
ZBTB4	ENSG00000174282		NS	2.3	0.51	0.966	0.215	0.795	1.00	0.005	1.000	1.000
ZCCHC24	ENSG00000165424		NS	2.6	0.59	0.450	0.269	0.911	1.00	0.005	1.000	1.000
ZDHHC20	ENSG00000180776		2.2	NS	1.00	0.000	0.999	1.000	0.14	0.045	0.020	0.269
ZFYVE1	ENSG00000165861		NS	2.6	0.57	0.556	0.250	0.890	1.00	0.005	1.000	1.000

ZMAT3	ENSG00000172667	2.0	5.5	0.59	0.475	0.266	0.904	1.00	0.005	1.000	1.000
ZNF204P	ENSG00000204789	NS	-7.4	0.44	0.585	0.124	0.746	1.00	0.005	0.000	0.000
ZNF33B	ENSG00000196693	-1.9	-5.8	0.61	0.334	0.054	0.716	1.00	0.005	0.000	0.000
ZNF518A	ENSG00000177853	NS	-3.3	0.73	0.053	0.526	0.934	1.00	0.005	0.000	0.000
ZNF828	ENSG00000198824	NS	2.1	0.11	0.001	-0.004	0.214	0.97	0.009	0.902	1.031
ZNF860	ENSG00000197385	3.7	NS	1.00	0.000	0.999	1.000	0.09	0.021	-0.011	0.189
ZYX	ENSG00000159840	2.0	4.4	0.62	0.313	0.290	0.953	1.00	0.005	1.000	1.000

q-values<.001; AUC: area under the curve; CI: confidence interval; *the most up-regulated transcripts (fold change≥4) that discriminates between each of the GEP-A and GEP-B subgroup of PDAC tumors vs. pancreatic non-tumoral tissues are displayed in bold .
